# The sustainable use of diverse plants accustomed by different ethnic groups in Sibi District, Balochistan, Pakistan

**DOI:** 10.1371/journal.pone.0294989

**Published:** 2024-02-21

**Authors:** Bibi Maria, Shazia Saeed, Alia Ahmed, Maria Ahmed, Abdul Rehman

**Affiliations:** 1 Department of Botany, University of Balochistan, Quetta, Pakistan; 2 Department of URSMIT and FAHS, University of Lahore, Lahore, Pakistan; CIFRI: Central Inland Fisheries Research Institute, INDIA

## Abstract

The present study was conducted to analyze the utilization of medicinal plants (traditional as well as cultivated) and there recipes accustomed by different ethnic groups of Sibi District (SD), Balochistan, Pakistan. The study was carried out between 2018 and 2021 by using semi-structured and open-ended questionnaire.. The randomly selected methods applied for this study were mainly based on household surveys walk through and interview with indigenous communityage 40 to 80, a total of 75 plants, belonging to 63 genera and distributed among 33 plant families were recorded. The dominant Plant families were the Fabaceae (12%) of all studied taxa, followed by the Amaranthaceae (7%), Asteraceae (6%), Cucurbitaceae, Solanaceae, Poaceae (4% each), Rhamnaceae and Zygophyllaceae (3%). Thirty traditional Food Recipes (TFR) and Traditional Medicinal Recipes (TMR) were novel being first time reported from SD., which are utilized by the local communities in their daily routine. These ethnic TFR and TMR have a tremendous role in preservation and sustainable use of traditional food habits and culture. It was also documented that along with cultivated, the wild edible and medicinal plant preparations play a significant role in in the economic potential and primary health care system of the local communities. The study recommends the specific measures, such as small industries, improved export means, tourism and educational activities, to protect the traditional knowledge and biocultural heritage of the region before its erosion.

## Introduction

Plant are the basic component of human life, which are used in different ways such as medicine, food, cloths, shelter, and other different products [1]. Distinct knowledge and practices of diverse cultures, ethnic groups and religions that live within the same environment are interesting to provide an insight in better understanding of human interaction with in the ecosystem and use of the resources that can be utilized either in the same manner or differently [2]. The local people possess plenty of traditional knowledge about the wild edible plants and use to transmit it verbally to generations. The relationship between human communities and use of plants has been considered as an ecological balance system since ancient times to preserve this resource. Therefore, in this domain of ethnobotany, it is aimed to investigate and document different wild plants being used by ethnic groups of various localities for nutrition and economic purposes [3]. Historically, women have been considered to be important repositories of plant knowledge, and considered to play an important role in the maintenance of knowledge on the use of plant resources [4]. Women recognize more useful plant species than men, and that women’s knowledge is more homogeneous [5]. The medicinal plant knowledge is being transferred and retained, perhaps not between mother and daughter as in the past but between two groups with a personal interest in maintaining it [6].

The developed countries of Europe wildly use wild edible plant (WEP) and consider it as an important factor of ecosystem [7]. Numbers of ethnobotanical researches have proven the importance of wild edible plants for the local communities especially in emergency situations like war, famine and drought throughout the world [8]. Along with the practice of modern agricultural techniques, the local communities also have dependence on the wild edible plants (WEPs) up to a great extent. These wild plants are satisfying the food security issues by providing alternative and diverse food sources to the traditional communities of the area [9]. Beside the importance of plants as edible, their pharmacological properties have also been proven by many researches throughout the world [10, 11]. Due to the existence of many bioactive compounds like fatty acids, complex sugars, vitamins and proteins, the WEPs can be used to cope with the malnutrition problems [12]. The phytochemical constituents of many plants have been investigated before on the basis of medicinal and nutritional aspects [13, 14].

Ethnobotanical knowledge of Wild Food Plants (WFP’s has been transferred from generations. Wild Food Plants (WFP’s) have remained an important resource since ancient times. Historically, local and traditional Food systems have given sufficient space to WFP’s and their existence in daily food practice among local communities, could be a parameter to qualitatively measure the socio-cultural histories and economic instabilities of different communities over time and space [15]. Though, the present world food system is capable enough in provision of food for human, but, people around the world still lacking nutritious food or experience hunger. On the other hand, highly processed food also affected the human health. Malnutrition is considered another global threat, in addition to climate change, specifying an urgent need for more sustainable food system. WFP’s thus, can be a vital constituent in people’s dietary system around the globe. These food plants are also eaten for their robust health-giving properties, and many other species are commonly used as herbal medicines in primary health care system [16]. Due to their clearly positive influence on health, they are often identified as functional foods, thanks to their higher contents of vitamins, phenols, flavonoids, antioxidants, microelements, and fiber than in cultivated crops. Wild plants are also perceived as a healthy alternative to cultivated vegetables that might be rich in pesticides and other chemicals. Therefore, wild species may have great potential as sources of unusual colors and flavors, bioactive compounds, and of dietary supplements.

Pakistan is ranked 6th among the over populated countries of the world with very low income. Being an underdeveloped country, the alternative food resources are needed for the people. It has been estimated that around 60% of the population of the country is food insecure [17]. Though, the country has been blessed by the four seasons, Hence, rich in wide diversity of natural resources, even ranked 11th most food insecure country worldwide [18]. Overpopulation, poverty, less availability of food resources and local livelihood strategies are considered as major issues of food insecurity. The wild edible plants (WEPs) can be used as alternative food resource for local communities to compete with the issues of hunger and malnutrition if managed properly [19].

The variables known to affect medicinal plant knowledge include education, occupation, age, gender and psychosocial variables [20, 21]. Age and gender are generally the factors most examined for their influence on knowledge about plants. In different areas knowledge regarding medicinal plants play key role in their medical system [22]. Much of this knowledge is traditional, that is, learned long ago and passed on with varying degrees of faithfulness for at least two or three generations. From different areas of Pakistan, different studies have been reported on medicinal plants [23–25] as well as from Balochistan the wild edible plants reported earlier by Aziz and his research team [26]. The purpose of this study was to explore the bio-culture diversity of a region, the SD which is famous for its harsh climate, ethnic and religious rituals and utilization of the wild and cultivated flora for daily subsistence and for their primary health care system. In this regard, women, especially above the age of forty five years are playing a vital role in livelihood and sustaining bio-culture diversity. Unfortunately, no data of the area regarding bio-culture, indigenous knowledge of the community or the valuable recipes accustomed by the women of the area was documented. This is the first report from the SD, Balochistan, Pakistan, highlighting the bio-culture diversity role of indigenous women of various ethnic groups and the exploitation of the recipes accustomed by them before its erosion.

## Materials and methods

### Study area and climatic conditions

The field study was carried out in eight villages of SD, Balochistan province of Pakistan. The landscape of the area is characterized by planes. It is also known as the **"Hot spot"** of Pakistan where the temperatures in the summer exceed 52.6°C (126.7°F) in the month of June. Precipitation is light and mainly falls in two distinct periods: early spring in March and April, and during monsoon season in July and August. Total geographical attributes are 7121 square kilometer area, at 29° 33’ N 67° 53 E, elevation 130 meter above sea level.

### Biological diversity and communities

**Fig 1:**
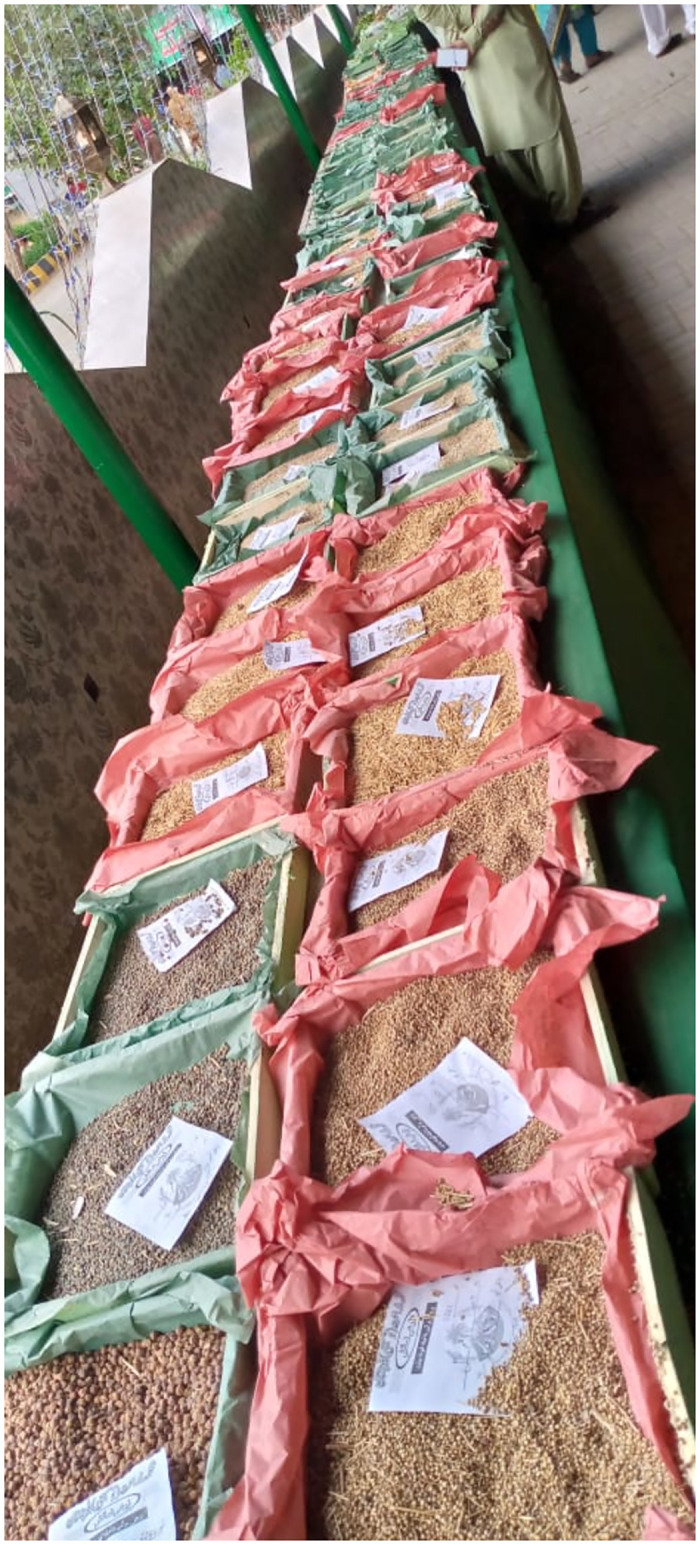
Stall of different agricultural crops seeds display in Sibi Mela 2023.

Sibi district is a hub of diverse communities and ethnic groups, most of them are Baloch, its sub tribes are Rind, Marri, Silachi speak Balochi, other communities are Pashto (Khajjak, Barozai, Lohni), Sindhi (Soomro), Siraiki, Biravhi (Bangulzai), Panjabi and Urdu speaking people. In ancient times, many Baloch tribes were pastoralists, herding goats and sheep; many were nomadic (Spooner 1988). Hindu communities are also living since centuries, contributing in bio-culture conservation. The origin of the town’s name (SIBI) is ascribed to Rani Sewi, a Hindu lady of the “Sewa Empire” who ruled Balochistan before the 7th century. Sindhi language is adopted language from Hindus. “Sibi Mela” is a cultural festival that has been regularly celebrated every year in early spring and has been observed since the 18th century. This is one the popular festival of the region; people come across the country and celebrate their bio-culture. Mainly the livestock and agriculture are the main focus of the event (Fig 1). Many livestock breeders gather every spring at Sibi town for sale and purchase, competition, and display of various breeds of camels, cattle, and goats. The residents spend six months to prepare for the festival and the remaining six months to remember it. Besides, cultural festivities, folk music, traditional food and handmade folk embroidery are popular and exported during this event.

### Ethnobotanical survey

Approval of the research has been granted by the Advance studies & Research Board, University of Balochistan, Quetta Pakistan under the Reg. No. 2011/UB-2018/Q-3776 dated 26-03-2018. Field survey was conducted in August 2018–21 by means of semi-structured interviews with the help of Questionnaire (S1 File) from household women, participant observation, as well as market survey. Random method is used for data collection. Eight villages share the boundaries of Sibi were selected for this study are as follow:

**i. Mall village**:
  Situated 20 kilometers (KM) from Sibi city (SC), Mall, Ghishkori, Chandio tribe are settled here basically they are known as Baloch community. Balochi is their speaking language whereas the Chandio tribe speaks in Sindhi language.
**ii. Kurak Village**:
  It is situated from 7KM from SC, the living Communities are Ghuramzai (Bangulzai), Ghulam bolak (sub-tribe of Rind). They are settled in Bakhraro with other tribes of Baloch (Ghorchani, Lashari, Patafi, Chandio, Gorgaige).
**iii. Bakhro Ghulam Bulak Village**:
  It is situated 8.2 KM from SC, mostly Balochi language is their speaking medium.
**iv. Gulu Shar Village**:
  Situated at 9.2KM distance from SC, it is another village of Baloch community.
**v. Talli Village**:
  It is situated at 27KM awayfrom SC. The dominant community of Talli is Silachi basically they are Tareen and they speak Sindhi and Balochi language.
**vi. Luni Villages**:
  It is situated at 11KM north of city of Sibi, Luni village Tribes are Pashto speaking. Lunni tribe migrated in 1660s from Afghanstan (Muqor) and settled in Sibi.
**vii. Khajjak Village**:
  It is situated far away 14KM from SC. Most of the people of Khajjak speak Pashto language, some of them have adopted Sindhi language. Khajjak village is very rich in wheat cultivation. According to the Khajjak’s elder they lived in Mekhtar area nowadays that area is the part of the district Loralai. About 1700 A.D, their elders migrated to Sibi with their livestock.
**viii. Marghzani Village**:
  It is situated at 6.2KM away from SC people of Marghzani are Pastoon. In 1511 the Arghon Government constructed Sibi Fort twice, after the decline of Arghoon era. In 1575 the Panni tribe (Dahpal, Barozai and Marghazani) were settled around the fort. Dhapal is now become small town, community of dahpal is pastoon community. And few tribes of panni are settled in Dhapal.

The recommendations provided by the International Society of Ethnobiology [27] were strictly followed before the data collection. The informants consent was taken to share their knowledge during interviews and photos for the completion of the study. As many informants’ (Women) were villagers adapting the culture of secluding, so special precautions and measures i.e., interviewers were also women so the informants were quite comfortable, elders also allowed to publish the data with their photographs. Furthermore, Informants were comfortable in their local languages so the data was recorded in their languages and further translated into Urdu.

The interviews focused on TFR & WFPs, i.e., plants used as domestic food, cooked vegetables, snacks, pickles, seasoning and other selling products of these plants. As well as the medicinal use of the plants are used as household remedies of the women. Each plant was recorded along with the local name, recorded plants were collected, and voucher specimens were identified by Author two. Plants were further identified with the help of Plants of the World Online [28]. The voucher specimens were submitted in the open-herbarium of Botanical Garden (OHBG), University of Balochistan Quetta. Accession numbers of OHBG were provided after verification of plant.

## Results and discussion

The present study is the first document which describes the checklist of wild and cultivated plants of SD and their ethnobotanical uses with traditional recipes and products prepared by household women of the area, utilizing the ancient bio-culture traditions not only for their own subsistence but also for improving their statuses after generating some income. The study was carried out between 2018 and 2021 by using semi-structured, open-ended questionnaire. Five ethnic groups, i.e., Pashto, Balochi, Sindhi, Siraiki and Biravhi along with Hindus are the participating in sustainable bio-culture and food security in the region. A total of 75 plants, belonging to 63 genera and distributed among 33 plant families were recorded. The women shared their valuable knowledge about daily used food plants (FP’s), medicinally important plants with recipes and other customary utilization of bio-culture. The uses of plants by local people since ancient times play a vital role in food security [29]. The plants quoted by the informants, Table 1, are grouped in to two major types of wild and cultivated, comprising of 62% and 32% respectively, whilst few are naturalized. Furthermore, the documented plant species (Table 1) of the area revealed that herbs and trees are the major life form comprising of 56% and 24% respectively, followed by 12% shrubs, 4% grasses 3% under shrubs and only one reed taxon. The major use categories of documented plants are grouped into different categories of multipurpose or only for a single purpose including medicinally important, Food plants, fodder, fuel or commercially used plants [30, 31]. Many plants documented were grouped as multipurpose and constituting 77% of the total plants, remaining are used as for a single category such as edible, fodder cosmetic or weed etc.

**Table 1:** List of plants collected from SD with their accession numbers and relevant details.

Family	Plant Botanical Name	Local Name	Plant type	Life form	Parts used	Major use category
Amaranthaceae	*Aerva javanica* (Burm.f.) Juss. ex. Schult. QUETTA000051	Bal Buh	Wild	Herb	Flower Leaves stem	Commercial, fodder, fuel
=	*A*. *javanica* var. *bovei* Webb QUETTA000475	Surkhri	Wild	Herb	Flower Stem	Cosmetic, fuel
=	*Amaranthus viridis* L. QUETTA000128	Sarmi	Wild	Herb	Whole plant	Weed, vegetable
=	*Chenopodium album* L. QUETTA000021	Kalpir	Wild	Herb	Whole plant	Weed, vegetable, medicinal
=	*Caroxylon incanescens* (C.A.Mey.) Akhani & Roalson QUETTA000465	Hashok	Wild	Shrub	Whole plant	Fuel
=	*Haloxylon salicornicum* (Moq.) Bunge ex Boiss. QUETTA000328	Lana	Wild	Shrub	Whole plant	Fodder, poisonous
=	*Suaeda fruticosa* Forssk. ex J.F. Gmel. QUETTA000053	Lani/ Lar,ri	Wild	Shrub	Whole plant	Detergent.
Amaryllidaceae	*Allium cepa* L. QUETTA000466	Pimaz	Cultivated	Herb	Root and leaves	Vegetable
Apiaceae	*Elwendia persica* (Boiss.) Pimenov & Kljuykov QUETTA000480	Zera Siyah	Wild	Herb	Seeds	Condiments, medicinal
=	*Coriandrum sativum* L. QUETTA000487	Gishneez	Cultivated	Herb	Leaves, seeds	Condiments, commercial, medicinal
Apocynaceae	*Apteranthes tuberculate* (N.E.Br.) Meve & Liede QUETTA000255	Marmoot	Wild	Herb	Whole plant	Vegetable, medicinal
=	*Calotropis procera* (Aiton) W.T.Aiton QUETTA000230	Aak	Wild	Shrub	Leaves, flower, root, milky extract	Medicinal, poisonous
=	*Rhazya stricta* Decne. QUETTA000019	Aishwarg/ Sewar	Wild	Shrub	Whole plant	Medicinal
Arecaceae	*Nannorrhops ritchieana* (Griff.) Aitch. QUETTA000059	Mazari, Pish	Wild	Shrub	Leaves	Commercial, fruit
=	*Phoenix dactylifera* L. QUETTA000130	Khajoor	Cultivated	Tree	Leaves, Fruits and Seeds	Commercial fruit,
Asphodelaceae	*Aloe vera* (L.) Burm.f. QUETTA000162	Kanwar gandal	Naturalized	Herb	Whole plant	Medicinal, cosmetic, commercial
Asteraceae	*Achillea wilhelmsii* K.Koch QUETTA000003	Zawal/ Boyemadran	Wild	Herb	Whole plant	Medicinal
=	*Helianthus annuus* L. QUETTA000429	Sij Gul	Cultivated	Herb	Flowers, Seeds	Commercial Ornamental, Edible
=	*Pentanema divaricatum* Cass. QUETTA000476	Phosri	Wild	Herb	Whole plant	Medicinal
=	*Sonchus asper* (L.) Hill QUETTA000432	Ghorili gaah	Wild	Herb	Whole plant	Fodder
=	*Xanthium spinosum* L. QUETTA000198		Wild	Herb	Whole plant	Weed
Boraginaceae	*Cordia myxa* L. QUETTA000260	Lesuro	Cultivated	Tree	Fruit	Edible, commercial, medicinal
=	*Heliotropium europaeum* L. QUETTA000016	Gidar wal	Wild	Herb	Whole plant	Medicinal, fodder, weed
=	*H*. *ulophyllum* Rech.f. & Riedl QUETTA000483	Popat	Wild	Under shrub	Whole Plant	Fodder
Brassicaceae	*Brassica juncea* (L.) Czern. QUETTA000467	Jambo	Cultivated	Herb	Whole plant	Vegetable, Condiments
=	*B*. *rapa* L. QUETTA000468	Sirah saag	Cultivated	Herb	Whole plant	Commercial, vegetable, fodder
Cannabinaceae	*Cannabis sativa* L. QUETTA000101	Bhang	Wild	Herb	Leaves	Medicinal, commercial
Capparidaceae	*Capparis decidua* (Forssk.) Edgew. QUETTA000113	Kirar	Wild	Shrub	Whole plant	Commercial, fruit, Medicinal
Caryophyllaceae	*Spergularia diandra* (Guss.) Heldr. QUETTA000464	Dandalo	Wild	Herb	Leaves	Vegetable
Convolvulaceae	*Cuscuta cassytoides* Nees ex Englem. QUETTA000484	Amarbell	Wild	Herb	Whole plant	Parasite
Cucurbitaceae	*Bryonia aspera* Steven ex Ledeb. QUETTA000261		Wild	Herb	Root	Weed, medicinal
=	*Citrullus colocynthis* (L.) Schrad. QUETTA000256	Gunj	Wild	Trailing herb	Fruit	Medicinal
=	*Cucumis prophetarum* L. QUETTA000469	Zaran chibit	Wild	Trailing herb	Fruit, leaves, root	Medicinal, fodder
=	*C*. *melo* L. QUETTA000488	Kharbooza	Cultivated	Herb	Fruit Seeds	Edible
Elaeagnaceae	*Elaeagnus angustifolia* L. QUETTA000199	Sinjid	Cultivated	Tree	Fruit	Edible, ornamental
Euphorbiaceae	*Chrozophora plicata* (Vahl). A.Juss. ex Spreng. QUETTA000470		Wild	Herb	Whole plant	Weed
=	*C*. *tinctoria* (L.) A.Juss. QUETTA000236	Kappo	Wild	Herb	Leaves	Weed, dye
=	*Euphorbia helioscopia* L. QUETTA000056	Zehrichik	Wild	Herb	Whole plant	Poisonous
Fabaceae	*Alhagi maurorum* Medik. QUETTA000140	Kandero/ Shinz	Wild	Undershrub	Whole plant	Medicinal, fodder
=	*Cassia fistula* L. QUETTA000477	Chimkini	Cultivated	Tree	Fruit, Bark Wood, Resin	Ornamental, medicinal, dye, commercial
=	*Melilotus indicus* (L.) All. QUETTA000035		Cultivated	Herb	Whole plant	Fodder
=	*Medicago sativa* L. QUETTA000038	Spishta	Cultivated	Herb	Whole plant	Fodder, Medicinal
=	*Prosopis juliflora* (Sw.) DC. QUETTA000081	Biscot	Naturalized	Tree	Leaves, fruit, wood	Medicinal, fodder, fuel
=	*P*. *cineraria* (L.) Druce QUETTA000013	Kandi	Naturalized	Tree	Bark, wood	Medicinal, fodder
=	*Senegalia modesta* (Wall.) P.J.H.Hurter QUETTA000442	Palosa	Wild	Tree	Wood, Resins Flower	Fuel, commercial, medicinal,
=	*Senna alexandrina* Mill. QUETTA000478	Sana makki	Wild	Shrub	Leaves, pods	Medicinal, ornamental, fodder
=	*Vachellia nilotica* (L.) P.J.H.Hurter & Mabb. QUETTA000239	Babur/ Kikar	Wild	Tree	Bark, wood Resin	Medicinal, dye, commercial.
Lamiaceae	*Ocimum basilicum* L. QUETTA000064	Niazbo	Cultivated	Herb	Whole plant	Medicinal, Tea, insectifuge, ornamental
=	*O*. *americanum* L. QUETTA000463		Cultivated	Herb	Whole plant	Ornamental, medicinal
Lythraceae	*Lawsonia inermis* L. QUETTA000485	Mehndi	Wild	Shrub	Leaves	Cosmetic, medicinal
Malvaceae	*Gossypium sp*. L.	Kapan/ Kapah/kapaas	Cultivated	Shrub	Whole plant	Commercial, fuel, medicinal
Meliaceae	*Azadirachta indica* A. Juss. QUETTA000192	Nim	Naturalized	Tree	Whole plant	Medicinal, anti-lice
Moraceae	*Ficus palmata* Forssk QUETTA000204	Anjer	Wild	Tree	Fruits, wood, latex	Fruit, medicinal, fuel
Moringaceae	*Moringa oleifera* Lam. QUETTA000313	Sohanjna	Cultivated	Tree	Whole plant	Vegetable, medicinal, fuel
Myrtaceae	*Syzygium aromaticum* (L.) Merr.& L.M.Perry QUETTA000479	Lawang	Cultivated	Tree	Fruit, seed	Condiments, medicinal, commercial
Nitrariaceae	*Peganum harmala* L. QUETTA000002	Hermal	Wild	Herb	Whole plant	Medicinal, evil eye
Pedaliaceae	*Sesamum indicum* L. QUETTA000482	Tirr/Till	Cultivated	Herb	Seeds, leaves	Edible, medicinal, commercial
Poaceae	*Cenchrus americanus* (L.) Morrone QUETTA000474	Bajra	Cultivated	Grass	Seeds, stem and leaves	Edible, fodder
=	*Phragmites karka* (Retz.) Trin. ex Steud. QUETTA000413	Nar Baaz	Wild	Reed	Whole plant	Commercial
=	*Sorghum bicolor* (L.) Moench QUETTA000481	Jawari	Cultivated	Grass	Seeds, stem and leaves	Edible, medicinal
=	*Triticum aestivum* L. QUETTA000415	Kholam/ karank, Ann, Gandum	Cultivated	Grass	Seeds, leaves	Edible, commercial
Polygonaceae	*Polygonum patulum* M. Bieb. QUETTA000471		Wild	Herb	Whole plant	Fodder
Rhamnaceae	*Ziziphus mauritiana* Lam. QUETTA000315	Sindhi beer (Bari beer)	Cultivated	Tree	Fruit, leaves,	Edible, medicinal,
=	*Z*. *nummularia* (Burm.f.) Wight & Arn. QUETTA000314	Karkan/ Jangli beer	Wild	Tree	Fruit, leaves, bark	Edible, medicinal
=	*Z*. *spina-chiristi* (L.) Desf. QUETTA000472	Konar	Wild	Tree	Fruit, leaves, bark	Edible, medicinal
Rutaceae	*Haplophyllum tuberculatum* (Forssk.) A.Juss. QUETTA000168	Ganderam	Wild	Herb	Whole plant	Medicinal
Salvadoraceae	*Salvadora persica* L. QUETTA000473	Khabbar	Wild	Tree	Stem, Leaves	Commercial, medicinal, fodder
Solanaceae	*Capsicum annum* L. QUETTA000486	Mirch	Cultivated	Herb	Fruit	Condiment, edible, commercial
=	*Solanum nigrum* L. QUETTA000009	Apri	Wild	Herb	Whole plant	Medicinal, fodder
=	*Withania coagulans* (Stocks) Dunal QUETTA000018	Panir, Awishk,	Wild	Herb	Seeds	Medicinal,
=	*W*. *somnifera* (L.) Dunal QUETTA000283	Lakri/ Aswagandh	Wild	Herb	Whole plant	Medicinal
Tamaricaceae	*Tamarix aphylla* (L.) H. Karst. QUETTA000194	Guz	Wild	Tree	Whole plant	Wind breaker, fodder, dye
=	*T*. *dioica* Roxb. ex Roth QUETTA000195	Lai Gez	Wild	Tree	Whole plant	Wind breaker, fodder, tanning, medicinal
Zygophyllaceae	*Fagonia indica* Burm.f. QUETTA000207	Karkawa	Wild	Herb	Whole plant	Medicinal
=	*Tribulus longipetalus* Viv. QUETTA000209	Gurgandako	Wild	Herb	Whole plant	Medicinal
=	*Tribulus terristris* L. QUETTA000085	Gurgandako	Wild	Herb	Whole plant	Medicinal

### Traditional recipes (TR) accustomed by indigenous women

Traditionally, women are recognized as housekeepers with immense traditional knowledge of medicinal and edible plants in their repositories. The recipes they utilize are the part of their daily life from ancient times [32]. *Calotropis procera* (Aak) is a wild and dominant plant of the area. Used by indigenous women in different ways: The leaves of the aak are tied on knee to cure knee pain. Leaves are boiled for 3–4 times, every time fresh water is added, after straining water is discarded residue is applied on joints to cure rheumatism. Aak’s flowers, leaves, root are dried to make powder and it is taken orally for treating stomach problem and Asthma. Milky white latex of aak is anti-poisonous used against snake or scorpion bite. Milky latex is applied on unnecessary hairs, as a hair removal cream by the rural women. *C*. *procera* is a multipurpose plant it has been widely used in traditional medicinal systems in North Africa, Middle East Asia, South Asia, and South-East Asia [33].

**Fig 2:**
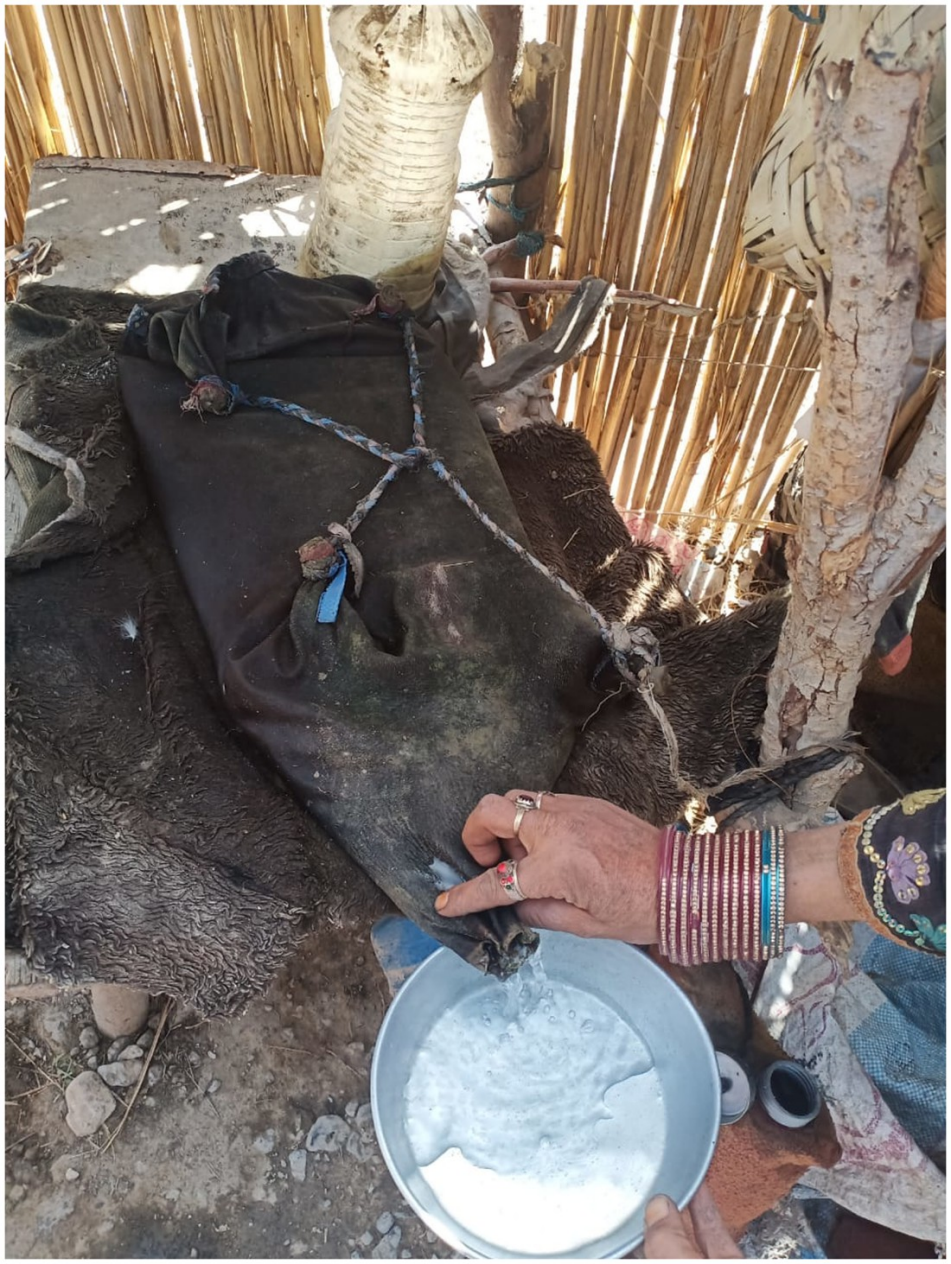
Nomadic women carrying water in Mashkeeza (Leather bag).

*Tamarix aphylla* (Guz) wild plant used as wind breaker, fodder and fuel purposes. *T*. *dioica* (Lai Gez) the leaves are crushed, after straining dried leaves powder, turmeric and mustard oil is added. This is applied on head and ribs for internal injury. The ashes of the leaves are made and it is applied on the wounds for healing. Leaves are placed in the ‘Mashkeeza’ (Fig 2) (small leather bag, used in villages for carrying water or keeping butter-milk) for 15 days in water. Change the leaves and water after each 5 days to make it strong. Powder of the leaves is taken orally with simple water for treating jaundice, hepatic disorders and allergies.

*Azadirachta indica* (Nim/Neem) an important naturalized tree has multiple uses. Due to very harsh climate in summers, the trees help in providing shades. The leaves are soaked for 2 hours, water used for bathing to treat prickly heat. Powder of dried leaves is mixed with mustard oil, applied on hair for healthy growth. Fresh leaves of the neem are boiled and applied in the roots of hair as anti-lice reagent. The leaves of neem are boiled for rinsing blister of the tongue. Seeds of Neem (Nimboli) are taken with water (1 seed on daily basis) for treating diabetes and to cure piles. Sometimes powder (Phaki) of leaves is taken with water for controlling diabetes. Older women prepared a recipe for curing miscarriages in pregnant females with the leaves of neem, rose flowers, raswat, sandal sufaid, celery all the ingredients are boiled and pressed, small tablets are prepared and prescribed once a day. Azadiracthtin an alkaloid is isolated from *A*. *indica* and other molecules such as salanine and melandriol after ingestion causes digestive disorders and loss of appetite (anti-feedant activity). *Azadirachtin* is said to be highest in the kernel than in the leaves and other tissues of the plants [34, 35].

*Salvadora persica* (Khabbar) stem is used as natural tooth brush (chewing stick) considered important for mouth freshening and being paid with spiritual reward frequently used with ablutions before prayers.

Leaves are washed, dried and powdered; a teaspoon is taken with water to cure stomach disorders. Leaves decoction is boiled with butter, cumin seeds, celery and Jaggery. This is considered a very important recipe for the regulation of female menstrual cycle. Leaves of khabbar are used as fodder for Camels.

*Withania coagulans* (Panir, Awishk) is very important wild plant. It has multiple medicinal uses. Seed of the paneer are soaked overnight; extract is taken on empty stomach for blood purification and obesity. Another recipe 1/4 of paneer seed, and ¼ pomegranate peels are mixed and crushed to make powder. Small pills are prepared taken with water orally for piles treatment. Another method of its usage is; Paneer, fennel seeds, mint candy, Choti hareer (*Terminalia chebula*), and cardamom are grind to fine powder. This powder is taken with water, locally known as ‘Ghutti Phakki’ is considered as one the valuable recipes used for abdominal pain and flatulation. Another recipe using paneer is fried *T*. *chebula* (choti hareer) in mustard oil added to the recipe as; Paneer, Neem, channy (whole black gram) and Almond are mixed in equal quantity and crushed to fine powder 1 table spoon is taken daily before breakfast, helps to cure diabetes. *W*. *somnifera* (Lakri/ Aswagandh) root is dried to make phakki (powdered) and taken orally for backache. Seeds are used for kidney and bladder infections [36].

### Cultivated plants recipes

**Fig 3:**
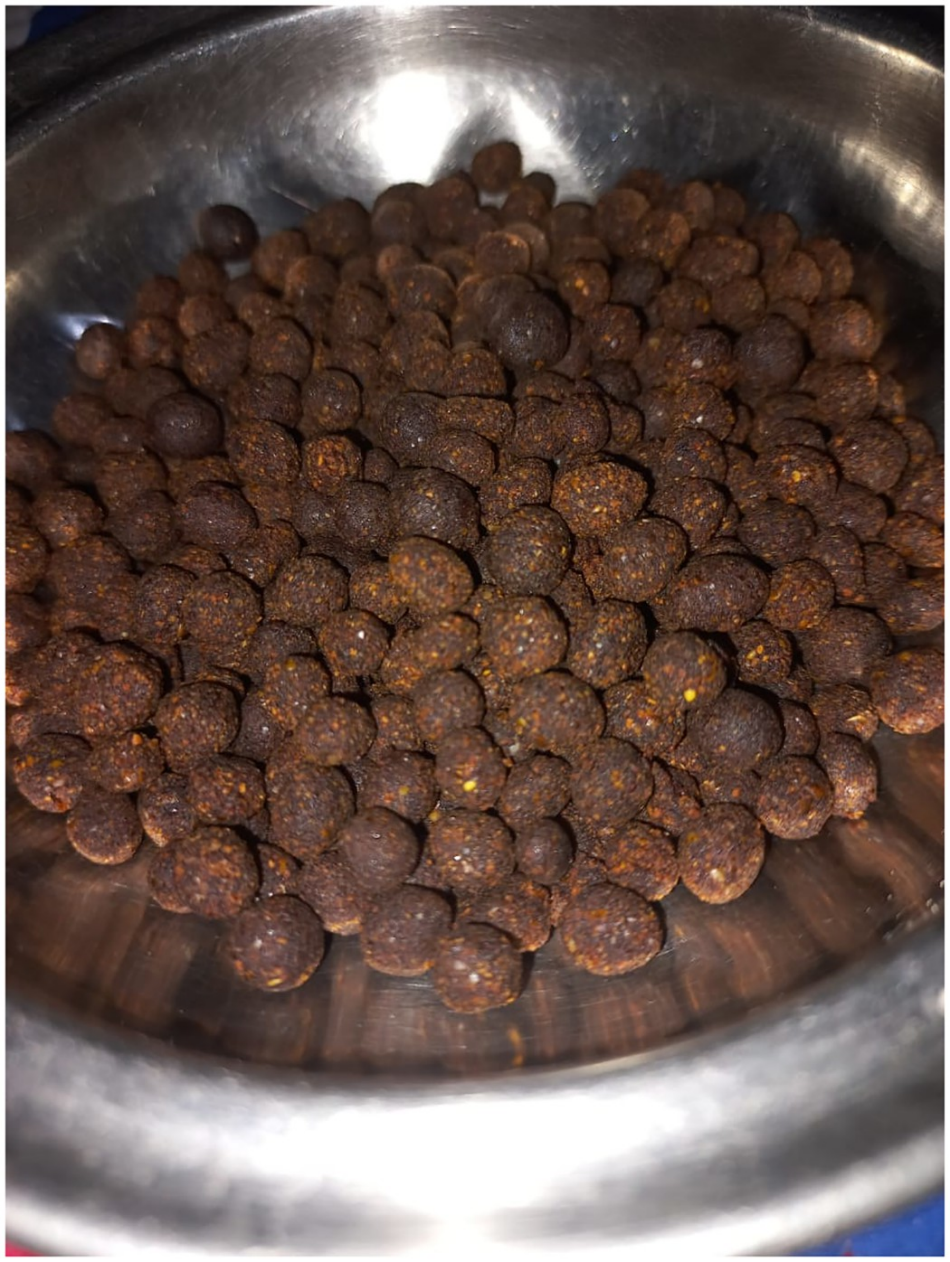
Traditional sweet sphere balls ‘ladoo’ prepared with wheat flour by indigenous women of SD.

Agriculture is the key occupation and means of survival of pastoral people of SD [37]. The terrain is favorable for the cultivation of crops such as *Triticum aestivum*, *Sorghum bicolor*, *Cenchrus americanus* and *Gossypium sp*. Livestock is also a source of finance. Other cultivated crops and vegetables are *Allium cepa*, *Brassica juncea*, *B*. *rapa*, *B*. *oleraceae*, *Coriandrum sativum*, *Capsicum annuum*, *Solanum tuberosum*, SD provide these vegetables in winters to the coldest zones of Balochistan, where most of the area is facing freezing temperature. These cultivated plants not only used as food but also their traditional recipes are famous and mostly prepared by the women for centuries. *Cenchrus americanus* (Bajra) is very important crop flour is used to make bread. Leaves and stems are crushed and used as fodder for livestock. *Sorghum bicolor* (Jowar/ Jawari) it is used as fodder for goats and cows. Its flour is used to make bread and female also use it as a cosmetic. A traditional drink known as “Sattu” is prepared from roasted grains or mixture of many grains together and sweetened with jaggery is used in hot summers that help in protection from heat strokes. *Triticum aestivum* (Wheat/Kholam/Karank/Ann/Gandum) is a very important crop cultivated in the area. Its flour is used for making bread. Wheat flour is fried in mustard oil on low flames till its change of color to light brown. Sweets ‘chashni’ is. That fried flour is kneaded with sweet chashni (prepared with sugar added to boiled water) to make ‘ladoo’ (traditional sweet sphere balls) (Fig 3). People make their traditional spicy dish using wheat with lentils known as “Haleem”. An old tradition of the region still existing in the villages is ‘waxing’ of The new born with flour of wheat that is kneaded with water, dip the flour in mustard oil to massage babies.

**Fig 4:**
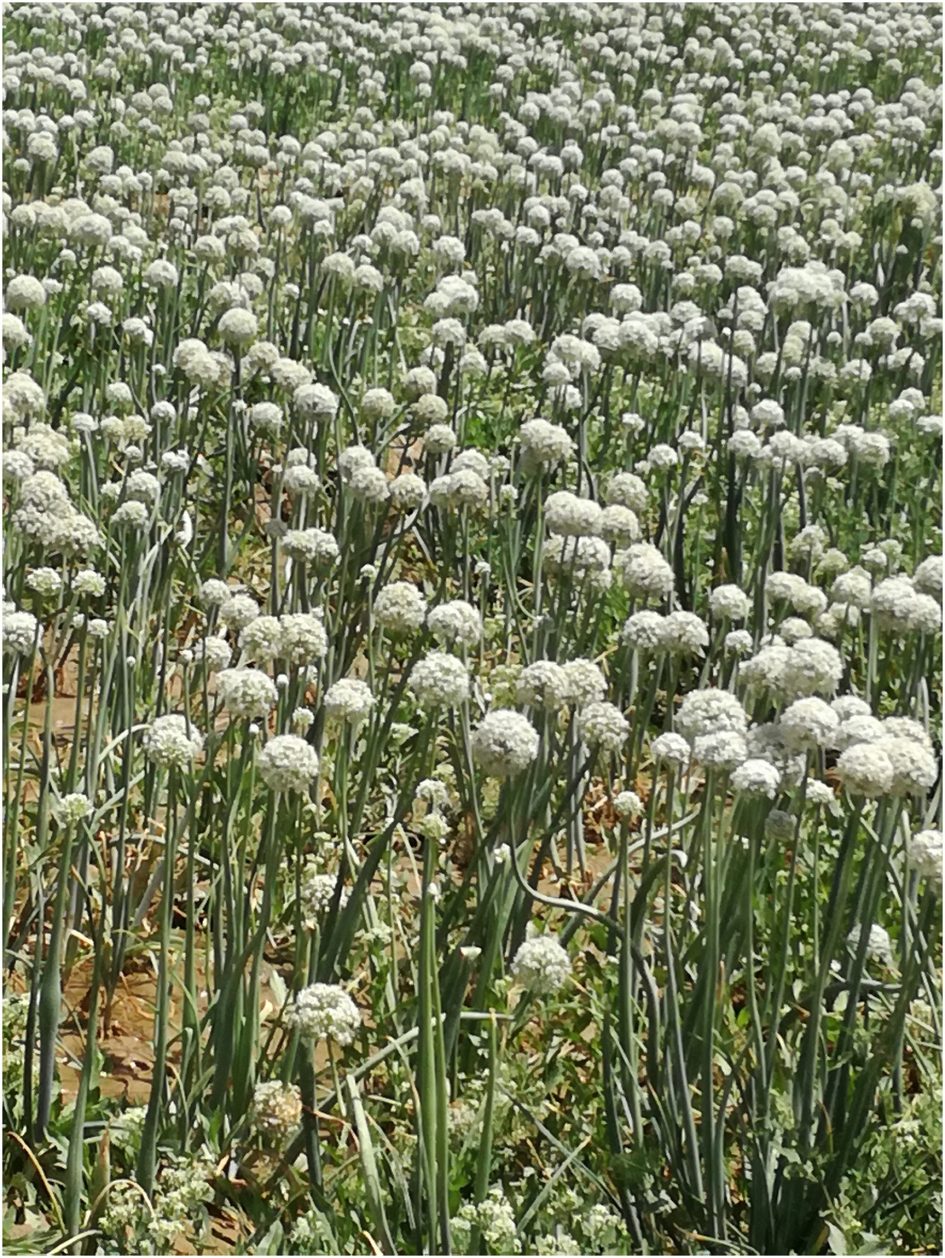
*Allium cepa* (Pimaz) cultivated vegetables in the terrains of SD.

*Gossypium sp*. (Kapah/kapaas) is important commercial crop for fiber and textile industry. Seed are crushed extracts of these seeds are applied for bone fractures. Dry parts are used as fuel. *B*. *oleraceae* cultivated in SD plant is used as raw in salad as well as cooked. *Allium cepa* (Pimaz) is cultivated in wide range of the SD (Fig 4). Plant is cooked for making curry. Leaves and root are used as salad. In summers onion with vinegar is used to be protected from heat stroke and diarrhea. Some of the food plants reported by SD indigenous people in current study are earlier reported from other regions as their folk foods [38, 39].

**Fig 5:**
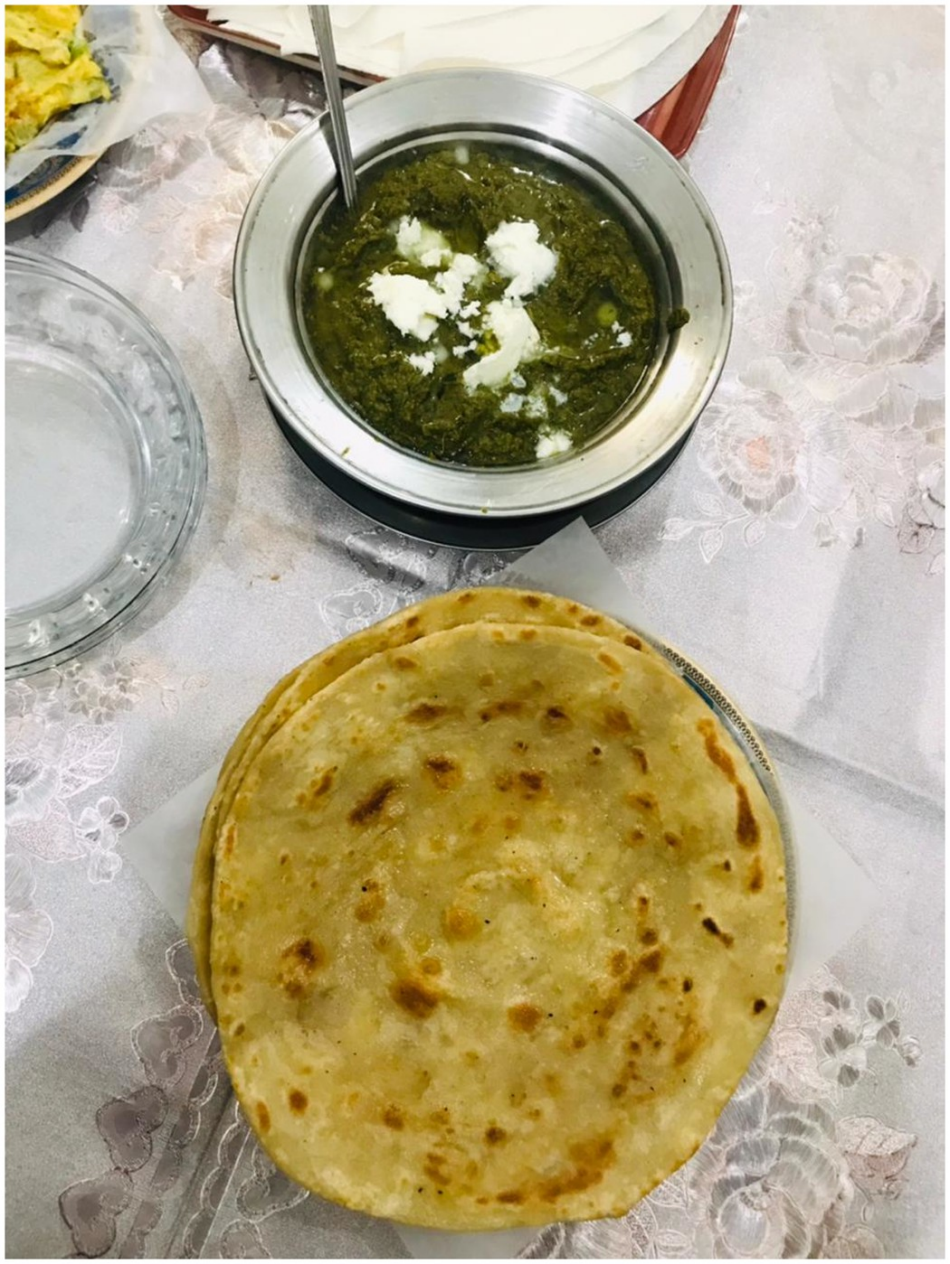
*B*. *rapa* cultural dish served with butter and wheat bread (Photo: A. Ahmed).

*Brassica juncea* (Jambo) cultivated plant used raw as a salad. *B*. *rapa* (Sirah saag) is very important cultivated plant. Used as multipurpose commonly known as mustard plant. Its oil is extracted from seed is edible used for cooking purposes as well as its medicinal properties increase its importance in different traditional recipes. Oil is used for hair massage. The remaining parts of seeds used as fodder for animals. The plant is boiled, some salt is added and drink is prepared for treating flue. Mustard plant is boiled; the decoction is prepared and given to the mother after the baby birth for fast recovery. *B*. *rapa* is also taken as vegetable and considered as the most delicious cultural dish served with butter and bread (Fig 5).

**Fig 6:**
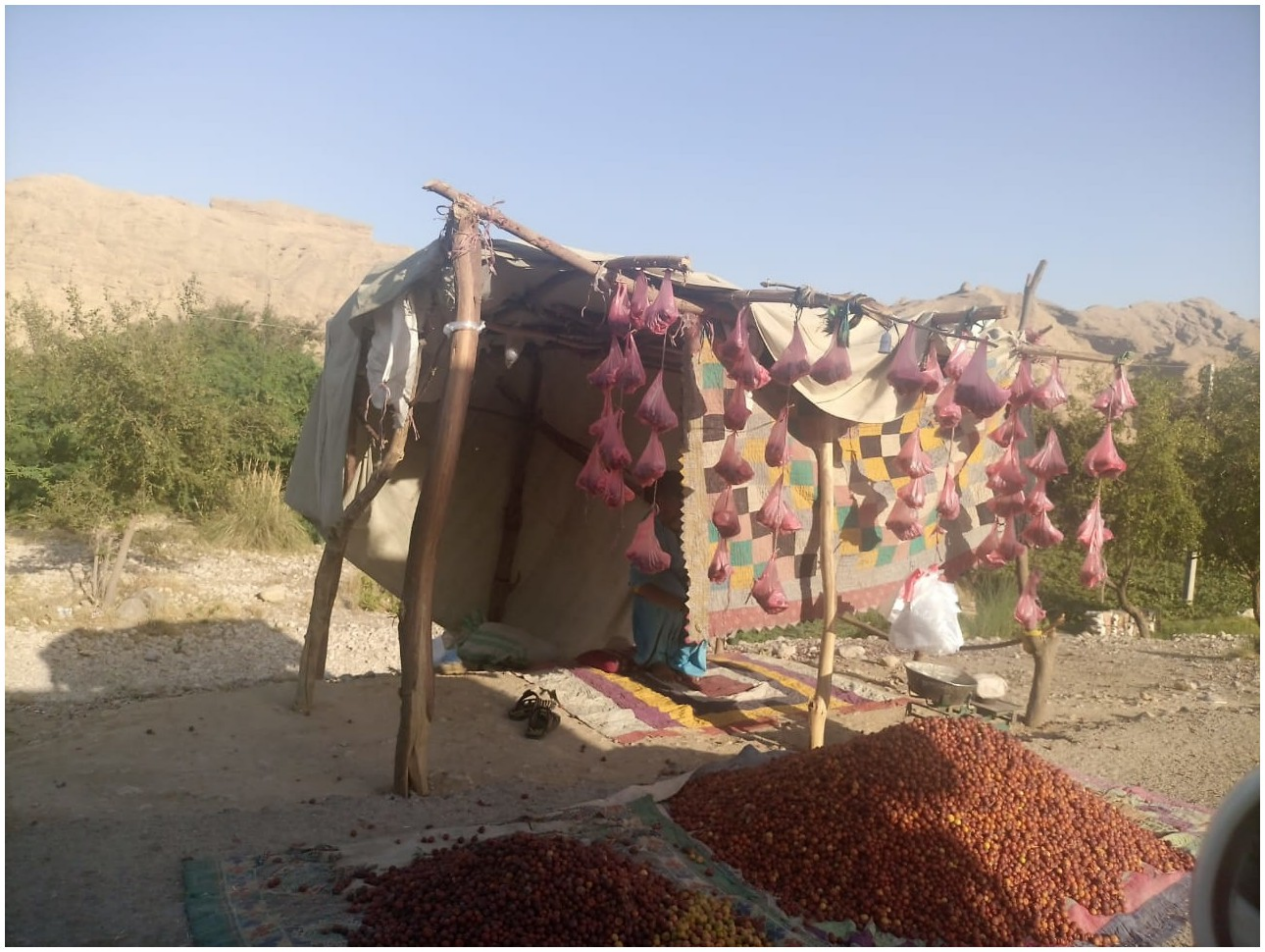
*Ziziphus spp*. fruits stall for sale at road side.

SD is also famous for *Ziziphus mauritiana* (Sindhi beer/Bari beer) edible fruit tree. People of the area depend upon these fruits for generating income (Fig 6). This fruit can play a key role during the food scarcity the fresh fruit as well as dried form of fruit is used. Honey of *Ziziphus* beri is preferabl, and collected abundantly by the local inhabitants and sold in the local market. No proper means are available for its export on a, large scale. Fruit and leaves are also used in different remedies for the treatment of allergy. Leaves of Sindhi beer are boiled in water and it is massaged on hairs. Leaves of beer are crushed and mixed with red lentils for allergy.

*Elaeagnus angustifolia* (Sinjid) fruiting trees are ornamental and cultivated for its sweet fragrance. Dried fruit is edible. Trees are grown on road sides for its shade and sweet scented flowers. Foods are edible and nutritious and helped in times of food scarcity.

*Phoenix dactylifera* (Khajoor) is a cultivated fruiting tree. It is one of the most important edible fruit culturally, traditionally and ritually. Also have medicinal importance. Leaves are used in making huts. Seeds are also used for making Tasbeeh.

*Helianthus annuus* (Sij Gul) is a cultivated plant. Oil is extracted and is used for cooking purposes. Seeds of sun flower are edible and taken as a rich source of nutrients. The plant can also grow ornamentally.

*Moringa oleifera* (Sohanjna) cultivated plant leaves are used. Fruit is taken raw or cooked as vegetable. Wood is also used as fuel.

*Cordia myxa* (Lesuro) is one of the economically important cultivated plants of SD. It is used to make pickle and also taken raw as salads.

**Fig 7:**
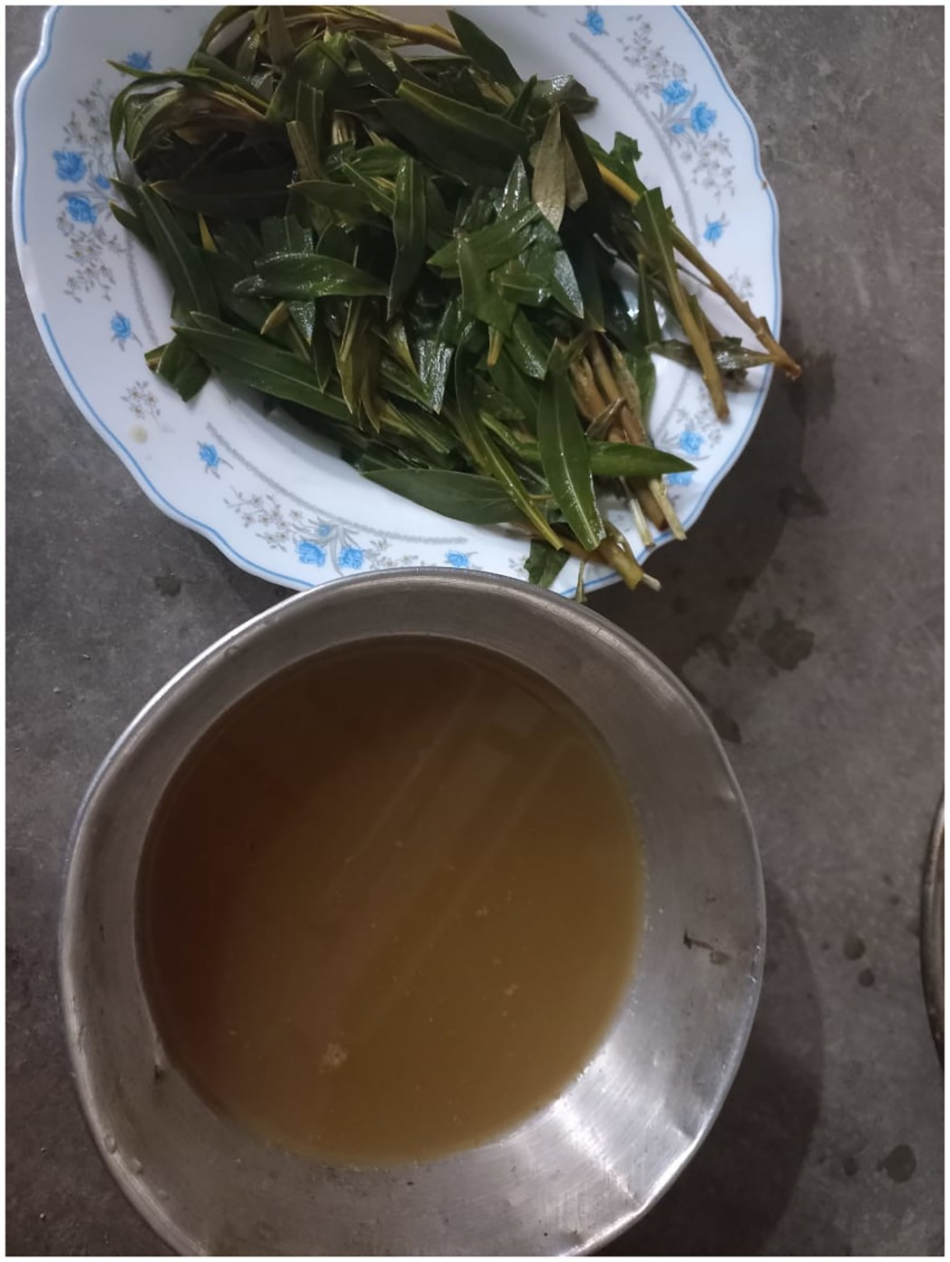
Traditional recipes preparation by women decoction of *Rhazya stricta*.

*Rhazya stricta* (Aishwarg/ Sewar) plant is soaked for 24 hours and applied this water for rashes applied on pimples (Fig 7) [36].

### Sustainable use of different marketable products

**Fig 8:**
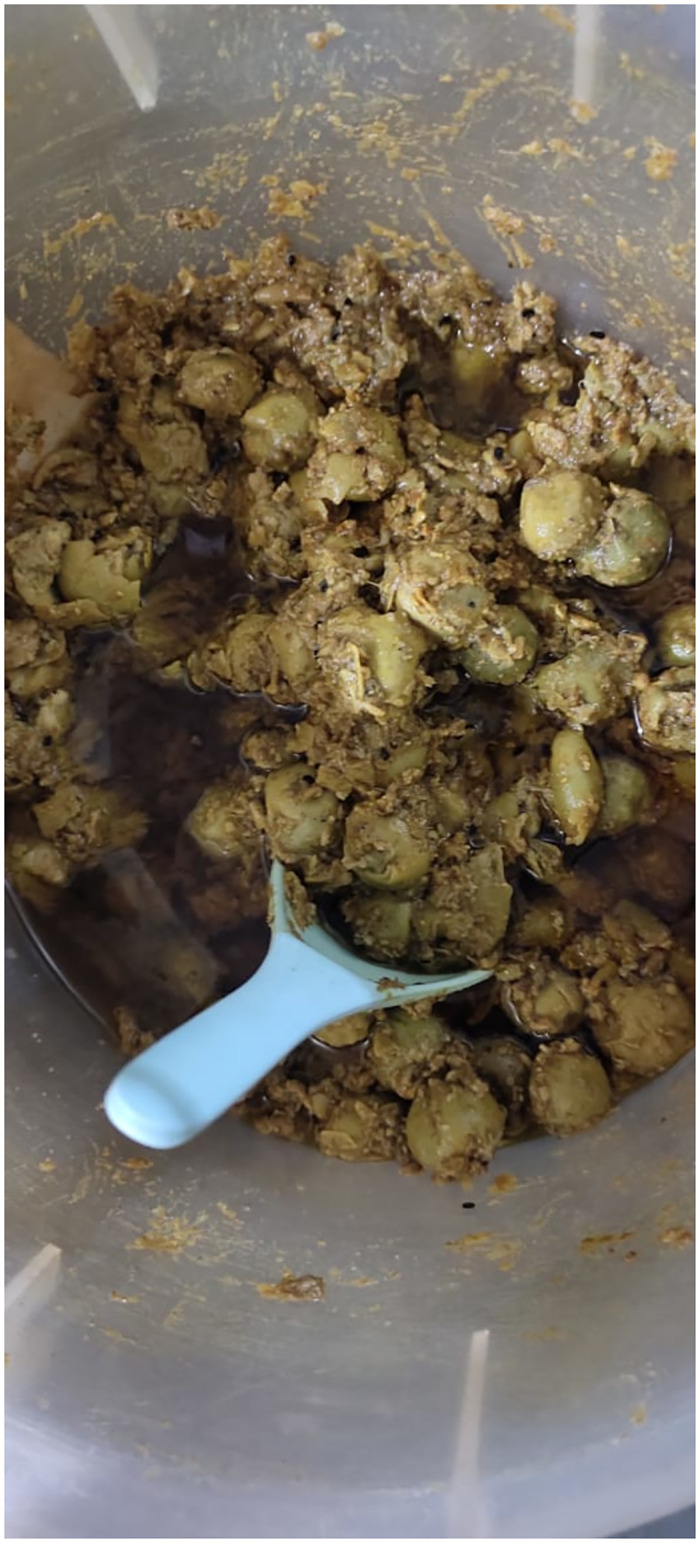
Homemade pickle of *Cordia myxa* (Lesuro).

**Fig 9:**
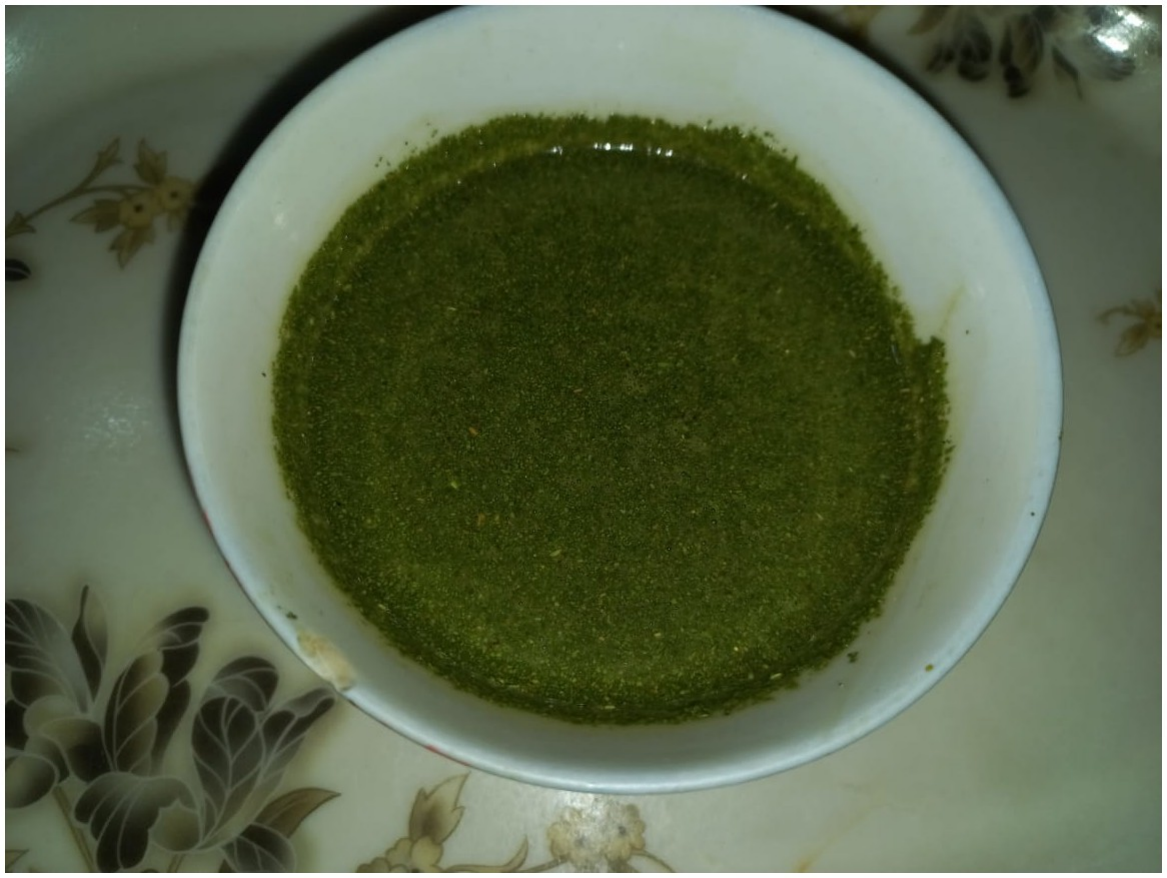
The green Sauce (chutni) of *Coriandrum sativum* (Dhania) and *Capsicum annuum* (Green chili) by the women villager.

Results of the current study indicates that the knowledge of indigenous community about the plants and their useful utilization contributes in communities developments [6]. The sustainable uses of their knowledge about the plants, cultural recipes and their byproducts can play a vital role improving the economy of the communities of the underdeveloped area such as SD. The women are familiar with more species and their uses than men and use the remedies at home to treat different diseases [6, 40]. ethnic community of SD makes pickles with fruits of *Capparis decidua* and *Cordia myxa* (Fig 8), the products are sold in local market, as well as in other parts of the country. These products are also presented to the relatives and friends as a delicacy of the area. “Green chutni” (Sauce) is prepared in vinegar with *Coriandrum sativum* (Dhania) and *Capsicum annuum* green chillies (Fig 9) is also the product made by women at home and sold in the local market. Fruit of *Capparis decidua* (Kirar) is edible taken raw as a salad. Indigenous women prepared pickles and jams from the mature fruit and. Its coal is made by burning kir’rar and it is applied on Boils. The wood decoction is added to black tea that helps in relieving body aches. Neddle like branches of kir’rar are used by women for ear piercing. Smoke of burning wood of kir’ars is also taken for allergy.

**Fig 10:**
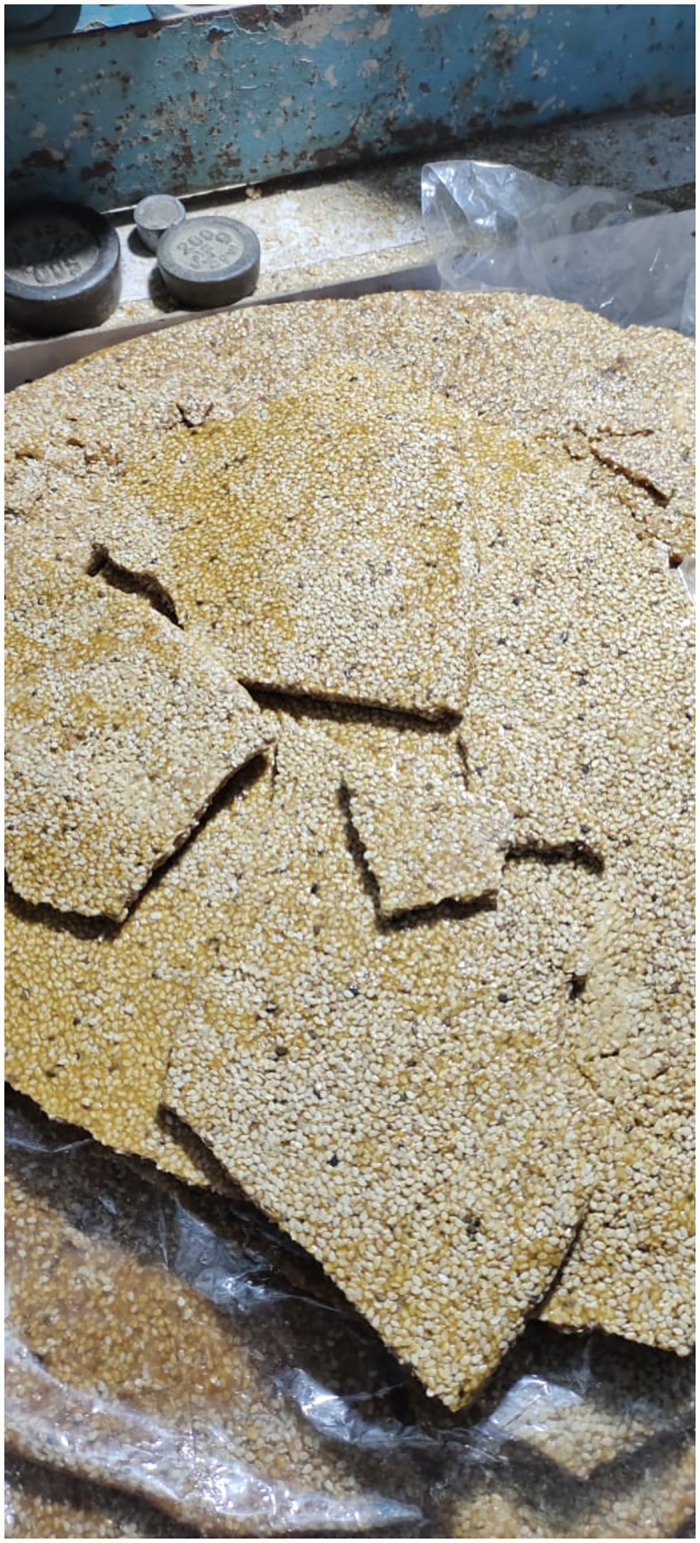
Traditional homemade dessert (Laai) on a cart in local market.

Laai is prepared by *Sesamum indicum* (Tirr/Till) (Fig 10) is the traditional dessert and a delicacy of the area. The product is marketable and sold in the local market as well as in different parts of the country. It also has a rich nutritional value and preferably used to treat weakness of the bladder, especially in women and kids its oil is also used to treat paralyzed people by applying the oil on body. ‘Till’ is taken either as seeds or cooked by indigenous women to regulate the menstrual cycle.

**Fig 11:**
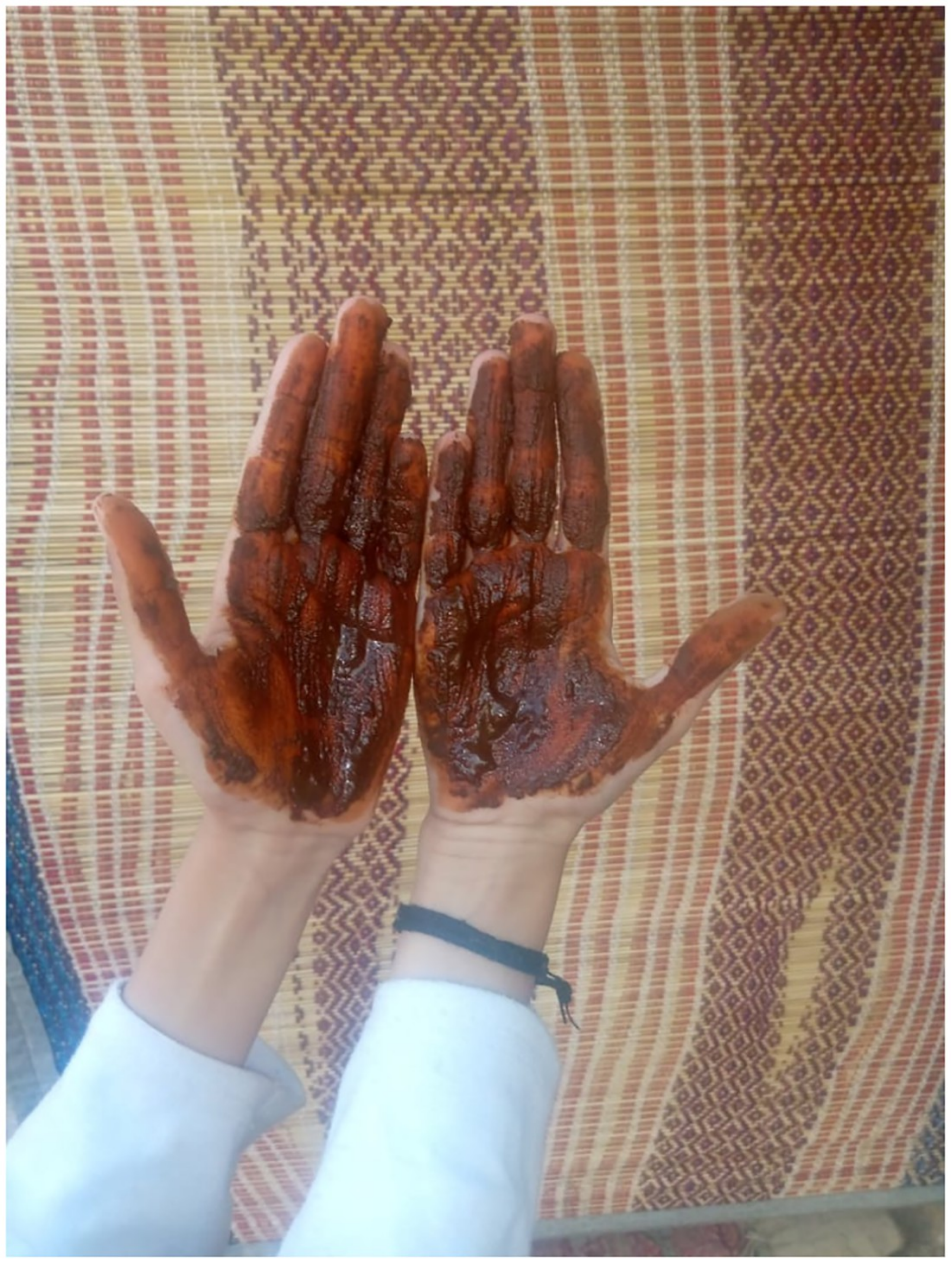
A young village girl applied Henna (*Lawsonia inermis*) on her hands in summers as a cooling agent.

*Lawsonia inermis* (Mahndi/Henna) has cooling properties as the Sibi is very hot climate in summers people apply paste on hand and foot for cooling purpose (Fig 11). Henna has many other health benefits like it can be used as an anti-bacterial paste or anti-fungal paste. It can also be used to enhance the growth of hair. Henna also has a pleasant smell. Henna bark is also used. Good quality found in the area and sale in different parts of country. Henna is very useful in treating “Migraine” and headaches. Women of the area used it frequently by applying on hand and feet not only as a ceremonial tradition but to lower body temperature in hot summers. Old women also used the leaves to dye their hair. *L*. *inermis* is having medicinal properties, contain carbohydrates, proteins, flavonoids, alkaloids, terpenoids, quinones aspirin [41].

**Fig 12:**
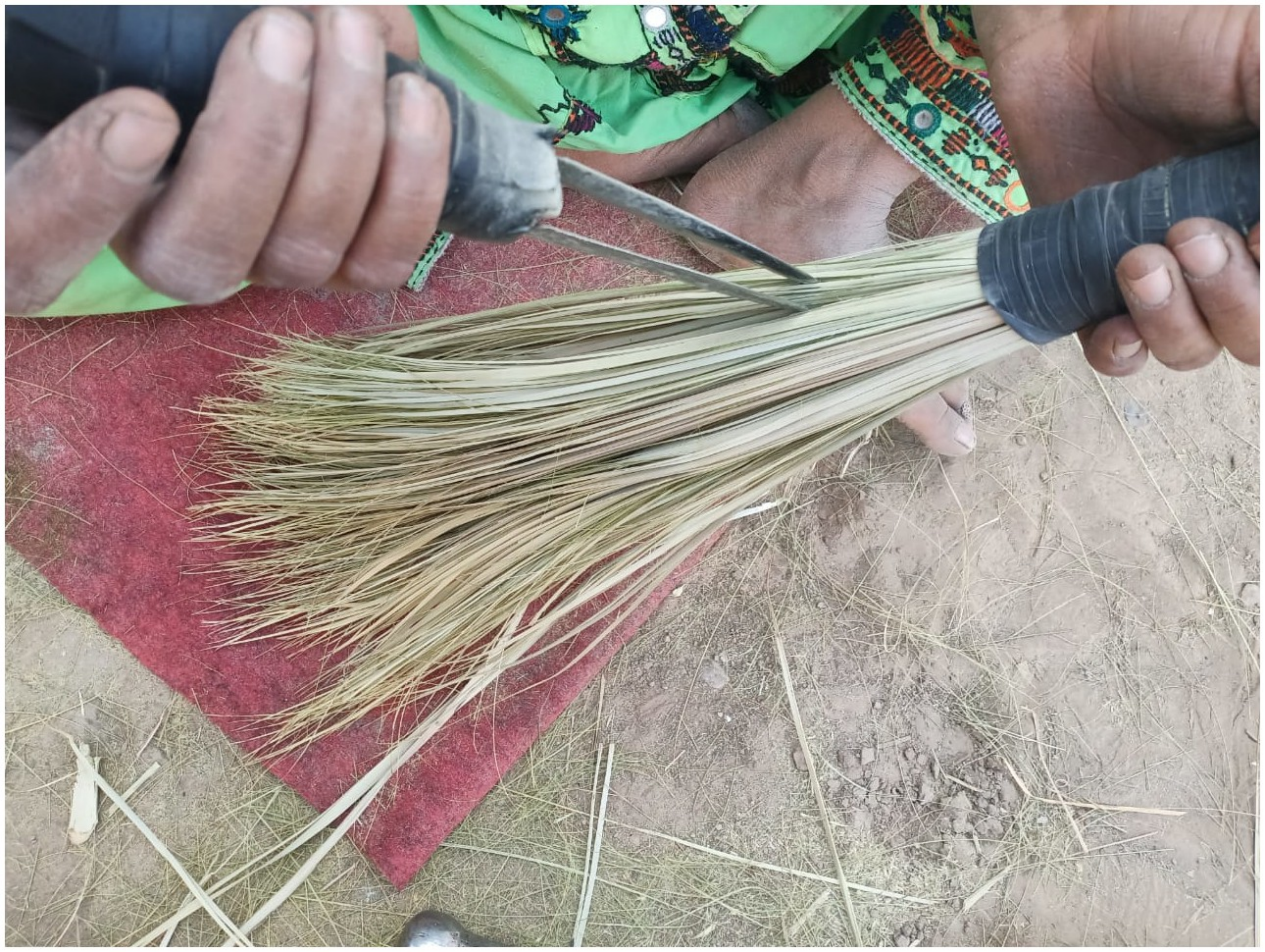
Weaving the basket and brooms for commercial purpose *Nannorhops ritchieana*.

Traditional Parch, carpet, prayer mats, Baskets, broom, hand fans and bed rope are made with leaf and tasbeeh with seeds of *Nannorrhops ritchieana* (Mazari/Pish) (Fig 12). These products are also the source of income for the poor people of the area. Local peopl prepare these products at their homes and sale in the market.

**Fig 13:**
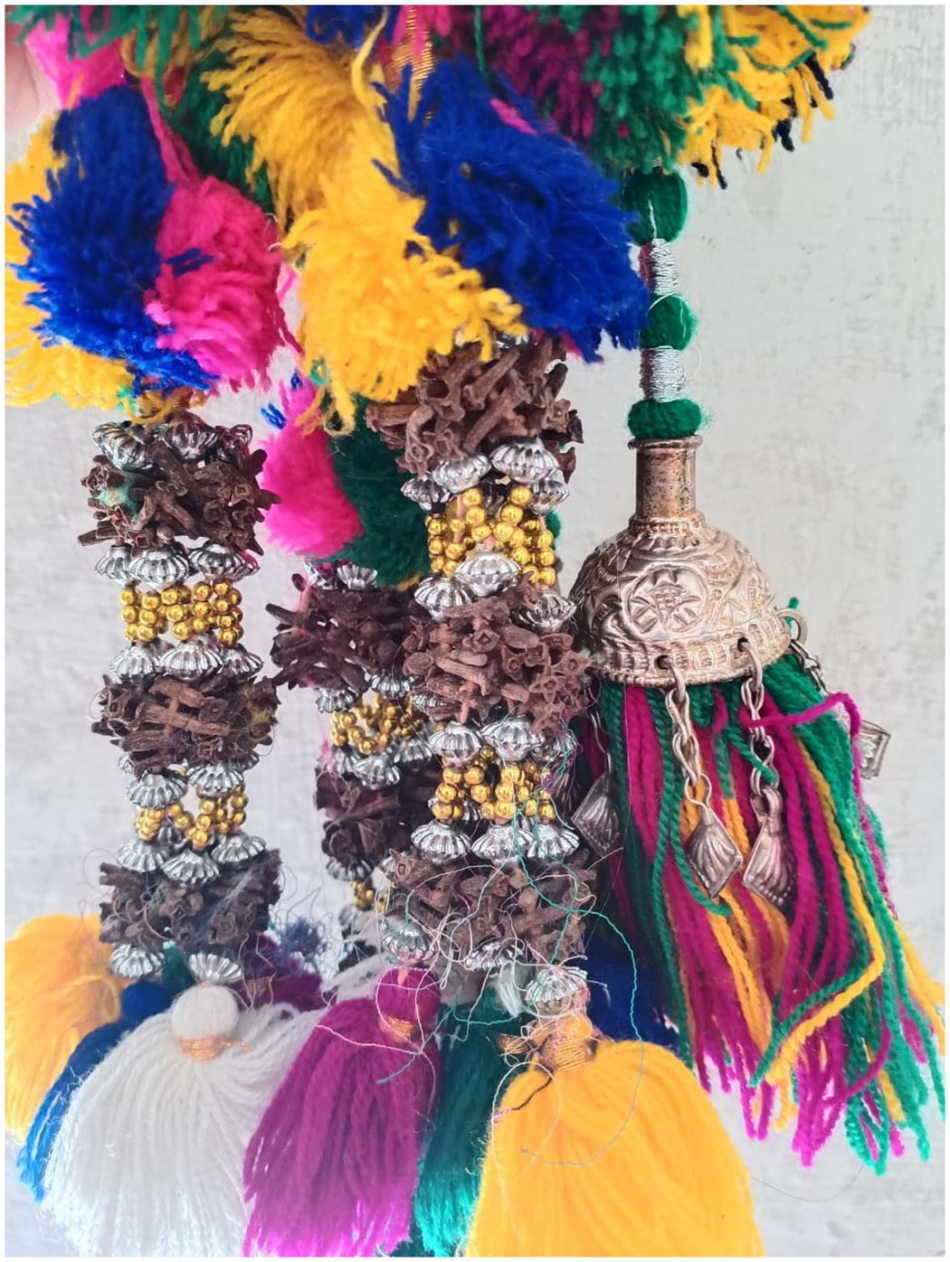
Hair tassel ‘saagi’ prepared with colorful threads, beads and Lavang (*Syzygium aromaticum)*.

Women of the area at home made the traditional braid tassel (saagi) with *Syzygium aromaticumat* (Long/Lavang) with the help of colorful threads (Fig 13). Women decorate hair and sale in the market. The braid tassel is a part of their ethnic clothing and used as a folk accessory of their culture. Lavang is also used as a condiment and it is chewed to cure toothache.

*Aerva javanica* (Bal Buh) utilized by the indigenous people. The thick, white inflorescences have traditionally been harvested for stuffing cushions and pillows. These pillows are used in their houses as well as sold in the market. Leaves are used as fodder whole plant is used as a fuel purposes. *A*. *javanica* var. *bovei* (Surkhri), the flower extract is used by the females in cosmetics as a lip-gloss. This plant is also used as a fuel [42].

### Multiple uses of divers flora of SD

*Phragmites karka* (Nar Baaz) is a reed common in warm swamps, used to make furniture and also used to make curtains. *Chrozophora tinctoria* (Kappo) wild plant used for making dye used commercially for dying threads. *C plicata* used to heal wounds. *Heliotropium europaeum* (Gidar wal) is a wild plant of SD. Plant is mashed and its extract is applied on skin for allergy by women of the area. Plant is also used as a fodder. *H*. *ulophyllum* (Popat) is endemic to Balochistan also used to treat skin allergy. *Cannabis sativa* (Bhang) has many traditional medicinal and commercial properties. The plant is used as tranquilizer. Leaves are crushed with milk in summers the drink is used as cooling agent. *Bryonia aspera* is a weed roots of plants are used traditionally to cure gastrointestinal problems. *Citrullus colocynthis* (Gunj) dried fruit pulp is used for gastrointestinal disorders and used as antidiabetic. *Euphorbia helioscopia* (Zehrichik/ zehrili boti) extract is used as anti-poison for scorpion bites. *Alhagi maurorum* (Kandero/ Shinz) wild thorny plant, root decoction is used against abscess and swellings. Whole plant is boiled and after cooling it is drunk especially the women take it after delivery for weight loss. Plant is good fodder for camels [42]. *Cassia fistula* (Chimkini) tree is cultivated ornamentally, Fruit and seed are purgative used traditionally for gastrointestinal problems, Resin is used to cure joint pains, Bark is used for tanning, Wood is used in making building materials tools etc. *Medicago sativa* (Spishta), an important herb is cultivated as fodder for livestock it is also used medicinally. *Prosopis juliflora* (Biscot), plant leaves extracts is applied on hairs for healthy growth. Fruit (Chichka) of Biscot is used as fodder for goats. Dry wood is used as fuel. *P*. *cineraria* (Kandi), peel is soaked for 24 hours and it is taken orally. It is considered as a good source of fodder for goats and camels because it improves milk production. *Senegalia modesta* (Palosa) wood is used as fuel and building materials, Resin is used in many recipes to cure arthritis. Honey of the tree is of good quality. *Senna alexandrina* (Sana makki), dried leaves are used medicinally against intestinal worms, gastrointestinal problems and rheumatism. Pods are a source of a fodder. *Vachellia nilotica* (Babur/ Kikar), is one of the valuable trees for honey bee keepers. Honey collected from the tree is considered of high quality collected by the local inhabitants and used as food and medicine. Resin is used to cure joint pains, Bark is used for tanning, wood is used in making building materials and tools etc. *Ocimum basilicum* (Niazbo), plant leaves are used as herbal tea to cure chest infections. Seeds are used in drinks in summers to lower body temperature. Plants are cultivated ornamentally in houses and it is considered as flies and mosquito repellent. *O*. *americanum* (Niazbo) is an ornamental plant. *Peganum harmala* (Hermal) Seed of harmal are soaked it is taken orally. Plant is crushed in form of powder and it is used as Phakki to treat obesity. Seeds are also utilized daily for one month to cure sciatic pain by the women of SD. *Haplophyllum tuberculatum* (Ganderam), is medicinally used to treat fevers. *Fagonia indica* (Karkawa) wild whole plant without root are dried to make phakki and taken orally for diabetes, cancer, fatness, stomach pain and for allergy [14]. *Tribulus longipetalus* (Bakhrda) is used by female of the area for treating female hormonal issues, backache and stomach infection. *T*. *terristris* (Gurgandako) is used for treating kidney problems. *Elwendia persica* (Zera Siyah), seeds are used as condiments. It also used medicinally importance. *Suaeda fruticose* (Lani/ Lar,ri), The plant is burned to make ashes which are called “Khaar” locally. This khaar is used for washing clothes. It is antimicrobial and gives fragrance to clothes. *Aloe vera* (Kanwar gandal) is the wild succulent plant having long history of being used for medicinal purposes, dating back to ancient Egypt. The women of SD also use its gel in many TFR. The extract of kanwar gandal is used against obesity, rheumatism. The pulp is used in making different cosmetics. The plant is also grown ornamentally. *Achillea wilhelmsii* (Zawal/ Boyemadran), whole plant is soaked in water, it is used to cure hypertension, Stomach disorders and obesity [43]. *Cuscuta cassytoides* (Amarbell) is a parasitic plant on important wild and cultivated plants of SD. It is used as fodder. *Sonchus asper* (Ghorili gaah) is also used as fodder. *Haloxylon salicornicum* (Lana) is poisonous plant can be used as fodder for camels in small amount. *Melilotus indicus* is wild weed used as fodder.

### Wild Food Plants (WFP)

It is recognized that indigenous foods of local flora within an area can be powerful sources of nutrients and benefits human health [39, 44]. Despite this fact, the use of indigenous foods has declined due to the negligence of the potential of these foods in modern commercialized and industrialized markets and lack of investment in research and development. Some poor people use these plants for fulfill their daily food requirement [29]. *Ficus palmate* (Anjer) tree is wildly grown in the area. Fruit is edible used fresh and dried. Fruit is laxative: Latex is used in skin disorders by applying directly or added to homemade white butter used to treat “vitiligo”. Wood is used as fuel. *Ziziphus*. *nummularia* (Karkan/Jungli beer) and *Z*. *spina-chiristi* (Konar) are wild edible fruits utilized by local people. Fruit of Jungli beer is crushed to make powder (phakki) and it is taken with water orally for cholera, abdominal aches. *Amaranthus viridis* (Sarmi) is a weed whole plant is cooked as vegetable and used by local people. *Chenopodium album* (Kalpir) is the Weed of winter season (Rabi) crops. Leaves extract and seeds are used as antimicrobial and antihelmintic. Leaves are also used as vegetable by local people plant can be utilized in food scarcity. *Apteranthes tuberculate* (Marmoot) wild plant is eaten raw or cooked as vegetables. The plant also has medicinal importance used to cure diabetes and rheumatism. The plant is also listed in endangered species. *Solanum nigrum* (Apri) wild FP given to pregnant women as it is considered healthy food. Seed extract is used as an anti-inflammatory reagent and to treat obesity. *Spergularia diandra* (Dandalo) wild plant leaves are edible and taken raw. Humans have gathered WEPs since ancient times, and they have become part of the human diet and traditional food systems. WEPs still play an important role when food crops are scarce, ensuring food sovereignty and food security, and they potentially contribute to well-being in vulnerable households. WEPs can also be central to efforts to empower local market actors and reduce the distance between consumers and producers, thereby diminishing the overreliance on globalized value chains [45].

## Conclusions

The current study conducted in eight villages of Sibi District is the first report on vegetation used traditionally in the area. The present survey illustrate the household food recipes based on traditional dishes that also have social, cultural, and economic impact on the sustainability of local communities of SD, Balochistan Pakistan. SD is one of the hot spots of Pakistan having rich bio-diversity as well as ironic cultural diversity. Rich agricultural land along with wild flora has profound potential for food security and sustainable use of nutrition and marketing potential for income generation of the living communities of the area. Due to low economic status, the local inhabitants still collect these plants for their own food as well as for generating revenue through selling different products in the market. Steps need to be taken for cultivation and utilization of important wild food plants, their cultivation and its effective marketing. The study recommends developing the marketing, ecological and social strategies that facilitate access to benefits from natural resources and conservation of women indigenous knowledge of bio-cultural heritage of the region before its erosion.

## Supporting information

S1 File Questioners filled by informants during study. (DOCX)

S2 File (PDF)
