# Supplementary figures and images for: The sustainable use of diverse plants accustomed by different ethnic groups in Sibi District, Balochistan, Pakistan
Source: PLoS One. 2024 Feb 21;19(2):e0294989. doi: 10.1371/journal.pone.0294989 (PMC10880983; doi:10.1371/journal.pone.0294989)

**Questioners filled by informants during study**


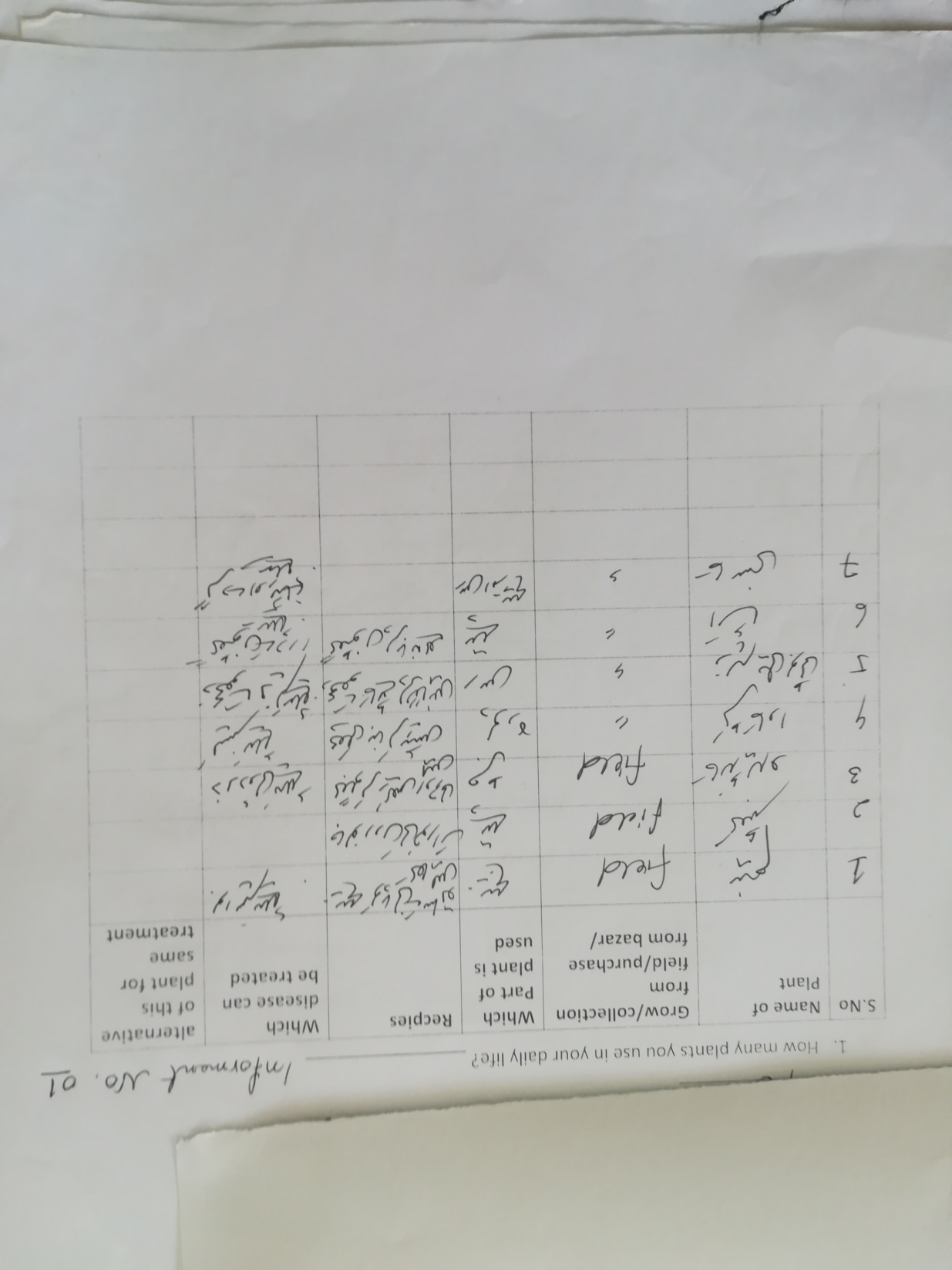


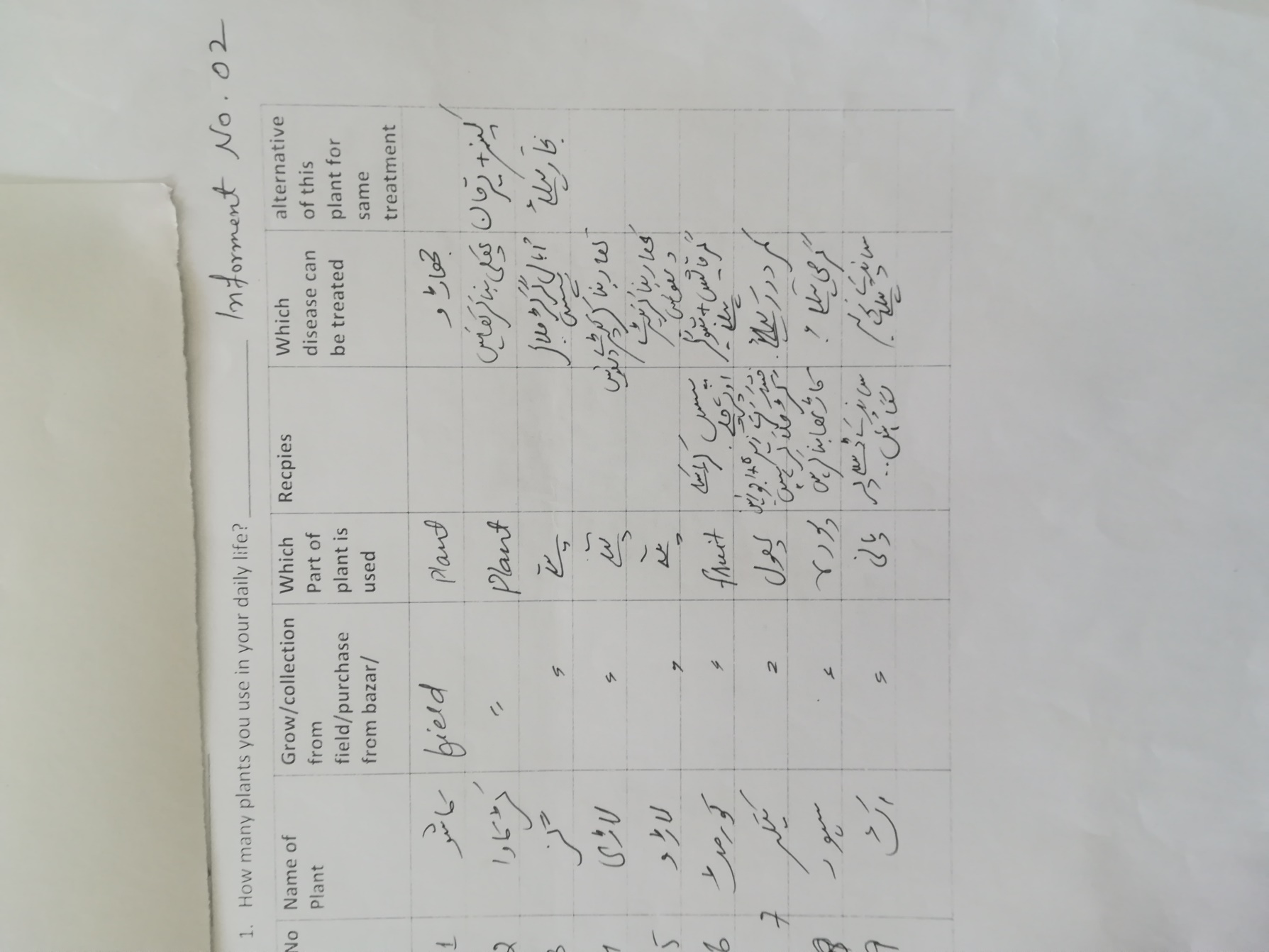


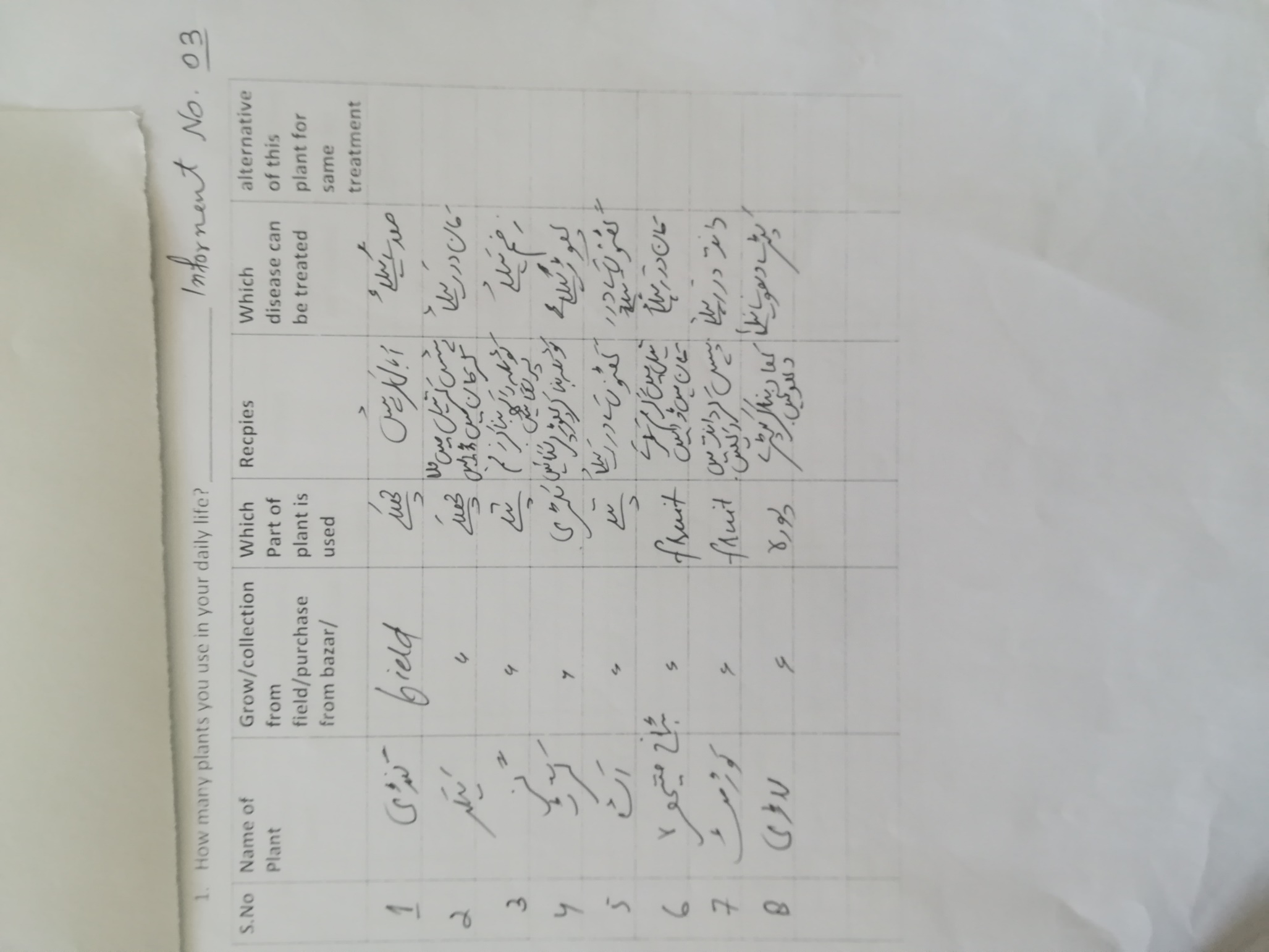


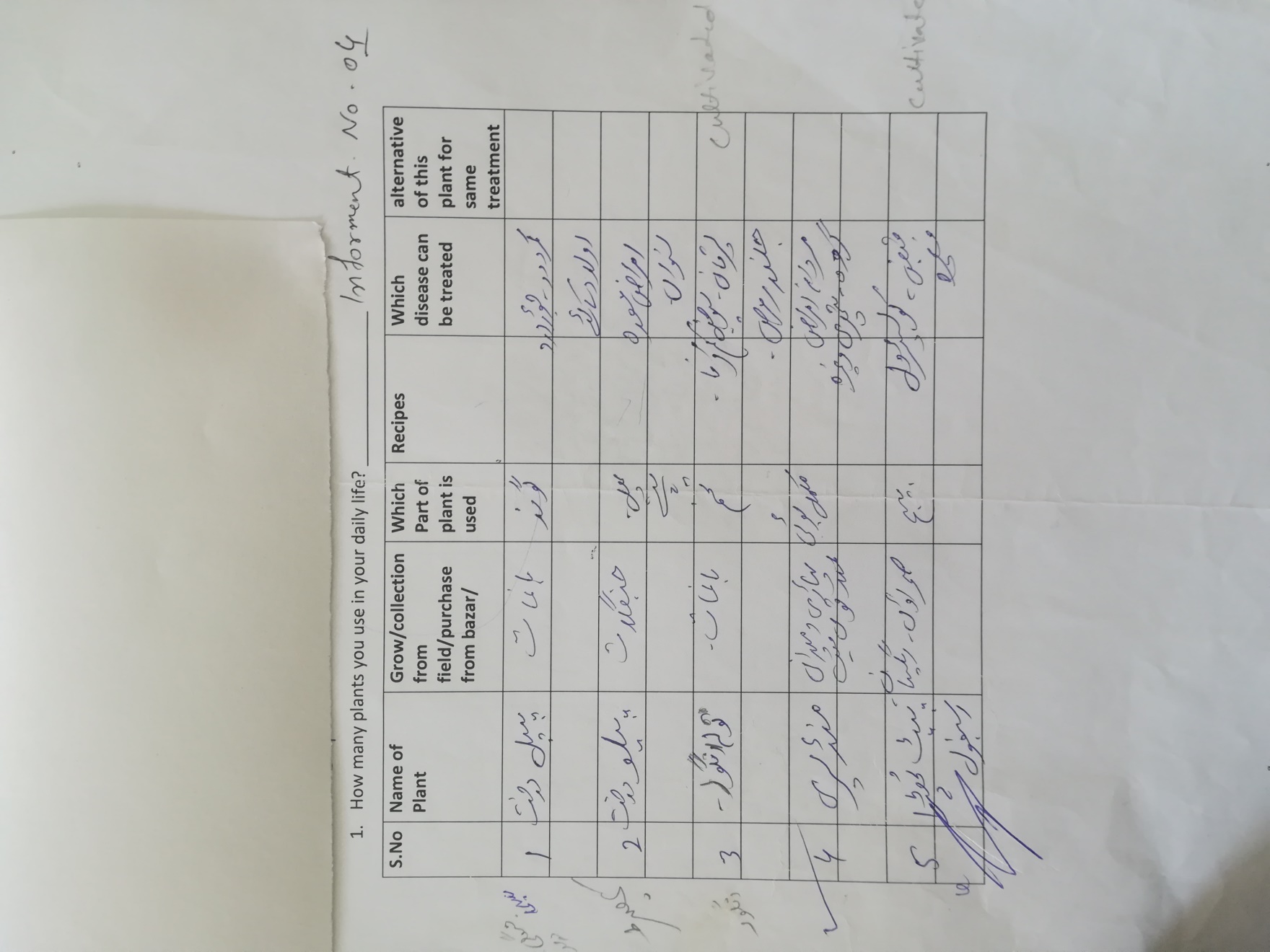


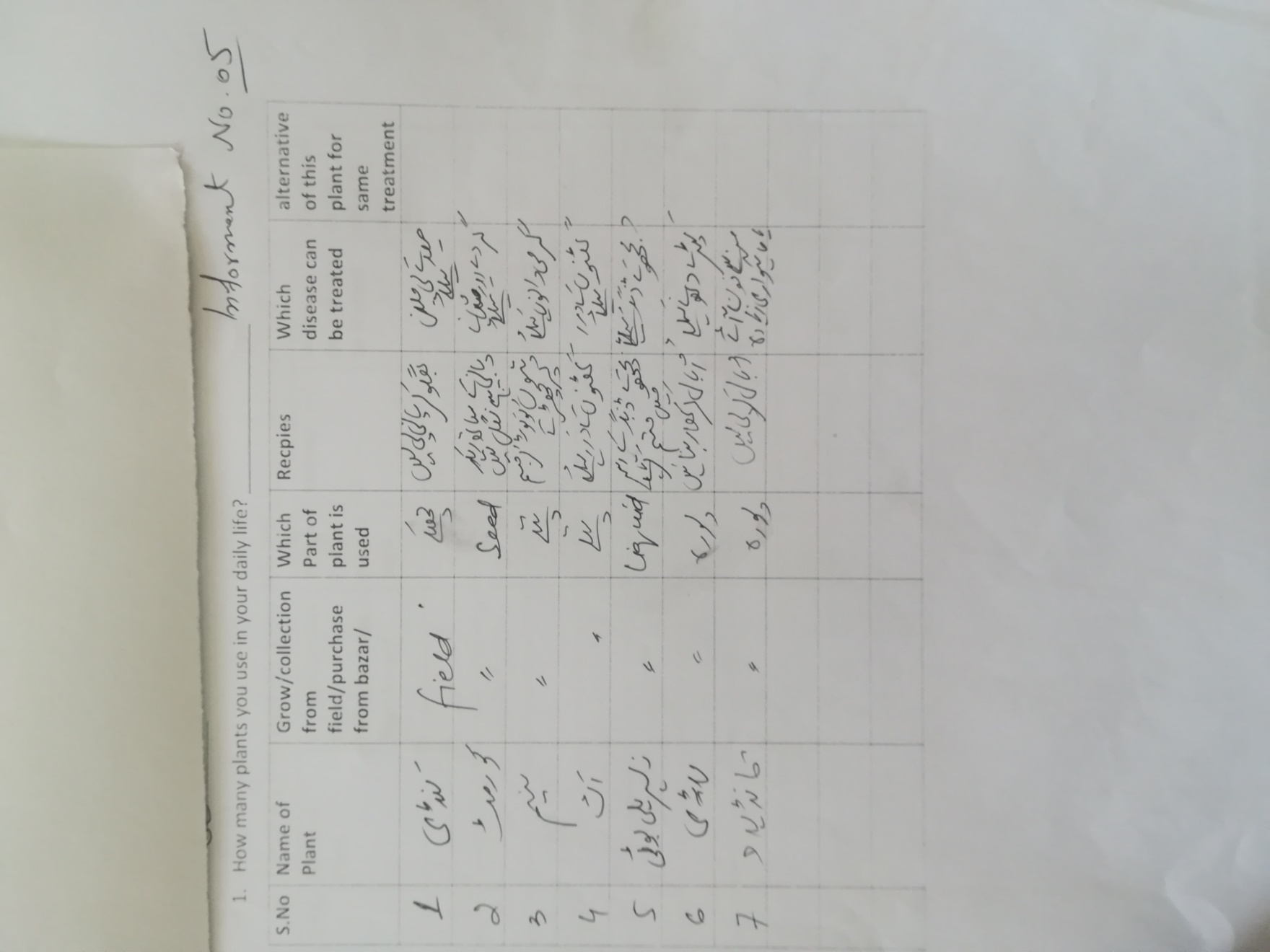


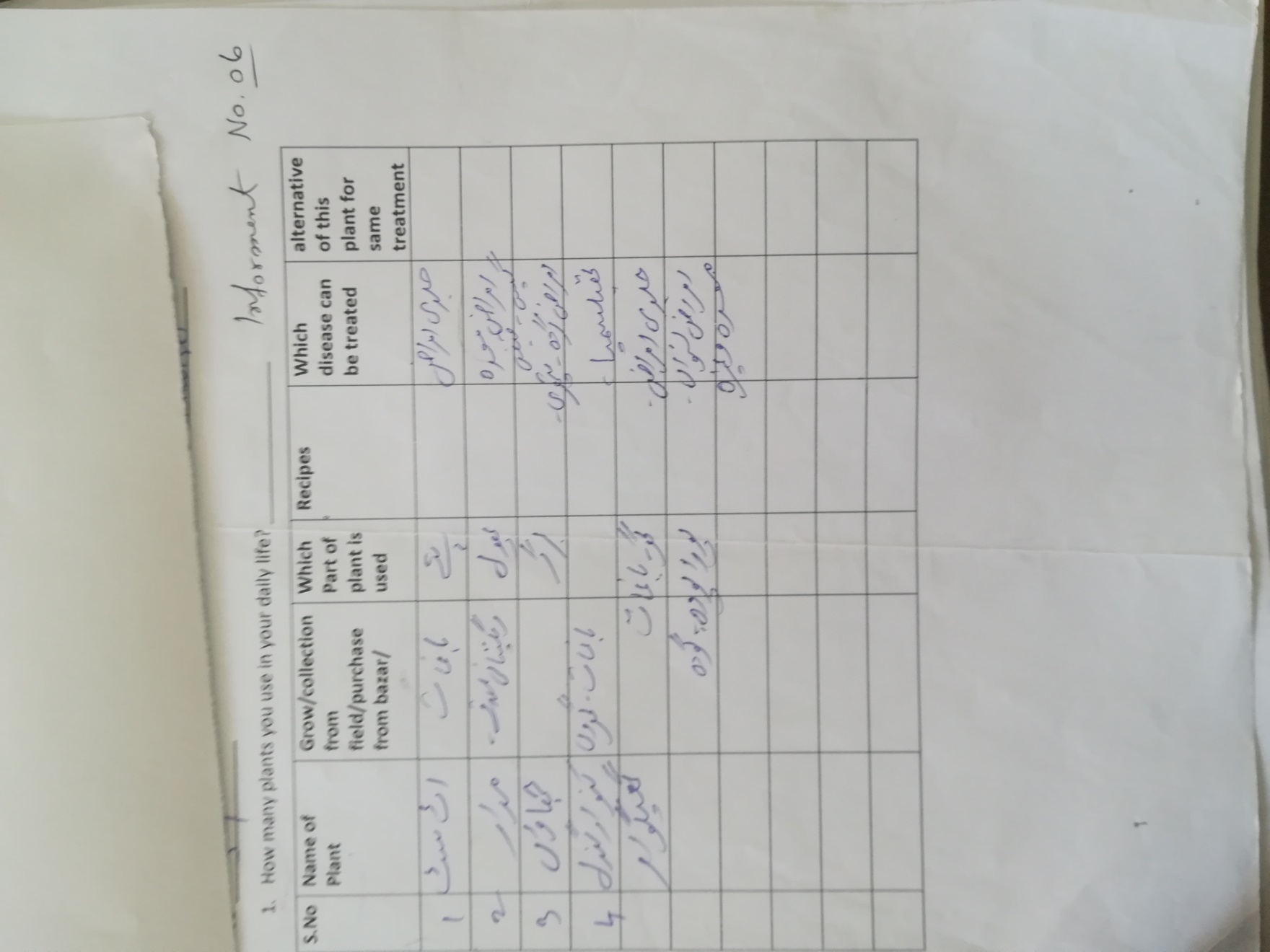


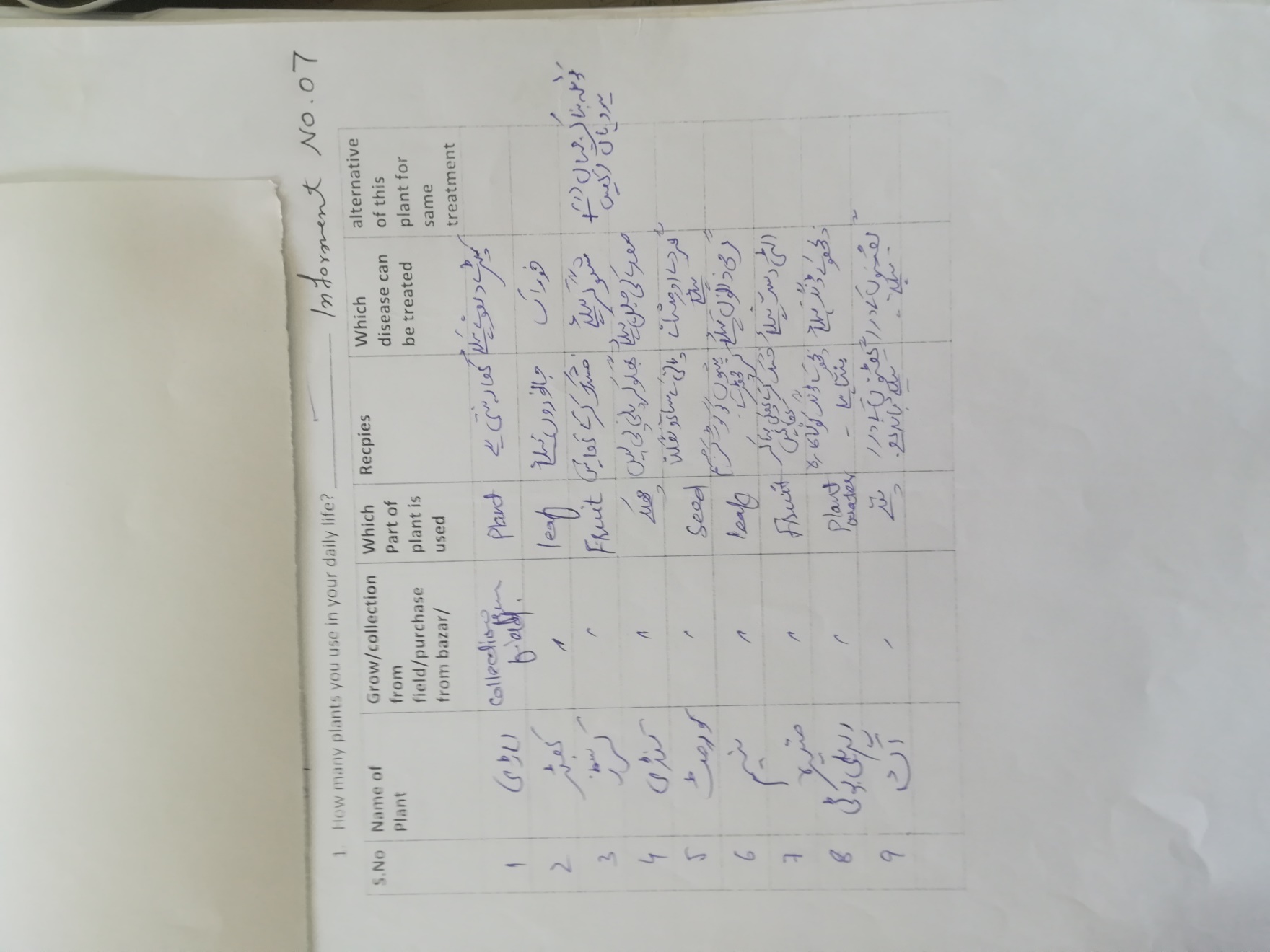


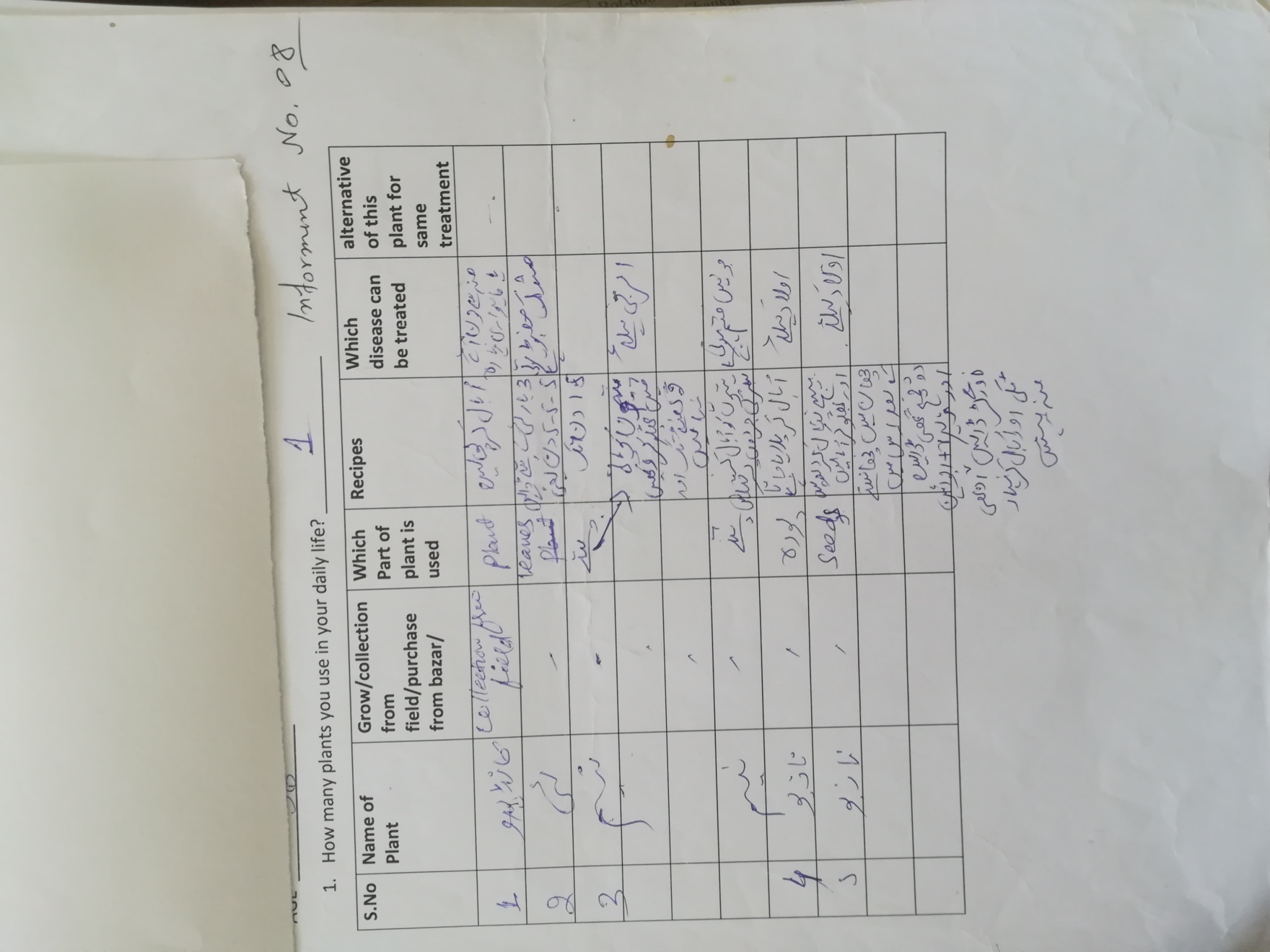


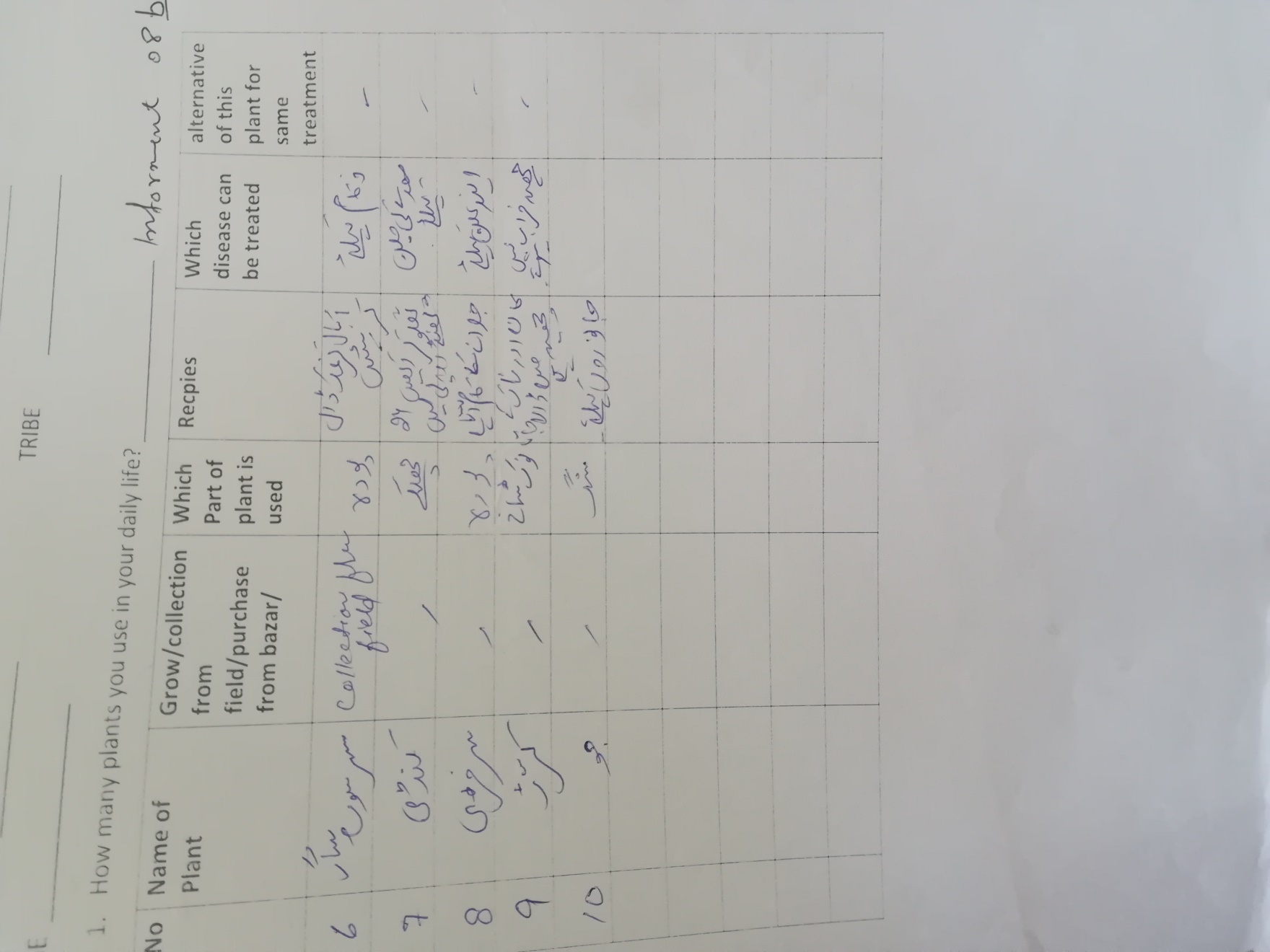


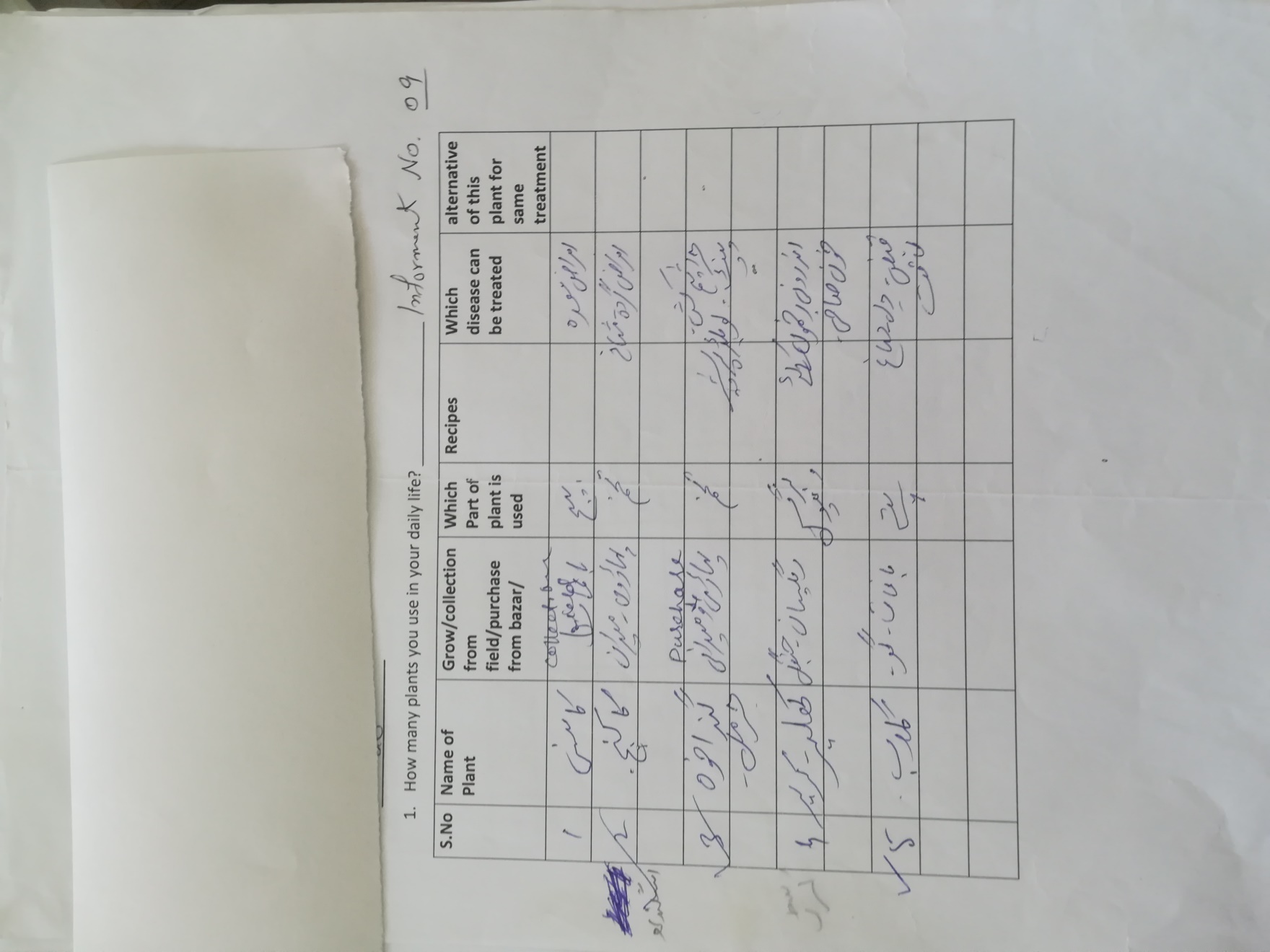


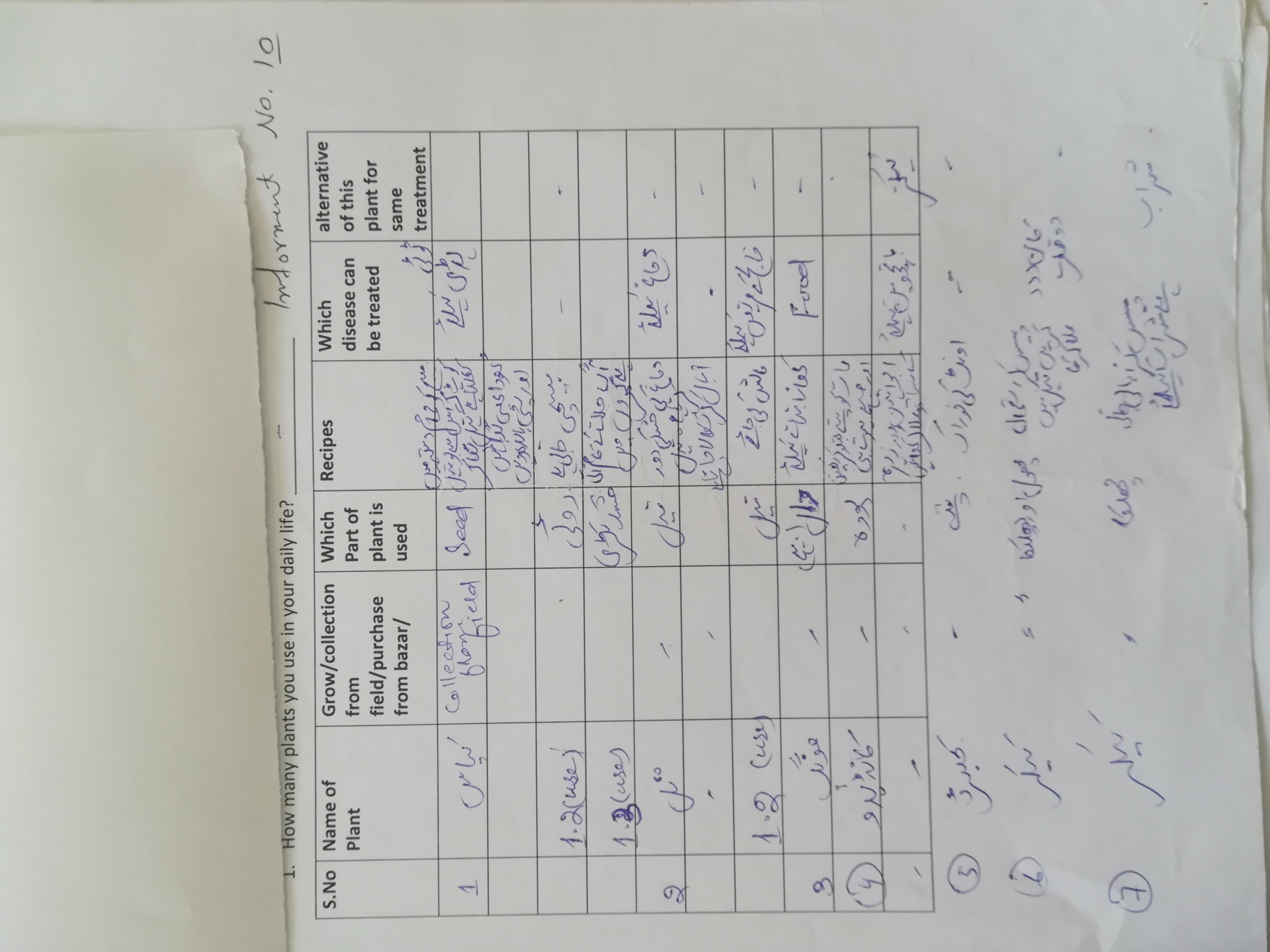


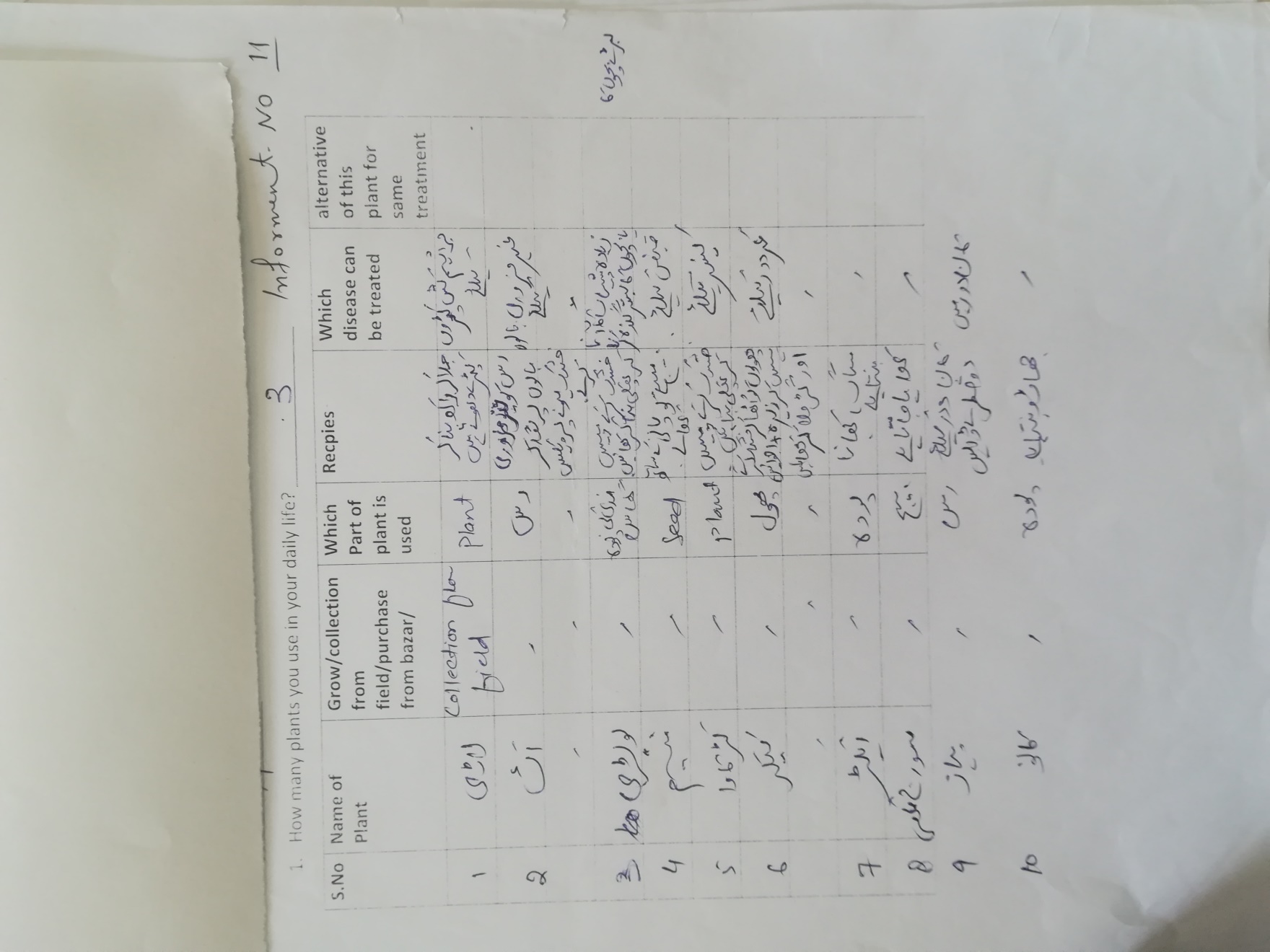


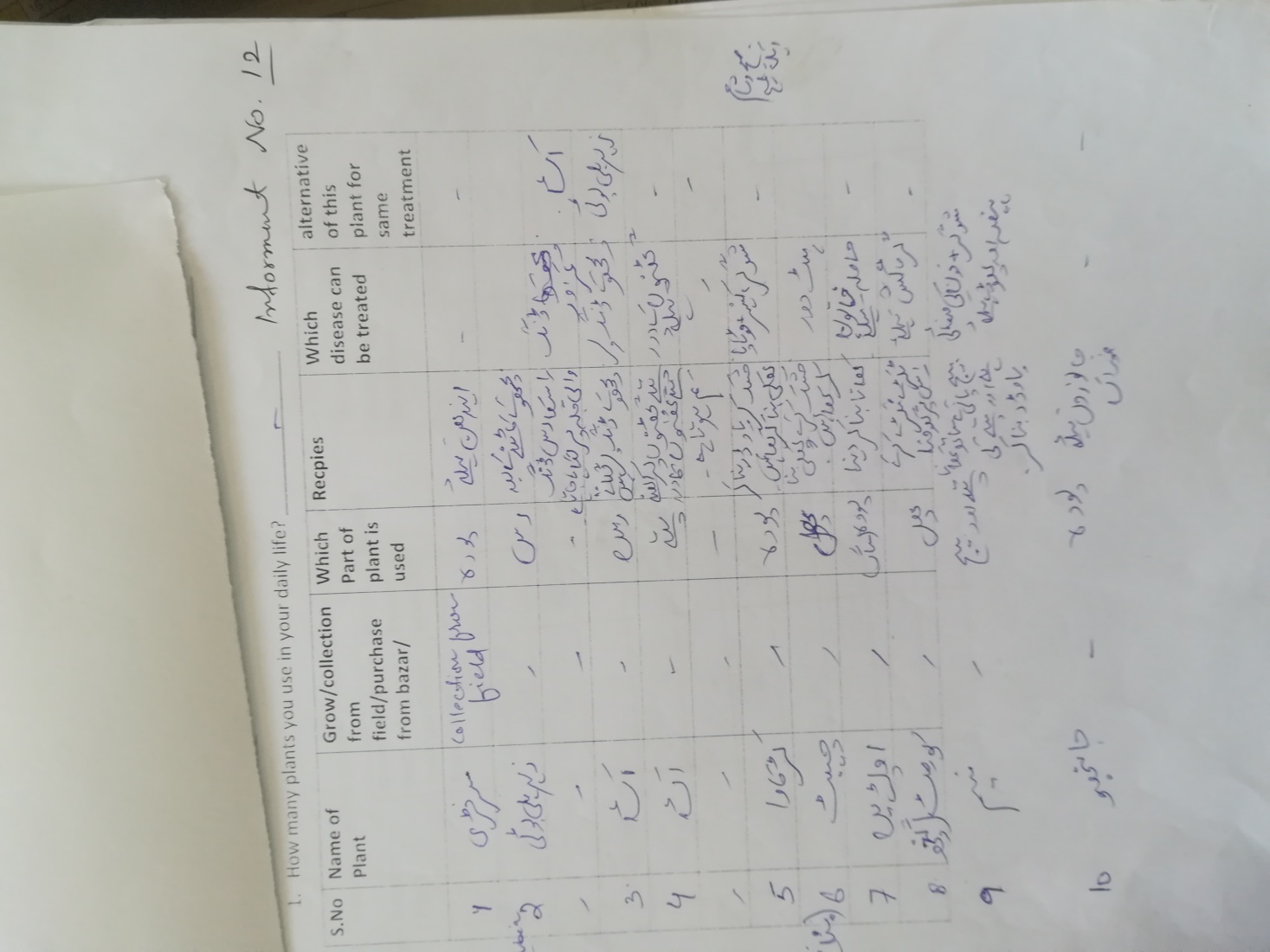


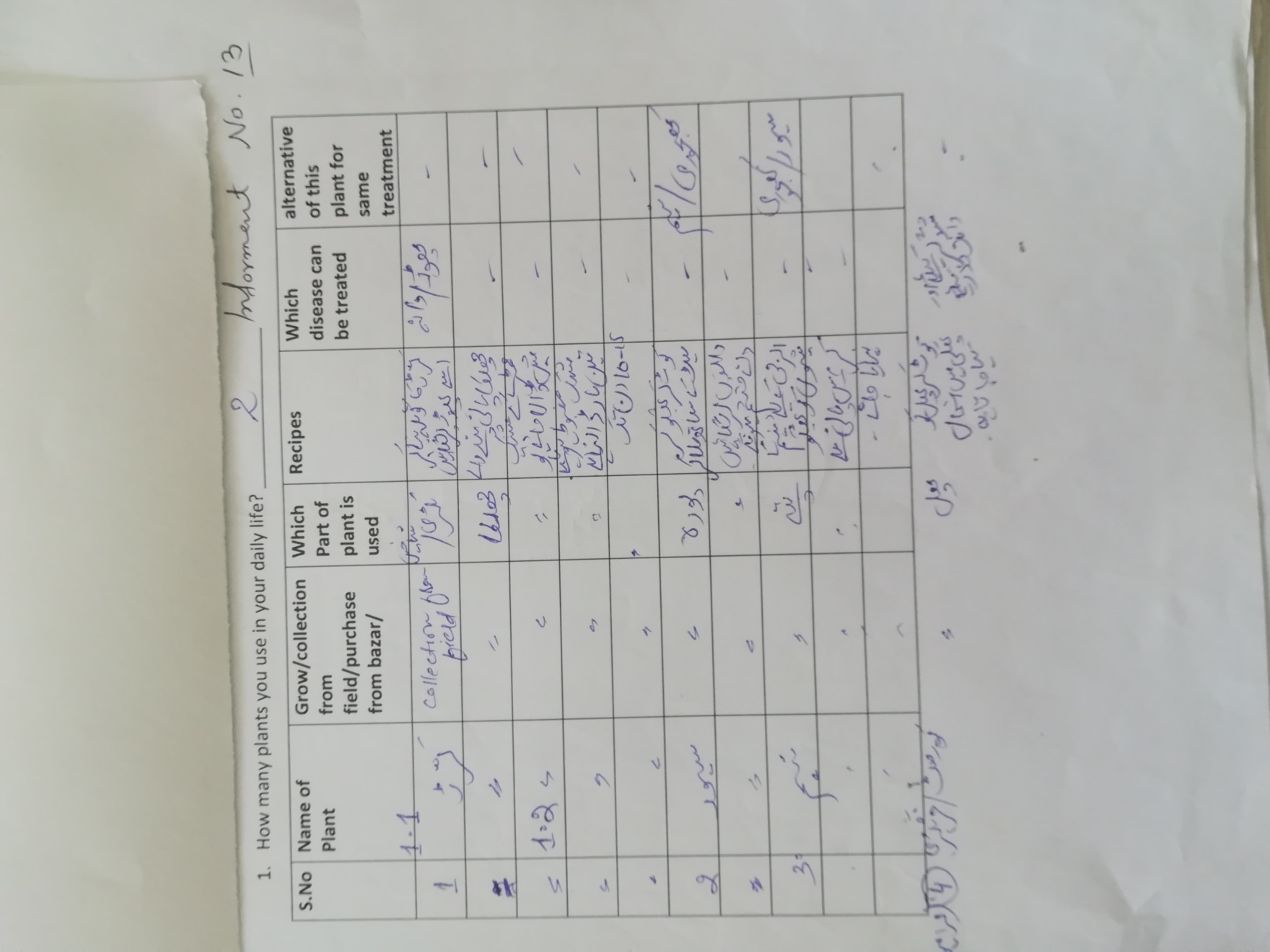


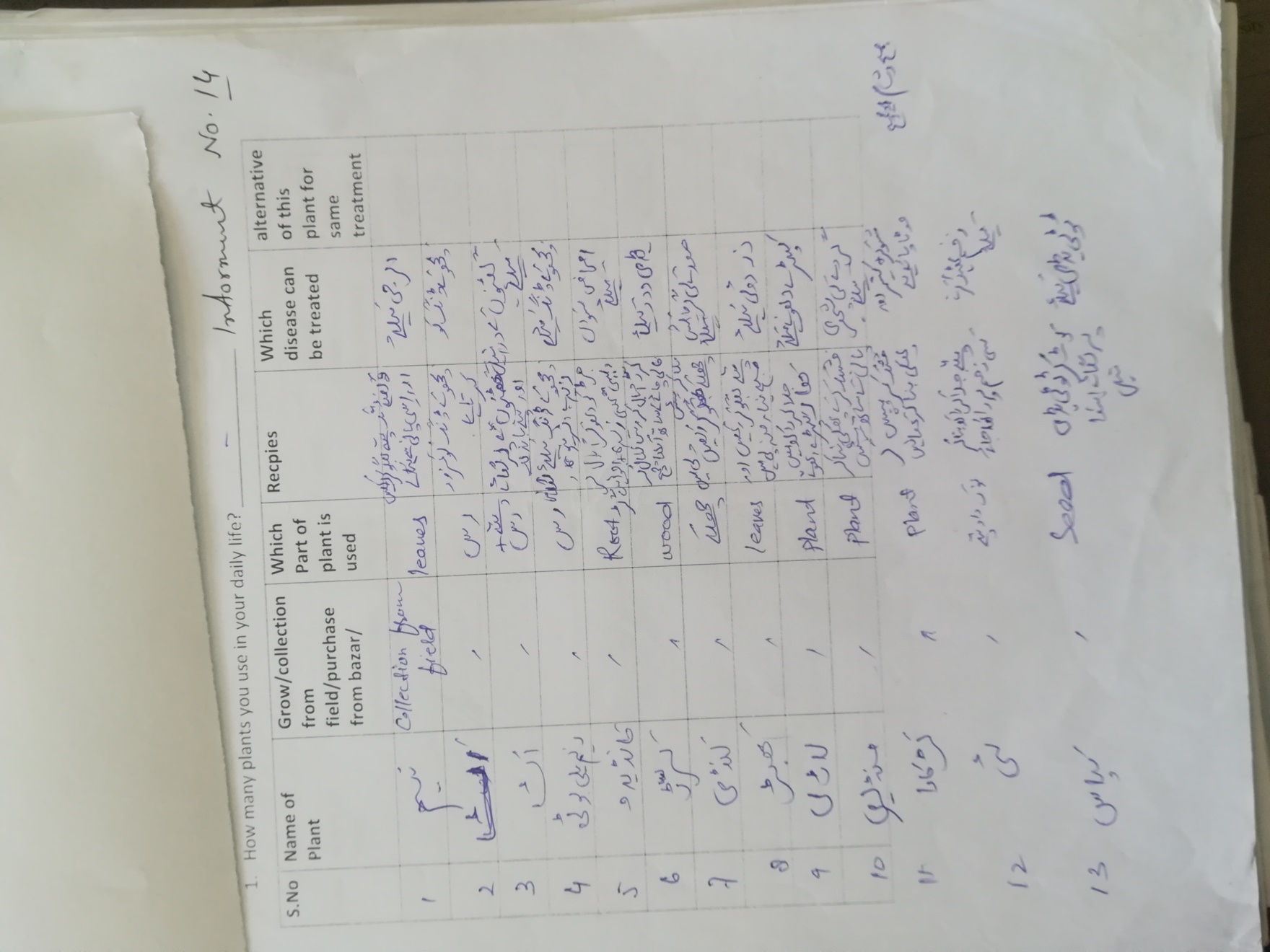


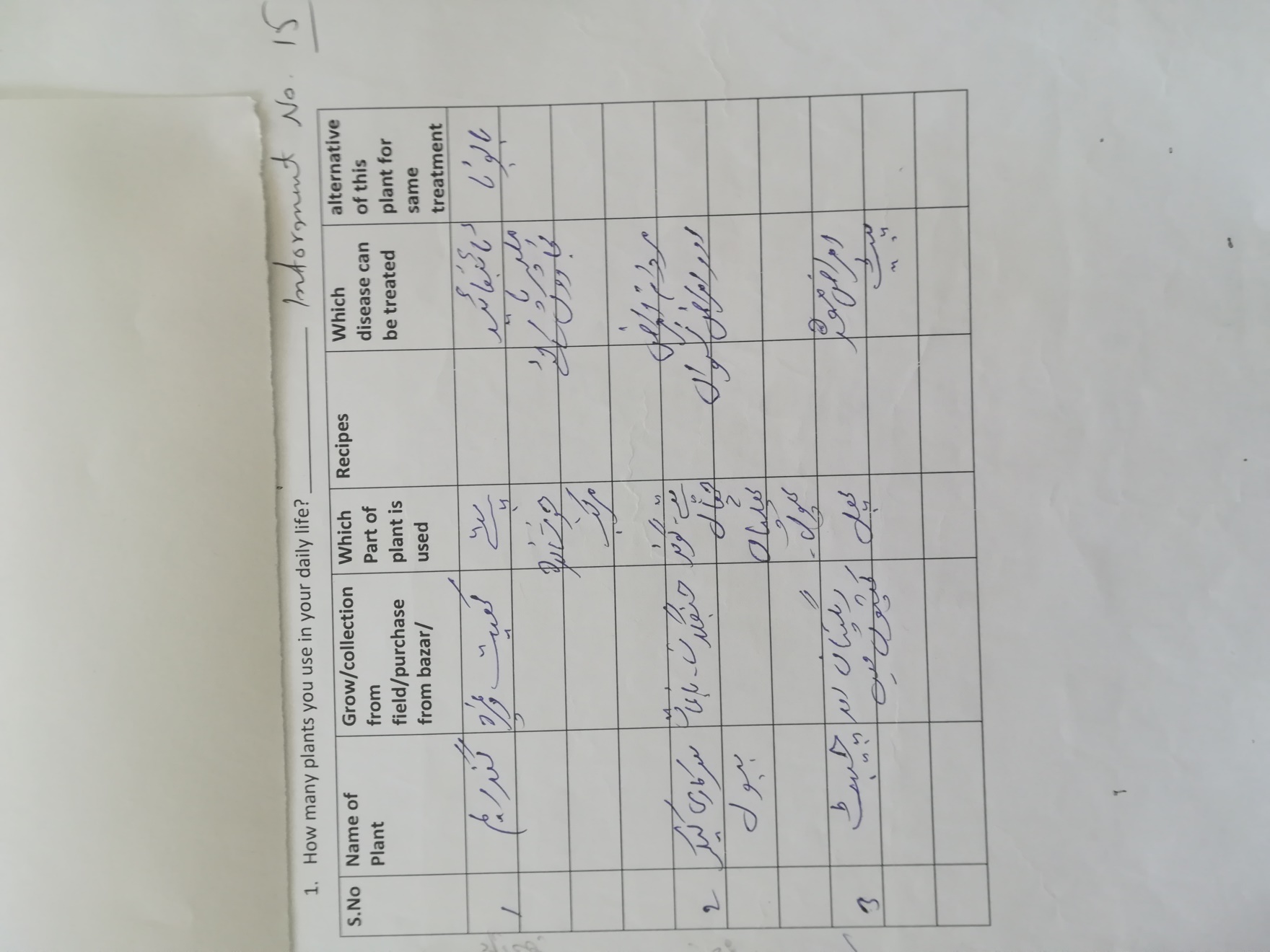


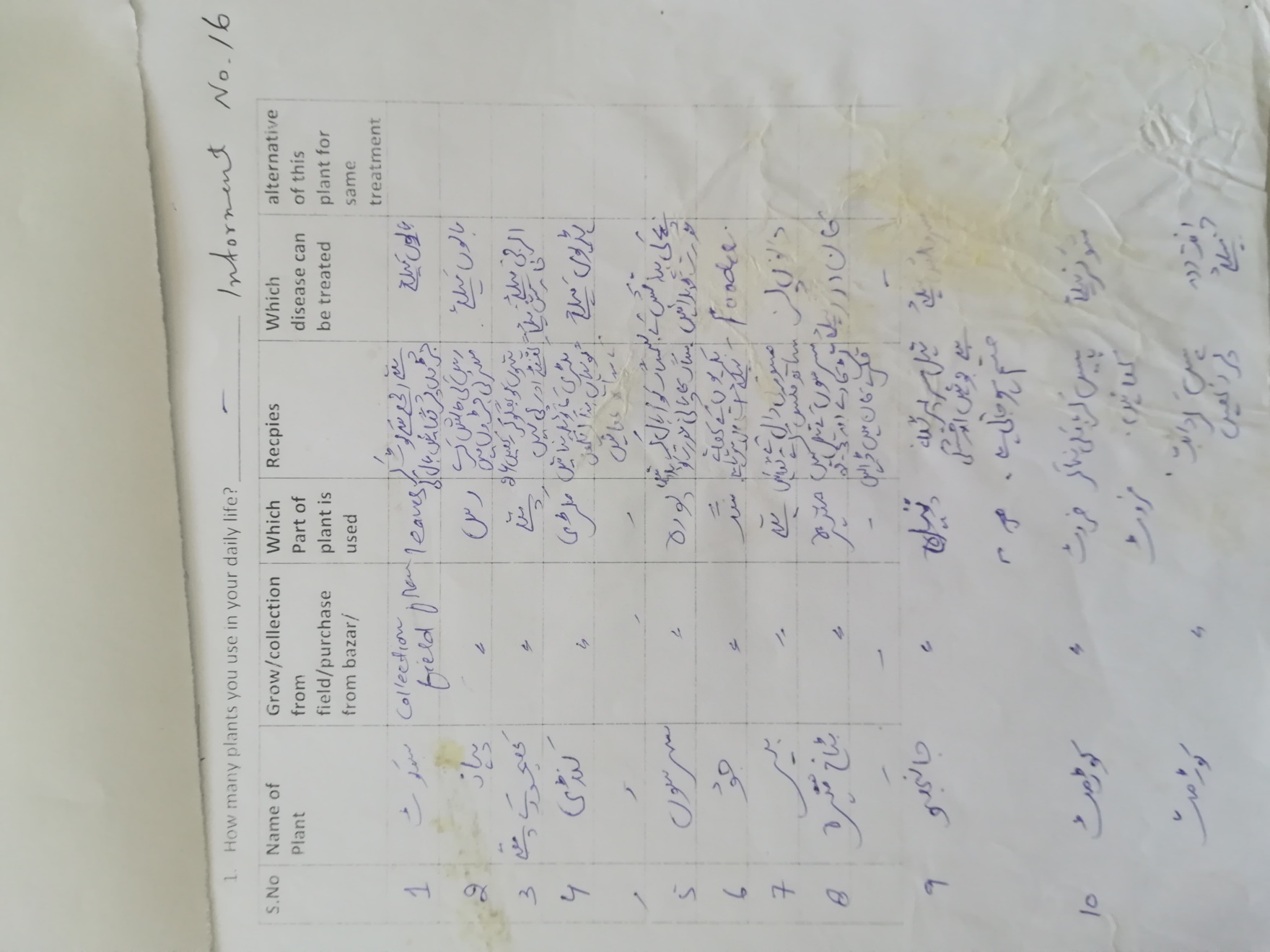


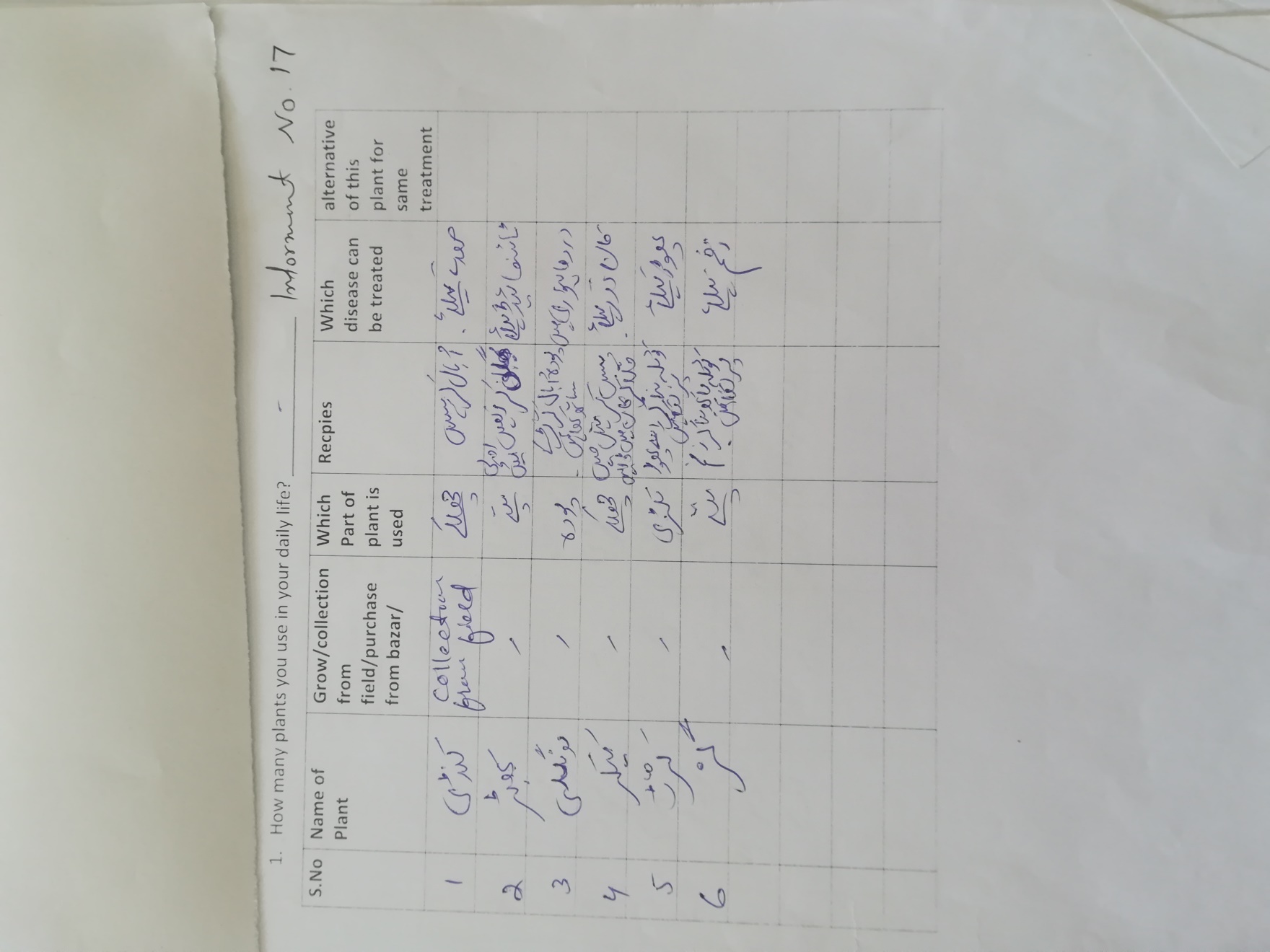


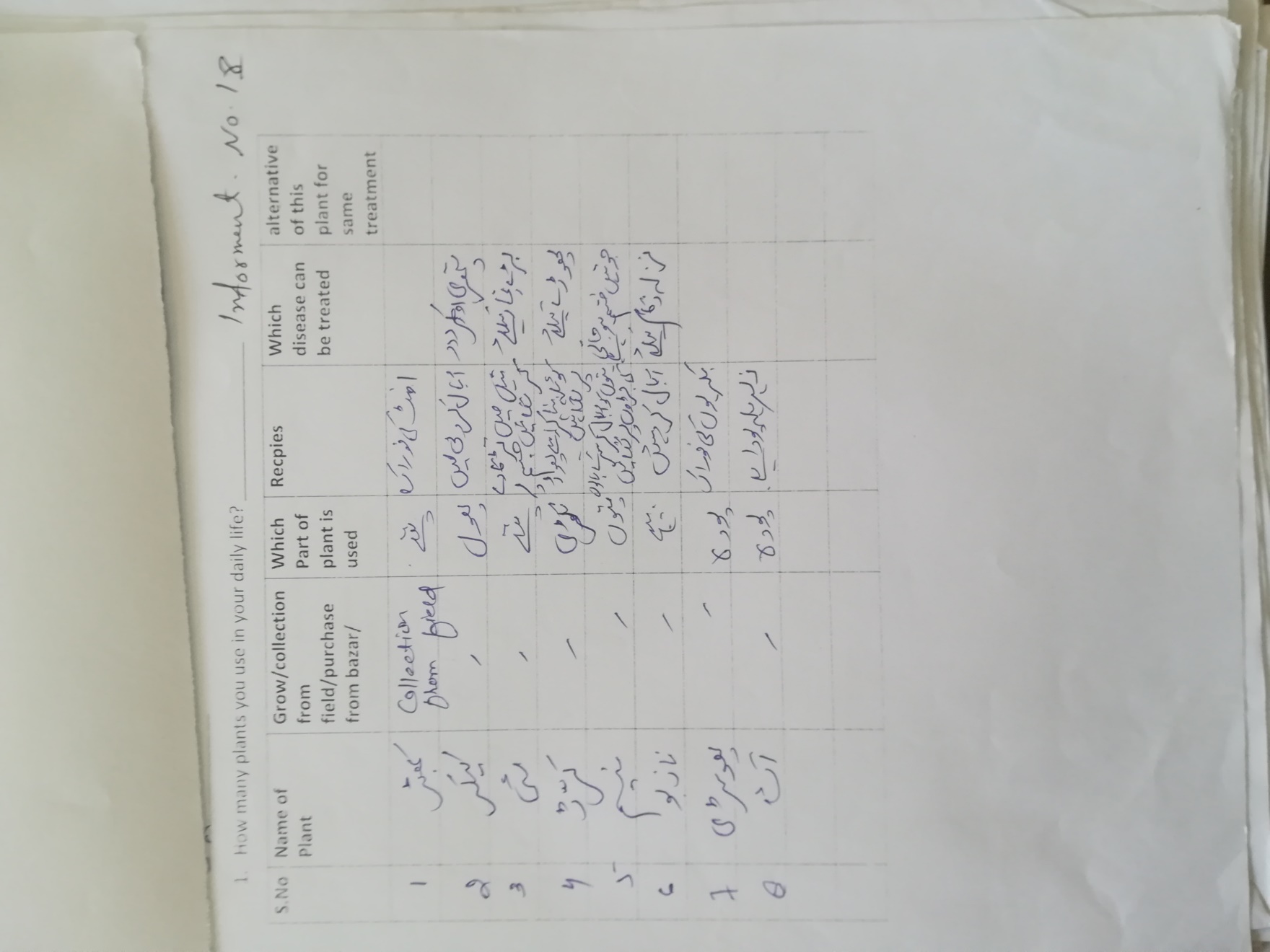


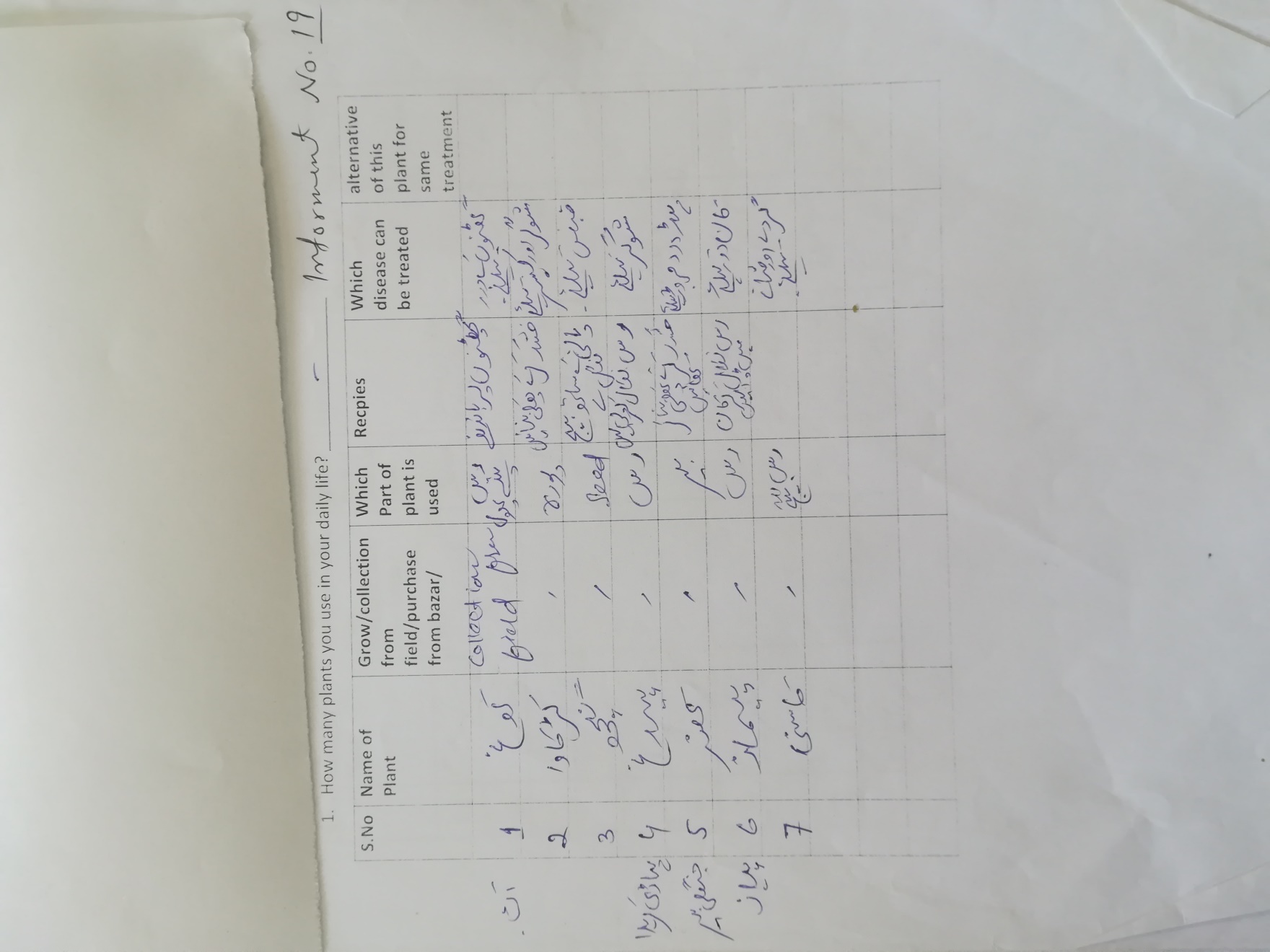


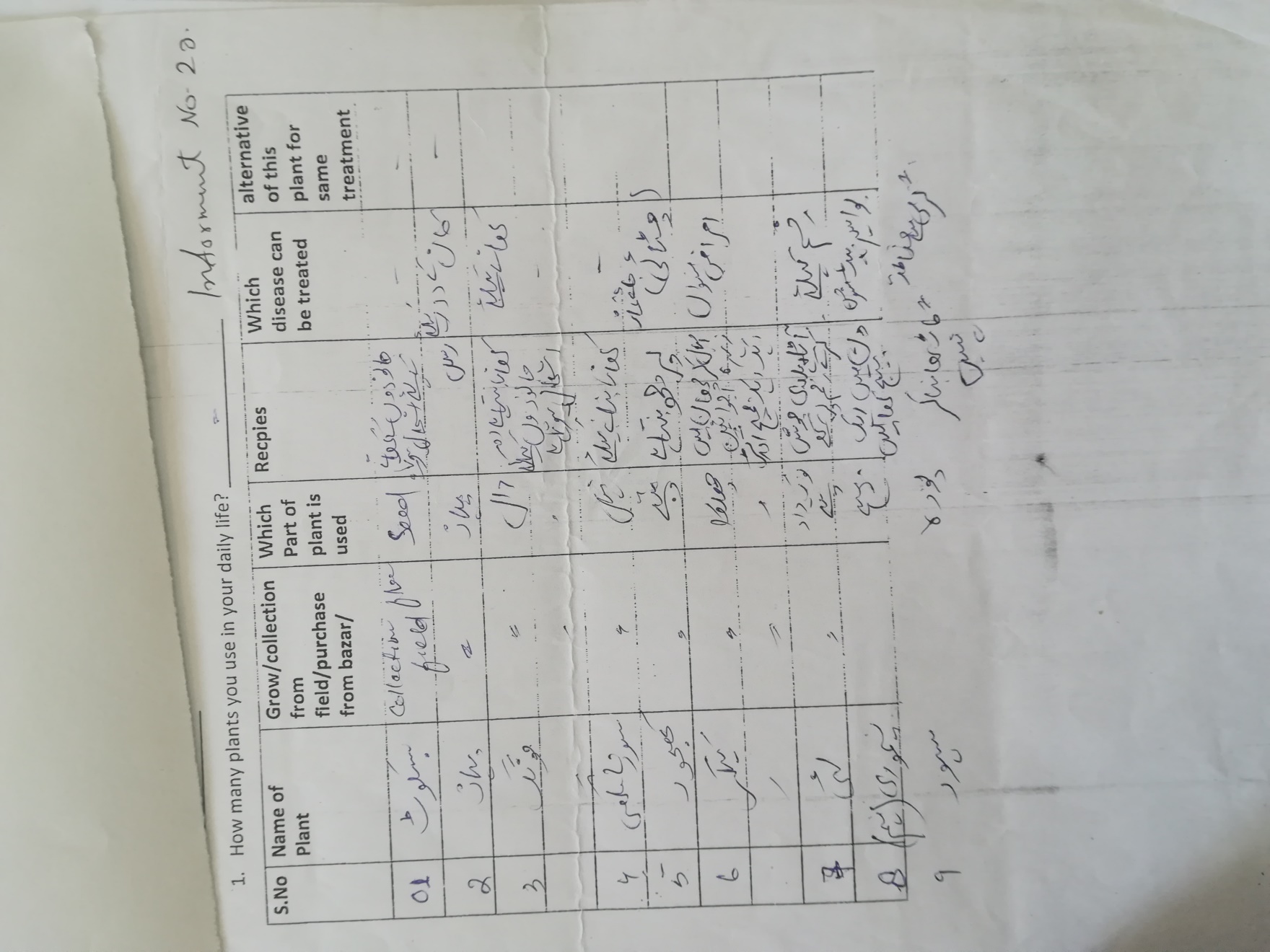


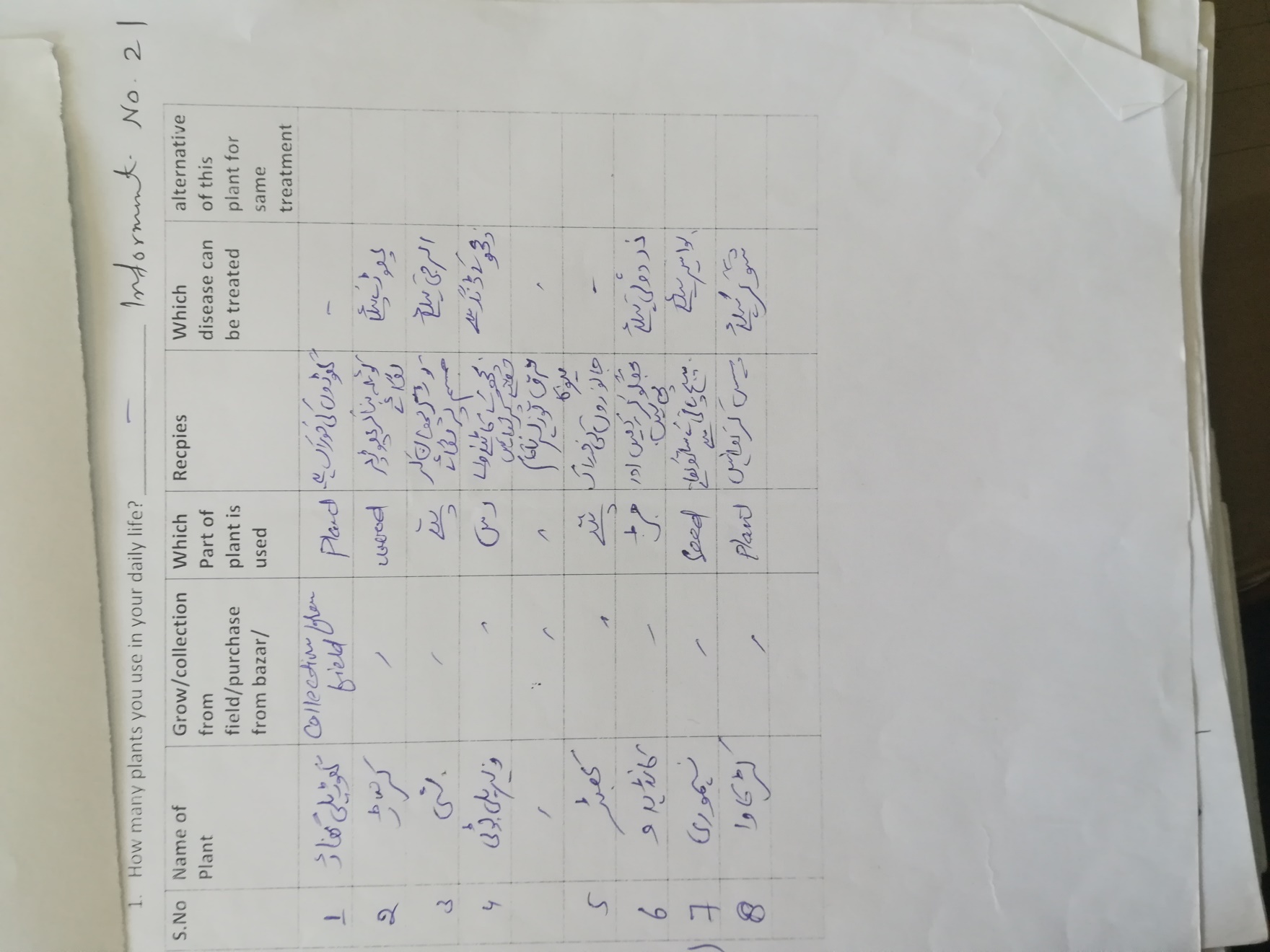


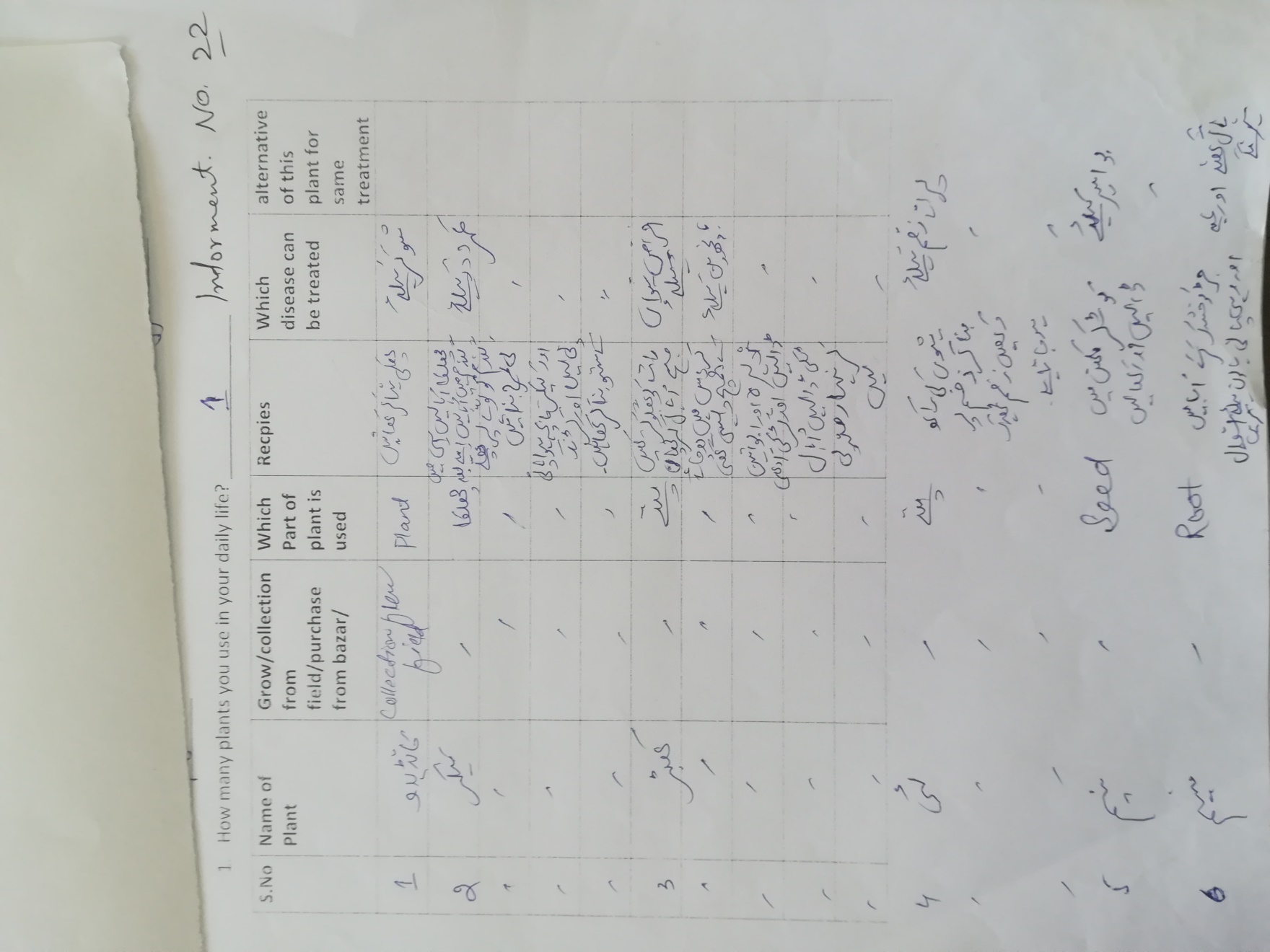


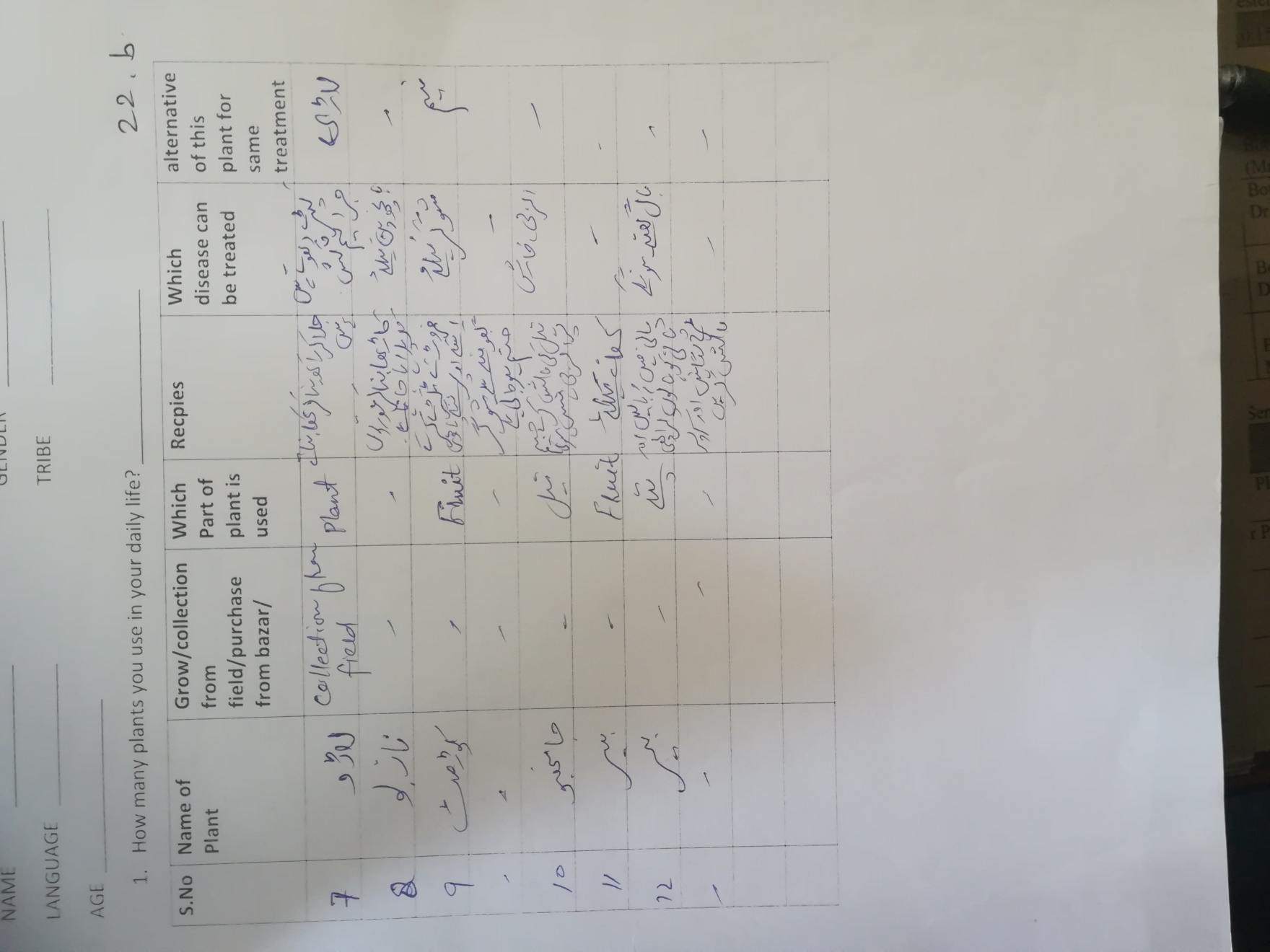


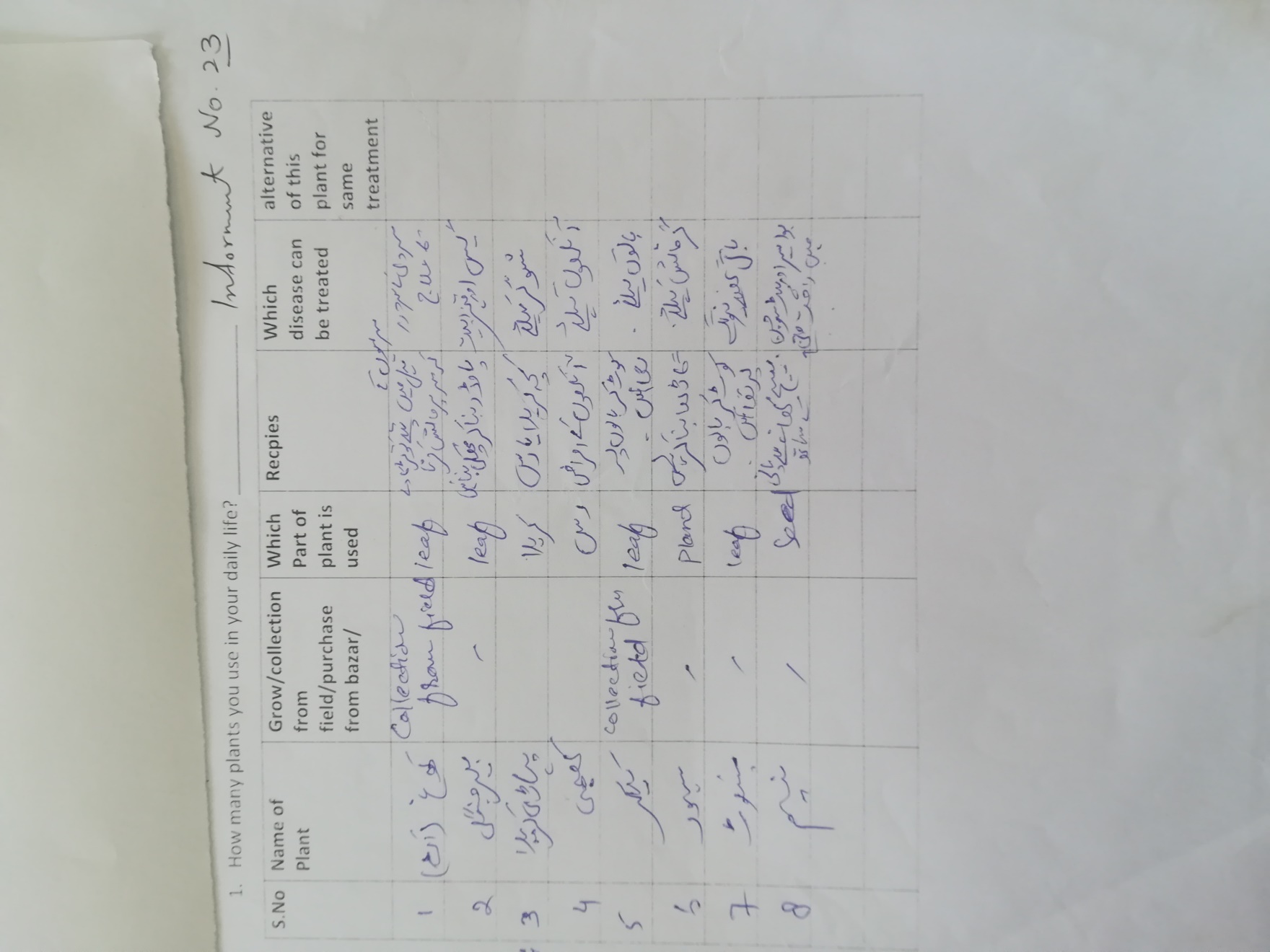


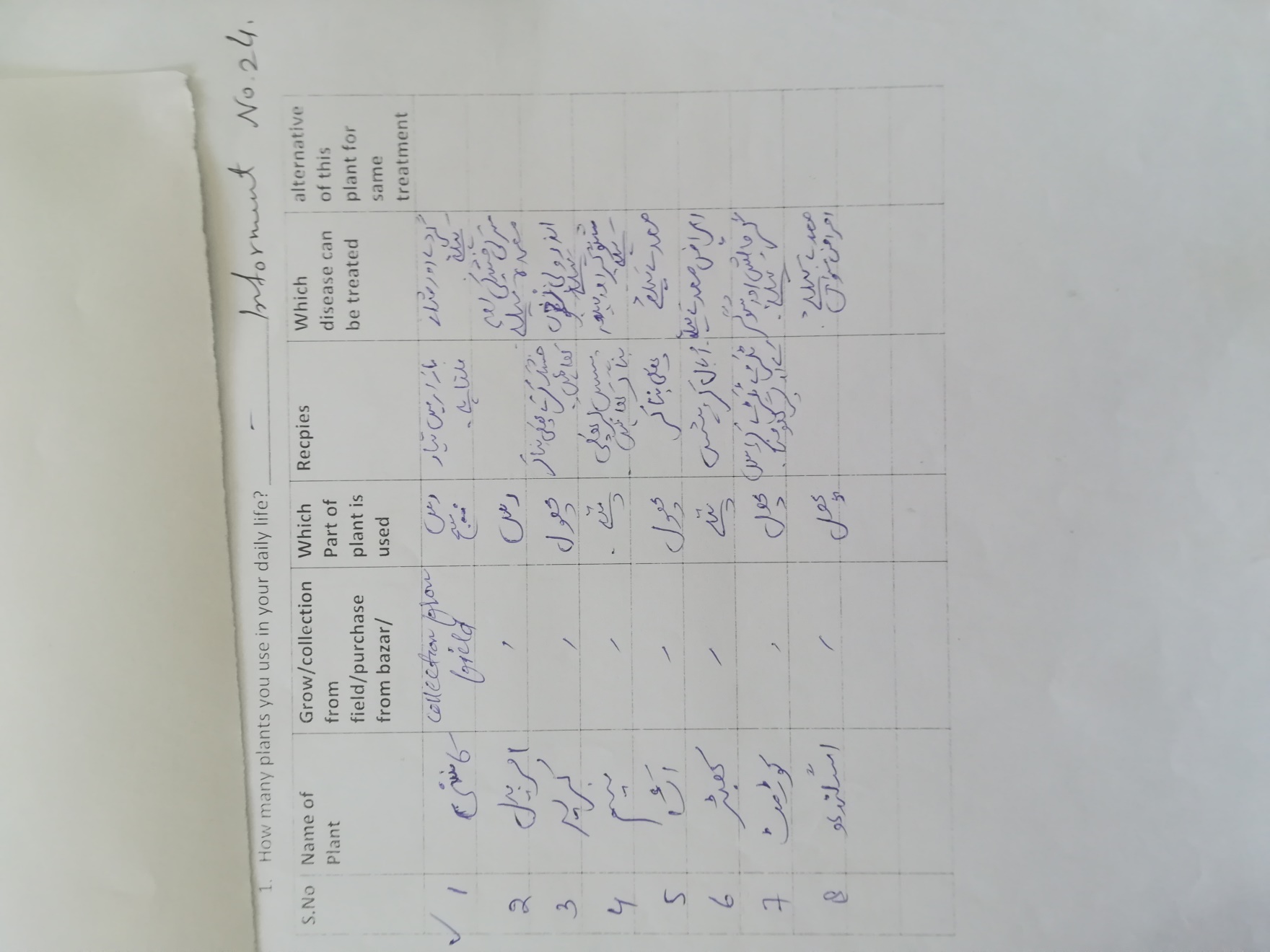


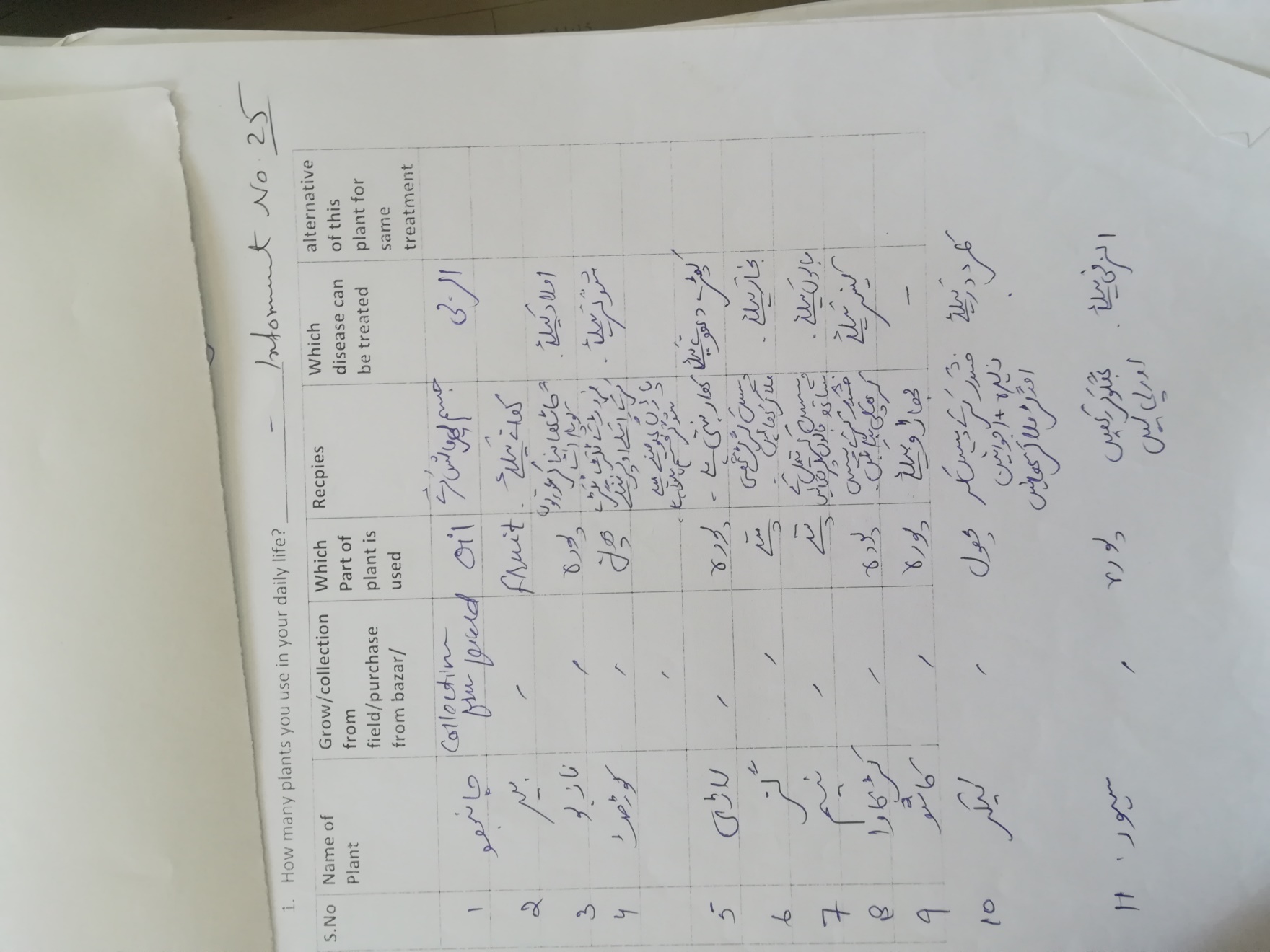


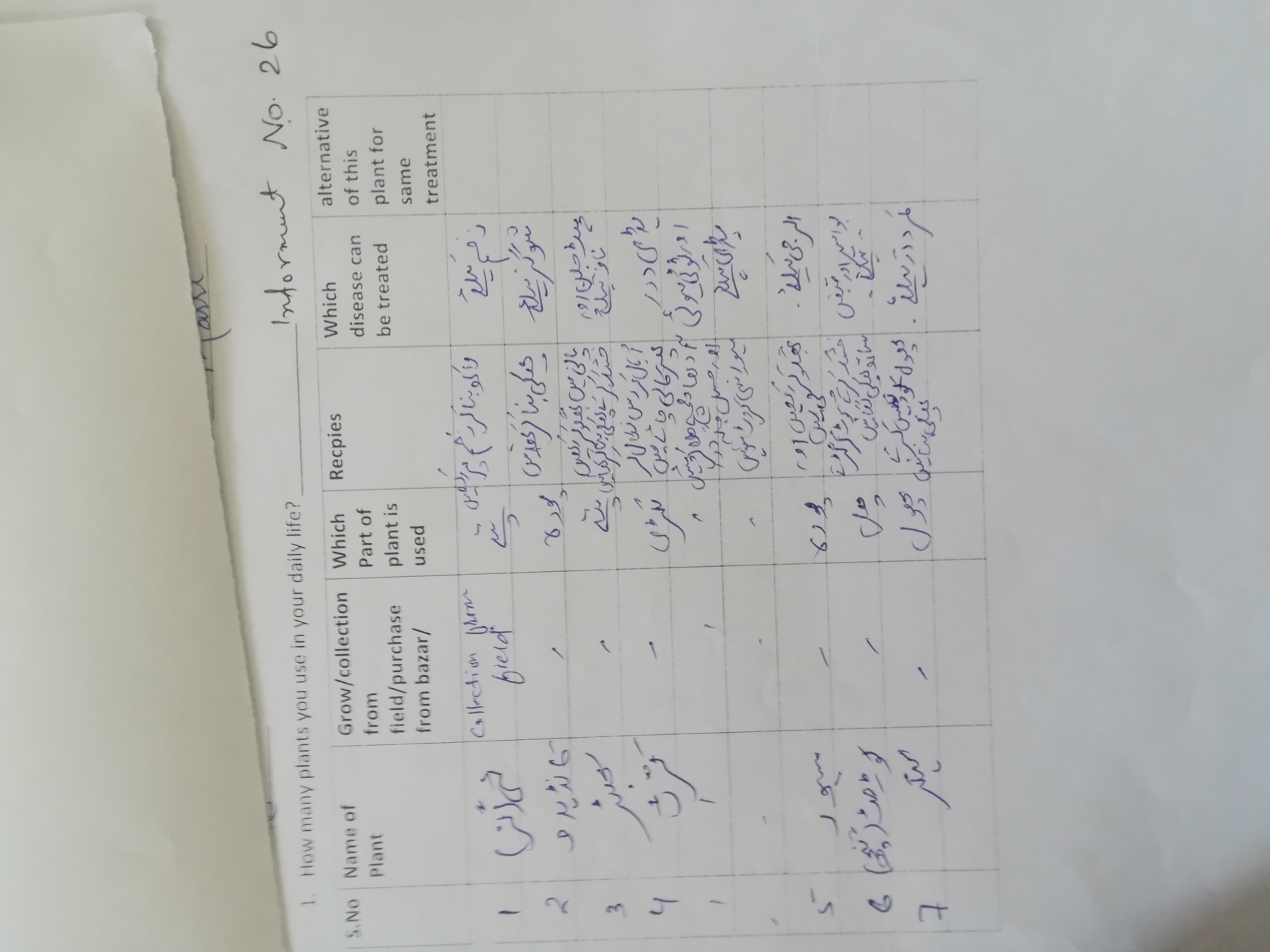


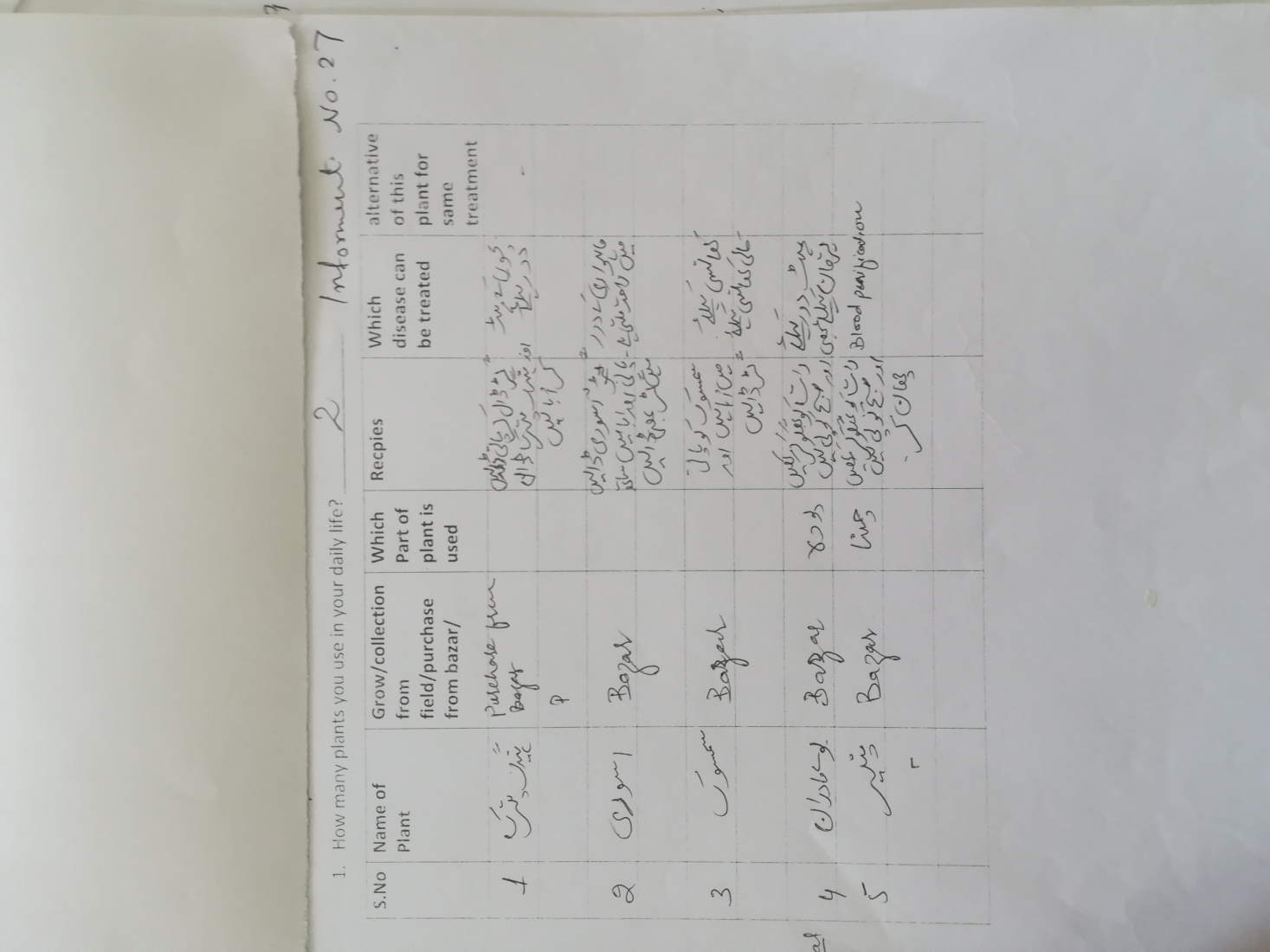


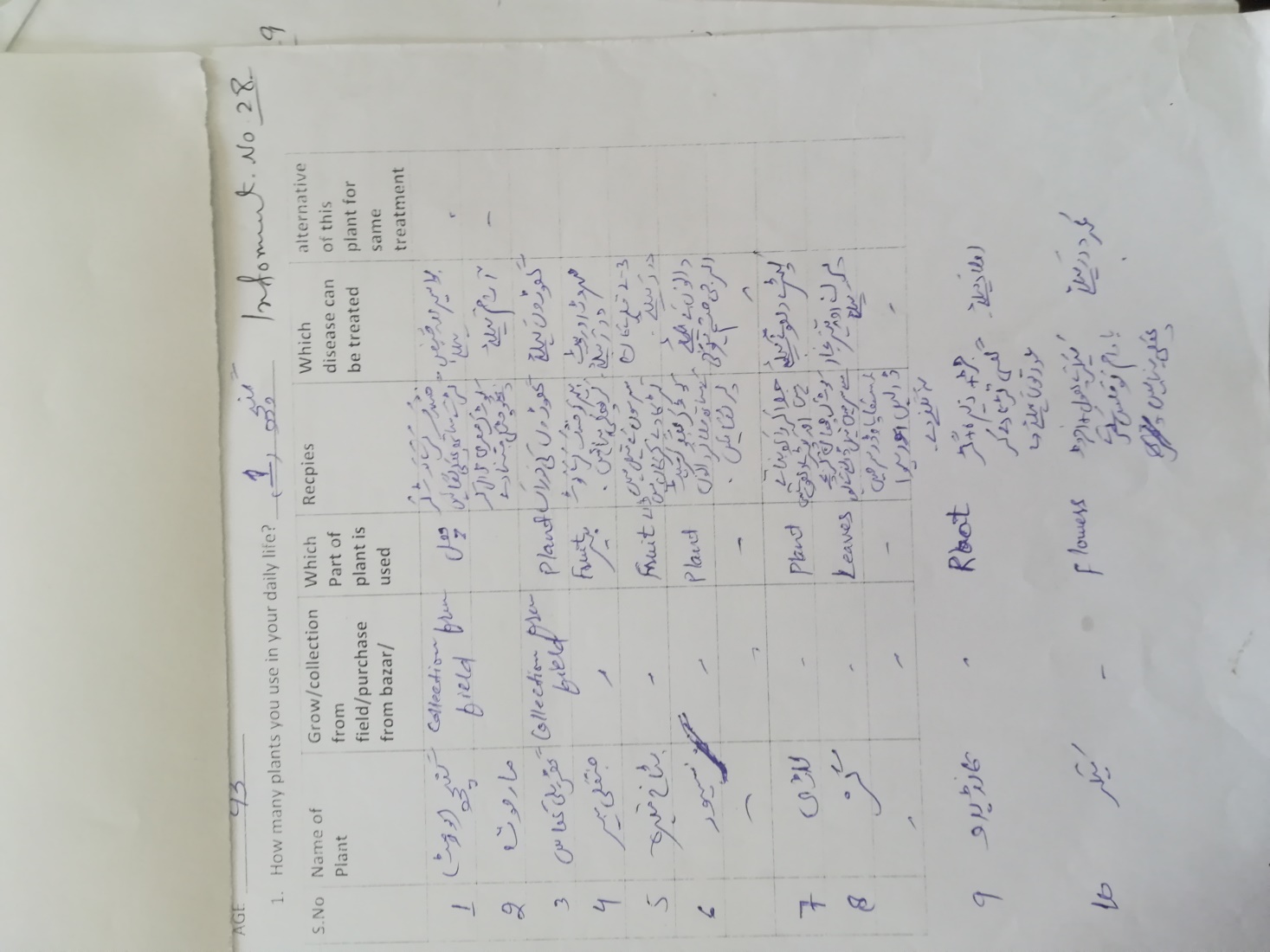


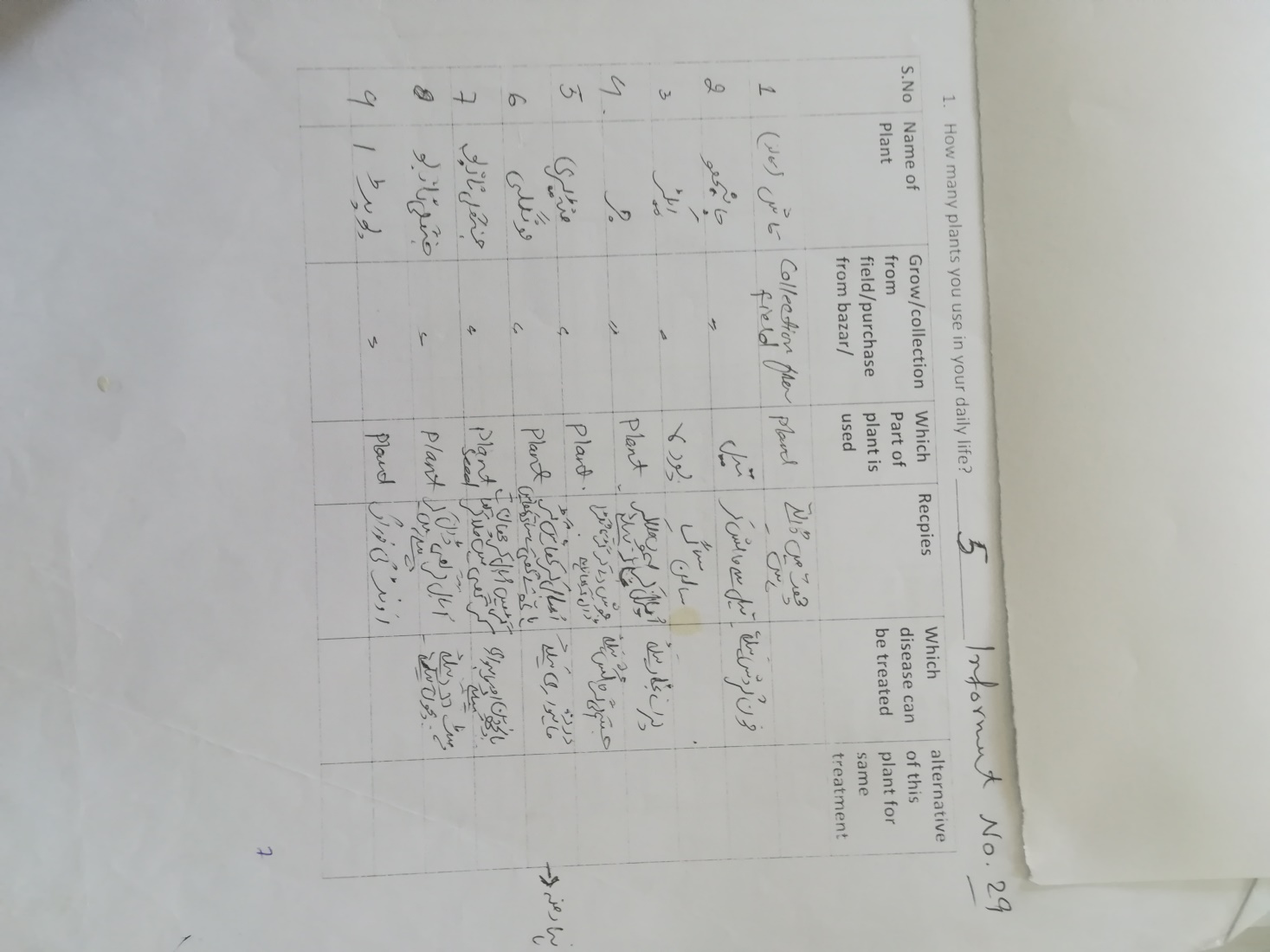


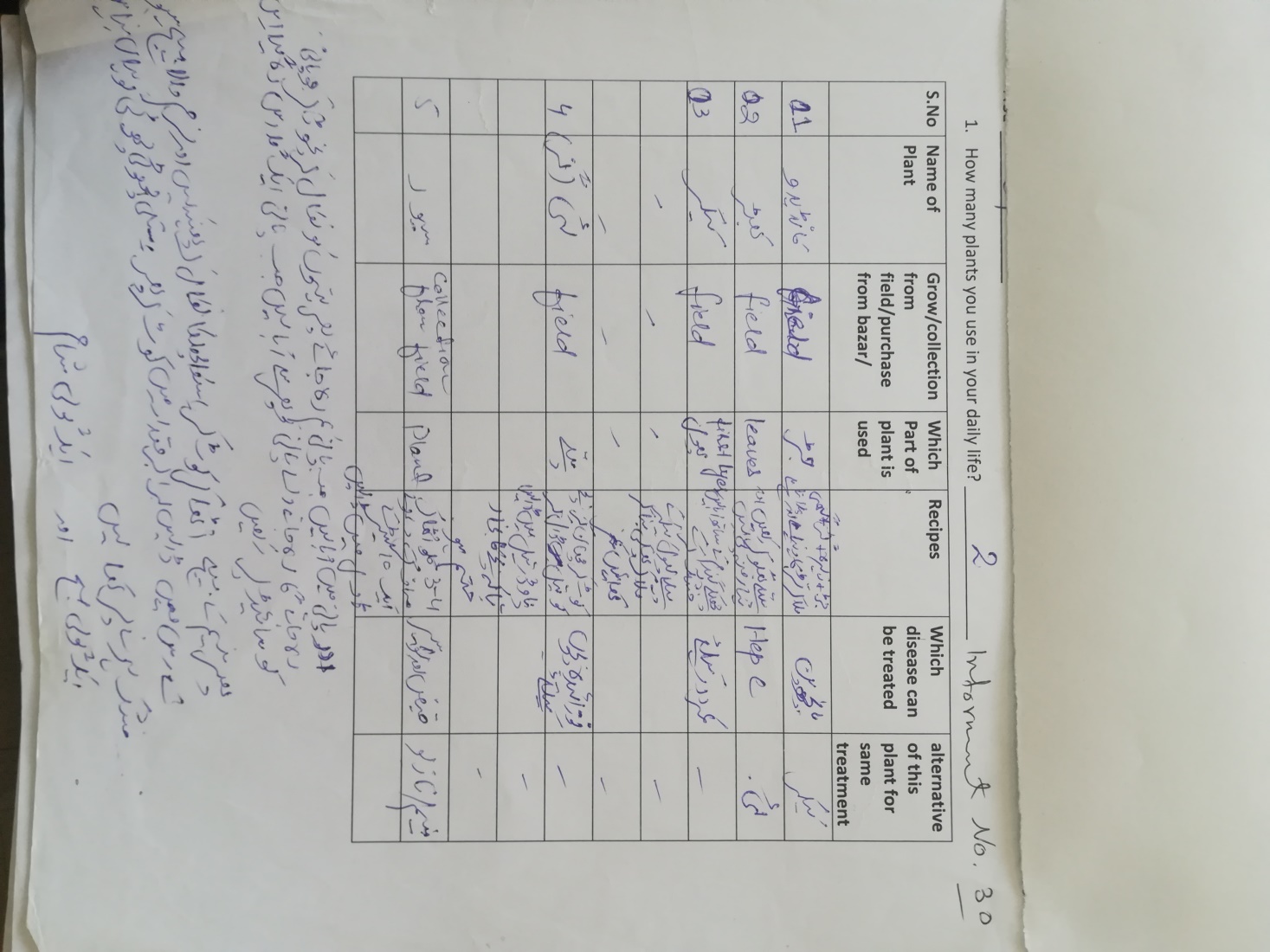


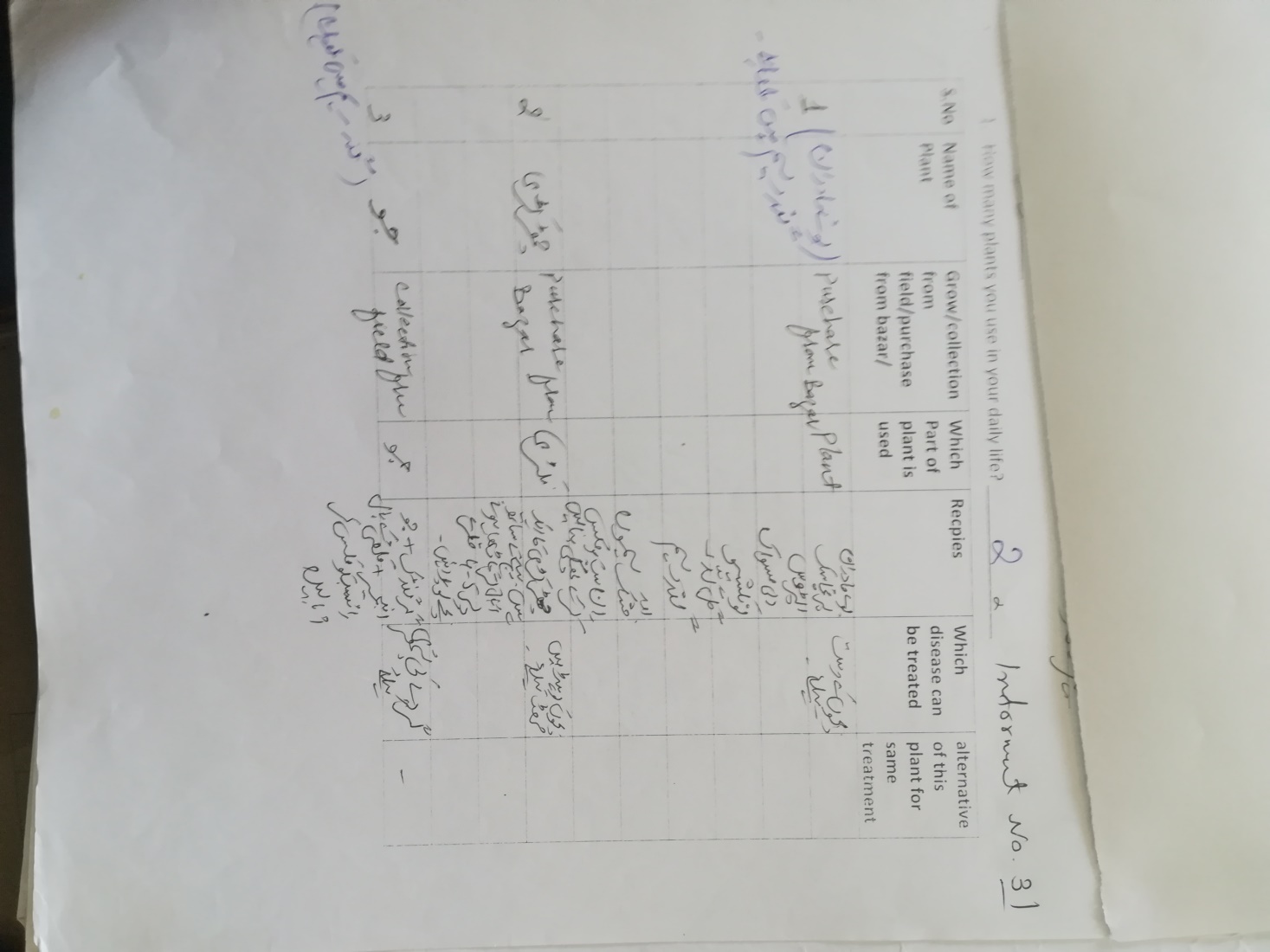


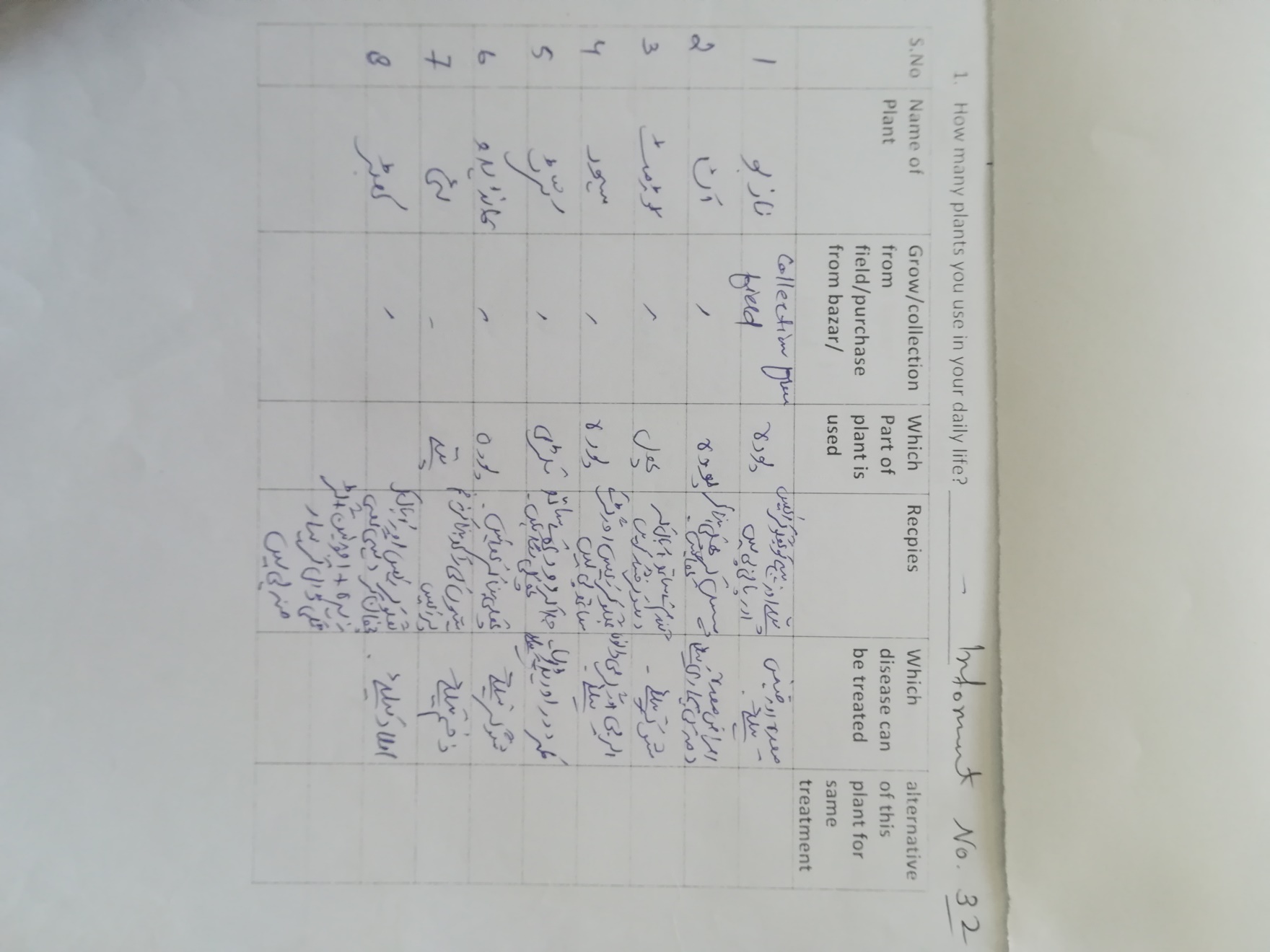


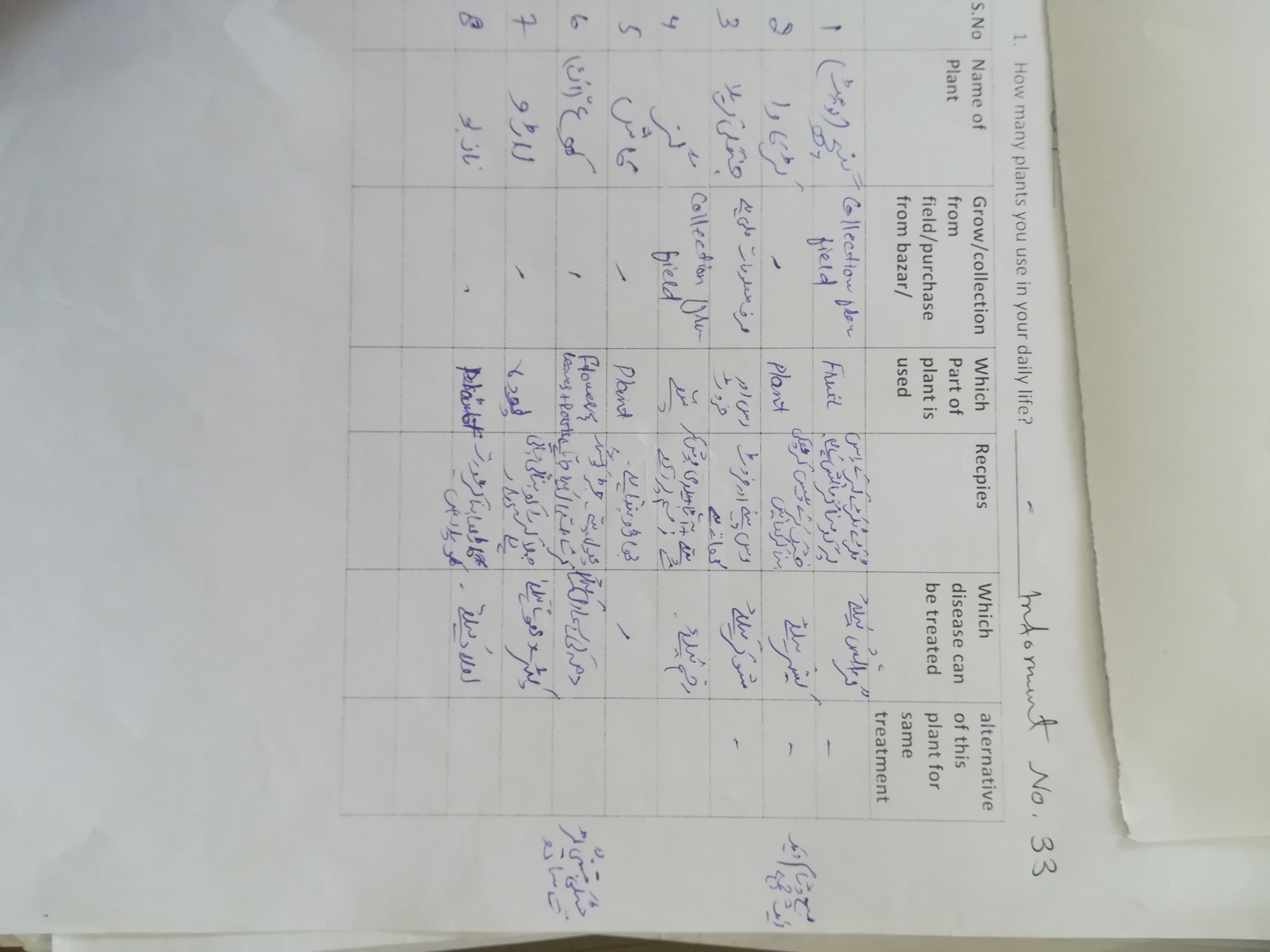


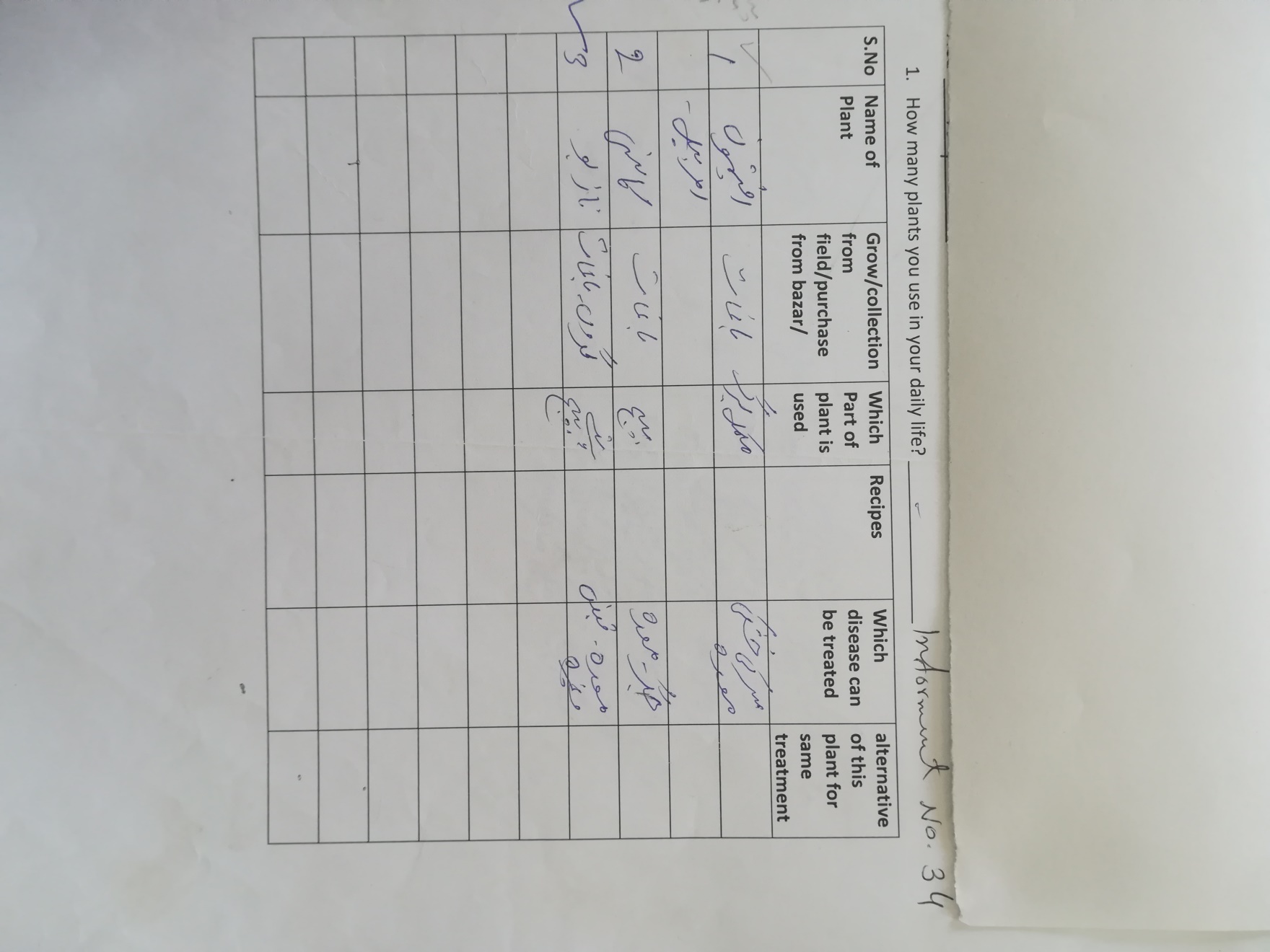


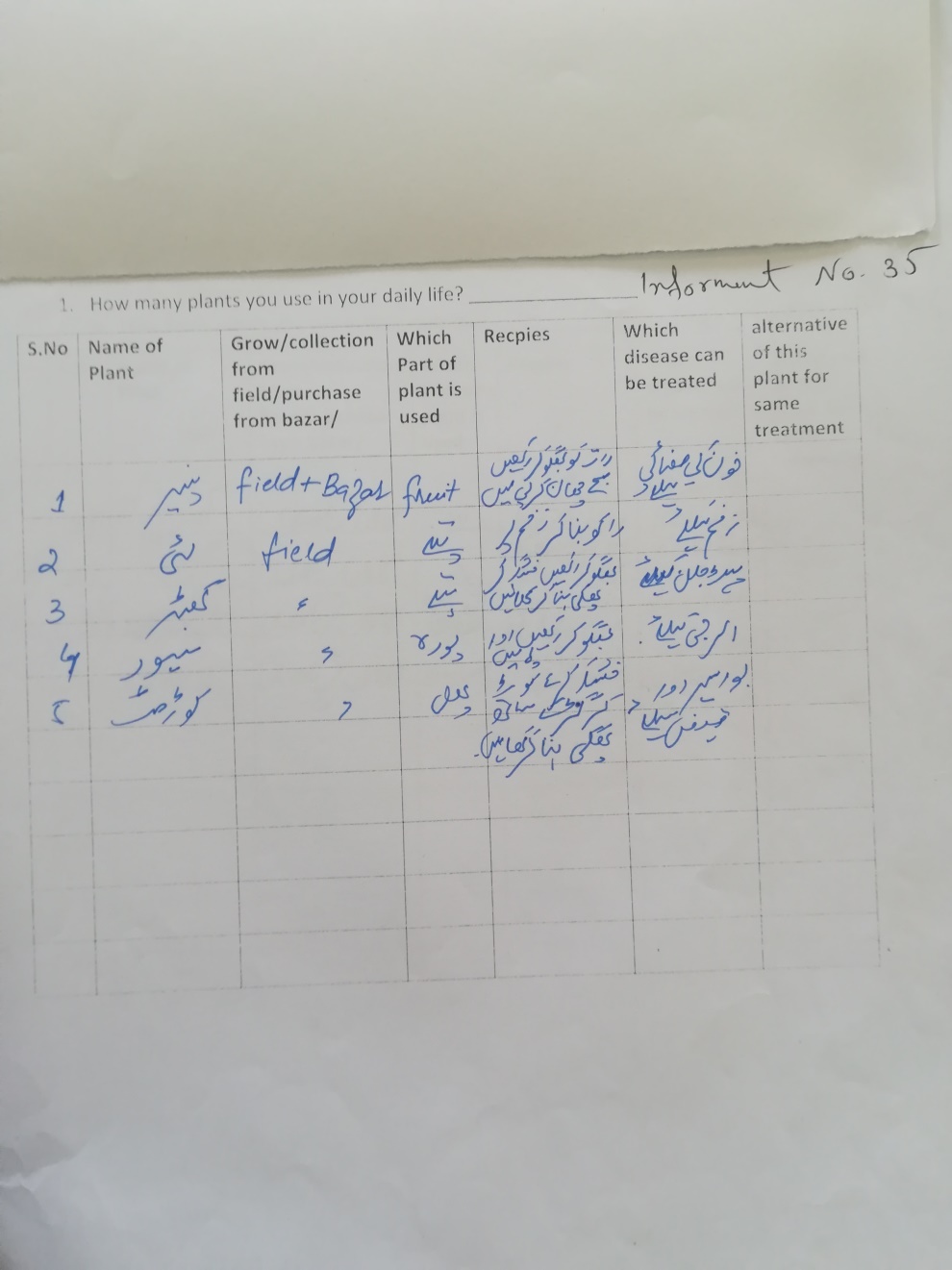


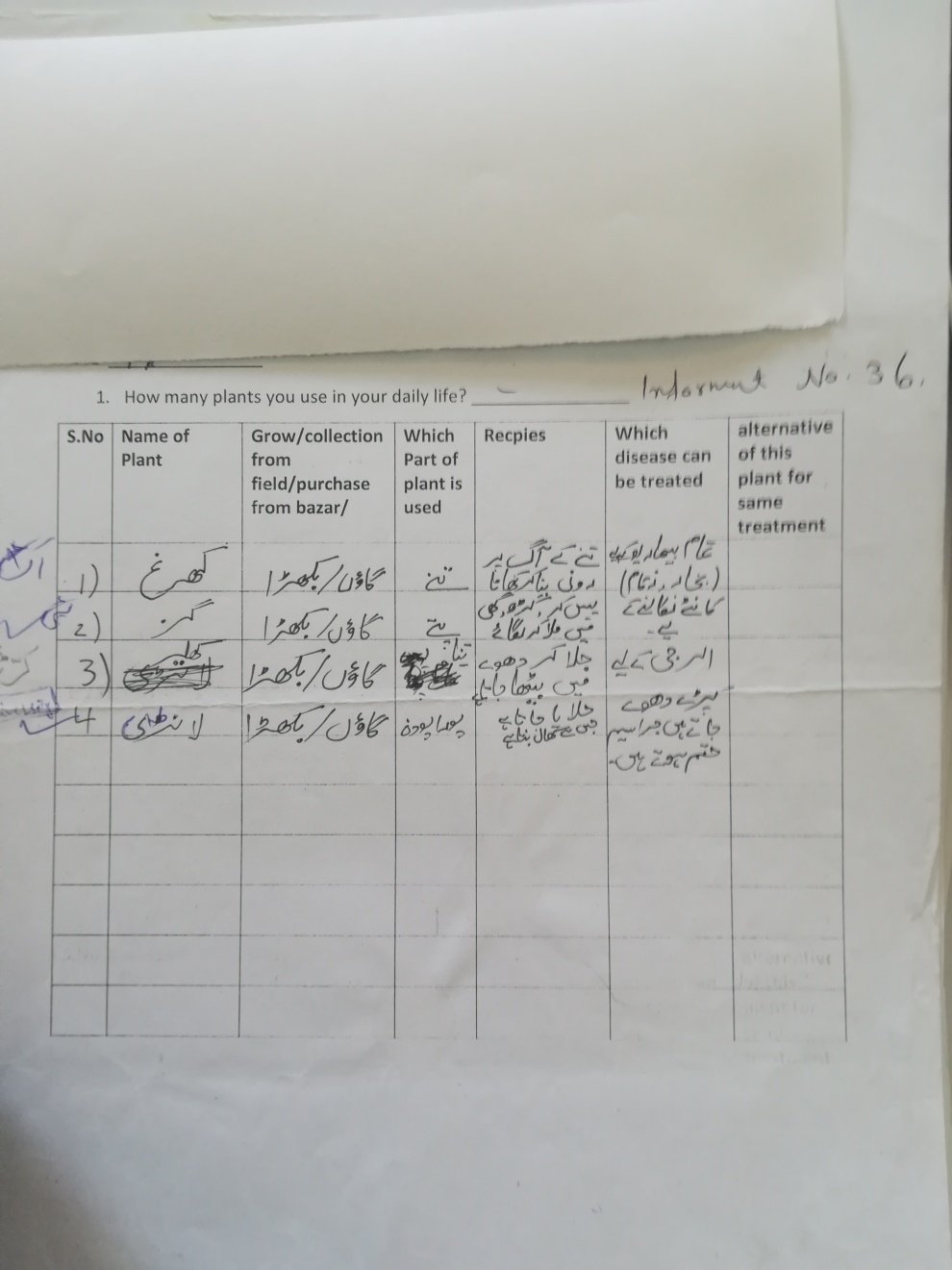


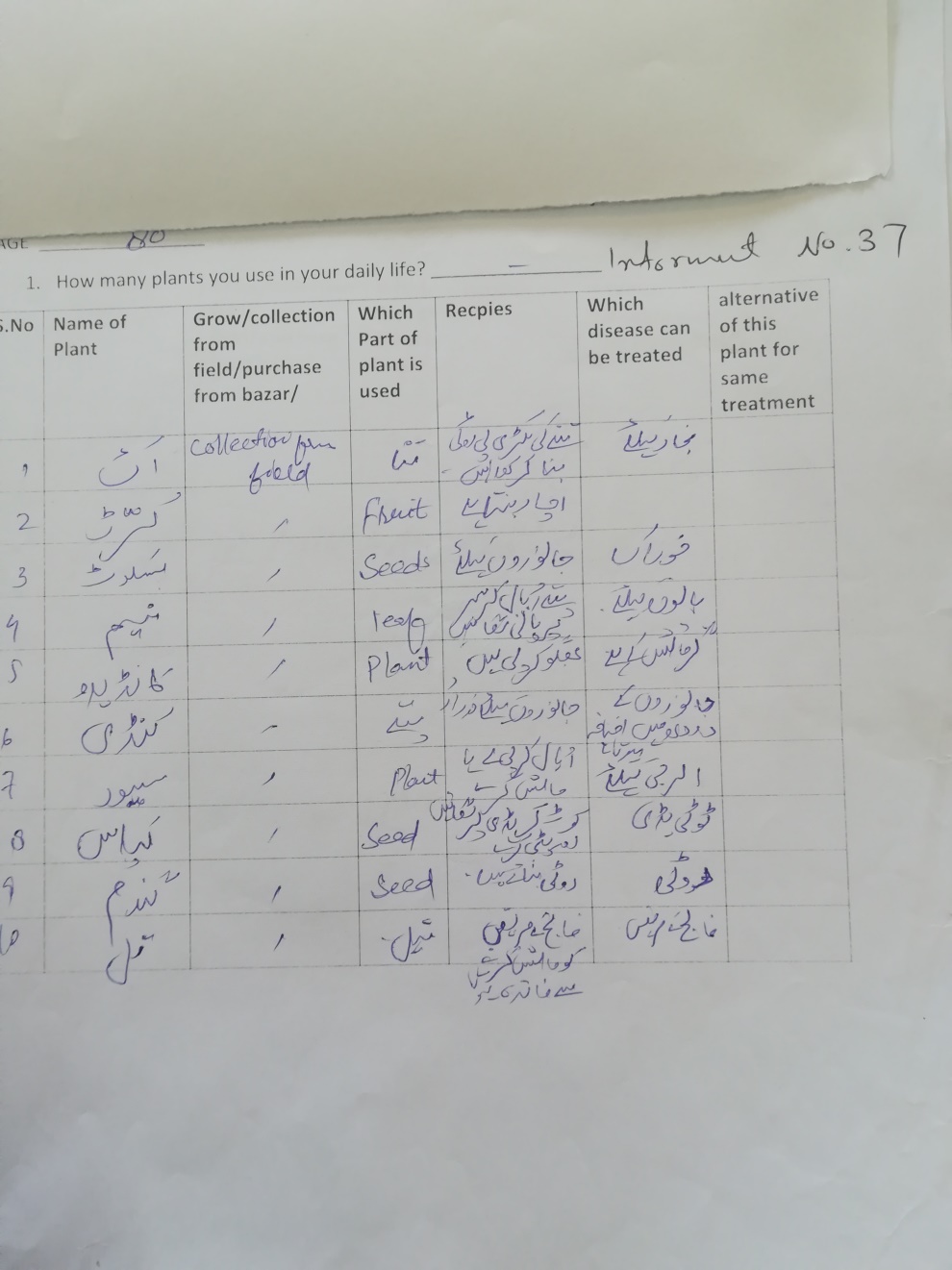


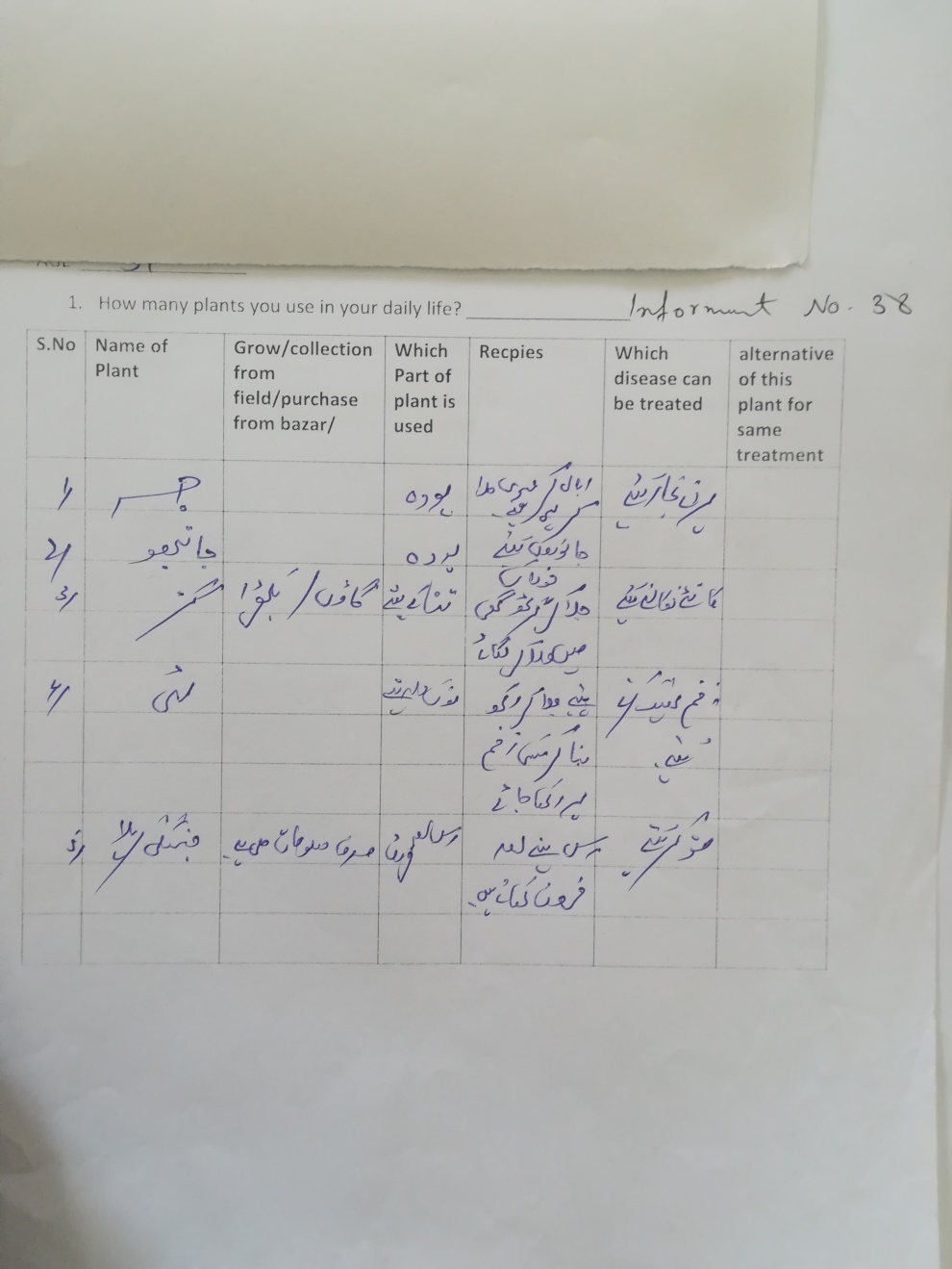


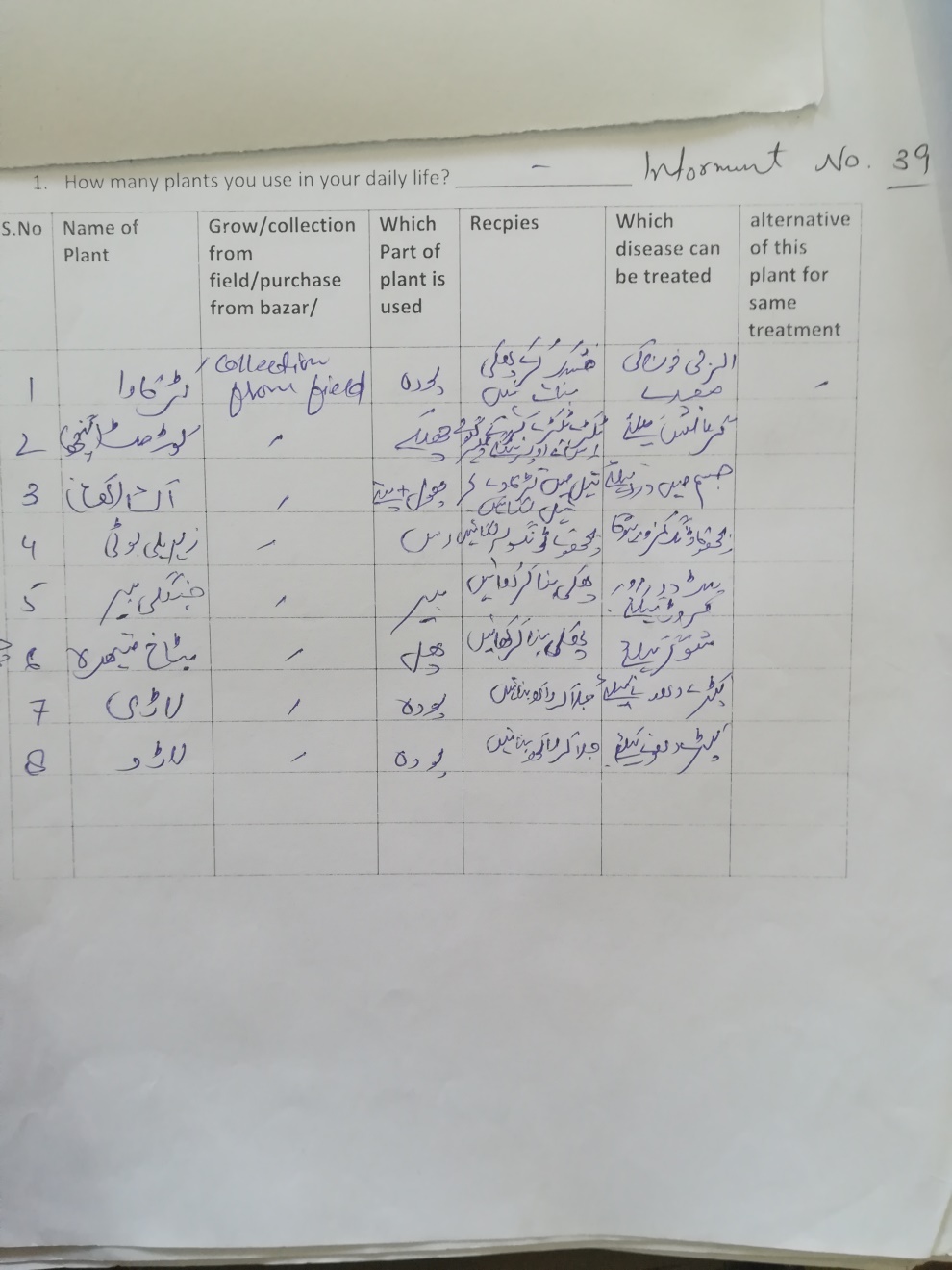


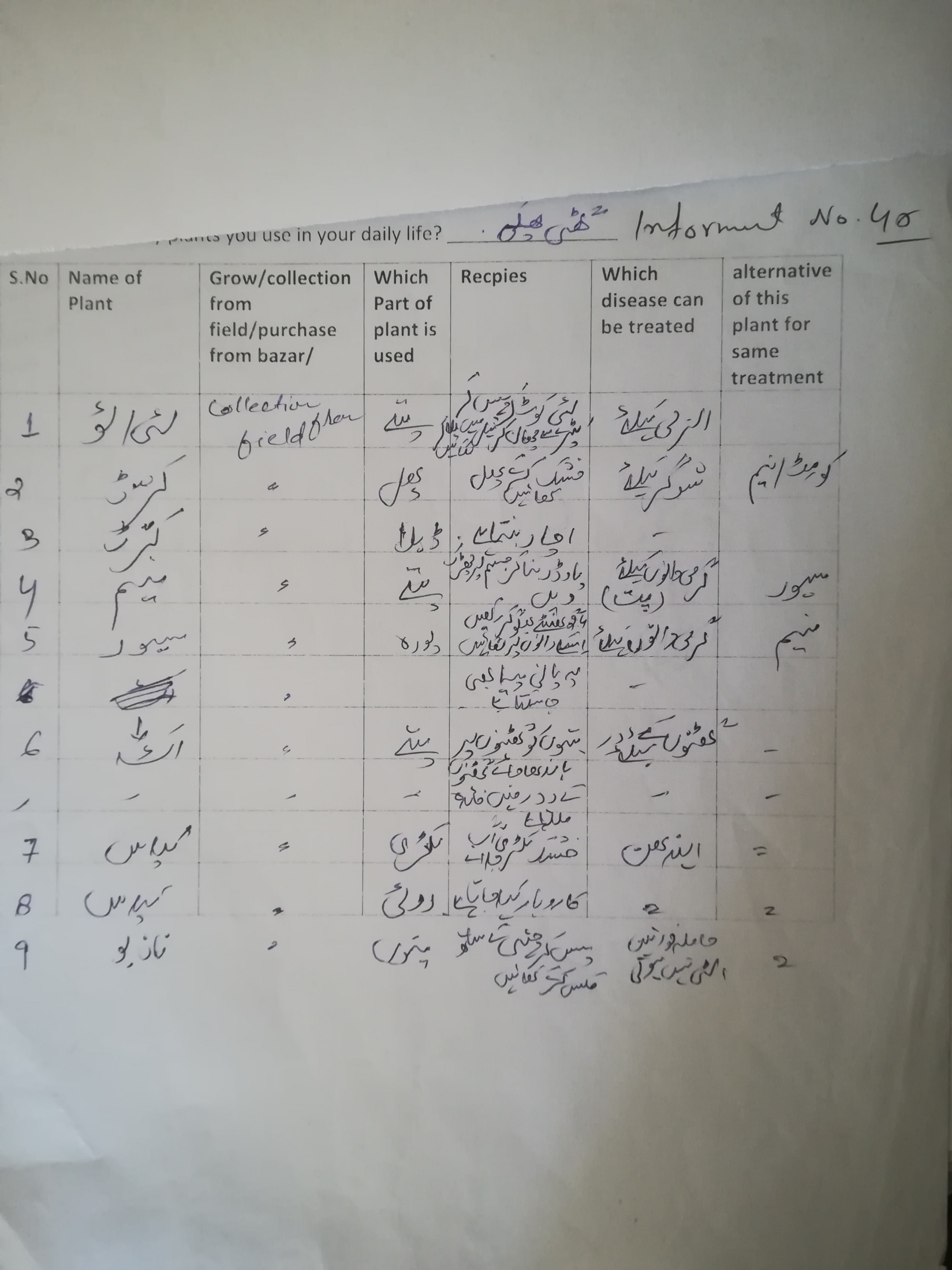


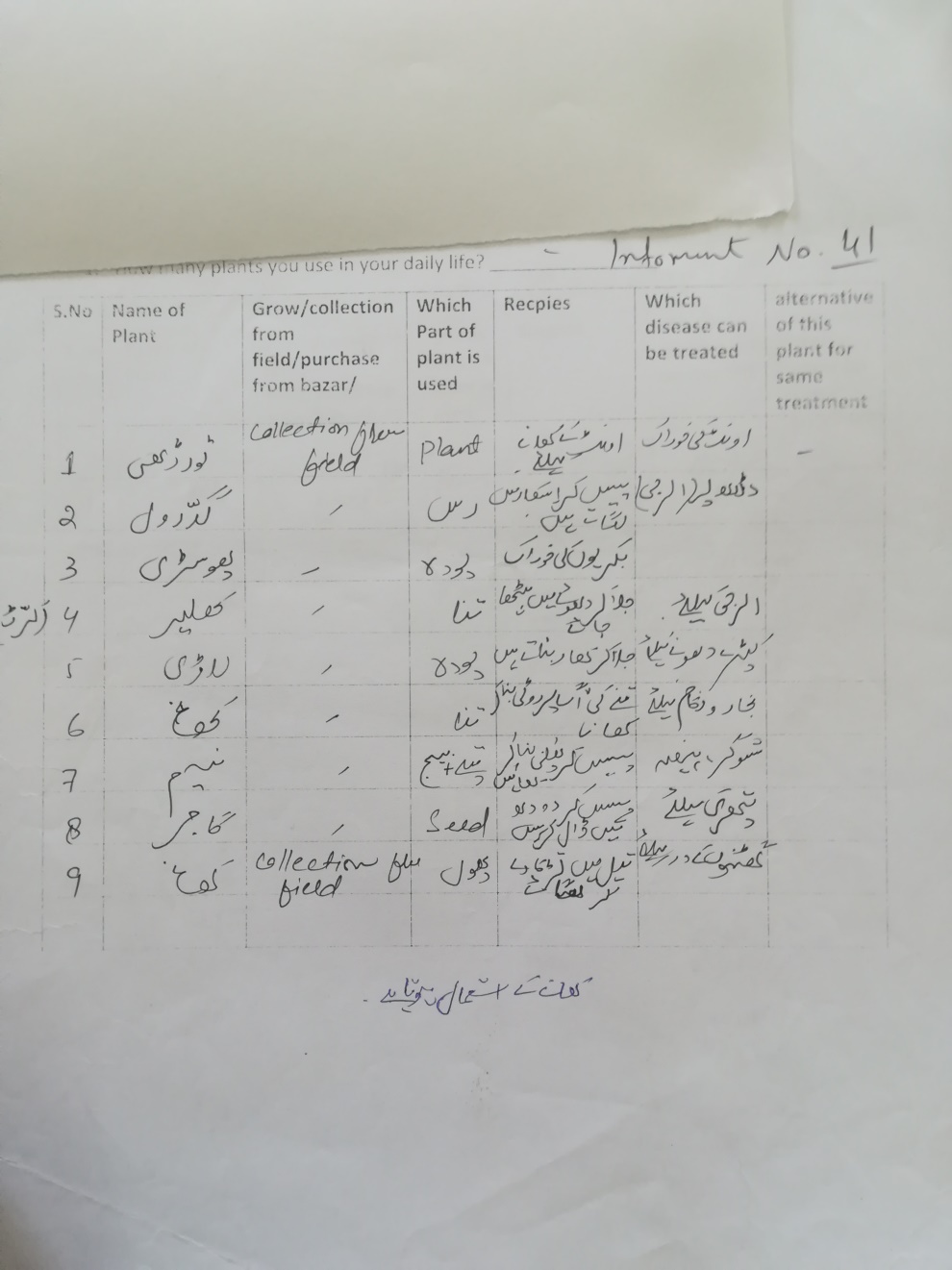


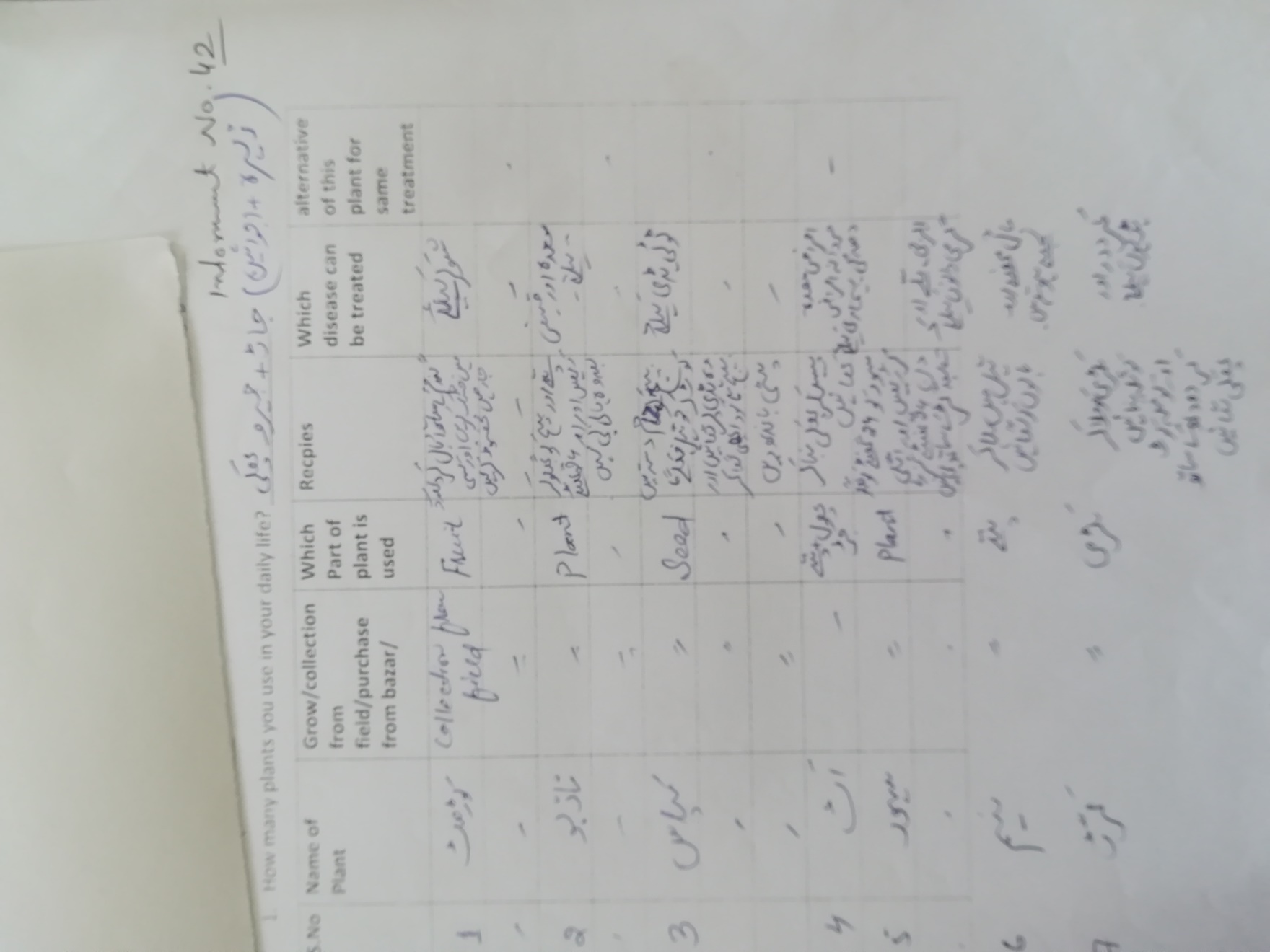


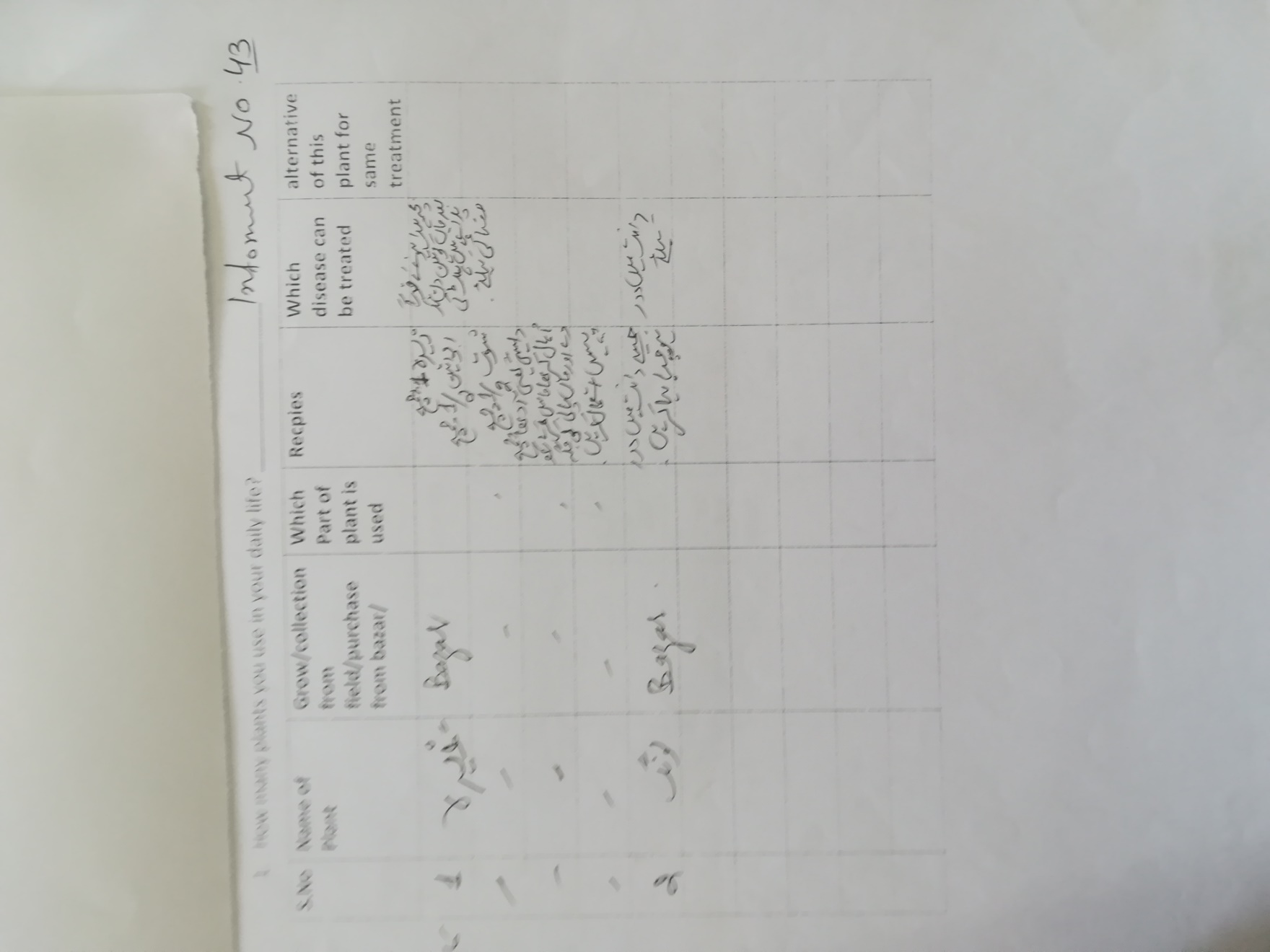


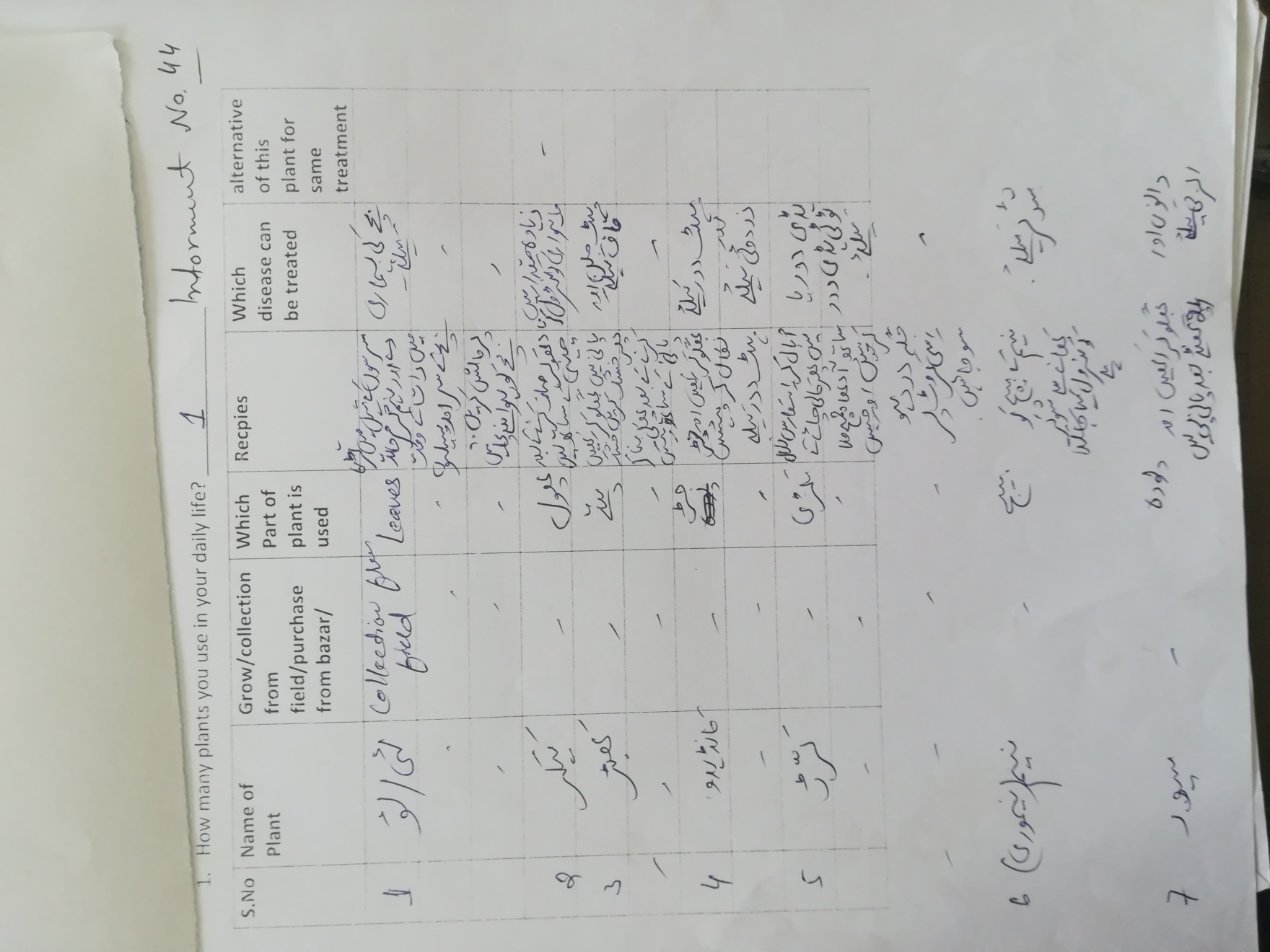


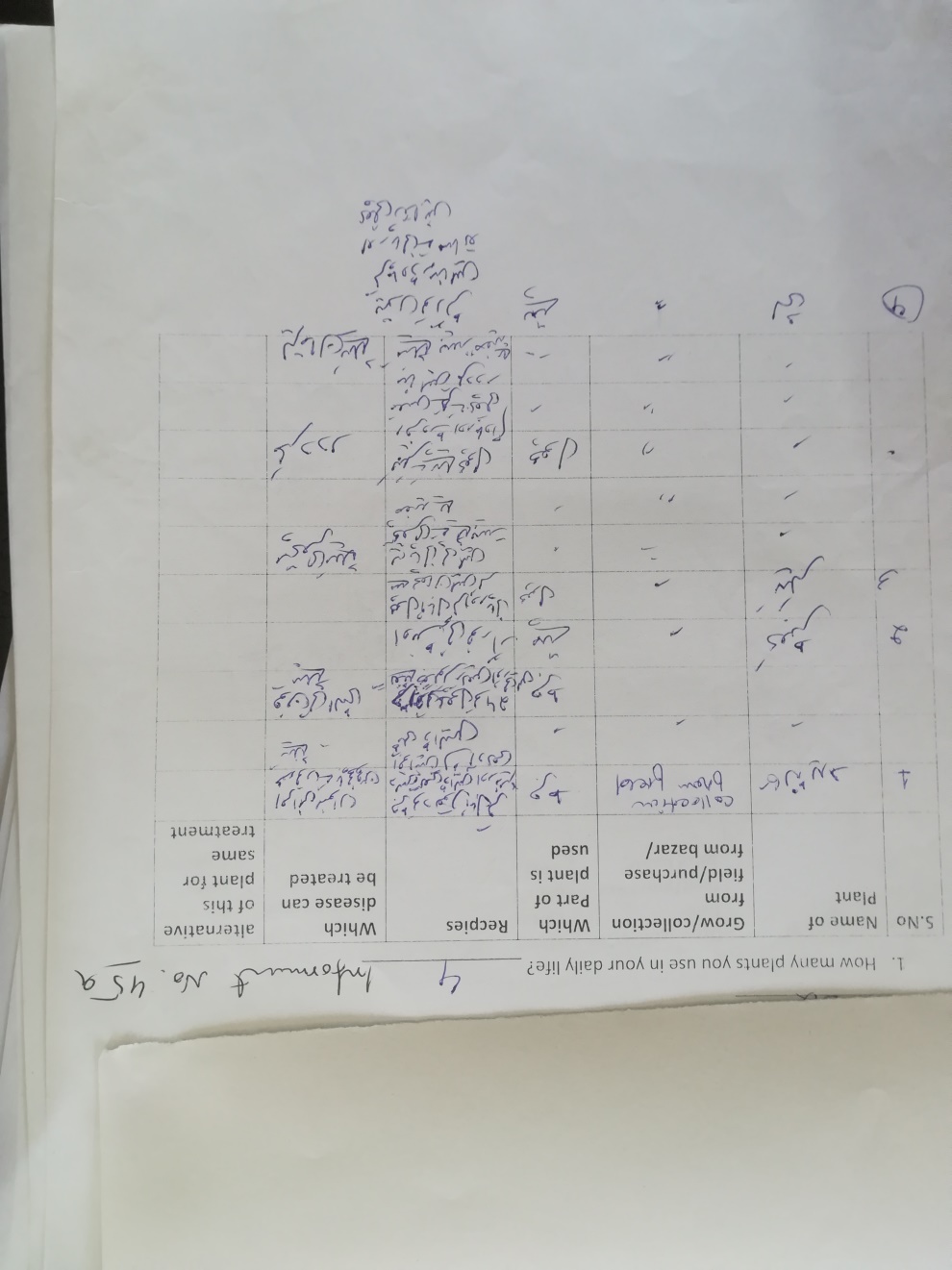


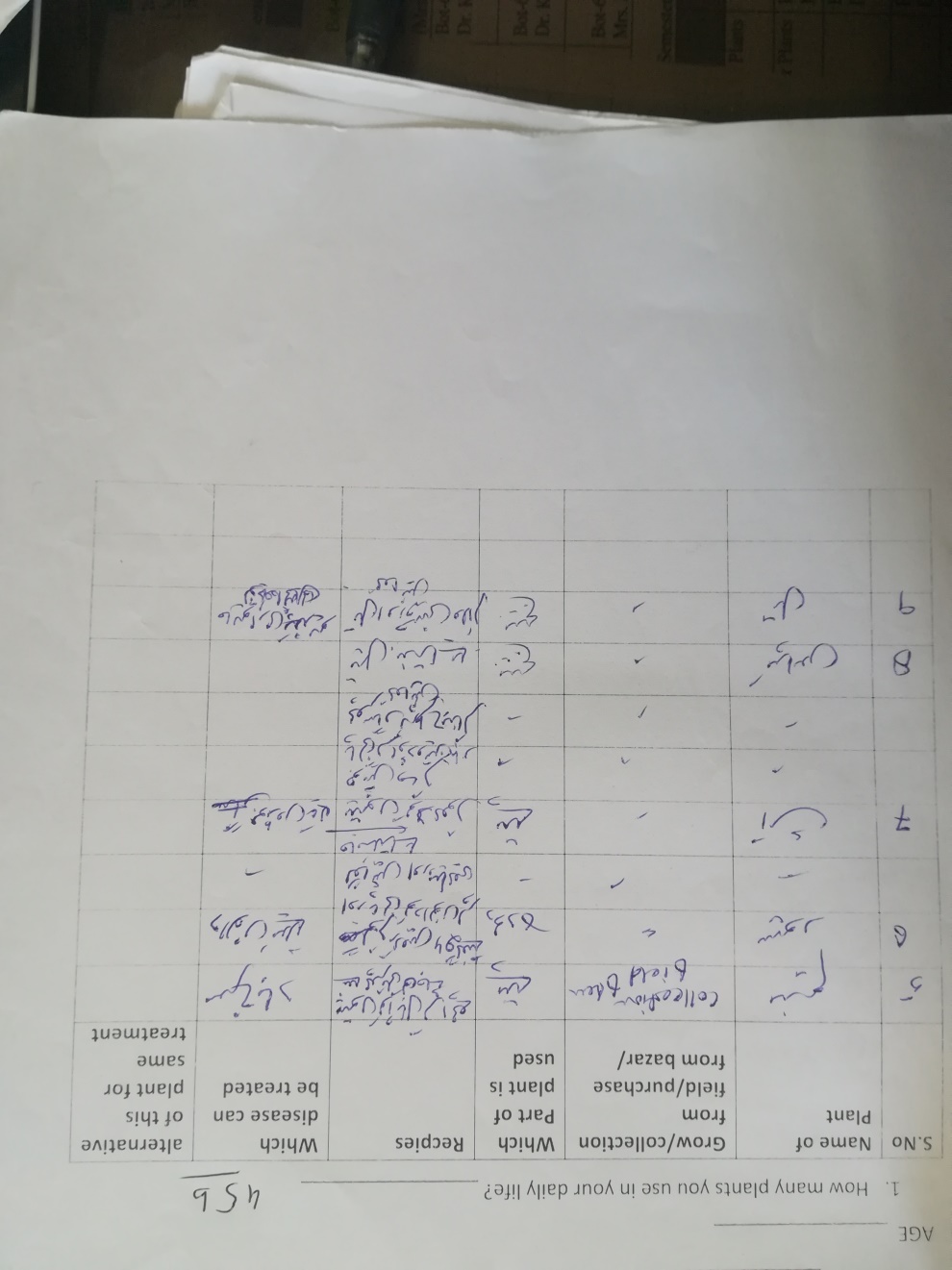


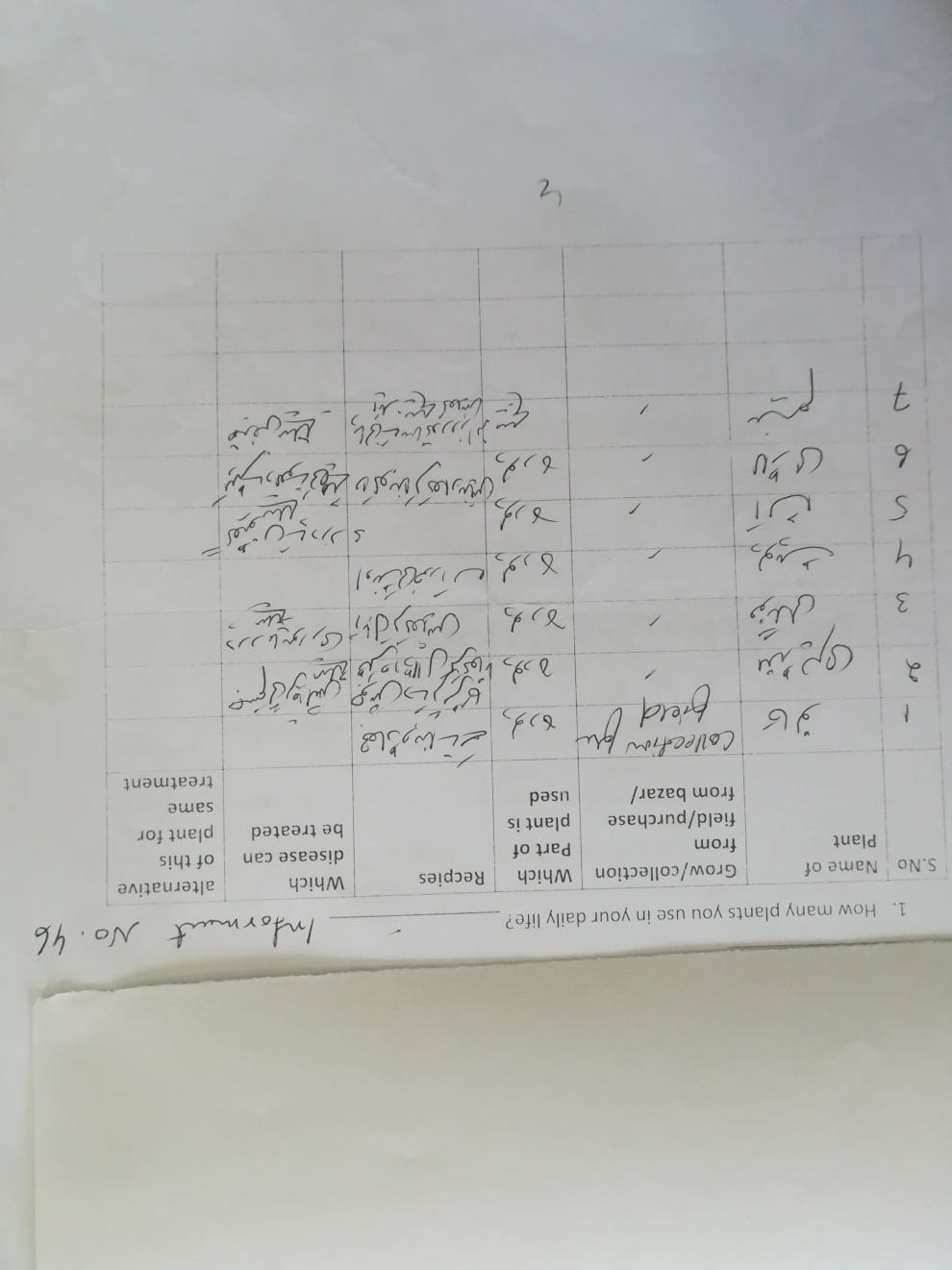


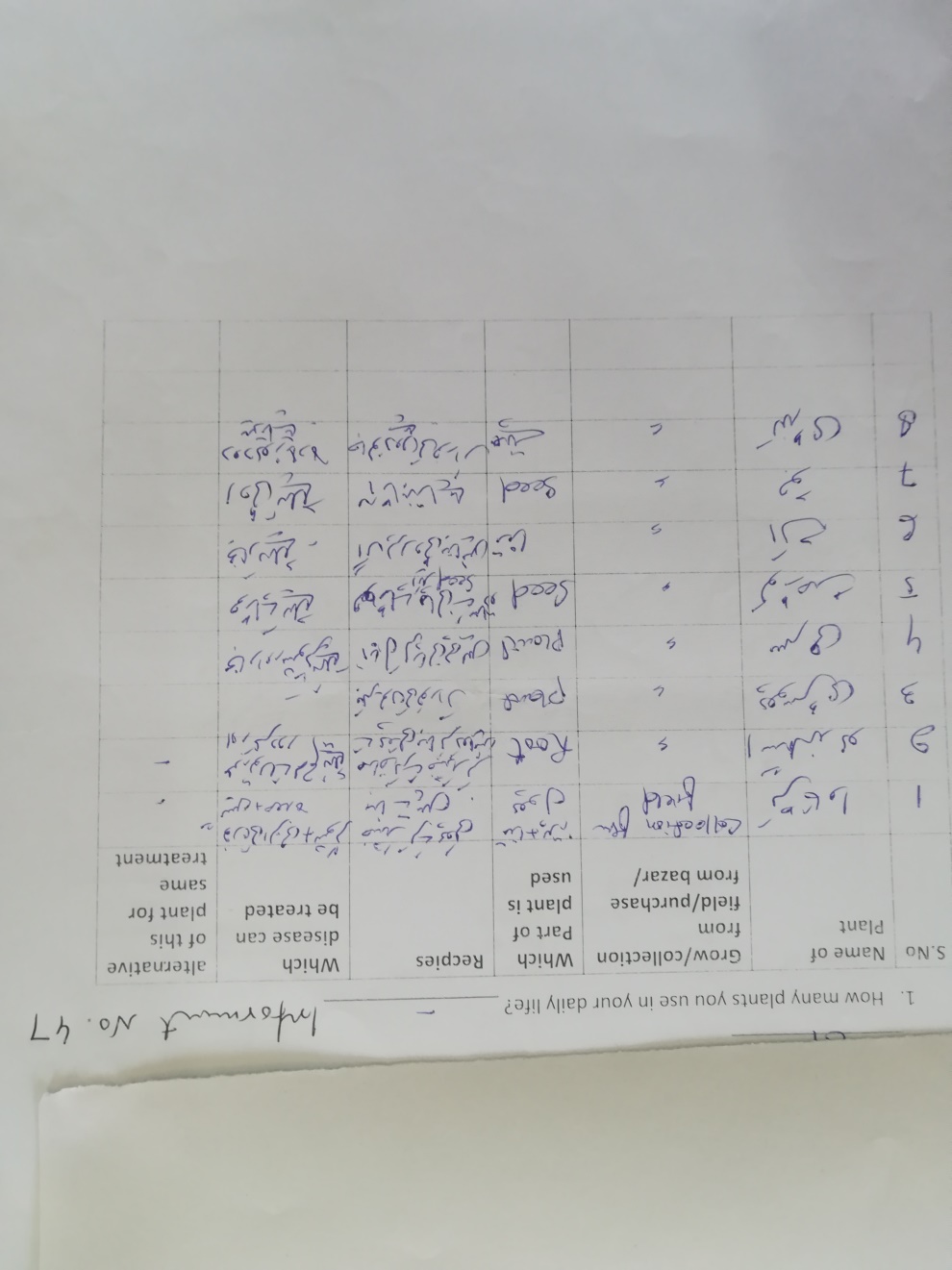


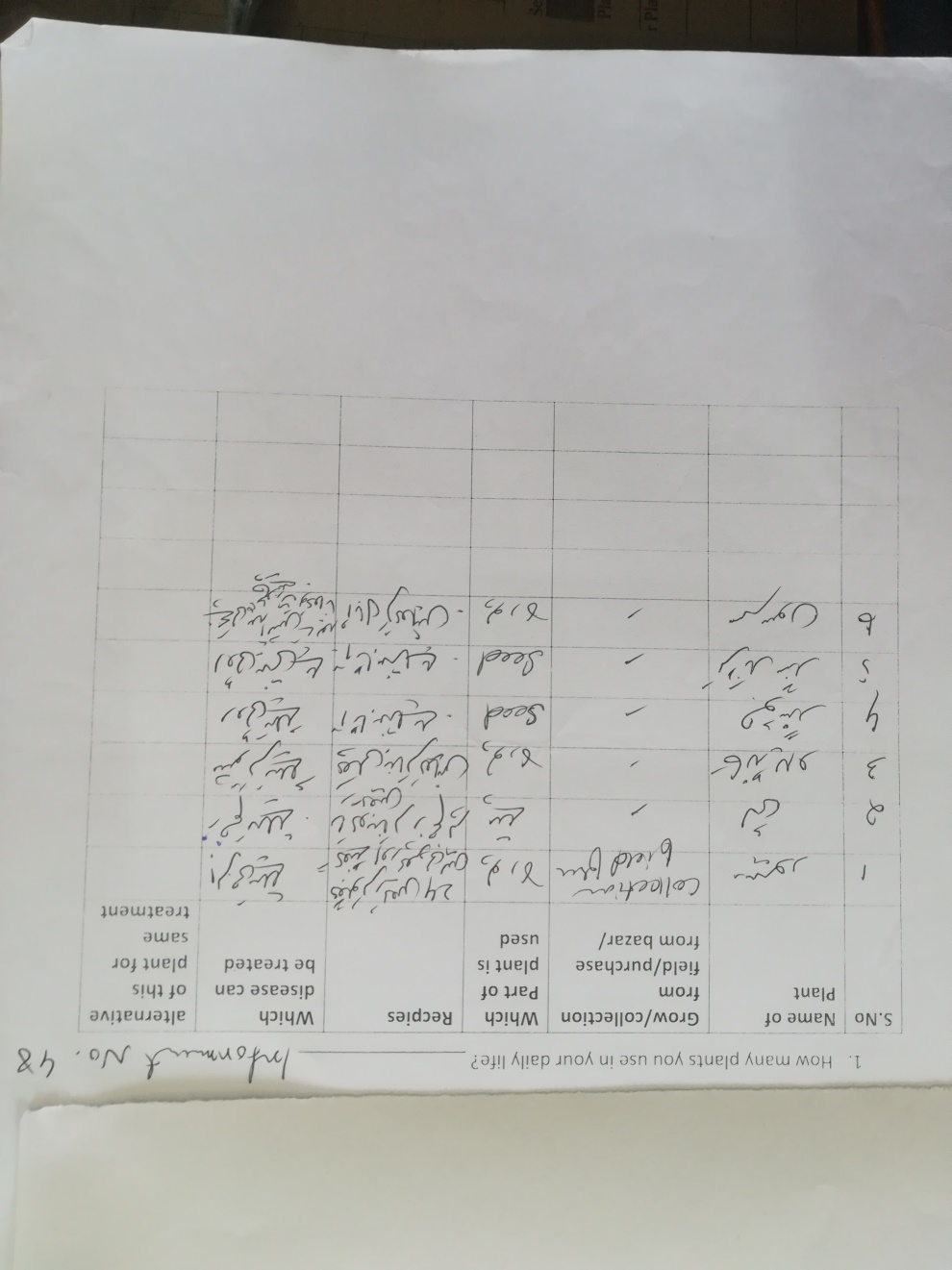


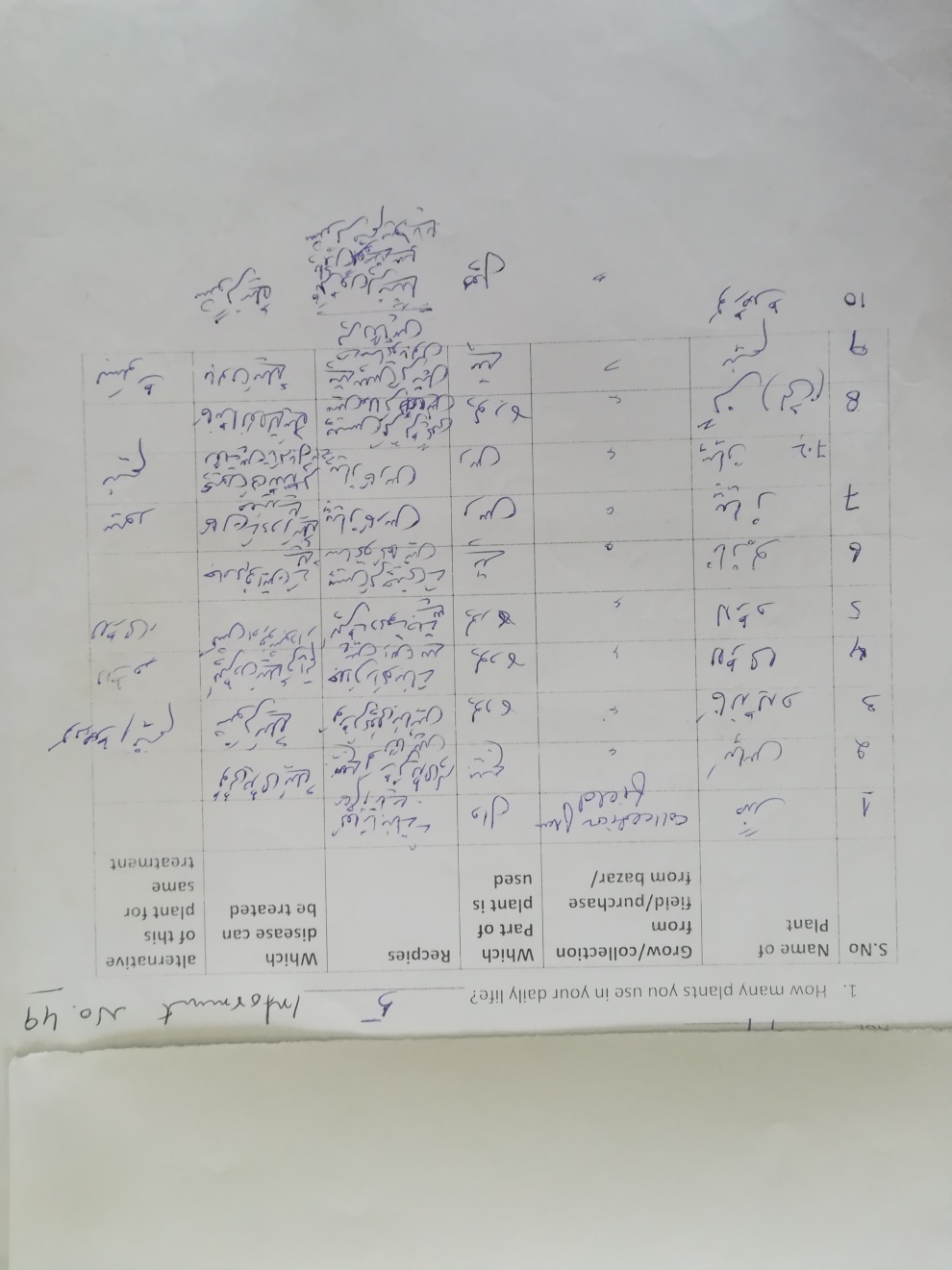


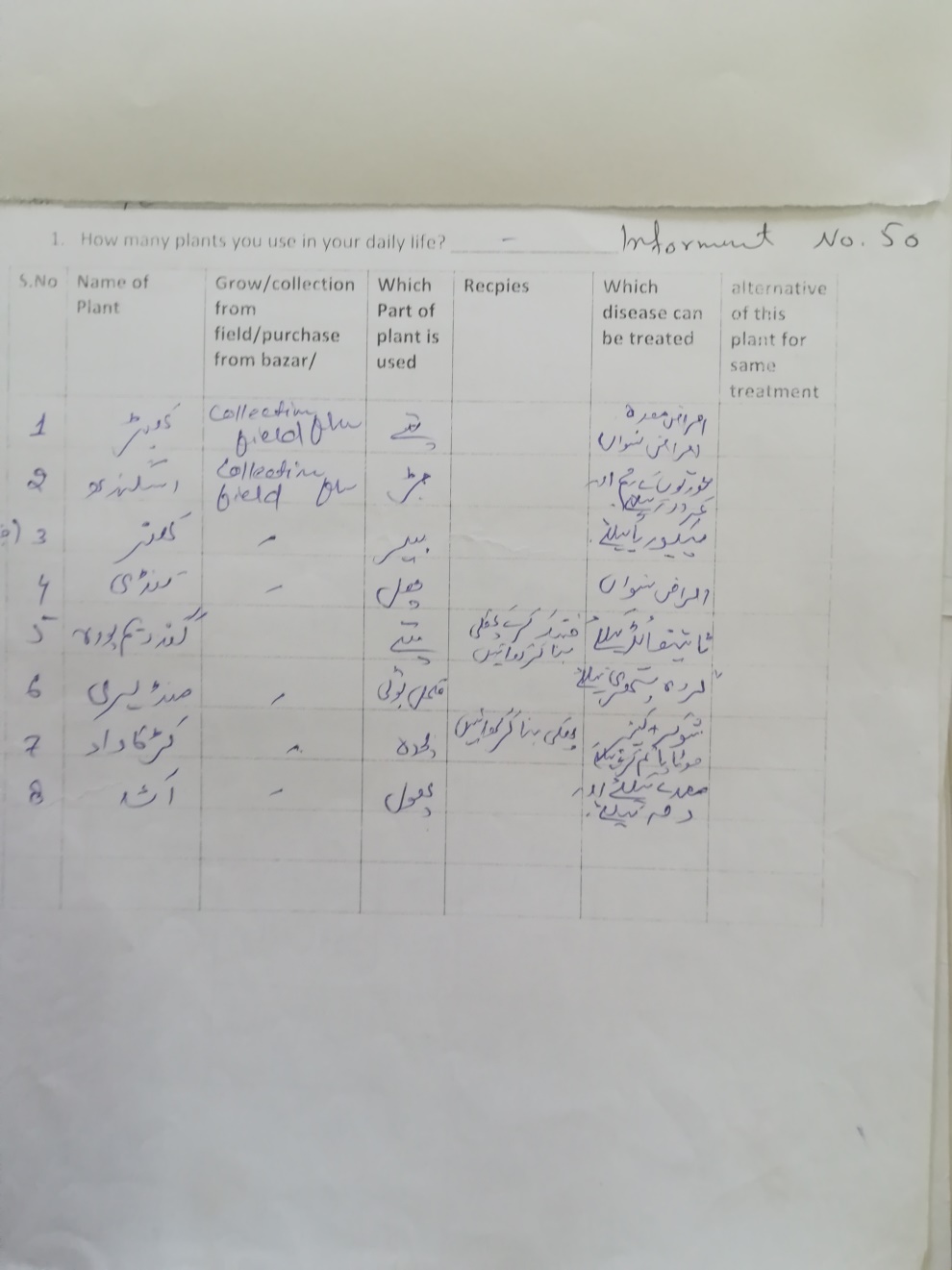


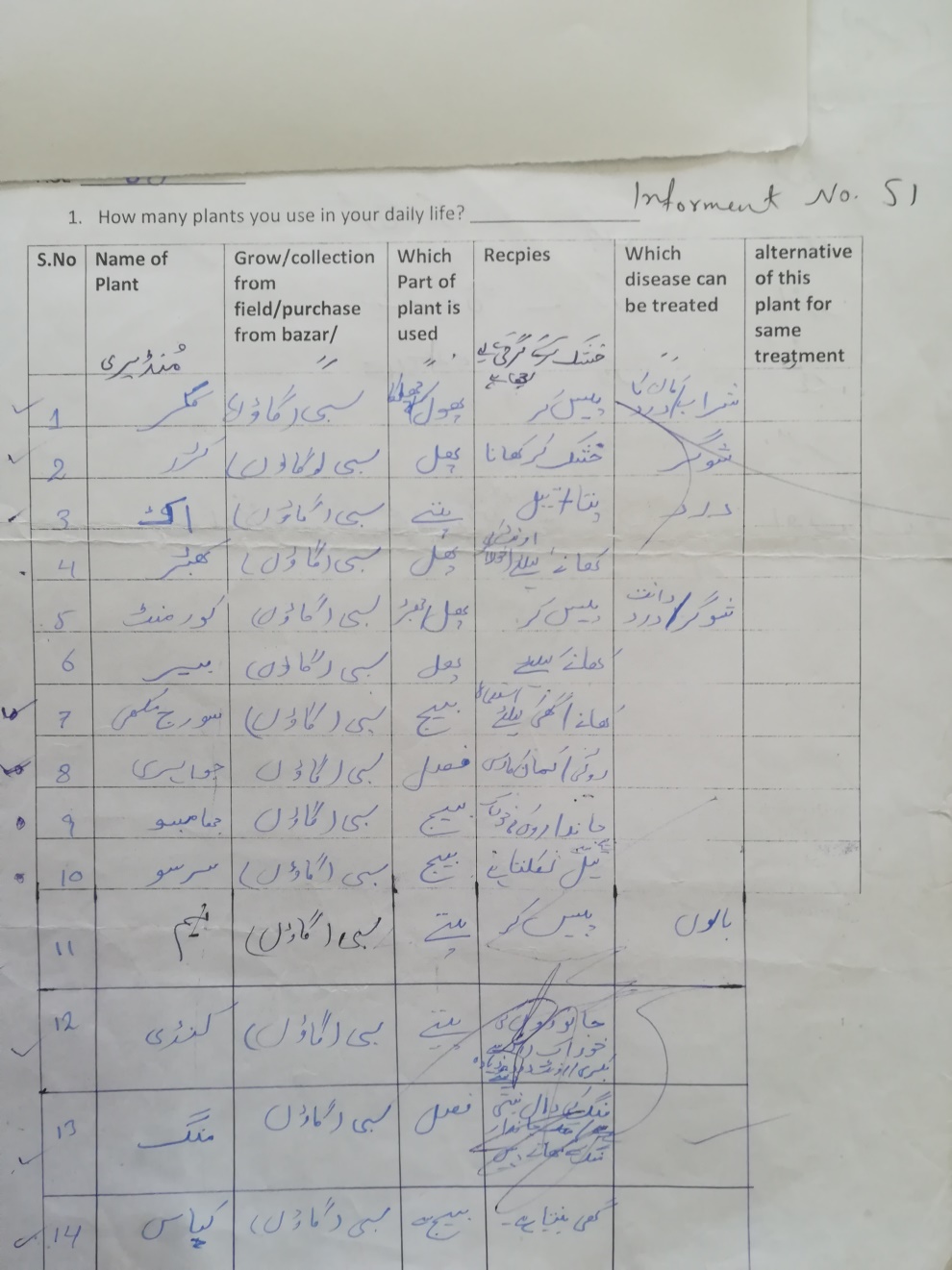


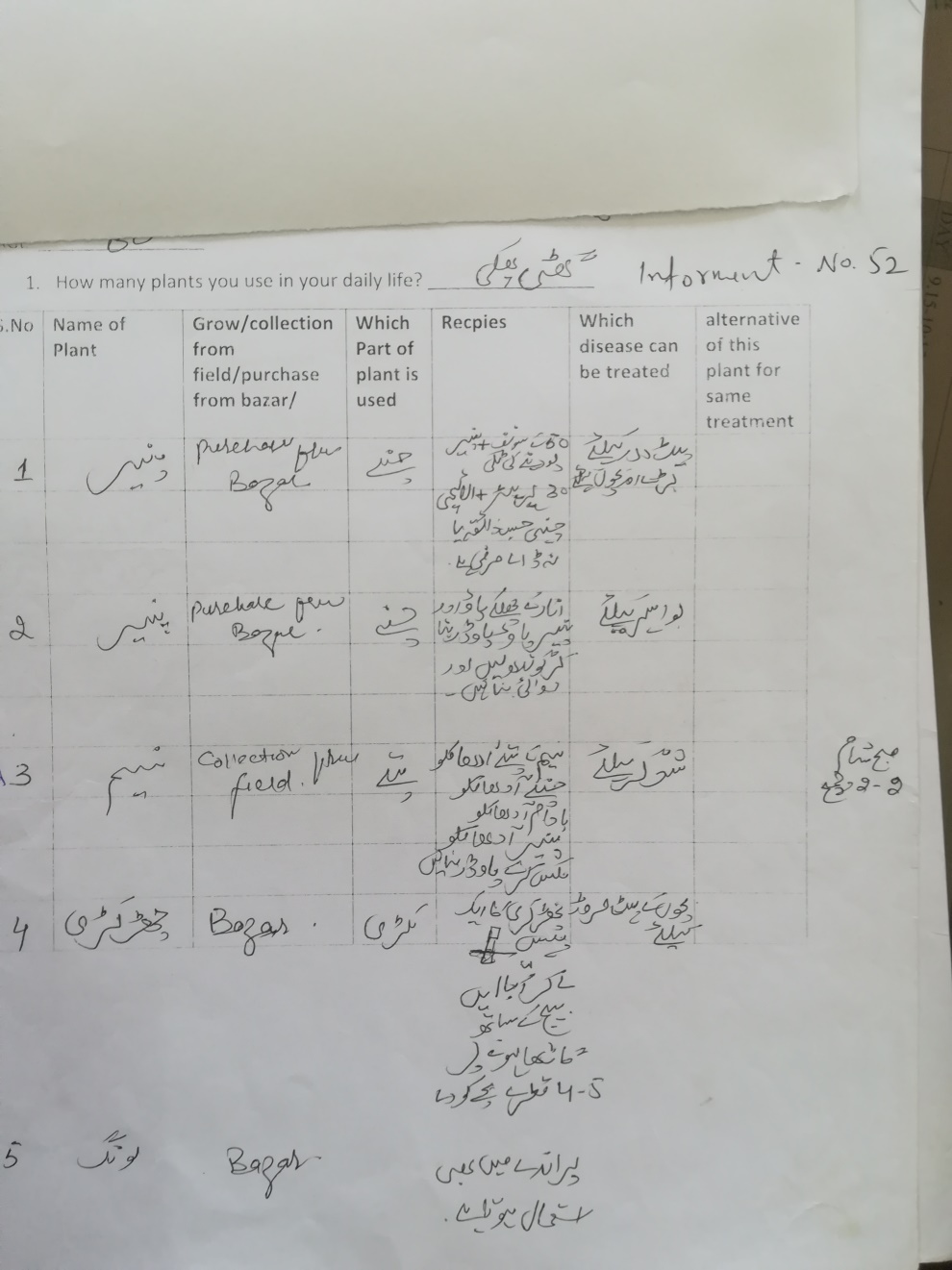


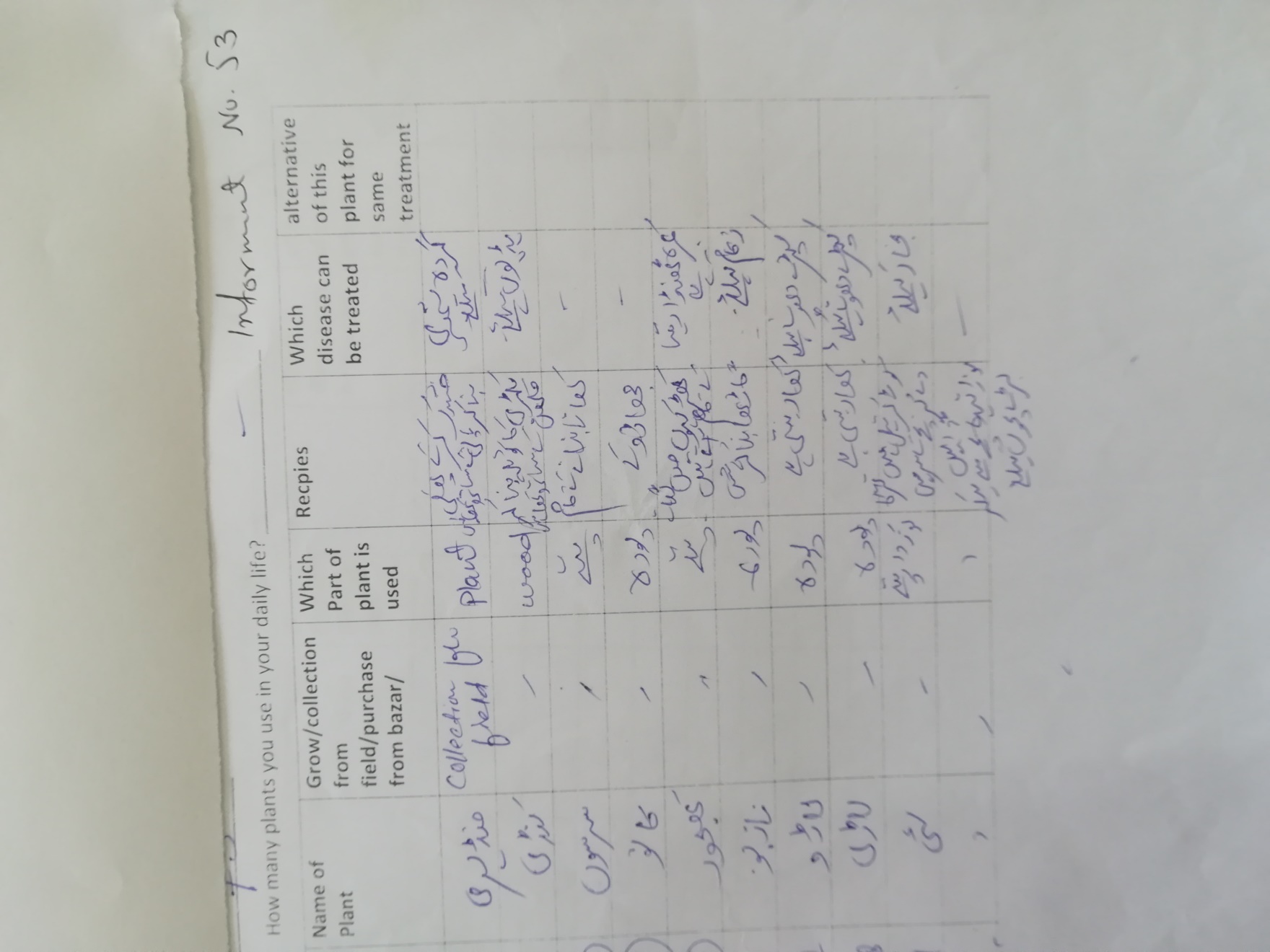


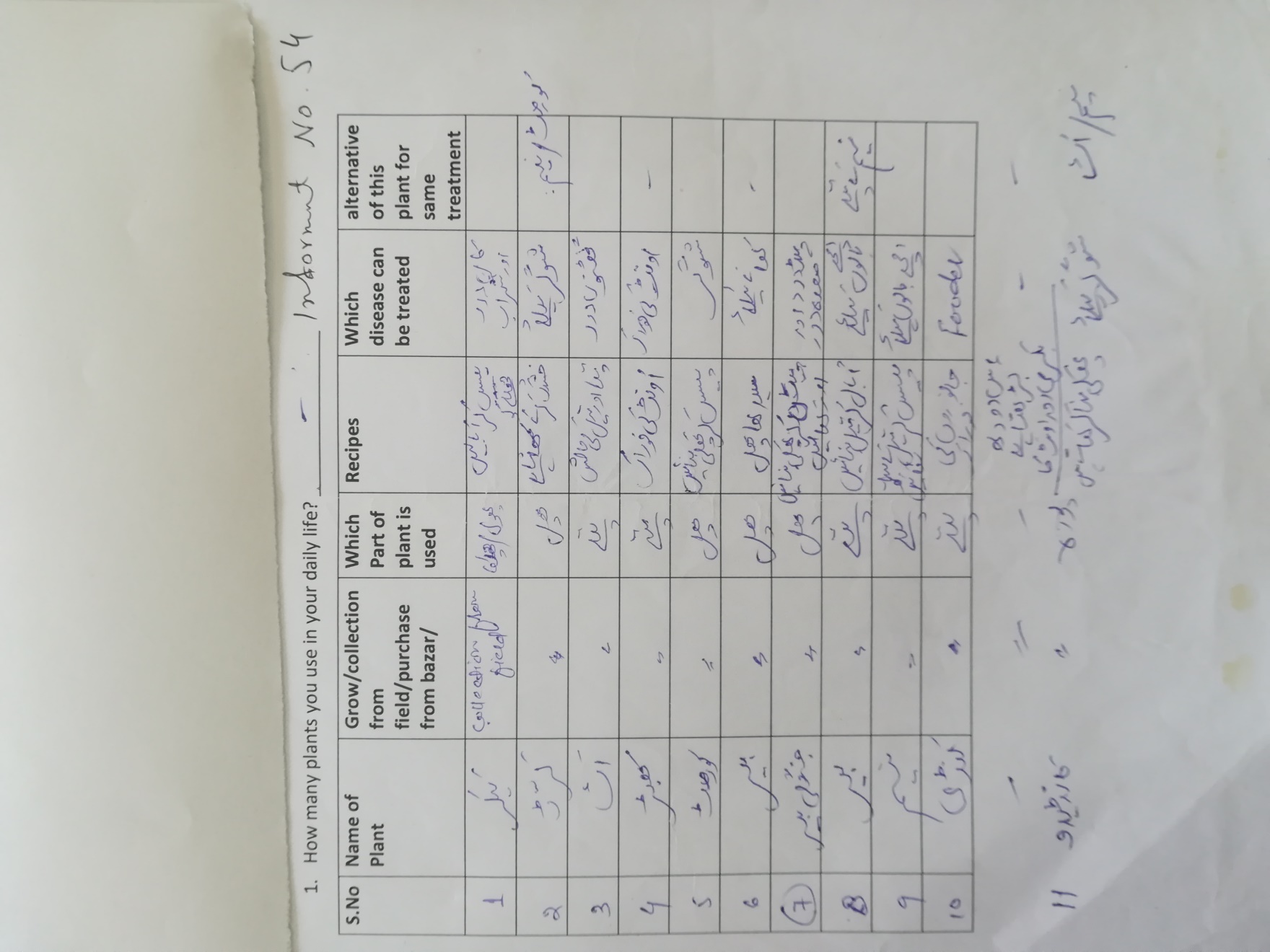


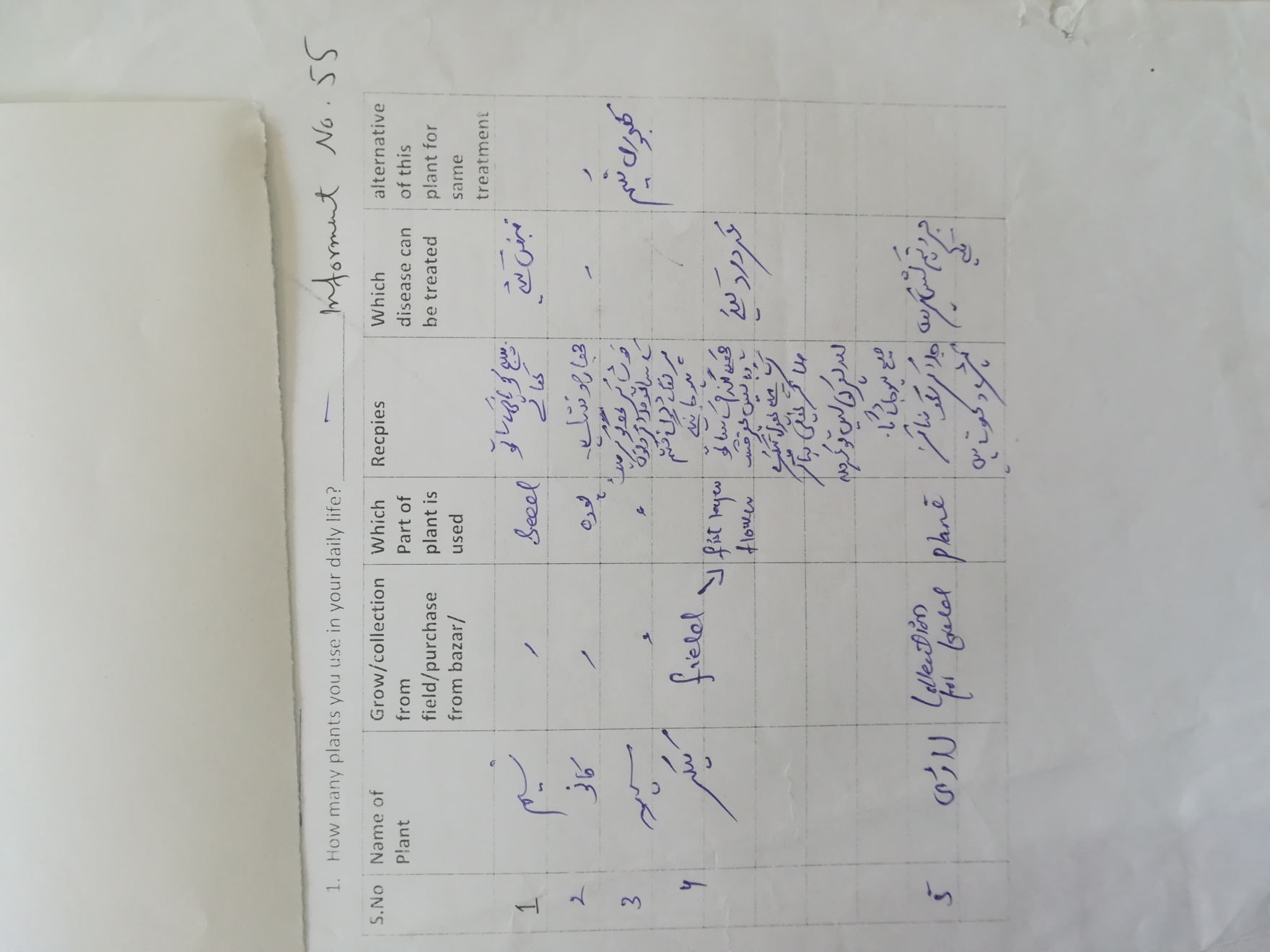


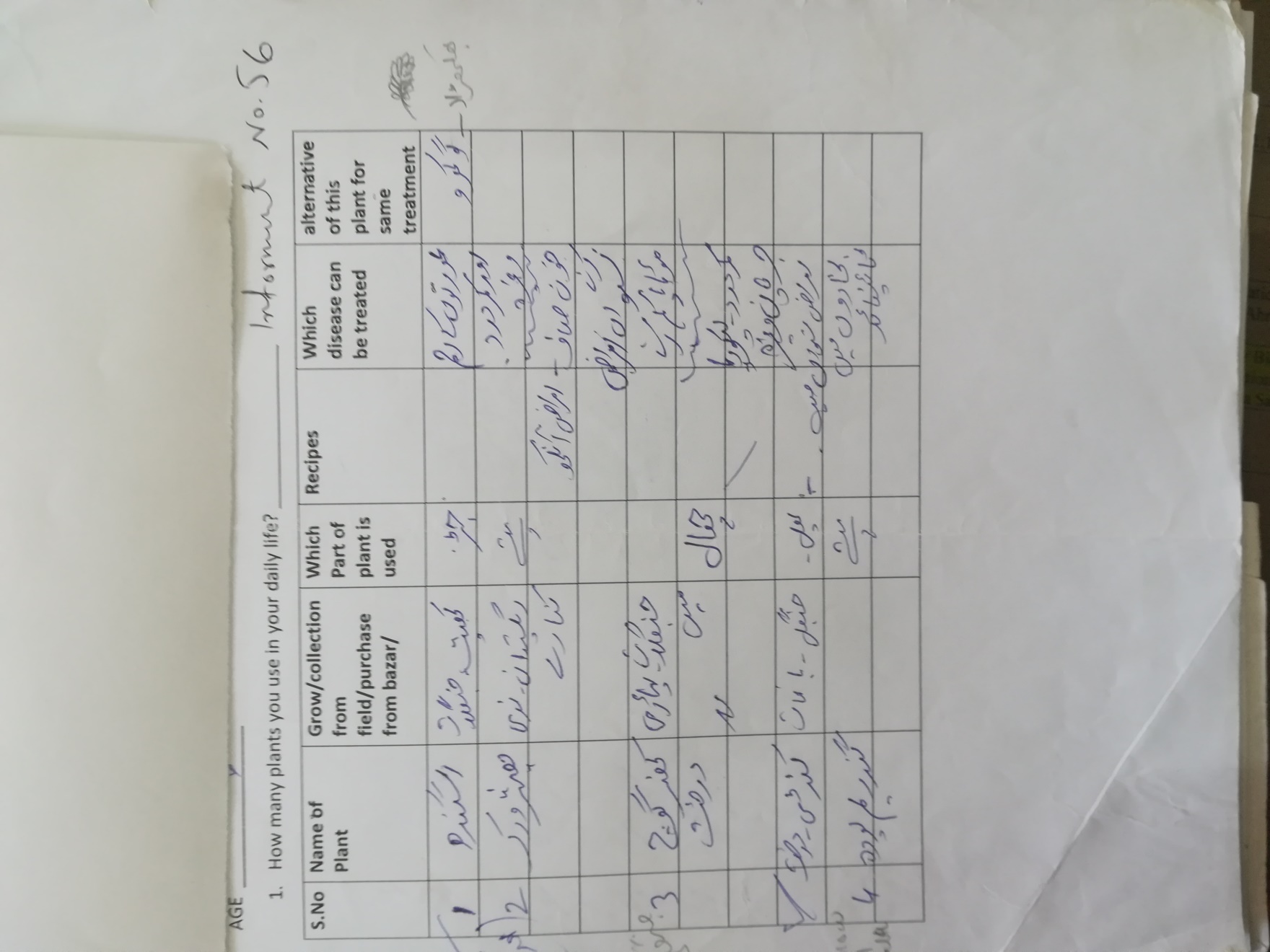


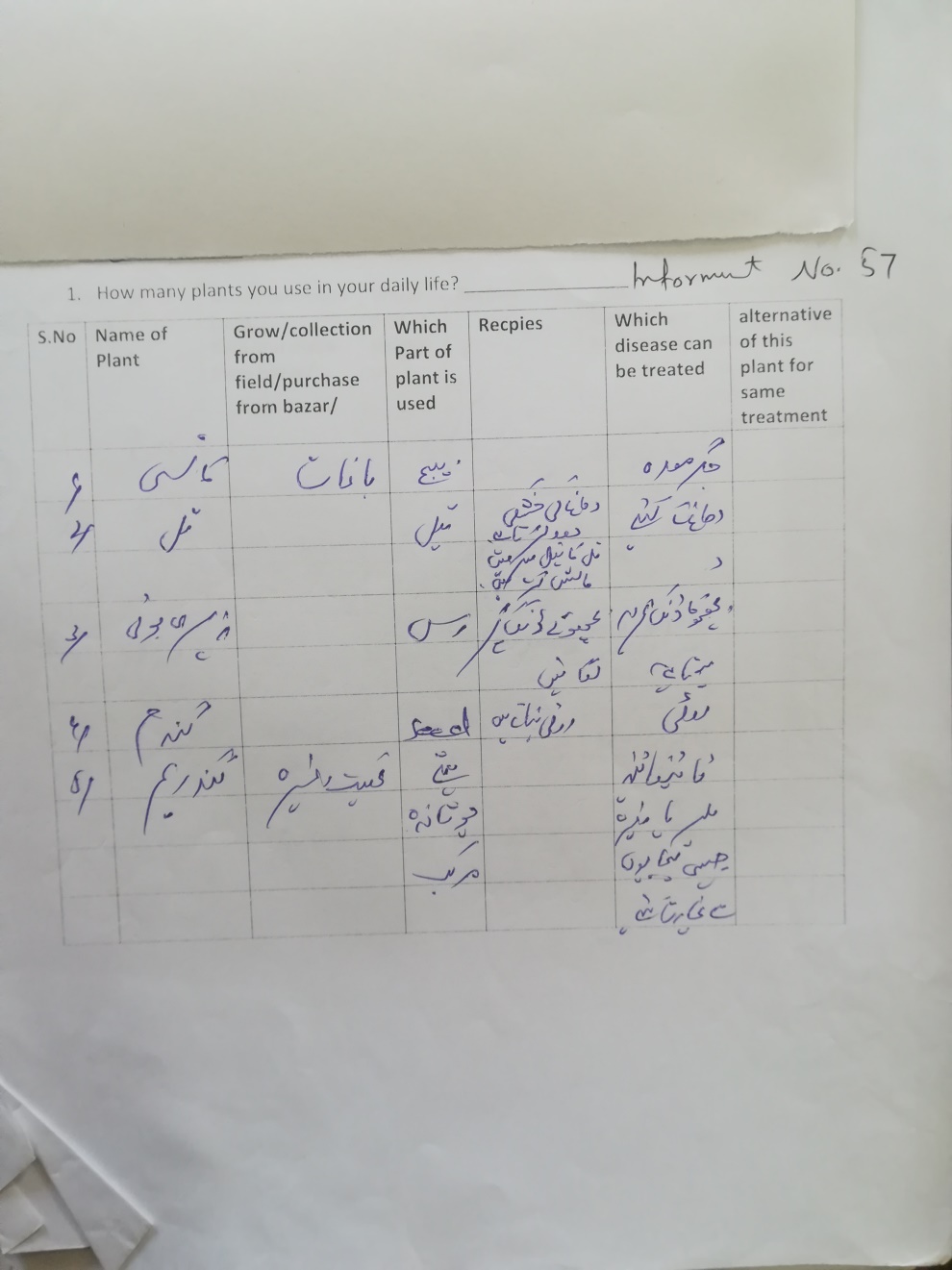


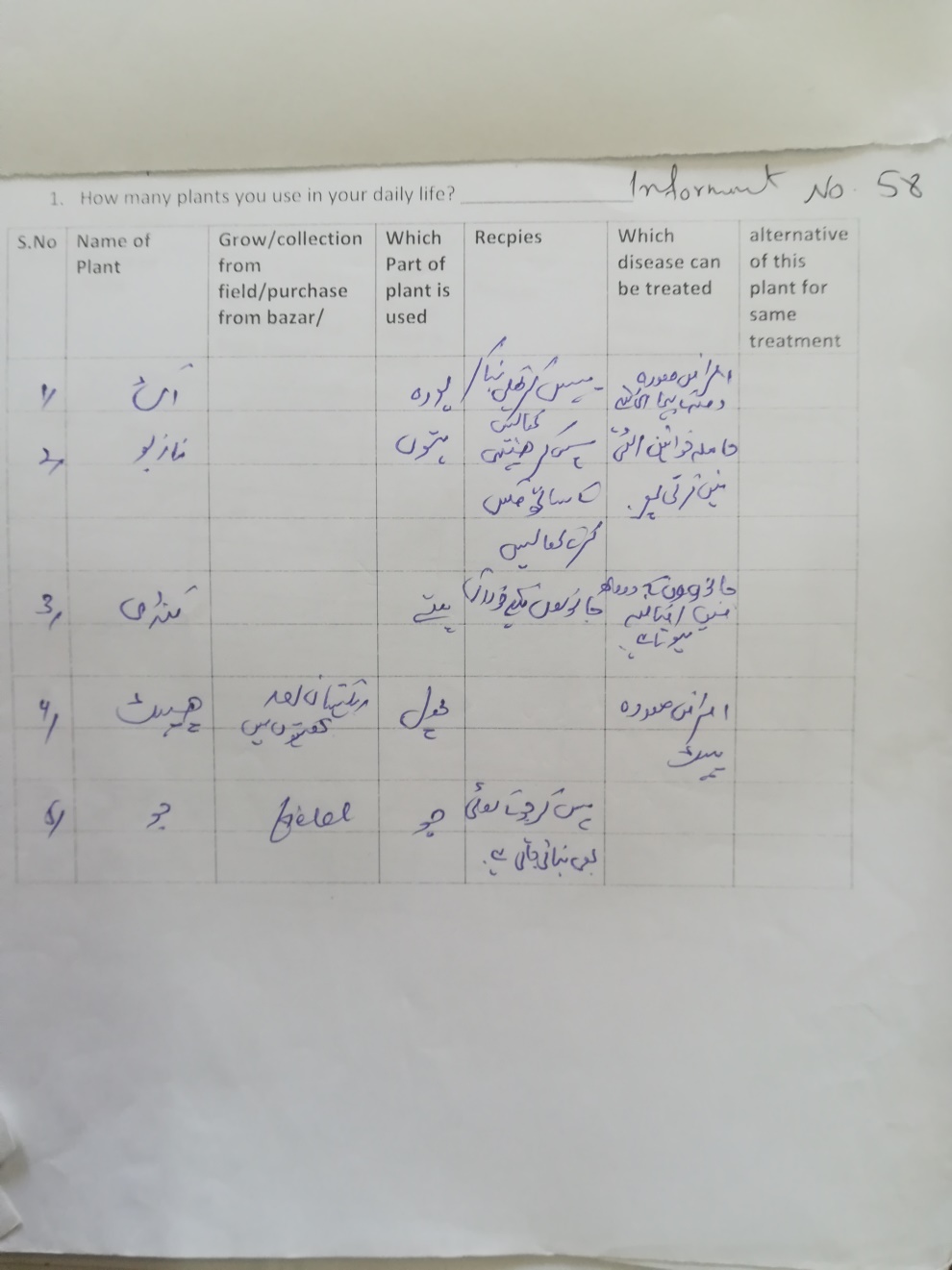


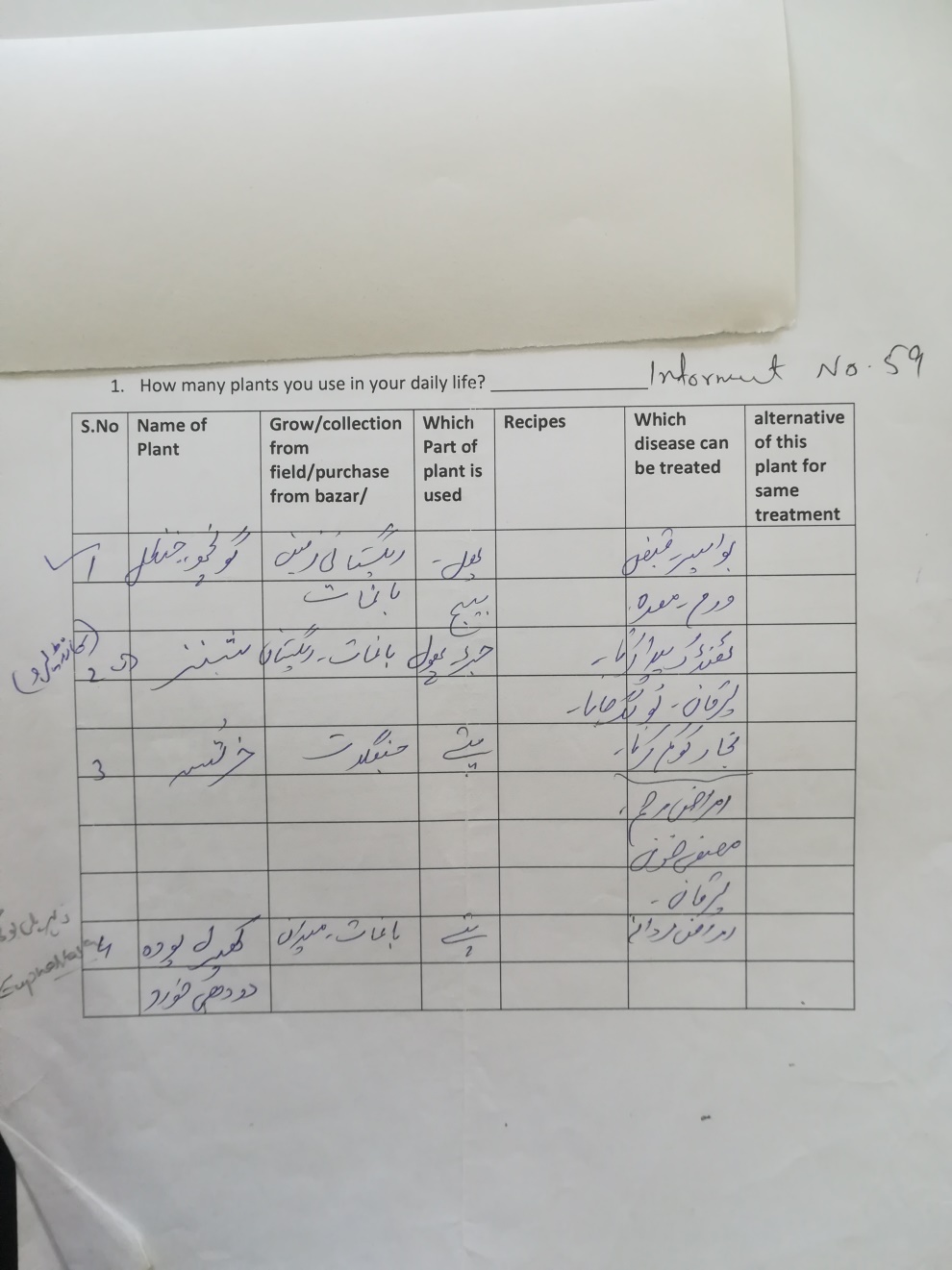


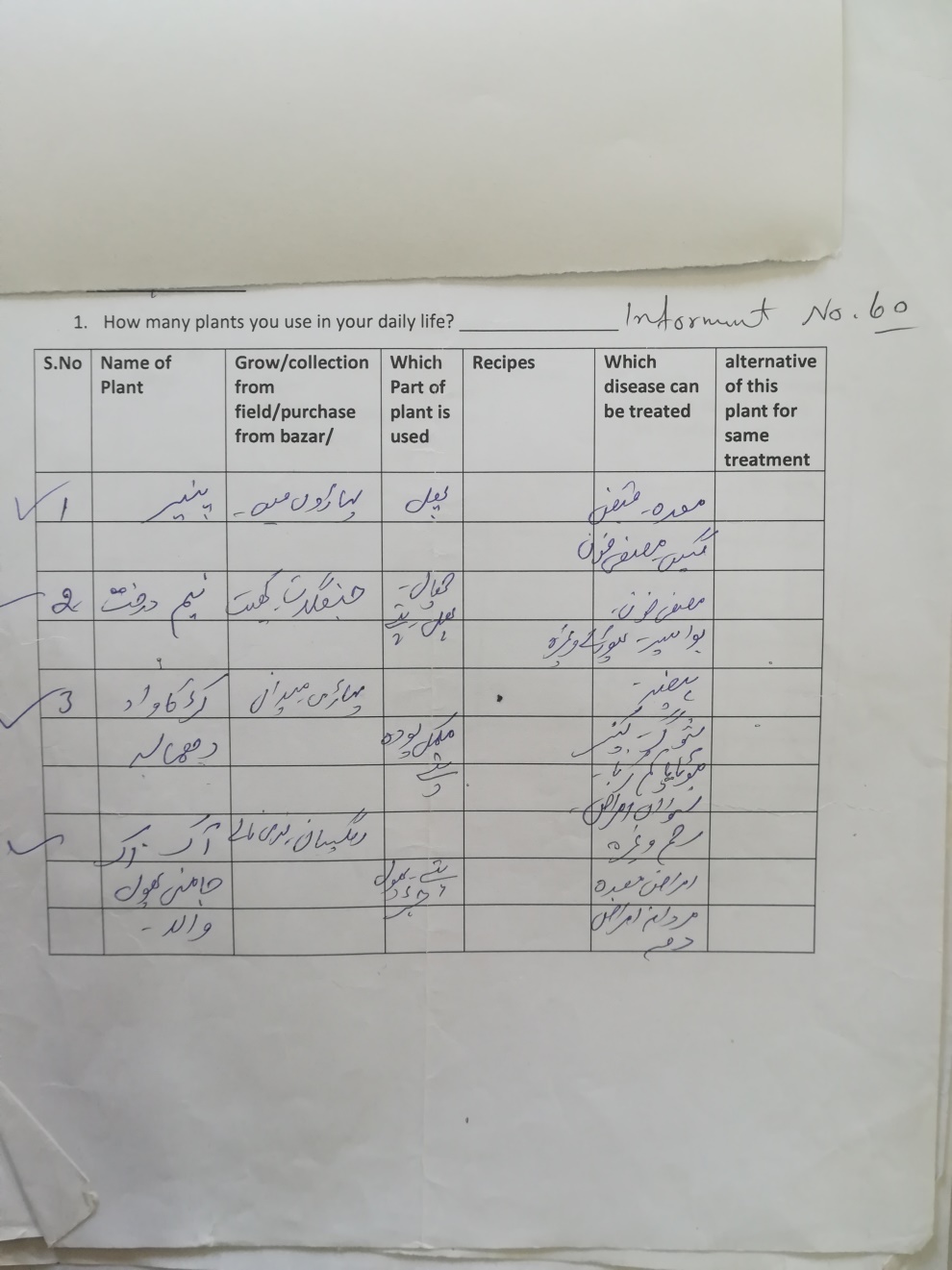


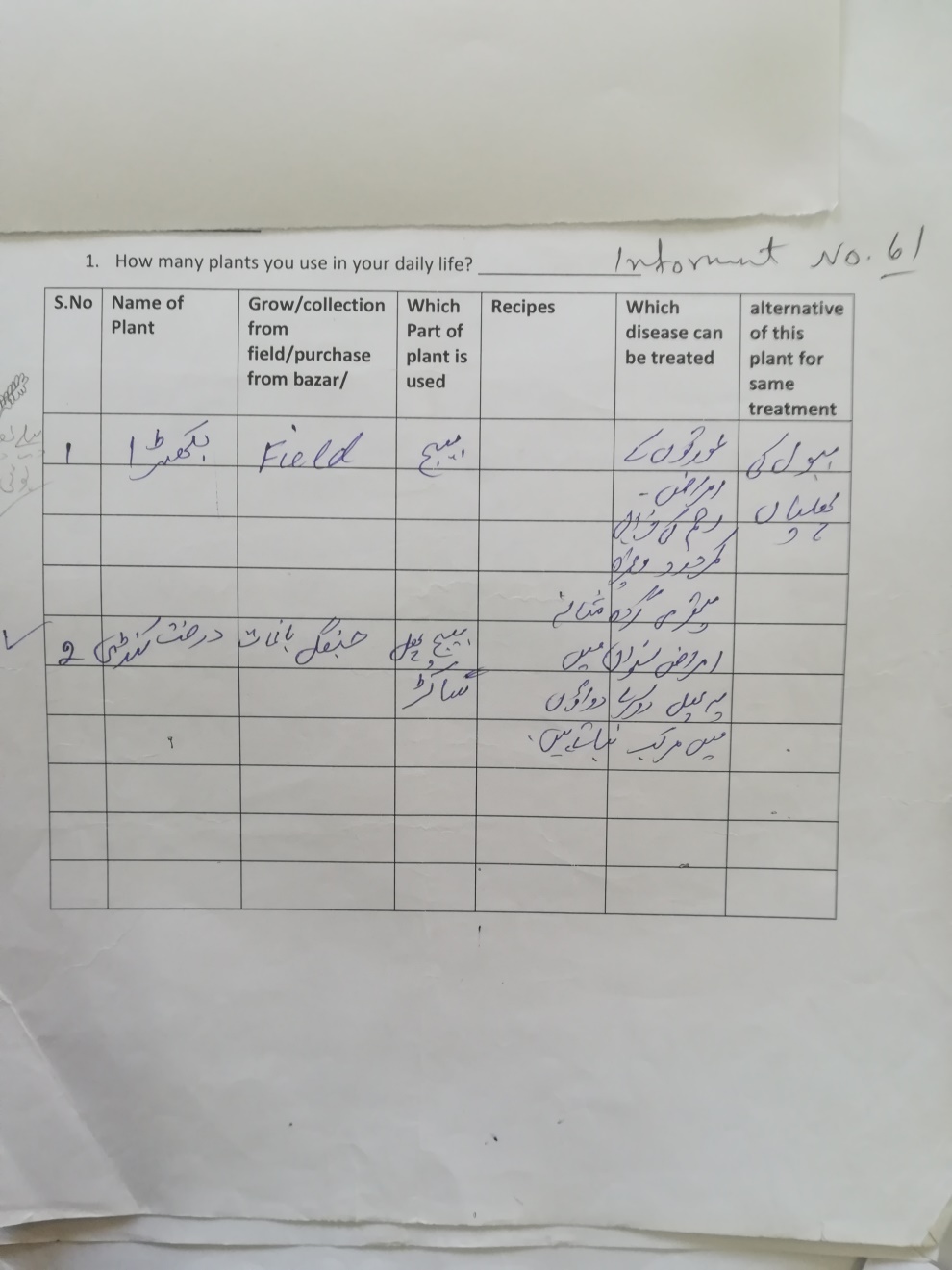


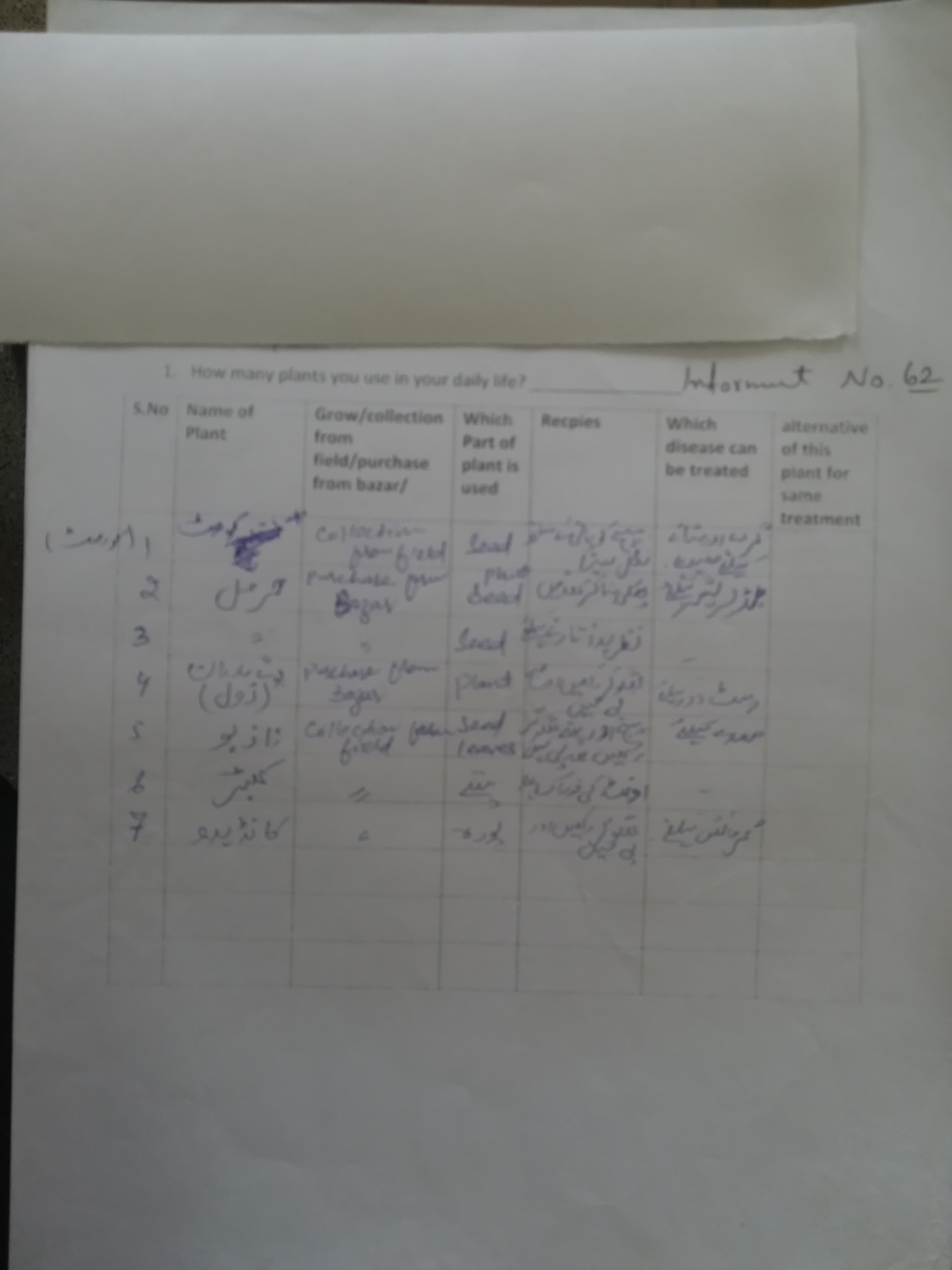


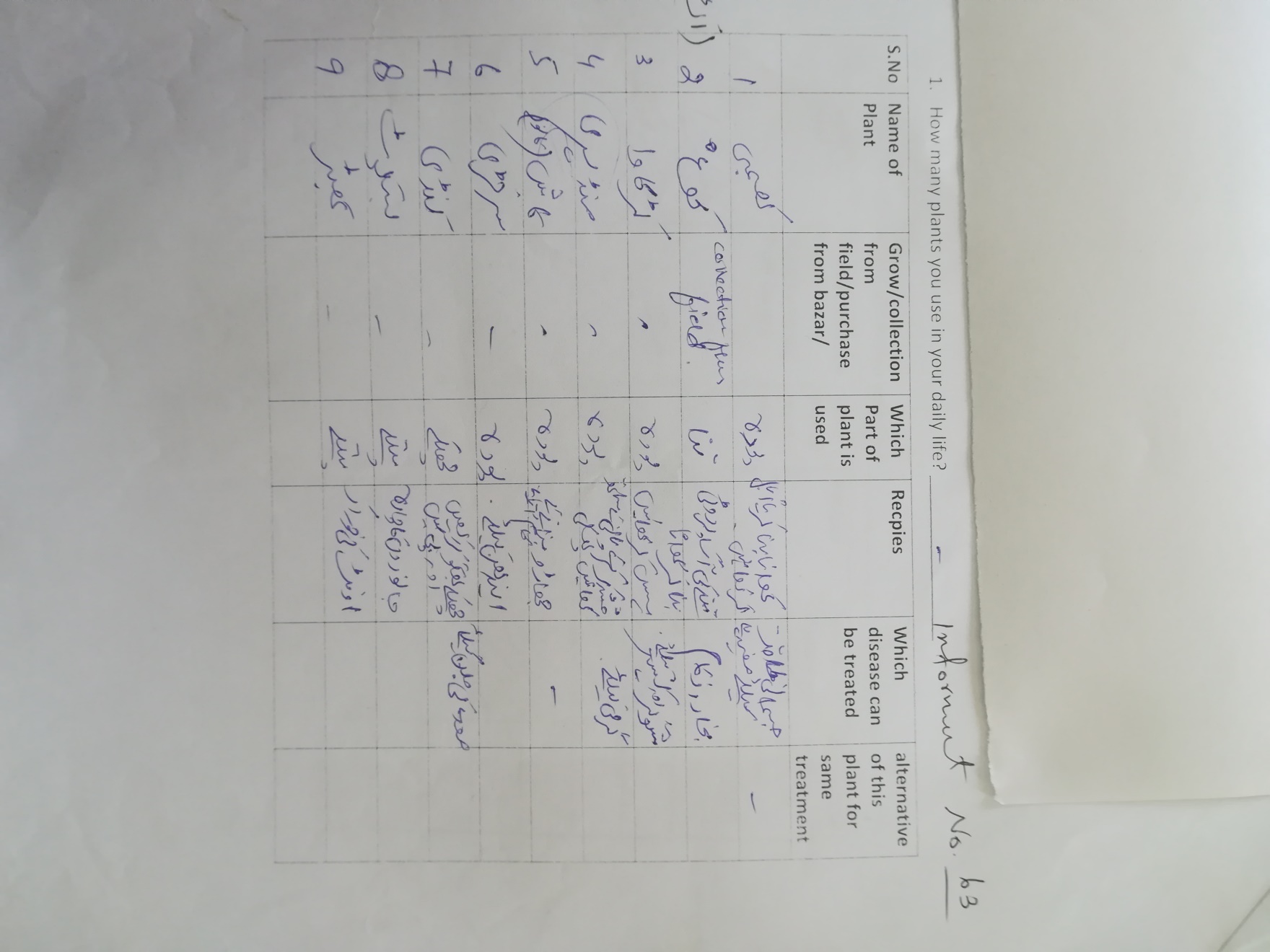


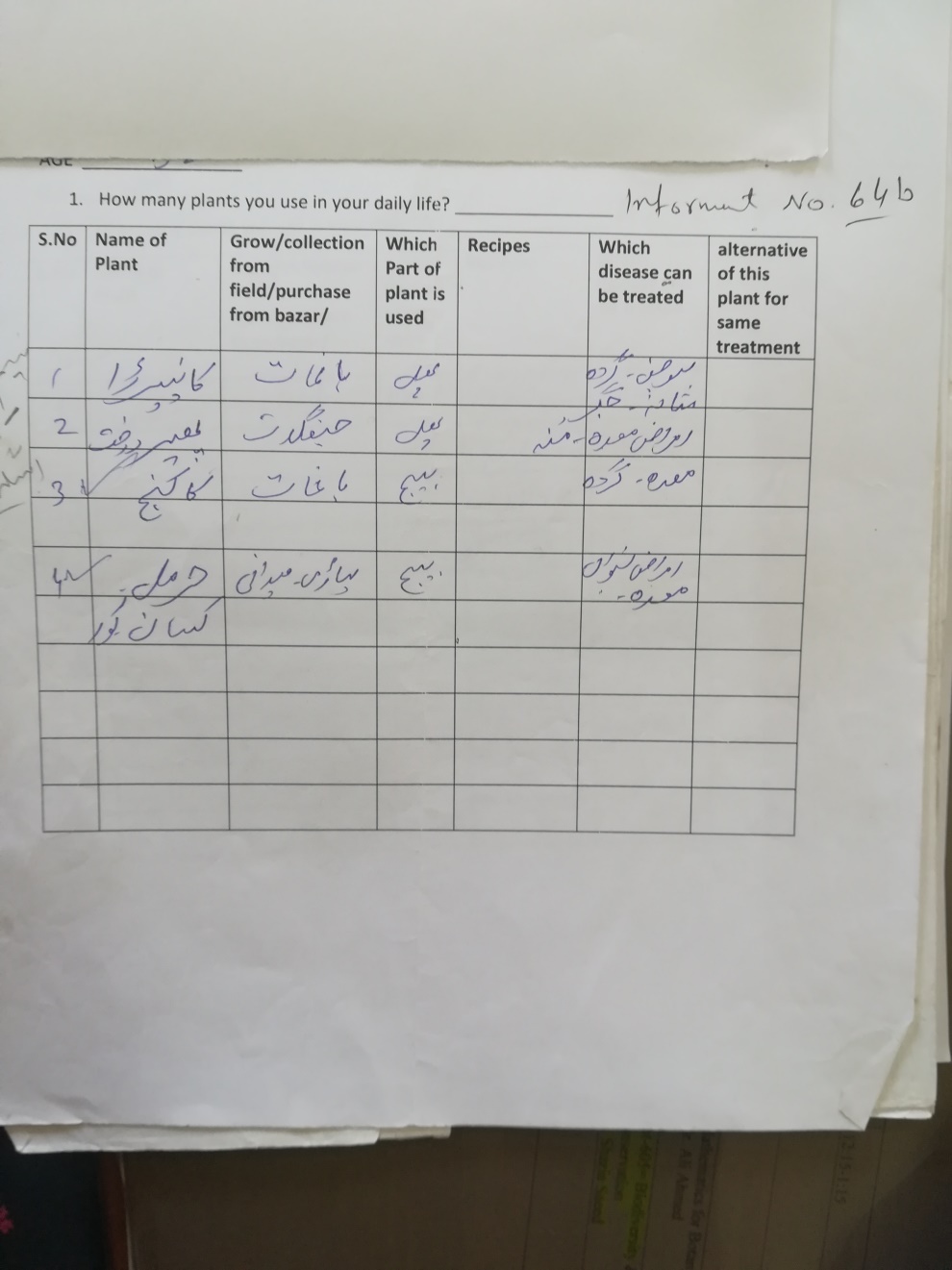


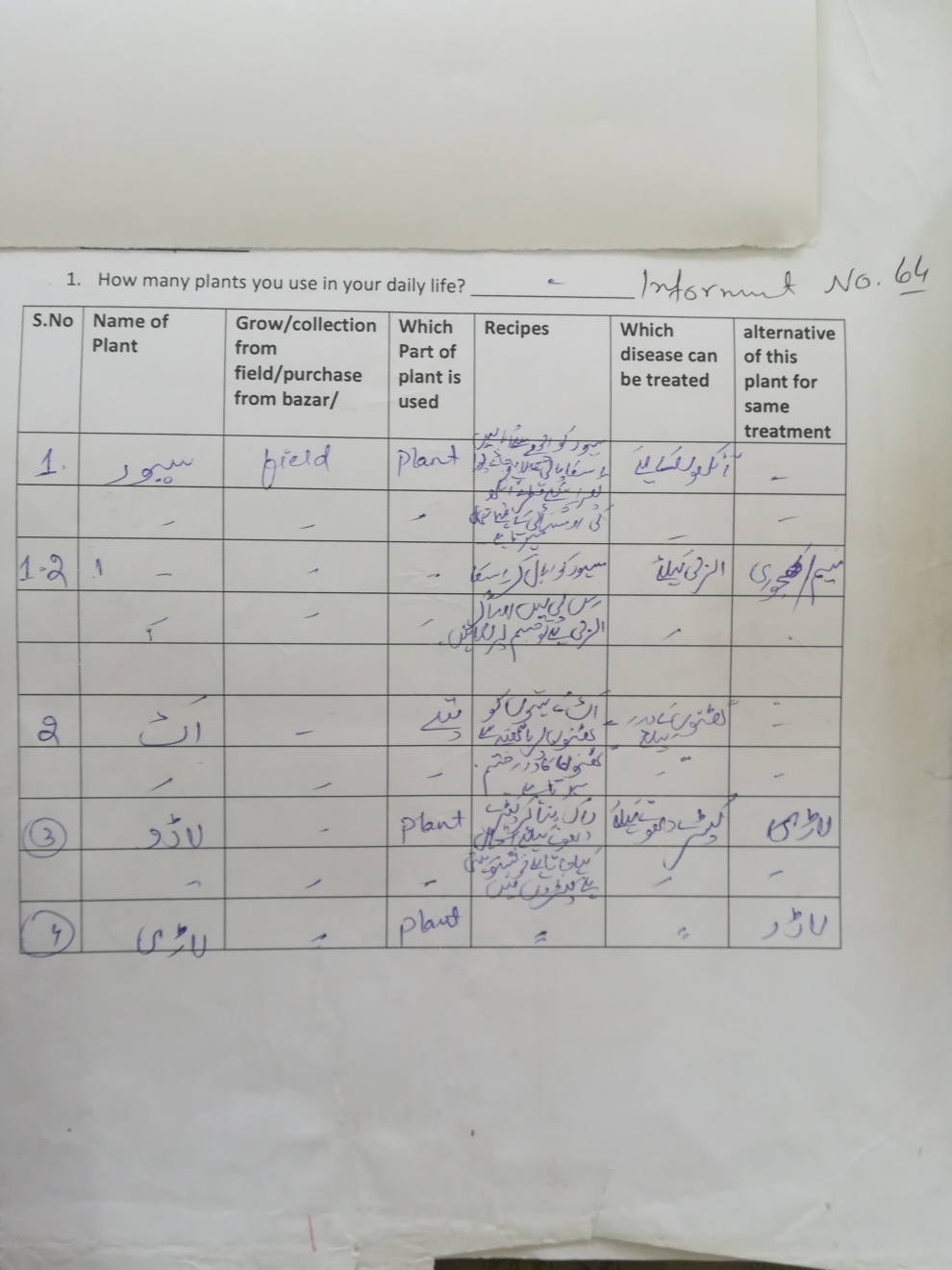


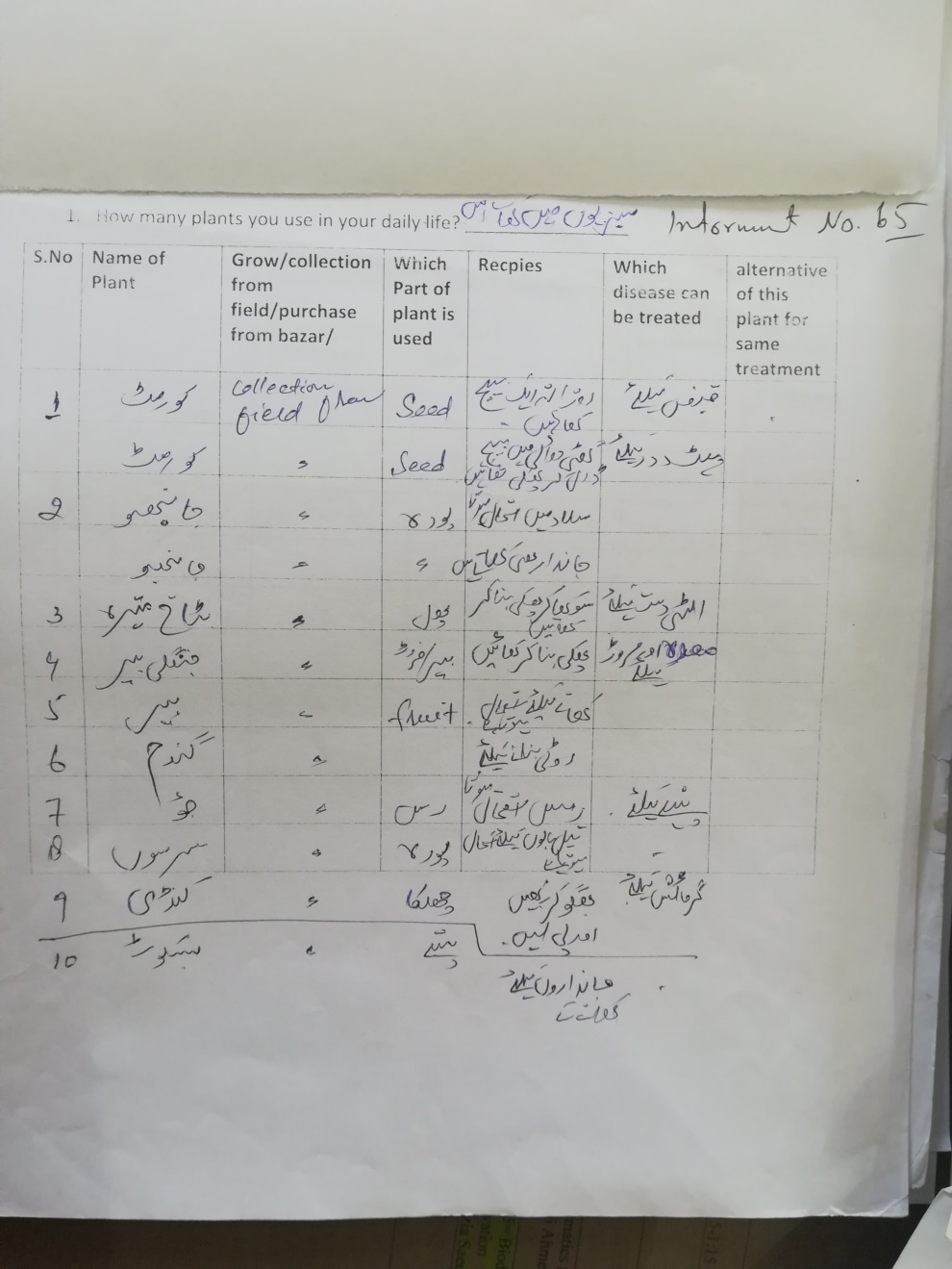


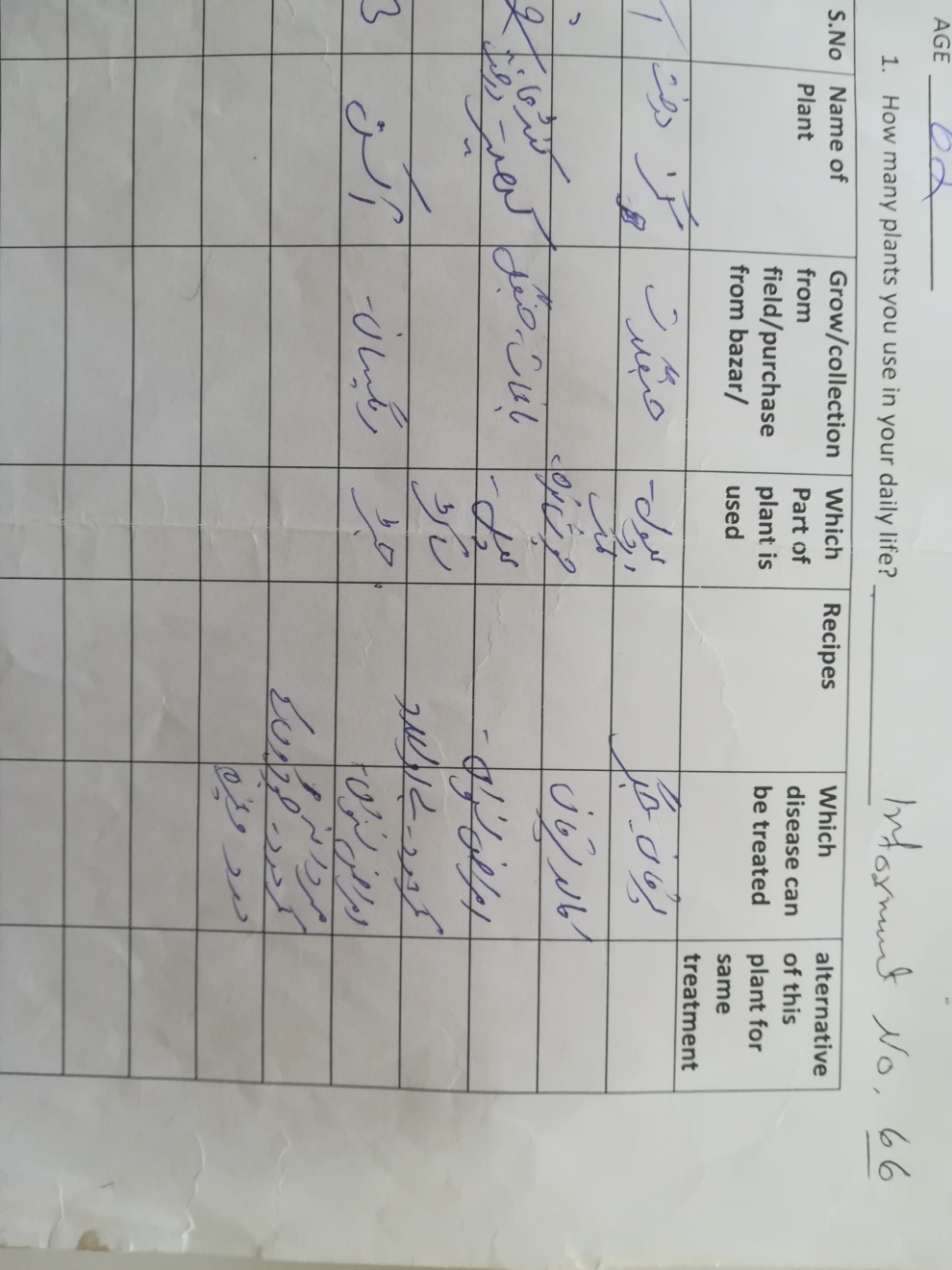

Supplement: S1 File — (DOCX) [file pone.0294989.s001.docx]
