# Supplementary material for: The sustainable use of diverse plants accustomed by different ethnic groups in Sibi District, Balochistan, Pakistan
Source: PLoS One. 2024 Feb 21;19(2):e0294989. doi: 10.1371/journal.pone.0294989 (PMC10880983; doi:10.1371/journal.pone.0294989)
Supplement: S2 File — (PDF) [file pone.0294989.s002.pdf]

ETHNOBOTANICAL QUESTIONNAIRE

DATE 2-2-2022 VILLAGE Ghulam Bulak

NAME Zeenat GENDER Female

LANGUAGE Balochi TRIBE Rind

AGE 62

1. How many plants you use in your daily life? ______________

S.No	Name of Plant	Grow/collection from field/purchase from bazar/	Which Part of plant is used	Recipes	Which disease can be treated	alternative of this plant for same treatment
1	کُر درخت	جنگلات	پھول - درخت<br>کھنک<br>جوش شدہ		یرقان - جگر<br>کالا یرقان	
2	کھمبیر<br>کنڈی درخت	باغات - جنگل	پھل -<br>ساگ		امراض نسواں -<br>گردہ درد - بے اولاد	
3	آکسن	ریگستان -	جڑ		امراض نسواں -<br>مردانہ<br>گردہ درد - جوڑوں کے<br>درد وغیرہ	

استعمال

ETHNOBOTANICAL QUESTIONNAIRE

DATE 3-8-2021 VILLAGE خجک

NAME موسیٰ خان GENDER مرد

LANGUAGE پشتو TRIBE خجک

AGE 60

1. How many plants you use in your daily life? ________________

S.No	Name of Plant	Grow/collection from field/purchase from bazar/	Which Part of plant is used	Recpies	Which disease can be treated	alternative of this plant for same treatment
	مُنڈیری	،،	،،	خشک کر کے گرمی کیلئے کھاتے ہے	،،	
1	ککر	سبی (گاؤں)	پھول / پھلی	پیس کر	شراب / [illegible] درد	
2	کرڑ	سبی (گاؤں)	پھل	خشک کر کھانا	شوگر	
3	اکڑ	سبی (گاؤں)	پتے	پتا + تیل	درد	
4	کھبڑ	سبی (گاؤں)	پھل	کھانے کیلئے / اونٹ کیلئے [illegible]		
5	گورمنٹ	سبی (گاؤں)	پھل / جڑ	پیس کر	شوگر / دانت درد	
6	بیر	سبی (گاؤں)	پھل	کھانے کیلئے		
7	سورج مکھی	سبی (گاؤں)	بیج	کھانے / گھی کیلئے استعمال		
8	جوا بسری	سبی (گاؤں)	فصل	روٹی / انسان جانور		
9	جماسبو	سبی (گاؤں)	بیج	جانداروں کی خوراک		
10	سرسو	سبی (گاؤں)	بیج	تیل نکلتا ہے		
11	بجم	سبی (گاؤں)	پتے	پیس کر	بالوں	
12	کنڈری	سبی (گاؤں)	پتے	جانوروں کی خوراک / بکرہ / اونٹ [illegible] زیادہ		
13	منگ	سبی (گاؤں)	فصل	منگ کی دال [illegible] جاندار منگ کے کھاتے ہیں		
14	کپاس	سبی (گاؤں)	بیج سے	گھی بنتا ہے۔		

CamScanner

پھلی — شوگر کے لیے — گوشت — کانسٹیپیرو

کھار — [illegible] — الرجی (حسیت)

کھاڑ — " — الرجیں

— خشک — بوہ

گرمی کے لیے (گروہ) — خشک — سیور

پیٹ سے خاتون کے لیے — خشک — دامٹرو

کھانے کے لیے — خشک — للکر

" — میر

— اور تیل — بچے کی پیدائش کے بعد — خشک — اور ساگ ۰

ETHNOBOTANICAL QUESTIONNAIRE

DATE 5-4-2022   VILLAGE Khajjak

NAME فاطمہ   GENDER Femall

LANGUAGE Sindhi   TRIBE Khajjak

AGE 80

1. How many plants you use in your daily life? [illegible]

S.No	Name of Plant	Grow/collection from field/purchase from bazar/	Which Part of plant is used	Recpies	Which disease can be treated	alternative of this plant for same treatment
[illegible] 1	پنیر	Purchase from Bazar	ڈوڈے	50 گرام سونف + پنیر ڈوڈے کی پھکی 50 گرام [illegible] + الائچی چینی حسب ذائقہ یا نہ ڈالا مرضی ہے.	پیٹ درد کیلئے بڑے اور بچوں کیلئے	
2	پنیر	Purchase from Bazar.	ڈوڈے	اتارے چھلکے پاؤڈر پنیر پاؤڈر بنا کر گولیاں بنیں اور دوائی بناتیں۔	بواسیر کیلئے	
[illegible] 3	نیم	Collection from field.	پتے	نیم کے پتے آدھا کلو چنے آدھا کلو بادام آدھا کلو مشری آدھا کلو مکس کرکے پاؤڈر بنا لیں	شوگر کیلئے	صبح شام 2-2 چمچ
4	[illegible]	Bazar.	لکڑی	[illegible] کا ایک پیس لے کر جلا لیں پیچ کے ساتھ [illegible] 4-5 قطرے بچے کو دیں	بچوں کے پیٹ درد کیلئے	
5	لونگ	Bazar.		ہر ایک ہنڈیا میں بھی استعمال ہوتا ہے.		

CamScanner

ETHNOBOTANICAL QUESTIONNAIRE

DATE 15-2-2022 VILLAGE Khajjak

NAME [illegible] GENDER male

LANGUAGE Sindhi TRIBE Khajjak

AGE 73

1. How many plants you use in your daily life? —

S.No	Name of Plant	Grow/collection from field/purchase from bazar/	Which Part of plant is used	Recpies	Which disease can be treated	alternative of this plant for same treatment
1	منڈیری	Collection from field	Plant	خشک کر کے پھکی بنا کر پانی کے ساتھ کھائیں	گردہ پتھری کے لیے	
2	کنڈی	"	wood	لکڑی کا کوئلہ بنا کر [illegible] کے ساتھ کھائیں	پیٹ درد کے لیے	
3	سرسوں	"	پتے	کھانا بنانے [illegible]	—	
4	کانو	"	پودا	جھاڑو کے	—	
5	کھجور	"	پتے	[illegible] کے کام آتے ہیں	گردہ کمزور [illegible]	
6	نازبو	"	پودا	[illegible]	زکام کے لیے	
7	لاڑو	"	پودا	کھاد بنتی ہے	پیٹ درد کے لیے	
8	لاڑی	—	پودا	کھاد بنتی ہے	پیٹ درد کے لیے	
9	لسی	—	لوز دار پتے	[illegible]	بخار کے لیے	
	"	"	"	[illegible]	—	

ETHNOBOTANICAL QUESTIONNAIRE

DATE 20-1-2022 VILLAGE کھوکر

NAME مرشی خان GENDER مرد

LANGUAGE پشتو و سندھی TRIBE کھوکر

AGE 67

1. How many plants you use in your daily life? –

S.No	Name of Plant	Grow/collection from field/purchase from bazar/	Which Part of plant is used	Recipes	Which disease can be treated	alternative of this plant for same treatment
1	کیکر	collection from field	پھول/پھلی	پیس کر کھائیں پھلی کو	کالا [illegible] اور شراب	
2	کرڑ	〃	پھل	خشک کر کے کھائیں	شوگر کیلئے	گورچٹ و نیم
3	آک	〃	پتے	پتہ اور تیل کی مالش	جوڑوں کا درد	
4	کھبڑ	〃	پتے	اونٹ کی خوراک	اونٹ کی خوراک	–
5	کورھٹ	〃	پھل	پیس کر پھکی بنائیں	شوگر	
6	بیر	〃	پھل	سیدھا کھا پھل	کھانے کیلئے	–
(7)	جنگلی بیر	〃	پھل	پیٹ کی پھکی بنائیں [illegible]	پیٹ درد اور معدہ درد	
8	بیر	〃	پتے	ابال کر تیل بنائیں	اچھے بالوں کیلئے	نیم کے پتے
9	نیم	〃	پتے	پیس کر تیل کے ساتھ لگائیں	اچھے بالوں کیلئے	
10	کنڈی	〃	پتے	جانوروں کی خوراک	Fooder	
11	کنڈیرو	〃	دودھ	اس کا دودھ [illegible] بکری اور اونٹ کا / پھکی بنا کر کھاتے ہیں	شوگر کیلئے	نیم/آک

ETHNOBOTANICAL QUESTIONNAIRE

DATE 20-2-2022 VILLAGE Silachi

NAME [illegible] GENDER Female

LANGUAGE Sindhi TRIBE Silachi

AGE 63

1. How many plants you use in your daily life? —

S.No	Name of Plant	Grow/collection from field/purchase from bazar/	Which Part of plant is used	Recpies	Which disease can be treated	alternative of this plant for same treatment
1	نیم	/	Seed	صبح کو پانی سا تو کھانے	قبض سے	
2	کانی	/	[illegible]	[illegible]	-	-
3	[illegible]	"	"	[illegible]		کھجور نیم
4	کیکر	field	fist layer flower	[illegible]	کمر درد لیے	
5	[illegible]	Collection for field	plane	[illegible]	[illegible]	

CamScanner

ETHNOBOTANICAL QUESTIONNAIRE

DATE 18-4-2023 VILLAGE Sibi

NAME Shahpari GENDER Female

LANGUAGE 63 Sindhi TRIBE Somoro

AGE ✓

1. How many plants you use in your daily life? ____________

S.No	Name of Plant	Grow/collection from field/purchase from bazar/	Which Part of plant is used	Recipes	Which disease can be treated	alternative of this plant for same treatment
1	اسگندھ	کھیت جنگل	جڑ		عورتوں کا رحم	گوکھرو — بھکھڑا
2	[illegible]	ریگستان، نہری	پتے		اور کمر درد وغیرہ	
		کنارے		امراض آنکھ	[illegible]	
					[illegible] امراض	
3	[illegible]	جنگلات پہاڑوں			[illegible]	
	درخت	میں	چھال		[illegible]	
					کمر درد لیکوریا	
	[illegible] درخت	جنگل، باغات	پھل		جوڑوں درد / امراض [illegible] میں	
4	[illegible] پودہ		پتے		[illegible] میں	
					[illegible]	

Yellow Phol walla

ETHNOBOTANICAL QUESTIONNAIRE

DATE 5-1-2022 VILLAGE Mall

NAME [illegible] GENDER Female

LANGUAGE Balochi TRIBE Gishkori

AGE 65

1. How many plants you use in your daily life? ________________

S.No	Name of Plant	Grow/collection from field/purchase from bazar/	Which Part of plant is used	Recpies	Which disease can be treated	alternative of this plant for same treatment
1/	کاسنی	باغات	بیج		درد معدہ	
2/	تل		تیل	دانتوں کی خشکی دور کرتا ہے تل کا تیل سر میں مالش کرتے ہیں	دانتوں کیلئے	
3/	[illegible]		[illegible]	بچھو کے ڈنگ پر لگاتے ہیں	بچھو کا ڈنگ [illegible]	
4/	گندم		~~Seed~~	روٹی بناتے ہیں	روٹی	
5/	[illegible]	[illegible]	[illegible] مربہ		[illegible]	

ETHNOBOTANICAL QUESTIONNAIRE

DATE 9-1-2021 VILLAGE Kusah

NAME Farzana GENDER Female

LANGUAGE Brahui TRIBE Banglzai

AGE 59

1. How many plants you use in your daily life? ____________

S.No	Name of Plant	Grow/collection from field/purchase from bazar/	Which Part of plant is used	Recpies	Which disease can be treated	alternative of this plant for same treatment
1/	آرچ		پورہ	پیس کر تھلی بنا کر کھالیں	اندرونی معدہ [illegible]	
2/	نازبو		پتوں	پیس کر چٹنی کے ساتھ [illegible] کر کے کھالیں	حاملہ خواتین الٹی [illegible]	
3/	[illegible]		بیج	(جانوروں [illegible])	جانوروں کے دودھ میں اضافہ [illegible]	
4/	[illegible]	ریگستان [illegible] کھیتوں میں	پھول		اندرونی معدہ [illegible]	
5/	جو	field	جو	پیس کر روٹی بنائی جاتی ہے		

ETHNOBOTANICAL QUESTIONNAIRE

DATE 7-4-2021 VILLAGE Bakhro

NAME غلام فاطمہ GENDER Female

LANGUAGE Balochi TRIBE Ghulam bolak

AGE 73

1. How many plants you use in your daily life? ________________

S.No	Name of Plant	Grow/collection from field/purchase from bazar/	Which Part of plant is used	Recipes	Which disease can be treated	alternative of this plant for same treatment
1	گوکھرو جنگلی	ریگستانی زمین	پھول -		بواسیر، قبض	
		باغات	بیج		ورم معدہ	
2 (کھانسی نزلہ)	شنبز [illegible]	باغات، ریگستان	جڑ، پھول		[illegible]	
					[illegible]	
3	خرشتر [illegible]	جنگلات	پتے		بخار [illegible]	
					[illegible]	
					[illegible]	
					[illegible]	
4 Euphorbia	[illegible] بوٹی	باغات، میدان	پتے		[illegible]	
	دودھی خورد					

[illegible] (margin note beside row 4)

ETHNOBOTANICAL QUESTIONNAIRE

DATE 12-11-2021

NAME خدا بخش

LANGUAGE Sindhi

AGE 42

VILLAGE Khajjak.

GENDER Male

TRIBE Khajjak (ملانی)

1. How many plants you use in your daily life? ____________

S.No	Name of Plant	Grow/collection from field/purchase from bazar/	Which Part of plant is used	Recipes	Which disease can be treated	alternative of this plant for same treatment
1	پنیر	پہاڑوں میں -	پھل		معدہ - قبض تکلیف صاف خون	
2	نیم درخت	جنگلات، کھیت	چھال - پھل پتے		صاف خون - بواسیر - پھوڑے وغیرہ	
3	[illegible] دھمالہ	پہاڑوں میدان	مکمل پودہ پتے		[illegible] پیشاب - [illegible] جلد کی امراض سلح وغیرہ	
	آک - [illegible] جامنی پھول والا -	ریگستان، پہاڑی علاقے	پتے - پھول جڑ		امراض معدہ مردانہ امراض دمہ	

ETHNOBOTANICAL QUESTIONNAIRE

DATE 13-07-2021

NAME Ahmad Khan

LANGUAGE Sindhi

AGE 45

VILLAGE Khajjak

GENDER Male

TRIBE Khajjak

1. How many plants you use in your daily life? ________________

S.No	Name of Plant	Grow/collection from field/purchase from bazar/	Which Part of plant is used	Recipes	Which disease can be treated	alternative of this plant for same treatment
1	بکھڑا	Field	بیج		عورتوں کے امراض - رحم کی سوزش، کمر درد وغیرہ	ببول کی پھلیاں ج د
					معدہ، گردہ، مثانے	
2	درخت کنڈی	جنگل، باغات	بیج، پھول، [illegible]		امراض سوزش میں، پھل دوسری دواؤں میں مرکب شامل ہیں۔	
	۲					

پیلے پھول والی بوٹی

ETHNOBOTANICAL QUESTIONNAIRE

DATE 20-1-2022

VILLAGE کملی ماشنجی Sافظی

NAME شمان

GENDER male

LANGUAGE شمان سرائیکی

TRIBE ماشنجی

AGE 48

1. How many plants you use in your daily life? ____________

S.No	Name of Plant	Grow/collection from field/purchase from bazar/	Which Part of plant is used	Recpies	Which disease can be treated	alternative of this plant for same treatment
(اسورھٹ) 1	[illegible] کوھٹ	Collection from field	Seed	بیج کو پانی کے ساتھ نگل لینا	گردے اور مثانے کیلئے مفید ہے۔	
2	حرمل	purchase from Bazar	Plant Seed	دھونی بنا کر دیتے ہیں	جلد [illegible] کیلئے	
3	"	"	Seed	نظر اتارنے کیلئے	—	
4	بوٹی مادران (اڑول)	Purchase from Bazar	Plant	ابال کر رکھیں اور صبح پی لیں	پیٹ درد کیلئے معدے کیلئے	
5	زانڑبو	Collection from field	Seed leaves	بیج اور پتے ابال کر رکھیں صبح پی لیں		
6	کھبڑ	//	پتے	اونٹ کی خوراک ہے	—	
7	کانڈیرو	"	پورا	ابال کر رکھیں اور پی لیں	خارش کیلئے۔	

ETHNOBOTANICAL QUESTIONNAIRE

DATE 21-2-2022 VILLAGE Sibi

NAME مولاداد GENDER Male

LANGUAGE Sindhi (Pashtoo) TRIBE Barozai

AGE 66

1. How many plants you use in your daily life? -

	S.No	Name of Plant	Grow/collection from field/purchase from bazar/	Which Part of plant is used	Recpies	Which disease can be treated	alternative of this plant for same treatment
	1	کھمبی		پودہ	کھانا بنا کر یا ابال کر کھائیں	جسم کی طاقت کیلئے مفید ہے	-
(آک)	2	کوع	collection from field.	تنا	تنے کی آٹا کر روٹی بنا کر کھانا	بخار، زکام	
	3	کڑکاوا	-	پودہ	پیس کر کھائیں	شوگر اور کینسر کیلئے	
	4	صندسیری	-	پودہ	خشک کر کے نمک کے ساتھ کھائیں ڈلیکی	گرمی کیلئے	
	5	کاشن (کانو)	-	پودہ	جھاڑو بنانے کے کام آتا ہے	-	
	6	سرکڑی	-	پودہ	ایندھن کیلئے		
	7	کنڈی	-	چھلکے	چھلکے کھا کر رنگ کرتے ہیں اور بھی بنتی ہیں	معدے کی جلن کیلئے	
	8	لسٹرٹ	-	پتے	جانوروں کا چارہ		
	9	کھبڑ	-	پتے	اونٹ کی خوراک		

ETHNOBOTANICAL QUESTIONNAIRE

DATE 23/4/2022

NAME عبدالحکیم

LANGUAGE سندھی

AGE 52

VILLAGE آملی

GENDER Male.

TRIBE سیلاچی

1. How many plants you use in your daily life? ________________

S.No	Name of Plant	Grow/collection from field/purchase from bazar/	Which Part of plant is used	Recipes	Which disease can be treated	alternative of this plant for same treatment
1	کانیسری / ڈرا	باغات	پھل		سوزش - گردہ، مثانہ - جگر	
2	بھیر دخت	جنگلات	پھل		امراض معدہ - مثلہ	
3	گاؤ کنج	باغات	بیج		معدہ - گردہ	
4	حرمل - کسان بور	پہاڑی - میدانی	بیج		امراض شوگر معدہ -	

سیم

کعبہ

اسلام کوٹ

ETHNOBOTANICAL QUESTIONNAIRE

DATE 10-1-2022 VILLAGE Talli

NAME جان بی بی GENDER female

LANGUAGE 39 سرائیکی TRIBE سیالوی

AGE 39

1. How many plants you use in your daily life? –

S.No	Name of Plant	Grow/collection from field/purchase from bazar/	Which Part of plant is used	Recipes	Which disease can be treated	alternative of this plant for same treatment
1.	سیوڑ	field	plant	سیوڑ کو اچھی طرح [illegible] اس کا پانی [illegible] کی اور [illegible] استعمال ہوتا ہے	آنکھوں کے لیے	–
	–	–	–		–	–
1-2	١ –	–	–	سیوڑ کو ابال کر اس کا رس [illegible] الرجی کے لیے [illegible]	الرجی کے لیے	نیم / کھجوری
	٢	–	–		–	
2	اک	–	پتے	اک کے پتوں کو گھٹنوں پر باندھنے سے گھٹنوں کا درد ختم ہوتا ہے	گھٹنوں کے درد کے لیے	–
	–	–	–		–	–
3	لاڑو	–	plant	[illegible]	[illegible]	لاڑاں
	–	–	–	[illegible]	–	–
4	لاڑی	–	plant	–	–	لاڑو

CamScanner

ETHNOBOTANICAL QUESTIONNAIRE

DATE 19-1-2022 VILLAGE Talli

NAME [illegible] GENDER Female

LANGUAGE Sindhi TRIBE Silachi

AGE 48

1. How many plants you use in your daily life? سبزیوں میں کھاتے ہیں

S.No	Name of Plant	Grow/collection from field/purchase from bazar/	Which Part of plant is used	Recpies	Which disease can be treated	alternative of this plant for same treatment
1	کورمٹ	Collection field [illegible]	Seed	روزانہ ایک چمچ کھاتے ہیں۔	قبض کیلئے	
	کورھٹ	"	Seed	دھنی دوائی میں چمچ ڈال کر پھکی کھاتے ہیں	پیٹ درد کیلئے	
2	جانبھو	"	پودا	سلاد میں استعمال ہوتا		
	وانبھو	"	"	جانداروں کو کھلاتے ہیں		
3	[illegible]	"	پھول	ستو کھا کر پھکی بنا کر کھاتے ہیں	الٹی دست کیلئے	
4	جنگلی بیر	"	بیر/فروٹ	پھکی بنا کر کھاتے ہیں	معدہ اور مروڑ کیلئے	
5	بیر	"	fruit	کھانے کیلئے استعمال ہوتا ہے۔		
6	گندم	"		روٹی بنانے کیلئے		
7	جو	"	رس	رس استعمال ہوتا	[illegible] کیلئے۔	
8	سرسوں	"	پودا	تیل صابون کیلئے استعمال ہوتا ہے		
9	[illegible]	"	چھلکا	[illegible]	[illegible] کیلئے	
10	[illegible]	"	پتے	جانداروں کیلئے کھلاتے		

ETHNOBOTANICAL QUESTIONNAIRE

DATE 15-2-2022 VILLAGE Talli

NAME صحبت بی بی GENDER Female

LANGUAGE سرائیکی TRIBE سیالوی

AGE 62

1. How many plants you use in your daily life? 4

S.No	Name of Plant	Grow/collection from field/purchase from bazar/	Which Part of plant is used	Recpies	Which disease can be treated	alternative of this plant for same treatment
1	[illegible]	Collection from field	جڑ	جڑ کو دھو کر ابال کر دیسی گھی ڈالیں اور [illegible] [illegible] [illegible] ڈالیں	امراض نسواں [illegible] کے لئے	
	″	″	″			
			جڑ	جڑ [illegible] کو 24 گھنٹے [illegible] اور [illegible]	خون کی الٹی کے لئے	
2	کھنبڑ	″	پتے	اونٹنی کی خوراک		
3	کیکر	″	پھول	پھول کو ابال کر [illegible] پانی سے [illegible]		
		″	″	[illegible] [illegible] سفید [illegible]	[illegible] کے لئے	
	″	″	″			
	″	″	پھول	کیکر کے پھول [illegible] اور بادام مکس کر کے [illegible] بنا لیں کمر درد	کمر درد	
	″	″	″			
	″	″	″	[illegible]	لیکوریا کے لئے	

(4) لئی ″ پتے پتوں کو [illegible] کر [illegible] اور پانی کے ساتھ [illegible]

ETHNOBOTANICAL QUESTIONNAIRE

DATE ____________ VILLAGE ____________

NAME ____________ GENDER ____________

LANGUAGE ____________ TRIBE ____________

AGE ____________

1. How many plants you use in your daily life? ____________

S.No	Name of Plant	Grow/collection from field/purchase from bazar/	Which Part of plant is used	Recpies	Which disease can be treated	alternative of this plant for same treatment
5	نیم	Collection field [illegible]	پتے	پتوں کو ابال کر [illegible]	سرخ باد	
6	[illegible]	〃	دودھ	[illegible] اور پانی کو دانوں پر لگائیں اور [illegible] فائدہ ہوتا ہے	دانوں [illegible]	
	〃	〃	〃		〃	
7	آک	〃	پتے	پتوں کو [illegible] جوش دے کر پانی [illegible] دو تین بار [illegible] کھائیں	[illegible] جوڑوں [illegible]	
	〃	〃	〃			
	〃	〃	〃			
8	[illegible]	〃	بیج	تیل بنتا ہے		
9	تل	〃	بیج	تل اور [illegible] کھائیں	ماہواری [illegible]	

ETHNOBOTANICAL QUESTIONNAIRE

DATE 13-4-2021

NAME مریم

LANGUAGE Sindhi

AGE 60

VILLAGE Khajjak

GENDER Female

TRIBE Khajjak

1. How many plants you use in your daily life? ______

S.No	Name of Plant	Grow/collection from field/purchase from bazar/	Which Part of plant is used	Recpies	Which disease can be treated	alternative of this plant for same treatment
1	کالو	Collection from field	ڈوڈہ	جھاڑو بنانے کے		
2	بلڈ بیری	/	ڈوڈہ	جوش دے کر اس کا [illegible] ابال کر کھانا	جسم کی خارش کے لیے	
3	فوگلی	/	ڈوڈہ	ابال کر کھاتے ہیں	درد یا سوزش کے لیے	
4	ڈوڈیٹ	/	ڈوڈہ	اونٹ کی خوراک ہے		
5	آک	/	ڈوڈہ		گھٹنوں کے درد کے لیے	
6	لاڑی	/	ڈوڈہ	[illegible] کو بنا کر کھاتے ہیں	پیٹ کے درد [illegible] کے لیے	
7	نیم	/		پانی کے ساتھ روزانہ [illegible] کھاتے ہیں	ضعف کے لیے	

ETHNOBOTANICAL QUESTIONNAIRE

DATE 15-2-2022 VILLAGE Talli

NAME [illegible] GENDER Female

LANGUAGE Sindhi TRIBE Siiachi

AGE 61

1. How many plants you use in your daily life? –

S.No	Name of Plant	Grow/collection from field/purchase from bazar/	Which Part of plant is used	Recpies	Which disease can be treated	alternative of this plant for same treatment
1	کڑتاوا	Collection from field	تنا + پتہ + پھول	خشک کر کے پوڈر بناتے ہیں	[illegible] + شوگر [illegible] + معدہ	–
2	اسگند دو	"	Root	صاف کر کے خشک کر کے پھکی بنا کر دوائی	[illegible] اور کمر درد	–
3	دھوسڑی	"	plant	بکریوں کی خوراک		
4	مسری	"	Plant	ابال کر پانی پیتے ہیں	بخار اور شوگر کیلئے	
5	کوڑتمہ	"	Seed	[illegible] Seed	جوڑوں کے لیے	
6	آک	"		آگ پر روٹی بناتے ہیں تنا	بخار کیلئے	
7	جو	"	Seed	آٹا بنتا ہے	روٹی کیلئے	
8	کنڈی	"		جانوروں کی خوراک ہے	دودھ زیادہ ہوتا ہے	

CamScanner

ETHNOBOTANICAL QUESTIONNAIRE

DATE 3-1-2022 VILLAGE Khajjak

NAME روزینہ بی بی GENDER Female

LANGUAGE Sindhi TRIBE Sabzani (magsi)

AGE 88

1. How many plants you use in your daily life? ______________

S.No	Name of Plant	Grow/collection from field/purchase from bazar/	Which Part of plant is used	Recpies	Which disease can be treated	alternative of this plant for same treatment
1	سیہور	Collection field fm	پودا	بھگو کر رکھیں 24 گھنٹے اور پھر پی لیں	الرجی کیلئے	
2	لئی	✓	پتے	راکھ بنا کر زخم پر رکھیں	زخم کیلئے	
3	کانڈیرو	✓	پودا	پھکی بنا کر کھائیں	شوگر کیلئے	
4	جوشنگ	✓	Seed	آٹا بنتا ہے	روٹی کیلئے	
5	گرنگ سنگر	✓	Seed	آٹا بنتا ہے	روٹی پکتی ہے	
6	سرسوں	✓	پودا	ابال کر کھائیں	بچے کی پیدائش کے بعد [illegible] ہونا چاہیئے	

CamScanner

ETHNOBOTANICAL QUESTIONNAIRE

DATE 3-1-2022 VILLAGE Khajjak

NAME آمنہ GENDER Female

LANGUAGE Sindhi TRIBE Sabhrani ([illegible])

AGE 49

1. How many plants you use in your daily life? 5

S.No	Name of Plant	Grow/collection from field/purchase from bazar/	Which Part of plant is used	Recpies	Which disease can be treated	alternative of this plant for same treatment
1	ڈنگ	collection from field	دال	کھانا بنانے کام آتا ہے		
2	کپاس	"	بیج	بیج کو [illegible]	[illegible] بیماری کیلئے	
3	کانڈیرو	"	پودا	پوڈر کر کے گولی بناتے ہیں	شوگر کیلئے	کوڑتمہ / نیم
4	لاڑی	"	پودا	جلا کر راکھ بناتے پیس [illegible]	[illegible] بیماری کیلئے	لاڑھو
5	لاڑو	"	پودا	[illegible]	کپڑے اور خوشبودار	لاڑی
6	[illegible]	"	پتے	پیس کر چٹنی کے ساتھ کھاتے ہیں	حاملہ خواتین کے لیے	
7	پیاز	"	رس	پیاز کا رس	کان کے درد کیلئے مفید ہے	سیور
7.2	پیاز	"	رس	پیاز کا رس	بچوں کی ٹھنڈ لگ [illegible]	نیم
8	[illegible] (املی)	"	پودا	پیس کر [illegible]	[illegible] کیلئے	
9	نیم	"	پتے	پتے پیس کر تیل کے ساتھ بالوں پر لگاتے ہیں	بالوں کیلئے	بسکوٹ
10	کوڑتمہ	"	پھل	[illegible]	شوگر کیلئے	

ETHNOBOTANICAL QUESTIONNAIRE

DATE 14-1-2022

NAME کونج

LANGUAGE Sindhi

AGE 40

VILLAGE Khajjak

GENDER Female

TRIBE Khajjak (کھاجک)

1. How many plants you use in your daily life? —

S.No	Name of Plant	Grow/collection from field/purchase from bazar/	Which Part of plant is used	Recpies	Which disease can be treated	alternative of this plant for same treatment
1	[illegible]	Collection field Olu	پتے		امراض معدہ امراض نسواں	
2	[illegible]	Collection field Olu	جڑ		عورتوں کے رحم اور [illegible] درد کے لیے	
(جنگلی بیر) 3	کلش	-	بیر		[illegible] کے لیے	
4	[illegible]	-	پھل		امراض نسواں	
5	[illegible]		پتے	[illegible] ٹکی بنا کر [illegible]	[illegible] کے لیے	
6	[illegible]	-	[illegible]		گردہ پتھری کے لیے	
7	[illegible]	-	[illegible]	ٹکی بنا کر [illegible]	شوگر + کینسر موٹاپا [illegible] کے لیے	
8	آشہ	-	پھول		معدے کے لیے اور دمہ کے لیے	

ETHNOBOTANICAL QUESTIONNAIRE

DATE 14-4-2022

NAME اماں خاتون

LANGUAGE Sindhi

AGE 57

VILLAGE Khajjak

GENDER Female

TRIBE Khajjak

1. How many plants you use in your daily life? –

S.No	Name of Plant	Grow/collection from field/purchase from bazar/	Which Part of plant is used	Recpies	Which disease can be treated	alternative of this plant for same treatment
1	تورڑکھی	Collection from field	Plant	اونٹ کے کھانے کیلئے	اونٹ کی خوراک	–
2	گدڑول	–	رس	پیس کر اسکا رس لگاتے ہیں	دکھ کیلئے (الرجی)	
3	پھوسڑی	–	دودھ	بکریوں کی خوراک		
(آکڑ) 4	کھلیر	–	تنا	جلا کر دھوئیں میں بیٹھا جاتا ہے	الرجی کیلئے	
5	لاڑی	–	دودھ	جلا کر کھار بناتے ہیں	پیٹ درد کیلئے	
6	کھوخ	–	تنا	مٹی کی آگ پر روٹی بنا کر کھانا	بخار و زکام کیلئے	
7	نیم	–	پتے + بیج	پیس کر ٹکی بنا کر لگاتے ہیں	شوگر، پھنسی	
8	گاجر	–	Seed	پیس کر دودھ میں ڈال کر پیتے	[illegible] کیلئے	
9	کھن	Collection from field	پھول	تیل میں گرم کر کے [illegible]	گھٹنوں کے درد کیلئے	

کھانے کے استعمال ہوتا ہے .

ETHNOBOTANICAL QUESTIONNAIRE

DATE 13-1-2022 VILLAGE Lohni

NAME رقیہ GENDER Female

LANGUAGE Sindhi (Pashtoo) TRIBE Lohni

AGE 43

1. How many plants you use in your daily life? چیرو پھکی + چاٹ (زیرہ + اجوائن)

S.No	Name of Plant	Grow/collection from field/purchase from bazar/	Which Part of plant is used	Recpies	Which disease can be treated	alternative of this plant for same treatment
1	کوڑمٹ	Collection from field	Fruit	گندم کے ساتھ ابال کر دھوپ میں خشک کریں اور کسی جار میں محفوظ کریں	شوگر کے لیے	
2	ناڑبو	〃	Plant	پتے اور بیج کو ابال کر رکھیں اور ہر 4 گھنٹے بعد وہ پانی پی لیں	معدہ اور قبض کے لیے	
3	کپاس	〃	Seed	بیج کو حمام دستہ میں کوٹ کر جو تیل نکلے گا وہ ہڈی پر لگائیں اور بیج کا بورا اکٹھا لگا کر پٹی باندھ دیں	ٹوٹی ہڈی کے لیے	
4	آک	〃	پھول + پتے + جڑ	پیس کر گولی بنا کر کھائیں	امراض معدہ، مردانہ امراض، دمہ کی بیماری کے لیے	—
5	سیور	〃	Plant	سیور کو 24 گھنٹے تک بھگو کر رکھیں اور اگلے دن 24 گھنٹے گزرنے کے بعد گڑ کے ساتھ پی لیں	الرجی، گلے اور گرمی دانوں کے لیے	
6	نیم	〃	پتے	تیل میں ملا کر بالوں کو لگائیں	بال مضبوط اور لمبے ہوتے ہیں۔	
7	کرڑ	〃	لکڑی	لکڑی کو جلا کر کوئلہ بنائیں اور کوئلہ کوٹ کر دودھ کے ساتھ پھکی کھائیں	کمر درد اور ہڈیوں کے لیے۔	

CamScanner

ETHNOBOTANICAL QUESTIONNAIRE

DATE 5-4-2022 VILLAGE Khajjak

NAME Zulaikha GENDER Female

LANGUAGE Sindhi TRIBE Khajjak

AGE 35

1. How many plants you use in your daily life? ____________

	S.No	Name of Plant	Grow/collection from field/purchase from bazar/	Which Part of plant is used	Recpies	Which disease can be treated	alternative of this plant for same treatment
کاڑھا	1	ڈلیہ	Bazar		زیرہ 1 چمچ<br>اجوائن 1/2 چمچ<br>سونف 1/2 چمچ<br>دیسی لسی آدھا چمچ<br>ابال کر گلاس میں بھر کر رکھ دے اور وہاں پانی کی جگہ پیس استعمال کریں.	بچے پیدا ہونے کے بعد جان کو [illegible] بڑھنے میں پیٹ کی صفائی کے لیے.	
	/	/	/	/			
	/	/	/	/			
	/	/	/	/			
	2	لونگ	Bazar.		جیسے دانت میں درد ہو چبایا کریں.	دانت میں درد کے لیے	

ETHNOBOTANICAL QUESTIONNAIRE

DATE: 3-1-2022 VILLAGE: Khajjak

NAME: حمیدہ GENDER: Female

LANGUAGE: Sindhi TRIBE: سبانی (Sabhani)

AGE: 39

1. How many plants you use in your daily life? 1

S.No	Name of Plant	Grow/collection from field/purchase from bazar/	Which Part of plant is used	Recpies	Which disease can be treated	alternative of this plant for same treatment
1	لُنی / لُو	Collection from field	Leaves	سرسوں کے تیل میں آدھی [illegible] ڈالیں اور نیم گرم حالت میں رات کے وقت بچے کے سر اور پسلیوں پر مالش کریں۔ بچے کو ہوا نہ لگے	بچے کی بیماری کیلئے	
2	کیکر	–	پھول	پھولوں کو صاف کرنے کے بعد چینی کے ساتھ لیں	زیادہ حیض آنے میں ماہواری کو کنٹرول کرنے	–
3	کھبڑ	–	پتے	پانی میں بھگو کر رکھیں پھر خشک کریں خشک کرنے کے بعد پھکی بنا کر پانی کے ساتھ پیئیں	پیٹ جلن اور قبض کیلئے	
4	آکانڈ ریدو	–	جڑ	بھگو کر رکھیں اور جڑ نکال کر پیئیں پیٹ درد کیلئے	پیٹ درد کیلئے، کمر درد کیلئے	
5	کنڈیر	–	سالم	ابال کر استعمال میں لائیں [illegible] میں بھر کالی چائے کے ساتھ آدھا چمچ ملا کر پئیں اور جس جگہ درد ہو اسی پر رکھ کر سو جائیں۔	ہڈی درد یا ٹوٹی ہڈی درد کیلئے	
6	نیم (نیموری)	–	بیج	نیم کے بیج کو کھانے سے شوگر کو کنٹرول کیا جا سکتا ہے	شوگر کیلئے	
7	سیور	–	دودھ	بھگو کر رکھیں اور [illegible] کے بعد پانی پی لیں	دالوں اور الرجی کیلئے	

CamScanner

ETHNOBOTANICAL QUESTIONNAIRE

DATE 14-1-2022 VILLAGE Khajjak

NAME پروا GENDER Female

LANGUAGE Pashtoo TRIBE Khajjak

AGE 33

1. How many plants you use in your daily life? –

S.No	Name of Plant	Grow/collection from field/purchase from bazar/	Which Part of plant is used	Recpies	Which disease can be treated	alternative of this plant for same treatment
✓ 1	کاسنی	collection from field	دانہ تخم	بازار میں تیار ملتا ہے۔	گردے اور مثانے کے لیے	
2	اسپغول	/	دانہ		سر کی خشکی کے لیے معدہ کے لیے	
3	کبریر	/	پھول	خشک کر کے پھکی بنا کر کھائیں۔	اندرونی زخموں کے لیے	
4	نیم	/	پتے۔	پیس کر پھکی بنا کر کھائیں۔	شوگر اور [illegible] کے لیے	
5	آک	/	پھول	پھکی بنا کر	معدے کے لیے	
6	کھبٹر	/	پتے	اُبال کر پیئیں۔	امراض معدے کے لیے	
7	کوڑتمہ	/	پھل	ٹکڑے ٹکڑے کر کے اس کو [illegible] کر کے [illegible]۔	گردوں کی صفائی اور شوگر کے لیے۔	
8	اسطخدوس	/	پھل		معدے کے لیے، امراض [illegible]۔	

ETHNOBOTANICAL QUESTIONNAIRE

DATE 4-3-2022 | VILLAGE Sibi
NAME نگینہ | GENDER Female
LANGUAGE Sibaiki | TRIBE [illegible]
AGE 31

1. How many plants you use in your daily life? —

S.No	Name of Plant	Grow/collection from field/purchase from bazar/	Which Part of plant is used	Recpies	Which disease can be treated	alternative of this plant for same treatment
1	جانبھو	Collection from field	Oil	جسم پر مالش کریں	الرجی	
2	بیر	✓	Fruit	کھانے کیلئے		
3	نازبو	✓	پودہ	کاڑھا بنا کر عورتوں کو پلایا جائے	اولاد کیلئے	
4	کوڑتمہ	✓	پھول	[illegible]	شوگر کیلئے	
		✓		[illegible]		
5	لاڑی	✓	پودہ	کھار بنتی ہے	کپڑے دھونے کیلئے	
6	گلر	✓	پتے	پیس کر شہد ملا کر کھائیں	بخار کیلئے	
7	نیم	✓	پتے	پیس کر تیل کے ساتھ بالوں پر لگائیں	بالوں کیلئے	
8	کڑتکاوا	✓	پودہ	خشک کر کے پیس کر گولی بنا لیں	کینسر کیلئے	
9	کاشو	✓	پودہ	جھاڑو کیلئے	—	
10	کیکر	✓	پھول	خشک کر کے پیس کر زیرہ + اجوائن اندر ملا کر کھائیں	کمر درد کیلئے	
11	سیوور	✓	پودہ	[illegible] اور [illegible]	الرجی کیلئے	

ETHNOBOTANICAL QUESTIONNAIRE

DATE 9-4-2022 VILLAGE کرنز

NAME زبیدہ GENDER Female

LANGUAGE Balochi TRIBE Masi

AGE 28

1. How many plants you use in your daily life? ________________

S.No	Name of Plant	Grow/collection from field/purchase from bazar/	Which Part of plant is used	Recpies	Which disease can be treated	alternative of this plant for same treatment
1	[illegible]	Collection from field	پتے	واکو بنا کر زخم پر لگائیں	زخم کیلئے	
2	کانڈیرو	-	پورا	ٹکی بنا کر کھائیں	شوگر کیلئے	
3	[illegible]	-	پتے	پانی میں بھگو کر رکھیں خشک کر کے ٹکی بنا کر کھائیں	پیٹ جلن اور ناف کیلئے	
4	[illegible]	-	لکڑی	ابال کر رس نکال کر پھر کالی چائے میں [illegible]	پیٹ کی درد اور [illegible]	
'	'	'	'	[illegible]	[illegible] کیلئے	
'	-	-	'	[illegible]		
5	[illegible]	-	پورا	بھگو کر رکھیں اور [illegible]	الرجی کیلئے	
6	[illegible] (تنجی)	-	پھل	خشک کر کے پیس کر گڑ کے ساتھ ٹکی بنائیں	بواسیر اور قبض کیلئے	
7	[illegible]	-	پھول	پھول کو پیس کر کے ٹکی بنائیں	کمر درد کیلئے	

CamScanner

ETHNOBOTANICAL QUESTIONNAIRE

DATE 4-4-2022 VILLAGE Dhadan (Dahipal)

NAME حاجرہ GENDER Female

LANGUAGE سرائیکی TRIBE [illegible]

AGE 70

1. How many plants you use in your daily life? 2

S.No	Name of Plant	Grow/collection from field/purchase from bazar/	Which Part of plant is used	Recpies	Which disease can be treated	alternative of this plant for same treatment
1	[illegible]	Purchase from Bazar P		پتے ڈال کر پانی ڈھکیں اور شہد مصری ڈال کر پی لیں	بچوں کے پیٹ درد کیلئے	-
2	اسوڑی	Bazar		[illegible] اسوڑی ڈالیں پانی اور ابالیں ساتھ میں گڑ بھی ڈالیں	ماہواری کے درد میں راحت ملتی ہے	
3	سمسوک	Bazar		سمسوک کو پانی میں ابالیں اور گڑ ڈالیں	کھانسی کیلئے خالی کھانسی کیلئے	
Zawal 4	[illegible]	Bazar	[illegible]	رات کو بھگو کر رکھیں اور صبح کو پی لیں	پیٹ درد کیلئے یرقان کیلئے بھی	
5	پنیر	Bazar	[illegible]	رات کو بھگو کر رکھیں اور صبح کو پی لیں چھان کر	Blood purification	

ETHNOBOTANICAL QUESTIONNAIRE

DATE 15-2-2022 VILLAGE بکھر ڑو

NAME نور خاتون GENDER Female

LANGUAGE Balochi TRIBE Rind

AGE 43

1. How many plants you use in your daily life? 1 [illegible]

S.No	Name of Plant	Grow/collection from field/purchase from bazar/	Which Part of plant is used	Recpies	Which disease can be treated	alternative of this plant for same treatment
1	[illegible] (کوریٹ)	Collection from field	پھول	[illegible]	بواسیر اور قبض کے لیے	-
2	مارووٹ			[illegible]	آرام کے لیے	-
3	کھٹریلی گھاس	Collection from field	Plant	گھوڑوں کی خوراک	گھوڑوں کے لیے	
4	جنگلی بیر	-	Fruit بیر	[illegible]	[illegible] اور پیٹ درد کے لیے	
5	[illegible]	-	Fruit [illegible]	سرسوں کے تیل میں [illegible]	2-3 [illegible] درد کے لیے	
6	[illegible]	-	Plant	[illegible]	[illegible] کے لیے الرجی [illegible]	
	-	-	-		-	
7	[illegible]	-	Plant	[illegible]	پیٹ درد کے لیے	
8	[illegible]	-	Leaves	[illegible]	[illegible] اور تیز بخار کے لیے	
	-	-	-	[illegible]	-	
9	[illegible]	-	Root	[illegible]	اولاد کے لیے	
10	[illegible]	-	Flowers	[illegible]	[illegible] درد کے لیے	

ETHNOBOTANICAL QUESTIONNAIRE

DATE 4-4-2022

VILLAGE Dhadar (Dachpal.

NAME قائم خاتون

GENDER Female.

LANGUAGE بلوچی

TRIBE جھلوڑ رند (میرانی)

AGE 63

1. How many plants you use in your daily life? 5

S.No	Name of Plant	Grow/collection from field/purchase from bazar/	Which Part of plant is used	Recpies	Which disease can be treated	alternative of this plant for same treatment
1	تاشں (سلاد)	Collection from field	plant	چھت میں ڈالتے ہیں		
2	جانبھو	"	تیل	تیل سے مالش کر	خون گردش کیلئے	
3	اپلٹر	"	پودا	سالن ساگ		
4	[illegible]	"	Plant	ابال کر [illegible] ملا کر [illegible] کیلئے	[illegible] بخار کیلئے	
5	[illegible]	"	plant.	جوش دے کر [illegible] ڈال کر کھاتے ہیں	جسم کی گرمائش کیلئے	
6	[illegible]	"	Plant	ابال کر کھاتے [illegible] یا کچے کھی سے ساتھ کھائیں	درد [illegible] کیلئے	
7	جنگلی [illegible]	"	Plant Seed	گرمیں ابال کر کھاتے [illegible] کر کھی میں ملا کر	بانجھ پن اور [illegible] کیلئے	
8	جنگلی [illegible]	"	Plant	ابال کر کھی ڈال کر [illegible]	پیٹ درد کیلئے بھوک کیلئے	
9	[illegible]	"	Plant	اونٹ کی خوراک		

٩

ETHNOBOTANICAL QUESTIONNAIRE

DATE 23-2-2022 VILLAGE Sibi City

NAME عائشہ GENDER Female

LANGUAGE بلوچی TRIBE خدارانی (آزانی) (Bangulzai)

AGE 69

1. How many plants you use in your daily life? 2

S.No	Name of Plant	Grow/collection from field/purchase from bazar/	Which Part of plant is used	Recipes	Which disease can be treated	alternative of this plant for same treatment
01	کانڈیرو	field	جڑ	جڑ + زیرہ + [illegible] ملا کر [illegible]	[illegible]	[illegible]
02	[illegible]	field	leaves	پتے [illegible]	Hep C	لئی.
03	[illegible]	field	first layer چھال	[illegible]	گردوں کے درد [illegible]	–
	–	–	–	[illegible]		–
	–	–	–	کھائیں [illegible]		–
4	لئی (گز)	field	پتے	[illegible]	[illegible]	–
				[illegible]		–
				[illegible] ختم ہو		–
5	[illegible]	collection from field	Plant	3-4 [illegible]	[illegible]	[illegible]
				ایک 10 [illegible]		

[illegible]

[illegible] ایک ٹولی صبح اور ایک ٹولی شام

ETHNOBOTANICAL QUESTIONNAIRE

DATE: 4-4-2022

VILLAGE: Dhadar (Dahpal)

NAME: نازو

GENDER: Female

LANGUAGE: Balochi

TRIBE: میرزئی (بلوچ) رند

AGE: 35

1. How many plants you use in your daily life? 2

S.No	Name of Plant	Grow/collection from field/purchase from bazar/	Which Part of plant is used	Recpies	Which disease can be treated	alternative of this plant for same treatment
1	(بوئے مادران) قند ریشم میں کھائے -	Purchase from Bazar	Plant	بوئے مادران، [illegible]، [illegible]، دی مسواک، [illegible]، گل کند، قند ریشم، خشک لیموں، ان سب کو مکس کر کے پھکی بنائیں	بچوں کے دست کیلئے	
2	[illegible]	Purchase Bazar Plant	[illegible]	[illegible] کا ایک پیس بیج کے ساتھ ابال کر ٹھنڈا ہونے دیں 4-5 قطرے بچے کو پلائیں -	بچوں کے پیٹ میں [illegible] کیلئے -	
3	جو (قند ریشم میں کھائے)	Collection field Plant	جو	گڑ + نمک + جو + انجیر + [illegible] بال ان سب کو مکس کر کے [illegible]	گردے کی پتھری کیلئے	-

ETHNOBOTANICAL QUESTIONNAIRE

DATE 8-4-2022 VILLAGE Kurak

NAME مریم GENDER Female

LANGUAGE Balochi TRIBE گرانی (Bangulzai)

AGE 72

1. How many plants you use in your daily life? —

S.No	Name of Plant	Grow/collection from field/purchase from bazar/	Which Part of plant is used	Recpies	Which disease can be treated	alternative of this plant for same treatment
1	نازبو	Collection from field	پودہ	پتے اور بیج کو بھگو کر رکھیں اور پانی پی لیں	معدہ اور قبض کیلئے۔	
2	آکش	/	پودہ	پیس کر ٹکی بنا کر کھائیں۔	امراض معدہ، دمہ کی بیماری کیلئے	
3	کوڑمٹ	/	پھل	گندم کے ساتھ ابال کر [illegible]	[illegible] کیلئے۔	
4	سیہور	/	پودہ	اُبال کر رکھیں اور نمک کے ساتھ پی لیں	الرجی اور گرمی دانوں کیلئے۔	
5	کنٹرٹ	/	شگوفے	جلا کر دودھ کے ساتھ ٹکی بنا کر۔	کمر درد اور ڈیلیوری کیلئے	
6	گانڈیرو	/	پودہ	ٹکی بنا کر کھائیں۔	شوگر کیلئے	
7	لئی	-	پتے	پتوں کی راکھ بنا کر زخم پر رکھیں	زخم کیلئے	
8	کھبڑ	/		بھگو کر رکھیں اور ابال کر چھان کر دیسی گھی زیرہ + اجوائن + گڑ کی ڈال کر تیار کر کے لیں	اولاد کیلئے۔	

ETHNOBOTANICAL QUESTIONNAIRE

DATE 10-2-2022 VILLAGE [illegible]

NAME [illegible] GENDER male

LANGUAGE Balochi TRIBE Malli

AGE 39

1. How many plants you use in your daily life? –

S.No	Name of Plant	Grow/collection from field/purchase from bazar/	Which Part of plant is used	Recpies	Which disease can be treated	alternative of this plant for same treatment
1	کنیر (اوجھٹ)	Collection from field	Fruit	ٹکڑے ٹکڑے کرکے اس پر گرم کر کے مالش [illegible]	مالش کیلئے	–
2	کڑوا	–	Plant	خشک کرکے پیس کر پھکی بنا کر کھائیں	[illegible] کیلئے	–
3	جنگلی کریلا	صرف معلومات ملی ہے	رس اور فروٹ	رس پیتے اور فروٹ کھاتے ہیں	شوگر کیلئے	–
4	گلر	Collection from field	پتے	پتے + آٹا + ہلدی [illegible] کر کے زخم پر رکھے	زخم کیلئے	
5	کاش	–	Plant	جھاڑو بنتا ہے	–	
6	کھو (ائی)	–	Flowers leaves + Roots	پھول پتے، جڑ [illegible] استعمال کیا جاتا ہے	دمہ کی بیماری [illegible]	
7	لاڑو	–	پودا	جلا کر راکھ بنائی جاتی ہے [illegible]	[illegible] کیلئے	
8	ناز بو	–	[illegible]	گاڑھا بنا کر عورت کو پلاتے ہیں	اولاد کیلئے	

صبح و شام [illegible]

جنگلی [illegible] ساتھ

CamScanner

ETHNOBOTANICAL QUESTIONNAIRE

DATE 12-11-2021 VILLAGE بکھر ڈگ

NAME رضا محمد GENDER male

LANGUAGE Balochi TRIBE Masti

AGE 27

1. How many plants you use in your daily life? ______

بیلی بیل
Sibi

S.No	Name of Plant	Grow/collection from field/purchase from bazar/	Which Part of plant is used	Recipes	Which disease can be treated	alternative of this plant for same treatment
✓ 1	افتیمون	باغات	مکمل برگ		سر کی خشکی معدہ	
	امر بیل -					
2	کاسنی	باغات	بیج		جگر - معدہ	
✓ 3	ناز بو	گردوں، باغات	پتے بیج		معدہ - قبض معیرہ	

CS CamScanner

m

ETHNOBOTANICAL QUESTIONNAIRE

DATE 1-1-2022 VILLAGE مرنزاني

NAME Sumaira GENDER female.

LANGUAGE Sindhi TRIBE مرنزاني

AGE 20

1. How many plants you use in your daily life? ______________

S.No	Name of Plant	Grow/collection from field/purchase from bazar/	Which Part of plant is used	Recpies	Which disease can be treated	alternative of this plant for same treatment
1	ڏنير	field+Bazar	fruit	رات تو ڀڳو ڪري رکيس صبح ڇاڻ ڪري پيس	خون جي صفائي لاءِ	
2	نم	field	پتا	راکو بنا ڪري زخم کي	زخم لاءِ	
3	[illegible]	"	پتا	ڀڳو ڪري رکيس ڦشڪر کي ڦڪي ٺاهي کائيس	پيٽ جلڻ لاءِ	
4	سيور	"	[illegible]	ڀڳو ڪري رکيس ۽ پيس	الرجي لاءِ.	
5	[illegible]	"	[illegible]	ڦشڪر ۽ سونڪ [illegible] سان گڏ ڦڪي ٺاهي کائيس.	بواسير ۽ [illegible] لاءِ	

ETHNOBOTANICAL QUESTIONNAIRE

DATE 9-12-2021 VILLAGE [illegible]

NAME Asad Ullah GENDER Male

LANGUAGE Balochi TRIBE Rind

AGE 19

1. How many plants you use in your daily life? –

S.No	Name of Plant	Grow/collection from field/purchase from bazar/	Which Part of plant is used	Recpies	Which disease can be treated	alternative of this plant for same treatment
1)	کھرغ	گاؤں / بکھرا	تنے	تنے کو آگ پر روٹی بنا کر کھانا	تمام بیماریوں کے لیے (بخار، زکام)	
2)	گرز	گاؤں / بکھرا	پتے	پیس کر [illegible] گھی میں ملا کر لگائے	کانٹے نکالنے کے لیے۔	
3)	~~[illegible]~~ کھل	گاؤں / بکھرا	تنا ~~[illegible]~~	جلا کر دھوئے میں بیٹھا جاتا ہے	الرجی کے لیے	
4	لانڑی	گاؤں / بکھرا	پورا پودہ	جلایا جاتا ہے جس سے [illegible] بناتے ہیں	کپڑے دھوئے جاتے ہیں جراثیم ختم ہوتے ہیں۔	

CamScanner

ETHNOBOTANICAL QUESTIONNAIRE

DATE 8-2-2021 VILLAGE Dahpal

NAME نیاز GENDER Male

LANGUAGE Biharchi TRIBE Bangulzai

AGE 80

1. How many plants you use in your daily life? –

S.No	Name of Plant	Grow/collection from field/purchase from bazar/	Which Part of plant is used	Recpies	Which disease can be treated	alternative of this plant for same treatment
1	آک	Collection from field	پتا	پتے کی ٹکڑی کی روٹی بنا کر کھائیں	بخار کیلئے	
2	کریڑ	/	Fruit	اچار بنتا ہے		
3	[illegible]	/	Seeds	جانوروں کیلئے	خوراک	
4	شیشم	/	leaf	پتے ابال کر سر دھو [illegible]	بالوں کیلئے	
5	[illegible]	/	Plant	[illegible]	[illegible]	
6	کنڈی	–	پتے	جانوروں کیلئے [illegible]	جانوروں کے دودھ میں اضافہ	
7	[illegible]	/	Plant	ابال کر [illegible] مالش کرتے	الرجی کیلئے	
8	کپاس	/	Seed	کوٹ کر [illegible]	ٹوٹی ہڈی	
9	گندم	/	Seed	روٹی بنتے ہیں	[illegible]	
10	تل	/	تیل	فالج کے مریض کو مالش کرتے سے فائدہ ہوتا	فالج کے مریض	

CamScanner

ETHNOBOTANICAL QUESTIONNAIRE

DATE 9-1-2022 VILLAGE Kurak

NAME Farishta. GENDER Female

LANGUAGE Bilavhi TRIBE Bangulzai

AGE 51

1. How many plants you use in your daily life? ________________

S.No	Name of Plant	Grow/collection from field/purchase from bazar/	Which Part of plant is used	Recpies	Which disease can be treated	alternative of this plant for same treatment
۱/	[illegible]		پودہ	ابال کر مصری ملا کر پیا جائے	[illegible] کے لئے	
۲/	[illegible]		پودہ	جانوروں کے لئے		
۳/	[illegible]	گاؤں / [illegible]	تنا اور پتے	[illegible] جلا کر [illegible] میں ملا کر لگائیں	[illegible] کے لئے	
۴/	[illegible]		[illegible]	پتے جلا کر [illegible] زخم پر لگایا جائے	زخم [illegible] کے لئے	
۵/	[illegible]	[illegible]	[illegible]	[illegible] کھاتے ہیں	[illegible]	

CamScanner

ETHNOBOTANICAL QUESTIONNAIRE

DATE 10-4-2022 VILLAGE Kulak

NAME [illegible] GENDER Female

LANGUAGE Brahvi TRIBE Bangulzai

AGE 29

1. How many plants you use in your daily life? -

S.No	Name of Plant	Grow/collection from field/purchase from bazar/	Which Part of plant is used	Recpies	Which disease can be treated	alternative of this plant for same treatment
1	[illegible]	/ Collection from field	پودہ	[illegible]	الرجی خارش کی مفید ہے	-
2	[illegible]	/	چھلکے	[illegible]	[illegible] کیلئے	
3	آک (اکھ)	/	پھول + پتے	تیل میں [illegible] کر [illegible] لگائیں	جسم میں درد کیلئے	
4	زہریلی بوٹی	-	[illegible]	[illegible]	[illegible]	
5	جنگلی بیر	/	بیر	[illegible]	پیٹ درد [illegible] کیلئے	
جیسٹ 6	[illegible]	/	پھل	[illegible] کھائیں	شوگر کیلئے	
7	لاڑی	/	پودہ	جلا کر راکھ بنائیں	[illegible] کیلئے	
8	لاڑو	/	پودہ	جلا کر راکھ بنائیں	[illegible] کیلئے	

CamScanner

ETHNOBOTANICAL QUESTIONNAIRE

DATE 19-1-2022 VILLAGE Talli

NAME Shah Bibi GENDER Female

LANGUAGE Shah Bibi Bsawhi TRIBE Bangulzai

AGE 51

1. How many plants you use in your daily life? کئی ہیں

S.No	Name of Plant	Grow/collection from field/purchase from bazar/	Which Part of plant is used	Recpies	Which disease can be treated	alternative of this plant for same treatment
1	لسی / لو [illegible]	Collection field other	پتے	[illegible] کوٹ (پیس) کر [illegible] میں ملا کر کھائیں	الرجی کیلئے	
2	کریلہ	〃	پھل	خشک کر کے پھل کھائیں	شوگر کیلئے	کوڑتمہ / نیم
3	[illegible]	〃	[illegible]	اچار بنتا ہے	-	
4	نیم	〃	پتے	پاؤڈر بنا کر جسم پر [illegible] دیں	گرمی دانوں کیلئے (پیٹ)	سیور
5	سیوڑ	〃	[illegible]	[illegible] رکھیں استعمال [illegible] کر کے کھائیں	گرمی دانوں کیلئے	نیم
~~6~~	~~[illegible]~~	〃		یہ پانی [illegible] جا سکتا ہے	-	
6	ارنڈ	〃	پتے	پتوں کو گھٹنوں پر باندھا جائے تو گھٹنوں کے درد [illegible]	گھٹنوں کے درد کیلئے	-
/	-	-	-	[illegible] ملتا ہے	-	-
7	[illegible]	〃	لکڑی	[illegible] لکڑی [illegible]	[illegible]	=
8	[illegible]	〃	روئی	کاروبار کیا جاتا ہے	=	=
9	ناز بو	〃	پتے	پیس کر چٹنی کے ساتھ [illegible] کھائیں	حاملہ خواتین [illegible]	=

ETHNOBOTANICAL QUESTIONNAIRE

DATE 11-2-2022 VILLAGE Khajjak

NAME Saadat GENDER female

LANGUAGE Sindhi TRIBE Safi

AGE 78

1. How many plants you use in your daily life? ________

S.No	Name of Plant	Grow/collection from field/purchase from bazar/	Which Part of plant is used	Recpies	Which disease can be treated	alternative of this plant for same treatment
1	نیم	field	بیج	بیج کو پانی کے ساتھ کھاتے ہیں	بواسیر کیلئے	
2	کیکر	field	پتے	جانوروں کی خوراک		
3	کانڈیرو	field	جڑ	[illegible] کر پیتے ہیں اور [illegible]	دردوں کیلئے	
4	کڑکاوا	"	[illegible]	چھلکی بنا کر پیتے ہیں	السر کیلئے	
5	زہریلی بوٹی	"	رس	بچھو کے کاٹے پر لگاتے ہیں	بچھو کے کاٹے کیلئے	
6	آک	"	پتے	گھٹنوں پر باندھتے	گھٹنوں کے درد کیلئے	
7	کاسنی	"	رس اور بیج		گردے اور مثانے کیلئے	

ETHNOBOTANICAL QUESTIONNAIRE

DATE 19-2-2022 VILLAGE Chandio

NAME Hajira GENDER Female

LANGUAGE Balochi TRIBE Solangi

AGE 32

1. How many plants you use in your daily life? ________________

S.No	Name of Plant	Grow/collection from field/purchase from bazar/	Which Part of plant is used	Recpies	Which disease can be treated	alternative of this plant for same treatment
1	کاشو	field	Plant		جھاڑو	
2	کڑکاوا	"	Plant		ٹکی بنا کر کھائیں	[illegible] + یرقان
3	کنر	"	پتے		ابال کر گڑ ملا کر پئیں	بخار میں
4	لاڑی	"	پتے		کاڑھا بنا کر کیڑے [illegible]	
5	لاڑو	"	پتے		کاڑھا بنا کر پیٹ [illegible]	
6	کورمٹ	"	fruit	پیس کر اس کا اوپر [illegible]	[illegible] + شوگر میں	
7	کیکر	"	پھول	[illegible]	کمر درد میں	
8	سیور	"	دھورا	کاڑھا بنا کر پئیں	گرمی میں	
9	اک	"	پانی	[illegible] لگائیں	[illegible]	

CS CamScanner

ETHNOBOTANICAL QUESTIONNAIRE

DATE 2-2-2022 VILLAGE [illegible] Sibi

NAME [illegible] GENDER Female

LANGUAGE Pashto TRIBE Basozai

AGE 52

1. How many plants you use in your daily life? ______

S.No	Name of Plant	Grow/collection from field/purchase from bazar/	Which Part of plant is used	Recpies	Which disease can be treated	alternative of this plant for same treatment
1	تنڈری	field	پتے	ابال کر پییں	معدے کیلئے	
2	[illegible]	"	پتے	پیس کر تیل میں ملا کر کان میں ڈالیں	کان درد کیلئے	
3	[illegible]	"	پتے	کوئلہ راکھ بنا کر زخم پر لگائیں	زخم کیلئے	
4	[illegible]	"	لکڑی	کوئلہ بنا کر پھوڑے پر لگائیں	پھوڑے کیلئے	
5	اک	"	پتے	گھٹنوں کے درد کیلئے	گھٹنوں کے درد کیلئے	
6	[illegible]	"	fruit	تیل میں گرم کر کے کان میں ڈالیں	کان درد کیلئے	
7	کوڑ تمہ	"	fruit	پیس کر دانت میں رکھیں	دانت درد کیلئے	
8	[illegible]	"	پورا	کھانا بنا کر [illegible]	پیٹ [illegible] کیلئے	

CamScanner

ETHNOBOTANICAL QUESTIONNAIRE

DATE: 11-4-2022 VILLAGE: Chandio

NAME: Rashid GENDER: Male

LANGUAGE: Sindhi TRIBE: Chandio

AGE: 57

1. How many plants you use in your daily life? ________________

S.No	Name of Plant	Grow/collection from field/purchase from bazar/	Which Part of plant is used	Recipes	Which disease can be treated	alternative of this plant for same treatment
1	پیپل درخت	باغات	گوند		کمر درد - جوڑ درد	
					اولاد کے لئے	
2	پیلو درخت	جنگلات	پھل - پتے		امراض معدہ	
					[illegible]	
3	[illegible] انگور -	باغات -	تخم		یرقان - [illegible] -	
					[illegible] -	
4	منڈی بوٹی	پہاڑی و میدانی علاقوں میں	مکمل بوٹی		مردانہ امراض	
					گردہ - پتھری وغیرہ	
5	[illegible]	صحرائی - ریگستان	بیج		قبض - کولیسٹرول	
	اسپغول				[illegible]	

Cultiva[illegible]

Cult[illegible]

ETHNOBOTANICAL QUESTIONNAIRE

DATE 17-2-2022 VILLAGE Luni

NAME ثمینہ GENDER Female

LANGUAGE 60 Pashto TRIBE Luni

AGE 60

1. How many plants you use in your daily life? ________________

S.No	Name of Plant	Grow/collection from field/purchase from bazar/	Which Part of plant is used	Recpies	Which disease can be treated	alternative of this plant for same treatment
1	کنڈی	field	چھلکے	بھگو کر پانی پی لیں	معدے کی جلن کیلئے	
2	گورمٹ	"	Seed	[illegible]	درد اور صفائی کیلئے	
3	نیم	"	پتے	پتوں کو توڑ کر جسم پر چھڑکے	گرمی دانوں کیلئے	
4	آک	"	پتے	گھٹنوں کا درد کیلئے	گھٹنوں کا درد کیلئے	
5	زہریلی بوٹی	"	Liquid	بچھو کے ڈنگ کے اوپر [illegible] لگائیں	بچھو کے ڈنگ کیلئے	
6	لاڑھی	"	دھورہ	ابال کر کھار بنائیں	پیٹ کے درد کیلئے	
7	ماندیلہ	"	دھورہ	ابال کر پی لیں	مہینے کا خون آنے [illegible]	

ETHNOBOTANICAL QUESTIONNAIRE

DATE 4-7-2022 VILLAGE Chandio

NAME Sadhori GENDER Female

LANGUAGE Sindhi TRIBE Abro

AGE 59

1. How many plants you use in your daily life? ________________

S.No	Name of Plant	Grow/collection from field/purchase from bazar/	Which Part of plant is used	Recipes	Which disease can be treated	alternative of this plant for same treatment
1	اک سٹ	باغات	پتے		جلدی امراض	
2	مدار	ریگستانی علاقے	پھول		امراض معدہ گیس، قبض	
3	گھاوس		ارگ		امراض گردہ، پتھری	
4	کنوار گندل	باغات، گھروں			تھیلیسیمیا	
	گھیکوار	گھر، باغات			جلدی امراض	
		پورا پودہ، گودہ			امراض نسواں، معدہ وغیرہ	

(Left margin notes: [illegible] — آک — کنر — ایلوویرا)

ETHNOBOTANICAL QUESTIONNAIRE

DATE 4-7-2022 VILLAGE Chandio

NAME Sadhori GENDER Female

LANGUAGE Sindhi TRIBE Abro

AGE 59

1. How many plants you use in your daily life? ____________

(سی
اک
گھر
ایلوویرا

S.No	Name of Plant	Grow/collection from field/purchase from bazar/	Which Part of plant is used	Recipes	Which disease can be treated	alternative of this plant for same treatment
1	ارک سٹ	باغات	پتے		جلدی امراض	
2	مدار	ریگستانی علاقے	پھول		امراض معدہ گیس، قبض	
3	[illegible]		برگ		امراض گردہ، پتھری	
4	کنوار گندل	باغات، گھروں			[illegible]	
	گھیکوار	گھر، باغات			جلدی امراض	
			پورا پودہ، گودہ		امراض نسواں معدہ وغیرہ	

ETHNOBOTANICAL QUESTIONNAIRE

DATE 3-2-2022 VILLAGE Chandio

NAME اصغر اللہ GENDER male

LANGUAGE سرائیکی TRIBE Chandio

AGE 57

1. How many plants you use in your daily life? —

S.No	Name of Plant	Grow/collection from field/purchase from bazar/	Which Part of plant is used	Recpies	Which disease can be treated	alternative of this plant for same treatment
1	اناڑی	Collection from field.	Plant	کھا رہتی ہے	[illegible] دے دوسے علاج	
2	کھبٹر	"	leaf	جانوروں علاج	خوراک	
3	کریڑ	"	Fruit	خشک کر کے کھائیں	[illegible] علاج	[illegible] + [illegible]
4	کنڈڑی	"	چھلکا	ابال کر پانی پی لیں	معدے کی جلن علاج	
5	کوڑتمہ	"	Seed	پانی سے ساتھ نگلنا	گردے اور مثانے علاج	
6	نیم	"	leaf	پتوں کو کوٹ کر جسم پر چھڑکیں	زخمی [illegible] علاج	
7	[illegible]	"	Fruit	خشک کر کے پیس بنا کر کھائیں	الٹی دست علاج	
8	[illegible] بوٹی	"	Plant water	بچھو کا ڈنگ کو لگا کر [illegible]	بچھوے ڈنگ علاج	
9	آک	"	پتے	گھٹنوں کے درد [illegible] باندھیں	گھٹنوں کے درد علاج	

ETHNOBOTANICAL QUESTIONNAIRE

DATE 19-1-2021 VILLAGE [illegible]

NAME [illegible] GENDER Female

LANGUAGE سرائیکی TRIBE [illegible] (ماچھی)

AGE 38

1. How many plants you use in your daily life? 1

S.No	Name of Plant	Grow/collection from field/purchase from bazar/	Which Part of plant is used	Recipes	Which disease can be treated	alternative of this plant for same treatment
1	[illegible]	Collection from field	Plant	ابال کر پیتے ہیں	[illegible]	-
2	[illegible]	-	leaves ~~Plant~~	3 بار [illegible] 5-5-5-5 دن [illegible]	[illegible]	
3	نیم	-	پتے	15 دن تک		
		-		[illegible]	الرجی کیلئے	
		-		[illegible]		
	نیم	-	پتے	پتوں کو ابال کر [illegible] سر کی جوئیں [illegible]	جوئیں ختم [illegible]	
4	[illegible]	/	پودا	ابال کر پلایا جاتا ہے	اولاد کیلئے	
5	[illegible]	/	Seeds بیج	بیج [illegible] اور [illegible]	اولاد کیلئے	
				[illegible] میں [illegible] کے بعد اس میں		
				دو چمچ [illegible] اور [illegible] [illegible] ابال کر [illegible]		

ETHNOBOTANICAL QUESTIONNAIRE

DATE ________________ VILLAGE ________________

NAME ________________ GENDER ________________

LANGUAGE ________________ TRIBE ________________

AGE ________________

1. How many plants you use in your daily life? ________________

S.No	Name of Plant	Grow/collection from field/purchase from bazar/	Which Part of plant is used	Recpies	Which disease can be treated	alternative of this plant for same treatment
6	سرسوں ساگ	Collection field سے	پودا	ابال کر نمک ڈال کر [illegible]	زکام کیلئے	–
7	[illegible]	/	چھلکا	[illegible]	معدے کی جلن کیلئے	/
8	[illegible]	/	پودا	[illegible]	[illegible] کیلئے	–
9	[illegible]	/	[illegible] شاخ	[illegible]	[illegible]	/
10	جو	/	[illegible]	[illegible] کیلئے		

ETHNOBOTANICAL QUESTIONNAIRE

DATE 15-12-2021 VILLAGE [illegible]

NAME غلام رسول GENDER Male

LANGUAGE Balochi TRIBE Rind

AGE 20

1. How many plants you use in your daily life? ________________

S.No	Name of Plant	Grow/collection from field/purchase from bazar/	Which Part of plant is used	Recipes	Which disease can be treated	alternative of this plant for same treatment
1	کاسنی	Collection باغات [illegible]	بیج		امراض معدہ	
2	کاکنج	پہاڑوں، میدان	تخم		امراض گردہ، مثانہ	
3	گندا خوہ / خرمل	Purchase پہاڑی و میدانی	تخم		جراثیم کش، پیٹ کے [illegible]	
4	کھلیہ۔ کریہ	ریگستان، جنگل	برگ و شگوفہ		اندرونی زخموں کے لیے، خون صاف	
5	گلاب	باغات۔ گھر	پتے		قبض، دل، دماغ، طاقت	

[illegible]

ETHNOBOTANICAL QUESTIONNAIRE

DATE 21-1-2022 VILLAGE جھلمورکی ۷

NAME عبدالکلیم GENDER male

LANGUAGE [illegible] TRIBE marri

AGE 70

1. How many plants you use in your daily life? -

S.No	Name of Plant	Grow/collection from field/purchase from bazar/	Which Part of plant is used	Recipes	Which disease can be treated	alternative of this plant for same treatment
1	کپاس	Collection from field	Seed	[illegible]	ٹوٹی ہڈی کیلئے	
				[illegible]		
	1.2(use)		روئی	[illegible]	-	-
	1.3(uses)		خشک لکڑی	آگ جلانے کے کام آتی ہے گھروں میں		
2	تل	-	تیل	دماغ کی خشکی دور کرتا ہے تیل	دماغ کیلئے	-
	-	-		ابال کر [illegible]	-	-
	1.2 (uses)		تیل	مالش کی جاتی ہے	خارش کے مرض کیلئے	-
3	مونگ	-	دال (بیج)	کھانا بنانے کیلئے	Food	-
(4)	[illegible]	-	دودھ	[illegible]		
-	-	-	-	[illegible]	[illegible]	کیکر
(5)	[illegible]	-	پتے	اونٹ کی خوراک	-	-
(6)	کیکر	-	پھول اور چھلکا	[illegible]	[illegible]	-
(7)	کیکر	-	چھلکا	[illegible]		شراب

ETHNOBOTANICAL QUESTIONNAIRE

DATE 1.3.2021　　　　VILLAGE Kurak

NAME نواب زادی　　　　GENDER Female

LANGUAGE Balochi　　　　TRIBE Bangulzai (آ نرانی)

AGE 49

1. How many plants you use in your daily life? 3

S.No	Name of Plant	Grow/collection from field/purchase from bazar/	Which Part of plant is used	Recpies	Which disease can be treated	alternative of this plant for same treatment
1	اشاری	Collection from field	Plant	جلا کر راکھ بنا کر پٹی پر لگاتے ہیں	جراثیم کش زخموں کیلئے	
2	آش	/	رس	رس کو تیل میں ملا کر بالوں پر لگا کر خشک ہونے پر دھو لیں	خشکی اور بالوں کیلئے	
/		/	/			
3	لونڑی ھٹو	/	ادرک کی طرح کھاتے ہیں	خشک کر کے پیس کر پھکی بنا کر کھائیں	زیادہ پیشاب آنا یا بچوں کا بستر گیلا کرنا	لیرے بچوں کا
4	نیم	/	Seed	بیج کو پانی کے ساتھ کھائیں	قبض کیلئے	
5	گڑکاوا	/	plant	خشک کر کے پیس کر پھکی بنائیں	کینسر کیلئے	
6	کیکر	/	پھول	پھولوں کو اکٹھا کر کے خشک کر کے پیس کر زیرہ + اجوائن اور گڑ ملا کر کھائیں	کمر درد کیلئے	
/		/	/		/	
7	آپلیٹ	/	پودا	ساگ کھانا بنائیں	/	
8	مسور کی دال	/	بیج	کھانا پکایا جاتا ہے	/	
9	پیاز	/	رس	کان درد کیلئے دو قطرے ڈالیں	کان درد میں	
10	کالو	/	پودا	کھانا بنائیں	/	

ETHNOBOTANICAL QUESTIONNAIRE

DATE 21-1-2021 VILLAGE Balcwase

NAME عبدالرشد GENDER Male

LANGUAGE Balochi TRIBE Rind

AGE 42

1. How many plants you use in your daily life? —

S.No	Name of Plant	Grow/collection from field/purchase from bazar/	Which Part of plant is used	Recpies	Which disease can be treated	alternative of this plant for same treatment
1	سرفرطی	Collection from field	دودھ	اینٹھن میں	–	–
Euphorbia 2	زہریلی بوٹی	–	رس	بچھو کے کاٹنے کی جگہ استعمال رس ڈنگ والی جگہ پر لگایا جاتا ہے	بچھو ڈنگ زہر دور	آک
/	–	–	-			
3	آک	–	رس	بچھو کے ڈنگ پر لگاتے ہیں	بچھو کے ڈنگ پر	زہریلی بوٹی
4	آک	–	پتے	پتے گھٹنوں پر لگائیں جس سے گھٹنوں کا درد کم ہوتا ہے۔	گھٹنوں کا درد پتے	–
/	–	–	–		–	–
5	کڑتاوا	/	دودھ	خشک کر کے پاؤڈر بنا کر پھکی بنا کر کھائیں	شوگر کینسر موٹاپا	–
(پیشاب پتھری) 6	چیبٹ	/	پھل	خشک کر کے پھکی بنا کر کھائیں	پیٹ درد	
7	اولٹ پیر	/	دودھ (ساگ)	کھانا بنا کر دینا	حاملہ خاتون پتے	–
8	کوہ سٹ آنکھ	/	پھل	ٹکڑے کر کے اس پر گوندھنا	کرمانکس پتے	–
9	نیم	/	پتے اور بیج	بیج پانی کے ساتھ کھانا پتے اور بیج کی پاؤڈر بنا کر	شوگر + خون کی صفائی پیٹ کے کیڑے	
10	جانتجبو	–	دودھ	جانوروں پیٹ خوراک	–	–

صبح و شام ایک چمچ

ETHNOBOTANICAL QUESTIONNAIRE

DATE 15-2-2022　　VILLAGE Sibi City

NAME زاہرہ　　GENDER Female

LANGUAGE بلوچی　　TRIBE [illegible] (شنکزئی)

AGE 73

1. How many plants you use in your daily life? 2

S.No	Name of Plant	Grow/collection from field/purchase from bazar/	Which Part of plant is used	Recipes	Which disease can be treated	alternative of this plant for same treatment
1	1.1 ترٹ	Collection from field	تکری / شاخیں	[illegible]	پھوڑے / دانہ	-
〃	〃	〃	چھلکا	چھلکا پانی میں ڈال کے [illegible]	-	-
〃	1.2 〃	〃	〃	[illegible]	-	-
〃	〃	〃	〃	[illegible] تین بار [illegible]	-	-
〃	〃	〃	〃	10-15 دن تک	-	-
2	سیور	〃	دودھ	[illegible]	-	کھجوری / نیم
〃	〃	〃	〃	[illegible] لگاتے ہیں [illegible]	-	
3	نیم	〃	پتے	[illegible]	-	سیور / کھجوری
.	〃	〃	〃	کر کے اس پانی سے	-	
	〃	〃		نہایا جاتا ہے۔		〃
(4) (البرابوی)	[illegible] / خلری / [illegible]	〃	پھول	[illegible] میں استعمال کیا جاتا ہے۔	[illegible]	-

ETHNOBOTANICAL QUESTIONNAIRE

DATE: 10-2-2021 VILLAGE: [illegible]

NAME: [illegible] GENDER: male

LANGUAGE: ✓Balochi + Sindhi TRIBE: Rind

AGE: 51

1. How many plants you use in your daily life? –

S.No	Name of Plant	Grow/collection from field/purchase from bazar/	Which Part of plant is used	Recpies	Which disease can be treated	alternative of this plant for same treatment
1	نیم	Collection from field	leaves	[illegible] پتے [illegible] اور اس پانی سے [illegible]	الرجی کیلئے	
2	[illegible]	/	رس	بچھو کے ڈنک [illegible] کرتا ہے	بچھو کے ڈنک کو	
3	آک	/	پتے + رس	[illegible]	[illegible] کیلئے	
4	ریم یلی بوٹی	/	رس	بچھو کے ڈنک کیلئے [illegible]	بچھو کے ڈنک کیلئے	
5	کانڈیرو	/	Root	[illegible]	امراض نسواں کیلئے	
6	[illegible]	/	wood	[illegible]	[illegible] درد کیلئے	
7	[illegible]	/	چھلکے	[illegible]	[illegible]	
8	[illegible]	/	leaves	[illegible]	[illegible] کیلئے	
9	لانڑی	/	Plant	جلا کر [illegible]	[illegible]	
10	[illegible]	/	Plant	[illegible]	[illegible] کیلئے	
11	[illegible]	/	Plant	[illegible] پیس کر [illegible] بنا کر کھائیں	[illegible]	[illegible]
12	[illegible]	/	[illegible]	پتے جلا کر [illegible]	[illegible] کیلئے	
13	کپاس	/	Seed	[illegible]	[illegible] کیلئے	

CamScanner

ETHNOBOTANICAL QUESTIONNAIRE

DATE 02-08. 2021 VILLAGE مل

NAME فاطمہ لال GENDER Female

LANGUAGE بلوچی TRIBE لنڈ

AGE 55

1. How many plants you use in your daily life? ________________

S.No	Name of Plant	Grow/collection from field/purchase from bazar/	Which Part of plant is used	Recipes	Which disease can be treated	alternative of this plant for same treatment
1	گندم	کھیت وزرہ	بیج، خوشہ اور مرکب		[illegible] ملیریا بخار [illegible]	[illegible]
2	سرکاری کیکر ببول	جنگلات، باغات	پتے، گوند، چھال، پھلیاں، پھول		مردانہ امراض، [illegible] امراض [illegible]	
3	[illegible]	ریگستان اور [illegible] میں	پھل		امراض معدہ پیٹ	

[illegible] (margin notes)

ETHNOBOTANICAL QUESTIONNAIRE

DATE 19-1-2022 VILLAGE Chandio

NAME فوزیہ GENDER Female

LANGUAGE Sindhi (Siraiki) TRIBE Chandio

AGE 42

1. How many plants you use in your daily life? –

S.No	Name of Plant	Grow/collection from field/purchase from bazar/	Which Part of plant is used	Recpies	Which disease can be treated	alternative of this plant for same treatment
1	سکوٹ	Collection field from	leaves	پتے اچھے سے کوٹ کر جڑوں پر لگائیں بال کی	بالوں کیلئے	
2	پیاز	〃	رس	رس کی مالش کریں سر کی جڑوں میں	بالوں کیلئے	
3	کھجور کے پتے	〃	پتے	پتوں کو بھگو کر رکھیں [illegible] اور پی لیں	الرجی کیلئے خارش کیلئے	
4	[illegible]	〃	[illegible]	[illegible]	[illegible] کیلئے	
〃	〃	〃	〃	[illegible]	[illegible]	
5	سرسوں	〃	[illegible]	[illegible]	[illegible]	
6	جو	〃	[illegible]	بکریوں کے کھانے [illegible]	Fodder	
7	بیر	〃	پتے	[illegible]	[illegible]	
8	[illegible]	〃	[illegible]	سرسوں کے تیل میں [illegible] کان میں ڈالیں	کان درد کیلئے	
9	[illegible]	〃	تیل	تیل سر پر لگانے سے جوئیں اور خشکی ختم ہو جاتی ہے	[illegible] کیلئے	
10	[illegible]	〃		پیس کر [illegible] بنا کر [illegible] کھائیں	سوزش کیلئے	
	[illegible]	〃		پیس کر دانت پر رکھیں	دانت درد کیلئے	

ETHNOBOTANICAL QUESTIONNAIRE

DATE 26-3-2022 VILLAGE [illegible]

NAME [illegible] GENDER Female

LANGUAGE Balochi TRIBE [illegible]

AGE 26

1. How many plants you use in your daily life? -

S.No	Name of Plant	Grow/collection from field/purchase from bazar/	Which Part of plant is used	Recpies	Which disease can be treated	alternative of this plant for same treatment
1	کنڈی	Collection from field	چھلکے	اُبال کر پیس	معدے علاج	
2	[illegible]	-	پتے	[illegible]	[illegible]	
3	[illegible]	-	پودہ	[illegible] ساتھ کھائیں	درد [illegible]	
4	[illegible]	-	چھلکے	پیس کر [illegible] کان میں ڈالیں	کان درد علاج	
5	[illegible]	-	لکڑی	کوئلہ بنا کر [illegible] پر لگائیں	[illegible] علاج	
6	[illegible]	-	پتے	کوئلہ [illegible] زخم پر لگائیں	زخم علاج	

CamScanner

ETHNOBOTANICAL QUESTIONNAIRE

DATE 28-2-2022 VILLAGE Talli

NAME کعتری بی بی GENDER Female

LANGUAGE Balochi TRIBE Silachi

AGE 52

1. How many plants you use in your daily life? ________________

S.No	Name of Plant	Grow/collection from field/purchase from bazar/	Which Part of plant is used	Recpies	Which disease can be treated	alternative of this plant for same treatment
1	کھبش	Collection from field	پتے	اونٹ کی خوراک		
2	ایپلکر	/	پھول	ابال کر پی لیں	پتھری اور کمر درد	
3	لش	/	پتے	تیل میں تڑکا دے کر لگائیں جسم پر	بڑے بخار کے لیے	
4	کرنڈ	/	لکڑی	کوئلہ بنا کر اسے پھوڑوں پر لگائیں	پھوڑے کے لیے	
5	نیم	/	پتوں	پتوں کو ابال کر سر کے بالوں کی جڑوں پر لگائیں	جوئیں ختم ہو جاتی ہیں	
6	نازبو	/	بیج	ابال کر پیئیں	نزلہ زکام کے لیے	
7	پھوسڑی	/	پودا	بکریوں کی خوراک		
8	آک	/	پودا	زہریلا پودا ہے		

CamScanner

ETHNOBOTANICAL QUESTIONNAIRE

DATE 21-3-2021 VILLAGE گل

NAME موگل GENDER Female

LANGUAGE Balochi TRIBE شلواری

AGE 74

1. How many plants you use in your daily life? —

	S.No	Name of Plant	Grow/collection from field/purchase from bazar/	Which Part of plant is used	Recpies	Which disease can be treated	alternative of this plant for same treatment
آک.	1	کوخ	collection field from	رس پتے پھول	گھٹنوں پر باندھے	گھٹنوں کے درد کیلئے۔	
	2	کڑکاوا	/	[illegible]	[illegible]	شوگر اور کینسر کیلئے	
	3	زنجو	/	Seed	پانی کے ساتھ بیج نگل لے	قبض کیلئے۔	
پہاڑی زیرا	4	پیریخ	/	رس	رس نکال کر پئیں	شوگر کیلئے	
جنگلی بیر	5	کھنتر	/	بیر	[illegible]	پیٹ درد [illegible] کیلئے	
پیاز	6	پیماز	/	رس	رس نکال کر کان میں ڈالیں	کان درد کیلئے	
	7	کاسنی	/	رس اور بیج		گردے اور [illegible] کیلئے۔	

CamScanner

ETHNOBOTANICAL QUESTIONNAIRE

DATE 14-2-2022 VILLAGE Sibi.

NAME M. Asif GENDER Male.

LANGUAGE Sindhi TRIBE Soomro.

AGE 38.

1. How many plants you use in your daily life? –

S.No	Name of Plant	Grow/collection from field/purchase from bazar/	Which Part of plant is used	Recpies	Which disease can be treated	alternative of this plant for same treatment
01	بسلوٹ	Collection from field	Seed	جانوروں کے لیے [illegible] استعمال ہوتا	–	–
2	پیاز	〃	پیاز	رس	کان کے درد کے لیے	–
3	[illegible]	〃	دال	کھانا بنانے اور جانوروں کے لیے استعمال ہوتا ہے	کھانے کے لیے	
		〃	.		–	
4	سورج مکھی	〃	تیل	کھانا بنانے کے لیے	–	
5	کھجور	〃	پتے	[illegible] بنانے	[illegible] (چٹائی)	(
6	کیکر	〃	چھلکا	ابال کر جو پانی [illegible] ایک ایک چمچ [illegible]	امراض مسوڑوں	
	〃	〃	〃			
7	[illegible]	〃	[illegible] پتے	[illegible] کرکے زخم پر رکھے	زخم کے لیے	
8	[illegible] (نیم)		بیج	دن میں ایک بیج کھائیں	بواسیر، بلڈ پریشر	
9	[illegible]		[illegible]	کاڑھا بنا کر پیئیں	گرمی سے حفاظت	

ETHNOBOTANICAL QUESTIONNAIRE

DATE 1-2-2022

VILLAGE بکھرڑو

NAME شیخ محمد

GENDER male

LANGUAGE Balochi

TRIBE البڑو

AGE 53

1. How many plants you use in your daily life? —

	S.No	Name of Plant	Grow/collection from field/purchase from bazar/	Which Part of plant is used	Recpies	Which disease can be treated	alternative of this plant for same treatment
	1	گھوڑیلی گھاڑ	Collection from field	Plant	گھوڑوں کی خوراک ہے	-	
	2	کرٹ	/	wood	کوئلہ بنا کر پھوڑے پر لگائے	پھوڑے علاج	
	3	بشی	/	پتے	[illegible] کر جلا کر جسم پر لگائے	الرجی علاج	
	4	وزیلی بوٹی	^	رس	بچھو کے کاٹنے والے جگہ پر لگائیں	بچھو کے ڈنگ سے	
		/	: /	^	عرق [illegible] کا کام [illegible]	^	
	5	کھبڑ	^	پتے	جانوروں کی خوراک	-	
	6	کانڈیرو	—	جڑ	بھگو کر دیں اور پی لیں	زردی علاج	
(نیم بیج)	7	نیموری	—	Seed	بیج پانی کے ساتھ کھائے	بواسیر علاج	
	8	لڑکاوا	/	Plant	پیس کر کھائیں	شوگر علاج	

CamScanner

ETHNOBOTANICAL QUESTIONNAIRE

DATE 19-1-2021 VILLAGE Khajjak

NAME فریدہ GENDER female

LANGUAGE پشتو TRIBE Khajjak

AGE 75

1. How many plants you use in your daily life? 1

S.No	Name of Plant	Grow/collection from field/purchase from bazar/	Which Part of plant is used	Recpies	Which disease can be treated	alternative of this plant for same treatment
1	کانڈیرو	Collection from field	Plant	دیگی بنا کر کھائیں	شوگر کیلئے	
2	کیکر	〃	〃	پھلیاں اُبالیں آئی میں گندم میں اُبالیں اس کے بعد پھلیاں گندم کو کوٹ لیں پھکی کی طرح بنائیں اور کیکر کا پھول ملا لیں اور گڑ کے ستو بنا کر کھائیں۔	کمر درد کیلئے	
3	[illegible]	〃	پتے	رات کو بھگو کر رکھیں صبح اُبال کر [illegible] [illegible] [illegible] [illegible] [illegible]	امراض نسواں کیلئے، بانجھ پن کیلئے	
4	لئی	〃	پتے	پتوں کی راکھ کو بنا کر زخم پر رکھیں زخم ٹھیک ہو جاتا ہے۔	پرانے زخم کیلئے	
5	نیم	〃	Seed	کوٹ کر مکھن میں ڈالیں اور کھائیں	بواسیر کیلئے	
6	نیم	〃	Root	جڑ کو خشک کر کے اُبالیں اور اس کا پانی بالوں کیلئے استعمال کریں	بال گرنے اور [illegible]	

ETHNOBOTANICAL QUESTIONNAIRE

DATE ____________ VILLAGE ____________

NAME ____________ GENDER ____________

LANGUAGE ____________ TRIBE ____________

AGE ____________

1. How many plants you use in your daily life? ____________

S.No	Name of Plant	Grow/collection from field/purchase from bazar/	Which Part of plant is used	Recpies	Which disease can be treated	alternative of this plant for same treatment
7	لاڑو	Collection from field	Plant	جلا کر راکھ بنا کر لگایا جاتا ہے [illegible]	پیٹ کے درد میں جراثیم کش	لاڑھی
8	نازبو	-	-	کاڑھا بنا کر قہوہ [illegible] پلایا جاتا ہے	[illegible]	-
9	کوڑتمہ	-	Fruit	[illegible] ٹکڑے کر کے [illegible]	شوگر [illegible]	نیم
-	-	-	-	کھانے سے شوگر ختم ہو جاتی ہے	-	
10	[illegible]	-	تیل	تیل کی مالش کر کے جسم پر الرجی [illegible]	الرجی، خارش	-
11	بیر	-	Fruit	کھانے کیلئے	-	-
12	بیر	-	پتے	پانی میں ابالیں اور پانی کو بالوں پر [illegible] کی طرح [illegible] اور پھر [illegible]	بال [illegible] ہونگے	-
-	-	-	-		-	-

ETHNOBOTANICAL QUESTIONNAIRE

DATE 20-1-2022 VILLAGE Killi Hanbi

NAME سالار حنیف GENDER Male

LANGUAGE سرائیکی TRIBE بانجھی

AGE 45

1. How many plants you use in your daily life? ________________

S.No	Name of Plant	Grow/collection from field/purchase from bazar/	Which Part of plant is used	Recpies	Which disease can be treated	alternative of this plant for same treatment
1	کھوغ (آدھم)	Collection from field	leaf	سرسوں کے تیل میں پتے کو گرم کر کے سر پر مالش کرنا	سردی کا سر درد کا علاج	
2	بیر جنگلی	~	leaf	پاؤڈر بنا کر پھکی بنائیں	گیس اور قبض اور پیٹ	
[illegible] 3	پہاڑی کریلا		کریلا	کچا کریلا یا رس	شوگر کیلئے	
4	کھمبی		رس	آنکھوں کے امراض	آنکھوں کیلئے	
5	کیکر	collection from field	leaf	کوٹ کر بالوں پر لگائیں۔	بالوں کیلئے۔	
6	سہوور	~	Plant	کاڑھا بنا کر پئیں	گر مالش کیلئے۔	
7	مشوش	~	leaf	کوٹ کر بالوں پر لگائیں	بال کالے کرنے	
8	نیم	/	Seed	صبح کھانے سے پہلے پانی کے ساتھ	بواسیر اور پیٹ سوجن میں راحت ہے	

CamScanner
